# An annotated checklist of the eukaryotic parasites of humans, exclusive of fungi and algae

**DOI:** 10.3897/zookeys.1069.67403

**Published:** 2021-11-09

**Authors:** Blaine A. Mathison, Sarah G. H. Sapp

**Affiliations:** 1 Institute for Clinical and Experimental Pathology, ARUP Laboratories, Salt Lake City, UT, USA Institute for Clinical and Experimental Pathology Salt Lake City United States of America; 2 Parasitic Diseases Branch, Division of Parasitic Diseases and Malaria, Centers for Disease Control and Prevention, Atlanta, GA, USA Centers for Disease Control and Prevention Atlanta United States of America

**Keywords:** Acanthocephalans, arthropods, cestodes, leeches, nematodes, parasitology, protozoa, trematodes

## Abstract

The classification of “parasites” in the medical field is a challenging notion, a group which historically has included all eukaryotes exclusive of fungi that invade and derive resources from the human host. Since antiquity, humans have been identifying and documenting parasitic infections, and this collective catalog of parasitic agents has expanded considerably with technology. As our understanding of species boundaries and the use of molecular tools has evolved, so has our concept of the taxonomy of human parasites. Consequently, new species have been recognized while others have been relegated to synonyms. On the other hand, the decline of expertise in classical parasitology and limited curricula have led to a loss of awareness of many rarely encountered species. Here, we provide a comprehensive checklist of all reported eukaryotic organisms (excluding fungi and allied taxa) parasitizing humans resulting in 274 genus-group taxa and 848 species-group taxa. For each species, or genus where indicated, a concise summary of geographic distribution, natural hosts, route of transmission and site within human host, and vectored pathogens are presented. Ubiquitous, human-adapted species as well as very rare, incidental zoonotic organisms are discussed in this annotated checklist. We also provide a list of 79 excluded genera and species that have been previously reported as human parasites but are not believed to be true human parasites or represent misidentifications or taxonomic changes.

## Introduction

The online Oxford English dictionary defines a parasite as: ‘an organism that lives in or on an organism of another species (its host) and benefits by deriving nutrients at the other’s expense’ (LEXICO 2020). From a strictly biological perspective, this definition can cover a wide variety of organisms, including, but not limited to, bacteria, viruses, fungi, helminths (worms), protozoans, cnidarians, tardigrades, rotifers, mollusks, and arthropods. However, in the historical concept of the medical community, the term ‘parasite’ refers to animals and other eukaryotic organisms with animal-like affinities, and is usually limited to the protozoans, helminths, and arthropods.

Historically, human parasites have been classified within the broad Linnaean kingdom of ‘Animals’ and divided into taxa with formal categorical names (phylum, class, order, family, etc.). While traditional Linnaean taxa are still commonly used for helminths and arthropods, it is becoming more common to use clade-based systems devoid of formal designations, especially with the taxa collectively called protozoans. Much of this work comes from a better understanding of the evolutionary relationships of organisms based on both morphological and molecular analyses (Adl and Mathison 2019).

Currently, eukaryotes are classified into two domains, Amorphea and Diaphoretickes, as well as additional large clades that do not fit into either of those domains. Within domains are large clades commonly referred to as supergroups. Eukaryotic parasitic organisms fall into five supergroups within these domains: SAR, Archaeplastida, Excavates, Amoebozoa, and Opisthokonta (Adl et al. 2019; Adl and Mathison 2019). For the purpose of this work, the focus is on the traditional human parasitic protozoans (Amoebozoa, SAR, Excavates) and helminths, annelids, and arthropods (all in Opisthokonta). Two groups of organisms that were historically considered protozoans, but are now considered fungi and are not included here, are *Pneumocystisjirovecii* Frenkel, 1999 (syn. P.carinii f. sp. hominis) and the microsporidia. The genus *Prototheca* is another opportunistically parasitic eukaryote, but is currently aligned with the algae in the clade Archaeplastida and is also not covered here (Lass-Flörl and Mayr 2007). For detailed descriptions on the higher classifications of eukaryotes, the readers are referred to the work by Adl and colleagues (Adl et al. 2019).

The Amoebozoa include many of the human parasites historically referred to as amebae, including the intestinal amebae *Entamoeba*, *Endolimax*, and *Iodamoeba*, and several of the ‘free-living’ amebae such as *Balamuthia* and *Acanthamoeba* (Adl and Mathison 2019). These organisms feed by phagocytosis and move by pseudopods. They lack cilia and most have an environmentally hardy stage (cyst) for dispersal and infection of a new host (Adl et al. 2019; Adl and Mathison 2019). The free-living amebae are not adapted for true parasitism and are only opportunistic parasites when conditions allow for colonization of the human host. Clinically, *Acanthamoeba* species are usually characterized by their genotypes rather than traditional Linnaean binominal nomenclature (Booton et al. 2005).

The SAR (which commonly refers to Stramenopiles-Alveolates-Rhizaria) includes parasitic coccidians (e.g., *Toxoplasma*, *Cyclospora*, *Cystoisospora*), gregarines (*Cryptosporidium*), piroplasmids (*Babesia*), haemosporidians (*Plasmodium*), ciliophores (*Balantioides*, formerly *Balantidium*), and the stremenopile *Blastocystis* (Adl and Mathison 2019).

Within the SAR, the coccidians, gregarines, piroplasmids, and hemosporidians are obligate parasites and part of the clade Apicomplexa that are characterized by a structure called an apical complex that is used for penetration of the host cell. Motility is usually by gliding or body flexion and feeding is by pinocystosis (ingestion by budding of small vesicles from the cell membrane). Apicomplexans may have one or two hosts and reproduce by sexual reproduction, the end result of which is an oocyst that when mature contains sporozoites that are the infectious stage for a new host (Adl et al. 2019; Adl and Mathison 2019). The Haemospororida (e.g., *Plasmodium*) and Piroplasmorida (e.g., *Babesia*) are vectored by sanguinivorous arthropods. Humans are intermediate hosts for *Plasmodium*, which are vectored by mosquitoes that serve as the definitive hosts. *Babesia* are zoonotic in humans and in nature typically cycle between a tick vector (definitive host) and a mammalian intermediate host (Adl and Mathison 2019).

The only human parasitic ‘ciliate’ is *Balantioides* (formerly *Balantidium*, syn. *Neobalantidium*). Movement is by numerous cilia and feeding is by phagocytosis. It forms an environmentally-hardy cyst that is the infectious stage for a new host (Adl et al. 2019; Adl and Mathison 2019).

*Blastocystis* is an enigmatic organism that has historically been variably classified among coccidians, fungi, and algae. The life cycle is still not completely understood, but it has been proposed that it reproduces by binary fission of vacuolar forms within the host and infects a new host via environmentally hardy cysts. Both vacuolar forms and cysts can be detected in stool specimens, and the variability in morphology of the former has created numerous descriptive terms. It has also been suggested it has a third form, an amoeboid form, that is responsible for pathogenesis in the host, but this hypothesis is not yet broadly accepted (Tan 2008). As the molecular epidemiology is becoming better understood, it is becoming less common to recognize *Blastocystis* by traditional Linnaean binominal nomenclature and more common to base identification on their genetic subtypes (Stensvold et al. 2007; Tan 2008).

The Excavates includes the traditional ‘flagellates’, such as *Giardia*, *Trypanosoma*, *Dientamoeba*, *Chilomastix*, *Leishmania*, and *Trichomonas*, as well as amoeboflagellates, such as *Naegleria* (Adl and Mathison 2019). All excavates move by cilia (historically referred to as flagella, which are not analogous to the bacterial flagellum) at some point in their life cycles. Most feed by pinocytosis (although the ameboid form of *Naegleria* and its relatives feed by phagocytosis). The life cycle patterns vary greatly among the excavates. Intestinal flagellates typically multiply by binary fission in the lumen of the intestine of their hosts and have an environmentally hardy cyst stage that is infectious for a new host. The trichomonads lack a cyst stage, and *Dientamoeba* is unusual in that it lacks external cilia and moves by pseudopods and feeds by phagocytosis, much like the Amoebozoa. The ‘hematoflagellates’ (*Leishmania*, *Trypanosoma*, *Crithidia*, *Endotrypanum*) have two host life cycles and are transmitted to the human host via an arthropod vector. Amoeboflagellates, such as *Naegleria*, *Paravahlkampfia*, *Tetramitus*, *Vahlkampfia*, *Allovahlkampfia* are not true parasites and only infect the human host when opportunities allow for infection and colonization (Adl et al. 2019; Adl and Mathison 2019).

The Opisthokonta includes the arthropods, leeches, and helminths, all within the clade Metazoa (Adl and Mathison 2019). The term ‘helminth’ is not a taxonomic unit in any classification scheme, but instead a basket term for parasitic worms of humans, which is divided into four broad categories: cestodes (tapeworms), trematodes (flukes), nematodes (roundworms), and acanthocephalans (thorny-headed worms) (Adl and Mathison 2019). Leeches (Annelida), while not historically considered helminths are blood-feeding animals that will opportunistically feed on humans. Within the Metazoa are the Spiralia (acanthocephalans, annelids, cestodes, trematodes) and Ecdysozoa (arthropods and nematodes).

The cestodes are obligate parasites that usually reside in the small intestine of their definitive host. Most have multiple-host life cycles, and the definitive host becomes infected after ingestion of a larval stage encysted in the tissues of an intermediate host. Adults are long, ribbon-like organisms that have three main body regions: scolex (for attachment to the intestinal mucosa of the definitive host), neck (the base of the strobila), and the strobila (the main body, which is made of up individual segments called proglottids). Historically, cestodes that parasitize humans were divided into two categories: Pseudophyllidea (*Dibothriocephalus*, *Adenocephalus*, *Diphyllobothrium*), and Cyclophyllidea (*Taenia*, *Dipylidium*, *Hymenolepis*, several others). However, Pseudophyllidea has been abandoned in favor of two broad groups, Bothriocephalidea and Diphyllobothriidea, the latter of which contains human parasites (Kuchta et al. 2008). From the perspective of species that parasitize humans, Diphyllobothriidea are characterized by adult worms that attach to the intestine of the definitive host by means of bothria (grooves in the scolex). Eggs have an operculum and are unembryonated when shed in feces, and the infectious stage for the definitive host is the plerocercoid larva (e.g., sparganum). The Cyclophyllidea however attach to the intestine of their definitive hosts by means of four suckers and (often) a rostellum, that may be armed with hooklets or not. Eggs lack an operculum, are shed embryonated, and contain a hooked oncosphere that is immediately infectious for an intermediate host. The infectious larva is highly variable and terminology is often taxon-dependent (cysticercus, hydatid cyst, tetrathyridium, etc.) (Chervy 2002; Adl and Mathison 2019).

The trematodes that infect humans are all within Digenea and are also obligate parasites with multi-host life cycles. All parasitic species of humans have a snail as a first intermediate host, and many digeneans have second intermediate hosts or paratenic hosts that may comprise a variety of animals; in some species, infective stages are encysted in the environment, including on the surface of plants. Adults are usually hermaphroditic, apart from the schistosomes which are dioecious but live *in copula*. Adults have two muscular suckers, an oral sucker for attachment and feeding and a ventral sucker (acetabulum) for attachment. Infection of the human host is either by direct penetration of the cercaria larval stage (*Schistosoma*) or ingestion of the metacercaria larval stage in an intermediate animal host or on contaminated plants (Adl and Mathison 2019).

The acanthocephalans are zoonotic parasites in humans. While superficially similar to nematodes, they are more closely related to the rotifers, or possibly nested within Rotifera, proper (Garcia-Varela et al. 2000). Adults are large, pseudocoelomate animals that attach to the intestinal mucosa of the definitive host be means of a heavily armed proboscis. Human infection usually occurs from the ingestion of an infected intermediate or paratenic host (Mathison et al. 2016). The leeches (Annelida: Hirudinea) include blood-feeding members that will opportunistically feed on humans. Most are aquatic and attacks on humans usually come from swimming, fishing, and wading in fresh water.

The nematodes are a large and diverse group of organisms, most of which are free-living, although some major parasitic lineages are of great public health importance. Despite their diversity in nature, few species parasitize humans with any regularity. Two major clades of human parasites are Dorylaimia and Chromadoria, which vary greatly in their biology, route of transmission, and morphology. Most nematodes have six developmental stages: egg, four larval stages (L1-L4), and adult (L5); some larviparous species do not lay eggs. For most parasitic nematodes of humans, the infectious stage is the L3 larva, this fact is often called the ‘Rule of the Infective Third State’. Notable exceptions are members of the Trichinellida, for which the infectious stage is the L1 larva, and *Eustrongylides* for which the infectious stage is believed to be the precocious L4 (Anderson 2000). Most intestinal nematodes infect the human host after ingestion of fully embryonated eggs in food, water, or fomites contaminated with feces, or after ingestion of an infected intermediate or paratenic host. L3 larvae of some species, such as *Strongyloides* and the hookworms, can penetrate intact human skin during walking on contaminated soil. Filarial nematodes (Spirudida: Filarioidea) are vectored by sanguinivorous arthropods (Ash and Orihel 2007; Adl and Mathison 2019; Carroll et al. 2019).

The arthropods are the largest group of Metazoa, but relatively few species are adapted to parasitism on humans. Two main clades are the Chelicerata and Mandibulata, which can be broadly separated by the structure of their mouthparts. The Chelicerata includes the mites and ticks. The Mandibulata includes Pancrustacea, which contains the clades Crustacea (crustaceans) and Hexapoda (insects). The only parasitic crustaceans of humans are zoonotic pentastomids (tongueworms). The pancrustacean clade Hexapoda includes parasitic insects such as lice, fleas, beg bugs, triatomine (kissing) bugs, and myiasis-causing flies. Arthropods are characterized by a jointed exoskeleton containing chitin. Development is variable and can be gradual (hemimetabolous) or complete (holometabolous). Holometabolous insects have morphologically and biologically markedly different life cycles stages including larvae, pupae, and adults (Mathison and Pritt 2014; Mathison and Pritt 2015; Adl and Mathison 2019).

Here we provide an updated checklist of the parasitic protozoans, helminths, annelids, and arthropods of humans, nearly 20 years since the last such endeavor (Ashford and Crewe 2003), while providing an updated higher taxonomy based on advances in eukaryotic taxonomy (Adl et al. 2019). Taxonomy and systematics are largely ignored in the education of medical and public health professionals. There are frequent updates to taxonomic changes in Medical Parasitology (Simner 2017; Adl and Mathison 2019; Mathison and Pritt 2019; Mathison and Pritt 2020; Mathison et al. 2021), but taxonomy remains a challenging discipline in the medical and public health realms. We also provide annotations on the geographic distribution, host range, route of infection, and anatomic site of infection for human eukaryotic parasites.

## Materials and methods

The bulk of this manuscript is an annotated checklist of protozoan, helminthic, annelid, and arthropod parasites reported from the human host, based on extensive literature searches through April 2021. A systematic literature search of the PubMed database (U.S. National Library of Medicine National Institutes of Health; https://www.ncbi.nlm.nih.gov/pubmed), Google Scholar (https://scholar.google.com/) and Google (https://www.google.com) was also performed using the keyword search phrases using various taxa in combination with human infection and case reports. We aimed to include all protozoan and helminthic parasites known to be reported from humans. The leeches and arthropods, however, provided a special challenge, as an exhaustive list of every sanguinivorous species that may feed on a human host would be large and beyond the scope of the audience. For the arthropods, the focus is on ectoparasites that spend prolonged time on the human host (lice, sarcoptid and demodecid mites, hard ticks, myiasis-causing flies, *Tunga* fleas) and visceral parasites (pentastomids). For leeches and arthropods that are short-term blood feeders or biters, such as non-*Tunga* fleas, soft ticks, zoonotic biting mites, bed bugs, and triatomine bugs, the focus is on those species that are commonly associated with humans and/or vector medically-important pathogens. Sanguinivorous flies (mosquitoes, black flies, deer flies, etc.) are not included, nor are those flies and other insects (e.g., cockroaches) that may passively transmit pathogens. Arthropods that are considered medically important due to envenomation, stings, or urticarial and allergic reactions are also not included.

Suprageneric taxa are listed in a hierarchal system lacking formal designations, following Adl et al. (Adl et al. 2019). Each suprageneric taxon in indicated by a series of icons (●) rather than using traditional Linnaean categories such as order, family, etc. Each taxon has one more icon than the preceding taxon in which it belongs. The number of clades presented varies based on the diversity within a given clade in relation to human parasites.

For organisms traditionally classified as protozoans (Amoebozoa, Excavates, and SAR), clades above the traditional ‘family’ level follow Adl and colleagues (Adl et al. 2019). The family-group taxa (typically ending in -idae) are presented based on the current classifications of the given organisms. For the helminths and arthropods, the organisms considered ‘true’ animals (Opisthokonta), the classification follows Adl and colleagues (Adl et al. 2019) to the level Holozoa, after which clades follow the current classifications for the given organisms. Genera within a family-group taxon, and species within each genus, are presented alphabetically.

Species, and genera for which isolates from human cases have not been characterized at the species level, have at least four bullet-points of information: 1) Geographic distribution, 2) Natural hosts, 3) Route of infection, and 4) Site in/on human host. In addition, many of the arthropods and leeches also have an additional bullet-point, 5) Vectored pathogens.

**Geographic distribution.** This is the known geographic distribution of a given species or genus. For widespread organisms, the distribution is presented generally (e.g., Worldwide, North America, Circumtropical). For organisms that are more geographically restricted, the information is presented at the country level.

**Natural hosts.** These are the hosts that are part of the parasite’s natural life cycle. For multi-host parasites, the hosts are presented in developmental order, starting with the first intermediate host and finishing with the definitive host. Hosts for species or genera with broad host ranges are presented generally (e.g., mammals, carnivores, birds), while hosts for parasites that are more host specific are presented at a more granular level (e.g., mosquitoes in the genus *Anopheles*).

**Route of infection.** This is route through which the human host becomes infected or colonized.

**Site in/on human host.** This is where the parasite resides in the human host, and includes both the typical site as well as common sites of ectopic infection.

**Vectored pathogens.** This bullet-point is for all the arthropods, except for the pentastomids and most of the myiasis-causing fly larvae, and leeches. It provides information on infectious agents (bacteria, viruses, parasites) that are pathogenic for the human host that are transmitted by the given arthropod. It includes agents that are believed to be vectored by a given arthropod in a natural setting and does not include agents that have simply been detected in an arthropod using molecular surveillance or in experimental models.

For all taxonomic levels, select synonyms are presented, especially for names that show up frequently in the literature regarding human cases.

Lastly, a list of excluded species is presented. These are species that have been reported as human parasites but are either not believed to be true parasites in the human host, represent data based on misidentifications, or are invalid names. The excluded species are presented alphabetically.

## Checklist of eukaryotic human parasites

### Amoebozoa Lühe, 1913, amend Cavalier-Smith, 1998

#### ● Tubulinea Smirnov et al., 2005

##### ●● Echinamoebida Cavalier-Smith in Cavalier-Smith et al. 2004

**Genus *Vermamoeba* Smirnov et al., 2011**

***Vermamoebavermiformis* (Page, 1967)**

**Geographic distribution.** Worldwide (Scheid 2019).

**Natural host.** None; occurs in natural freshwater environments, surface water, soil, and biofilms. Humans are incidental hosts (Scheid 2019; Scheid et al. 2019; Siddiqui et al. 2021).

**Route of infection.** Presumably environmental exposure into eyes, mucus membranes, and wounds (Abedkhojasteh et al. 2013; Scheid 2019; Scheid et al. 2019).

**Site in human host.** Skin and soft tissues, eye (Scheid et al. 2019).

**Notes.** Previous records of *Harmannella* from human clinical specimens probably refer to this species.

##### ●● Elardia Kang et al., 2017

●●● **Euamoebida Lepşi, 1960 sensu Smirnov et al., 2011**

●●●● **Hartmannellidae Volkonsky, 1931**

**Genus *Hartmannella* Alexeiff, 1912**

**Geographic distribution.** Worldwide.

**Natural host.** None (environmental); humans are incidental hosts (Bradbury 2014).

**Route of infection.** Unknown; presumed environmental exposure and contamination (Bradbury 2014).

**Site in human host.** Intestinal tract (Bradbury 2014).

**Notes.** Most extraintestinal reports of human infection with *Hartmanella* apply to *H.vermiformis* Page, which is now in the genus *Vermamoeba*.

#### ● Evosea Kang et al., 2017

##### ●● Archamoeba Cavalier-Smith, 1993 sensu Cavalier-Smith et al., 2004

●●● **Entamoebida Cavalier-Smith, 1993**

●●●● **Entamoebidae Chatton, 1925**

**Genus *Endolimax* Kuenen & Swellengrebel, 1913**

***Endolimaxnana* (Wenyon et O’Connor, 1917)**

**Geographic distribution.** Worldwide (Poulsen and Stensvold 2016).

**Natural host.** Humans (Poulsen and Stensvold 2016).

**Route of infection.** Ingestion of mature cysts in fecally contaminated water, food, fomites(Poulsen and Stensvold 2016).

**Site in human host.** Large intestine, cecum, colon (Poulsen and Stensvold 2016).

**Genus *Entamoeba* Casagrandi & Barbagallo, 1895**

***Entamoebabangladeshi* Royer et al., 2012**

**Geographic distribution.** Southeast Asia, sub-Saharan Africa (Hooshyar et al. 2015; Ngobeni et al. 2017).

**Natural host.** Humans (Hooshyar et al. 2015).

**Route of infection.** Ingestion of mature cysts in fecally contaminated water, food, fomites (Ali 2015).

**Site in human host.** Large intestine, cecum, colon (Ali 2015).

***Entamoebacoli* (Grassi, 1879)**

*Entamoebahominis* Casagrandi & Barbagallo, 1897

*Entamoebaloeschi* Lesage, 1908

*Councilmanialafleuri* Kofoid & Swezy, 1921

**Geographic distribution.** Worldwide (Hooshyar et al. 2015).

**Natural hosts.** Humans, monkeys (Hooshyar et al. 2015).

**Route of infection.** Ingestion of mature cysts in fecally contaminated water, food, fomites (Ali 2015).

**Site in human host.** Large intestine, cecum, colon (Ali 2015).

***Entamoebachattoni* Swellengrebel, 1914**

**Geographic distribution.** Africa (Verweij et al. 2001; Hooshyar et al. 2015).

**Natural hosts.** Non-human primates. Zoonotic in humans (Sargeaunt et al. 1992; Hooshyar et al. 2015).

**Route of infection.** Ingestion of mature cysts in fecally contaminated water, food, fomites (Sargeaunt et al. 1992).

**Site in human host.** Large intestine, cecum, colon (Sargeaunt et al. 1992).

***Entamoebadispar* Brumpt, 1925**

**Geographic distribution.** Worldwide (Ali 2015; Hooshyar et al. 2015).

**Natural hosts.** Humans, chimpanzee, baboons, macaques (Ali 2015; Hooshyar et al. 2015).

**Route of infection.** Ingestion of mature cysts in fecally contaminated water, food, fomites (Ali 2015).

**Site in human host.** Large intestine, cecum, colon (Ali 2015).

***Entamoebagingivali* s (Gros, 1849)**

*Amoebabuccalis* Steinberg, 1862

*Amoebadentalis* Grassi, 1879

*Amoebakartulis* Doflein, 1901

*Entamoebamaxillaris* Kartulis, 1906

*Entamoebacanibuccalis* Smith, 1938

*Endamoebaconfusa* Craig, 1916

*Entamoebaequibuccalis* Simitch, 1938

*Entamoebasuigingivalis* Tumka, 1959

**Geographic distribution.** Worldwide (Hooshyar et al. 2015).

**Natural hosts.** Humans, horses, pigs, cats, monkeys (Hooshyar et al. 2015).

**Route of infection.** Person-to-person contact (Bonner et al. 2018; Bradbury et al. 2019b).

**Site in human host.** Oral cavity; ectopic colonization of urogenital tract (Cembranelli et al. 2013; Bonner et al. 2018; Bradbury et al. 2019b; Dubar et al. 2020).

***Entamoebahartmanni* Von Prowazek, 1912**

*Entamoebahistolytica*, small race

*Entamoebaminuta* Woodcock & Penfold, 1916

*Entamoebatenuis* Kuenen & Swellengrebel, 1917

*Entamoebaminutissima* Brug, 1917

**Geographic distribution.** Worldwide (Hooshyar et al. 2015).

**Natural host.** Humans (Hooshyar et al. 2015).

**Route of infection.** Ingestion of mature cysts in fecally contaminated water, food, fomites (Ali 2015).

**Site in human host.** Large intestine, cecum, colon (Ali 2015).

***Entamoebahistolytica* Schaudinn, 1903**

*Amoebacoli* Lösch, 1875

*Amoebadysenteriae* Councilman & Lafleur, 1891

*Entamoebaafricana* Hartmann & Von Prowazek, 1907

*Entamoebaschaudinii* Lesage, 1908

*Entamoebaminuta* Elmassian, 1909

*Entamoebanipponica* Koidzumi, 1909

*Entamoebabrasiliensis* Aragao, 1912

*Entamoebavenaticum* Darling, 1915

*Entamoebacaudata* Carini & Reichenow, 1949

**Geographic distribution.** Worldwide; endemic areas of clinical amebiasis include Central and South America, Africa, Southeast Asia (Ali 2015; Shirley et al. 2018).

**Natural host.** Humans (Hooshyar et al. 2015).

**Route of infection.** Ingestion of mature cysts in fecally contaminated water, food, fomites; oral-anal sex (Shirley et al. 2018).

**Site in human host.** Large intestine, cecum, colon; ectopic colonization of liver, skin, lungs, brain (Ali 2015; Mathison and Pritt 2018; Shirley et al. 2018).

***Entamoebamoshkovskii* (Tshalaia, 1941)**

*Entamoebahistolytica*, Laredo strain

*Entamoebahistolytica*, Huff strain

**Geographic distribution.** Presumed worldwide; high areas of endemicity include Australia, India, Bangladesh, Tanzania, Pakistan, Iran (Ali 2015; Kyany’a et al. 2019).

**Natural host.** Humans (Hooshyar et al. 2015; Lopez et al. 2015).

**Route of infection.** Ingestion of mature cysts in fecally contaminated water, food, fomites (Ali 2015).

**Site in human host.** Large intestine, cecum, colon (Ali 2015).

***Entamoebanuttalli* (Castellani, 1908)**

*Loeschiaduboscqi* Mathis, 1913

*Amoebaateles* Eichhorn & Gallagher, 1916

*Loeschiacynomolgi* Brug, 1923

**Geographic distribution.** Native to Southeast Asia, probably worldwide in zoos and from the animal trade (Tanaka et al. 2019).

**Natural hosts.** Non-human primates. Zoonotic in humans (Hooshyar et al. 2015; Levecke et al. 2015).

**Route of infection.** Ingestion of mature cysts in fecally contaminated water, food, fomites.

**Site in human host.** Large intestine, cecum, colon (Levecke et al. 2015)

***Entamoebapolecki* (Von Prowazek, 1912)**

*Entamoebadebliecki* Nieschulz, 1923

**Geographic distribution.** Worldwide; spots of high endemicity include Venezuela, Iran, Southeast Asia, Papua New Guinea (Verweij et al. 2001; Stensvold et al. 2018).

**Natural hosts.** Pigs, monkeys. Zoonotic in humans (Hooshyar et al. 2015).

**Route of infection.** Ingestion of mature cysts in fecally contaminated water, food, fomites (Ali 2015).

**Site in human host.** Large intestine, cecum, colon (Ali 2015).

**Genus *Iodamoeba* Dobell, 1919**

***Iodamoebabuetschlii* (Von Prowazek, 1912)**

**Geographic distribution.** Worldwide (Ash and Orihel 2007).

**Natural host.** Human (Ash and Orihel 2007).

**Route of infection.** Ingestion of mature cysts in fecally contaminated water, food, fomites (Ash and Orihel 2007).

**Site in human host.** Large intestine, cecum, colon (Ash and Orihel 2007).

#### ● Discosea Cavalier-Smith et al., 2004

##### ●● Flabellinia Smirnov et al., 2005

●●● **Thecamoebida Schaeffer, 1926, emend. Smirnov et al., 2011**

●●●● **Thecamoebidae Schaeffer, 1926, emend. Smirnov et al., 2011**

**Genus *Sappinia* Dangeard, 1896**

***Sappiniapedata* Dangeard, 1896**

**Geographic distribution.** Presumed worldwide (Visvesvara 2013).

**Natural host.** None (environmental); humans are incidental hosts

**Route of infection.** Presumably by the entry of cysts or trophozoites through mucous membranes (e.g., nasal passages) or broken skin (Qvarnstrom et al. 2009; Visvesvara 2013).

**Site in human host.** Central nervous system (CNS) (Qvarnstrom et al. 2009; Visvesvara 2013).

##### ●● Centramoebia Cavalier-Smith et al., 2016

●●● **Acanthopodida Page, 1976**

●●●● **Acanthamoebidae Sawyer & Griffin, 1975**

**Genus *Acanthamoeba* Volonsky, 1931**

**Notes.** Clinically, *Acanthamoeba* species are usually characterized by their genotypes rather than traditional Linnaean binominal nomenclature (Booton et al. 2005).

***Acanthamoeba* Genotype T1**

**Geographic distribution.** Presumed worldwide (Booton et al. 2005).

**Natural host.** None (environmental); humans are incidental hosts.

**Route of infection.** Entry of environmental trophozoites or cysts through mucous membranes (e.g., eye, nasal passages) or broken skin (Booton et al. 2005; Khan 2006).

**Site in human host.** CNS (Booton et al. 2005; Khan 2006).

***Acanthamoeba* Genotype T2**

**Geographic distribution.** Presumed worldwide (Booton et al. 2005).

**Natural host.** None (environmental); humans are incidental hosts.

**Route of infection.** Entry of environmental trophozoites or cysts through mucous membranes (e.g., eye, nasal passages) (Booton et al. 2005; Khan 2006).

**Site in human host.** Eye (Khan 2006).

***Acanthamoeba* Genotype T3**

**Geographic distribution.** Presumed worldwide (Booton et al. 2005).

**Natural host.** None (environmental); humans are incidental hosts.

**Route of infection.** Entry of environmental trophozoites or cysts through mucous membranes (e.g., eye, nasal passages) (Booton et al. 2005; Khan 2006).

**Site in human host.** Eye (Booton et al. 2005; Khan 2006).

***Acanthamoeba* Genotype T4**

**Geographic distribution.** Worldwide (Booton et al. 2005).

**Natural host.** None (environmental); humans are incidental hosts.

**Route of infection.** Entry of environmental trophozoites or cysts through mucous membranes (e.g., eye, nasal passages) or broken skin (Booton et al. 2005; Khan 2006).

**Site in human host.** Eye, skin, lung, brain, sinus cavity, disseminated infection (Booton et al. 2005; Khan 2006).

***Acanthamoeba* Genotype T5**

**Geographic distribution.** Presumed worldwide (Booton et al. 2005).

**Natural host.** None (environmental); humans are incidental hosts.

**Route of infection.** Entry of environmental trophozoites or cysts through mucous membranes (e.g., eye, nasal passages) (Ledee et al. 2009).

**Site in human host.** Eye (Ledee et al. 2009).

***Acanthamoeba* Genotype T6**

**Geographic distribution.** Presumed worldwide (Booton et al. 2005).

**Natural host.** None (environmental); humans are incidental hosts.

**Route of infection.** Entry of environmental trophozoites or cysts through mucous membranes (e.g., eye, nasal passages) (Booton et al. 2005; Khan 2006).

**Site in human host.** Eye (Booton et al. 2005; Khan 2006).

***Acanthamoeba* Genotype T8**

**Geographic distribution.** Presumed worldwide (Booton et al. 2005).

**Natural host.** None (environmental); humans are incidental hosts

**Route of infection.** Entry of environmental trophozoites or cysts through mucous membranes (e.g., eye, nasal passages) (Orosz et al. 2018).

**Site in human host.** Eye (Orosz et al. 2018).

***Acanthamoeba* Genotype T10**

**Geographic distribution.** Presumed worldwide (Booton et al. 2005).

**Natural host.** None (environmental); humans are incidental hosts

**Route of infection.** Entry of environmental trophozoites or cysts through mucous membranes (e.g., eye, nasal passages) or broken skin (Booton et al. 2005; Khan 2006; Nuprasert et al. 2010).

**Site in human host.** CNS, eye (Booton et al. 2005; Khan 2006; Nuprasert et al. 2010).

***Acanthamoeba* Genotype T11**

**Geographic distribution.** Presumed worldwide (Booton et al. 2005).

**Natural host.** None (environmental); humans are incidental hosts.

**Route of infection.** Entry of environmental trophozoites or cysts through mucous membranes (e.g., eye, nasal passages) (Booton et al. 2005; Khan 2006).

**Site in human host.** Eye (Booton et al. 2005; Khan 2006).

***Acanthamoeba* Genotype T12**

**Geographic distribution.** Presumed worldwide (Booton et al. 2005).

**Natural host.** None (environmental); humans are incidental hosts.

**Route of infection.** Entry of environmental trophozoites or cysts through mucous membranes (e.g., eye, nasal passages) or broken skin (Booton et al. 2005; Khan 2006).

**Site in human host.** CNS (Booton et al. 2005; Khan 2006).

***Acanthamoeba* Genotype T13**

**Geographic distribution.** Presumed worldwide (Booton et al. 2005).

**Natural host.** None (environmental); humans are incidental hosts.

**Route of infection.** Entry of environmental trophozoites or cysts through mucous membranes (e.g., eye, nasal passages) (Grün et al. 2014)

**Site in human host.** Eye (Grün et al. 2014).

***Acanthamoeba* Genotype T15**

**Geographic distribution.** Presumed worldwide (Booton et al. 2005).

**Natural host.** None (environmental); humans are incidental hosts.

**Route of infection.** Entry of environmental trophozoites or cysts through mucous membranes (e.g., eye, nasal passages) (Di Cave et al. 2009).

**Site in human host.** Eye (Di Cave et al. 2009).

●●●● **Balamuthiidae Cavalier-Smith et al., 2004**

**Genus *Balamuthia* Visvesvara et al., 1993**

***Balamuthiamandrillaris* Visvesvara et al., 1993**

**Geographic distribution.** Worldwide (Visvesvara 2013; Cope et al. 2019).

**Natural host.** None (environmental); humans are incidental hosts.

**Route of infection.** Entry of environmental trophozoites or cysts through mucous membranes (e.g., eye, nasal passages) or broken skin (Visvesvara 2013; Cope et al. 2019).

**Site in human host.** Disseminated infection with predilection for CNS (Visvesvara 2013; Cope et al. 2019).

### SAR (Stremanopiles-Alveolata-Rhizaria) Burki et al., 2008, emend. Adl et al., 2012

#### ● Stramenopiles Patterson 1989, emend. Adl et al., 2005

##### ●● Bigyra Cavalier-Smith, 1998, emend. Cavalier-Smith et al., 2006

●●● **Opalozoa Cavalier-Smith, 1991, emend. Cavalier-Smith et al., 2006**

●●●● **Opalinata Wenyon, 1926, emend. Cavalier-Smith, 1997**

**Genus *Blastocystis* Alexeieff, 1911**

**Notes.** As the molecular epidemiology is becoming better understood, it is becoming less common to recognize *Blastocystis* by traditional Linnaean binominal nomenclature but rather by their genetic subtypes (Stensvold et al. 2007; Tan 2008).

***Blastocystis* subtype 1**

**Geographic distribution.** Worldwide (Stensvold et al. 2007; Tan 2008).

**Natural hosts.** Various mammals. Zoonotic in humans (Stensvold et al. 2007; Tan 2008).

**Route of infection.** Ingestion of cyst-forms and/or vacuolar-forms in contaminated food, water, fomites (Tan 2008).

**Site in human host.** Large intestine (Tan 2008).

***Blastocystis* subtype 2**

**Geographic distribution.** Worldwide (Stensvold et al. 2007; Tan 2008).

**Natural hosts.** Non-human primates, pigs. Zoonotic in humans (Stensvold et al. 2007; Tan 2008).

**Route of infection.** Ingestion of cyst-forms and/or vacuolar-forms in contaminated food, water, fomites (Tan 2008).

**Site in human host.** Large intestine (Tan 2008).

***Blastocystis* subtype 3**

**Geographic distribution.** Worldwide (Stensvold et al. 2007; Tan 2008).

**Natural hosts.** Humans (Stensvold et al. 2007; Tan 2008).

**Route of infection.** Ingestion of cyst-forms and/or vacuolar-forms in contaminated food, water, fomites (Tan 2008).

**Site in human host.** Large intestine (Tan 2008).

***Blastocystis* subtype 4**

**Geographic distribution.** Worldwide (Stensvold et al. 2007; Tan 2008).

**Natural hosts.** Rodents. Zoonotic in humans (Stensvold et al. 2007; Tan 2008).

**Route of infection.** Ingestion of cyst-forms and/or vacuolar-forms in contaminated food, water, fomites (Tan 2008).

**Site in human host.** Large intestine (Tan 2008).

***Blastocystis* subtype 5**

**Geographic distribution.** Worldwide (Stensvold et al. 2007; Tan 2008).

**Natural hosts.** Pigs, cattle. Zoonotic in humans (Stensvold et al. 2007; Tan 2008).

**Route of infection.** Ingestion of cyst-forms and/or vacuolar-forms in contaminated food, water, fomites (Tan 2008).

**Site in human host.** Large intestine (Tan 2008).

***Blastocystis* subtype 6**

**Geographic distribution.** Worldwide (Stensvold et al. 2007; Tan 2008).

**Natural hosts.** Birds. Zoonotic in humans (Stensvold et al. 2007; Tan 2008).

**Route of infection.** Ingestion of cyst-forms and/or vacuolar-forms in contaminated food, water, fomites (Tan 2008).

**Site in human host.** Large intestine (Tan 2008).

***Blastocystis* subtype 7**

**Geographic distribution.** Worldwide (Stensvold et al. 2007; Tan 2008).

**Natural hosts.** Birds. Zoonotic in humans (Stensvold et al. 2007; Tan 2008).

**Route of infection.** Ingestion of cyst-forms and/or vacuolar-forms in contaminated food, water, fomites (Tan 2008).

**Site in human host.** Large intestine (Tan 2008).

***Blastocystis* subtype 8**

**Geographic distribution.** Worldwide (Stensvold et al. 2007; Tan 2008).

**Natural hosts.** Monkeys, birds. Zoonotic in humans (Stensvold et al. 2007; Tan 2008).

**Route of infection.** Ingestion of cyst-forms and/or vacuolar-forms in contaminated food, water, fomites (Tan 2008).

**Site in human host.** Large intestine (Tan 2008).

***Blastocystis* subtype 9**

**Geographic distribution.** Worldwide (Stensvold et al. 2007; Tan 2008).

**Natural hosts.** Unknown; presumed zoonotic in humans (Stensvold et al. 2007; Tan 2008).

**Route of infection.** Ingestion of cyst-forms and/or vacuolar-forms in contaminated food, water, fomites (Tan 2008).

**Site in human host.** Large intestine (Tan 2008).

***Blastocystis* subtype 12**

**Geographic distribution.** Worldwide (Stensvold et al. 2007; Tan 2008).

**Natural hosts.** Goats, cattle. Zoonotic in humans (Stensvold et al. 2007; Tan 2008; Udonsom et al. 2018; Jiménez et al. 2019).

**Route of infection.** Ingestion of cyst-forms and/or vacuolar-forms in contaminated food, water, fomites (Tan 2008).

**Site in human host.** Large intestine (Tan 2008).

#### ● Alveolata Cavalier-Smith, 1991

##### ●● Colpodellida Cavalier-Smith, 1993, amend. Adel et al. 2005, 2019

●●● **Colpodellidae Simpson & Patterson, 1996**

**Genus *Colpodella* Cienkowski, 1865**

**Geographic distribution.** Worldwide (Getty et al. 2021).

**Natural hosts.** None; free-living predators of other protozoans. Humans are incidental hosts (Getty et al. 2021).

**Route of infection.** Unknown, proposed transmission via the bite of ticks in the genus *Ixodes* (Yuan et al. 2012; Jiang et al. 2018).

**Site in human host.** Blood (Yuan et al. 2012; Jiang et al. 2018).

**Notes.** In nature, *Colpodella* species are free-living predators of other protozoans. *Copodella*-like organisms have been diagnosed twice in patients from China, one from blood (Yuan et al. 2012) and once from CSF (Jiang et al. 2018); only the former also demonstrated morphologic evidence of a potential parasite. In both cases, the identifications were made by sequencing analyses. The species-level identification was not made in either case, and the organisms in at least one of the cases (Yuan et al. 2012) might represent an as-of-yet undescribed genus. Further work is needed to confirm the ability of *Colpodella* and related organisms to cause parasitic infections in humans.

##### ●● Apicomplexa Levine, 1970, emend. Adl et al. 2005

●●● **Aconoidasida Mehlhorn et al., 1980**

Hematazoa Vivier, 1982

●●●● **Haemospororida Danilewsky, 1885**

●●●●● **Plasmodiidae Mesnil, 1903**

**Genus *Plasmodium* Marchiafava & Celli, 1885**

***Plasmodiumbrasilianum* Gonder et von Berenberg-Gossler, 1908**

**Geographic distribution.** Brazil, Venezuela, Colombia , Panama, Peru (Coatney et al. 1971).

**Natural hosts.** Intermediate hosts are New World monkeys. Arthropod vector and definitive hosts are mosquitoes in the genus *Anopheles*. Zoonotic in humans as an intermediate host (Coatney et al. 1971; Lalremruata et al. 2015).

**Route of infection.** Introduction of sporozoites via the bite of infected *Anopheles* mosquito (Coatney et al. 1971).

**Site in human host.** Liver (initial infection), blood (Coatney et al. 1971).

**Notes.** It has been suggested that *P.brasilianum* is conspecific with *P.malariae* and that *P.malariae* adapted to non-human primates in Latin America after being introduced from Africa (Guimaraes et al. 2012).

***Plasmodiumcoatneyi* Eyles et al., 1962**

**Geographic distribution.** Peninsular Malaysia, Philippines (Coatney et al. 1971).

**Natural hosts.** Intermediate host is the crab-eating macaque (*Macacafascicularis*). Arthropod vector and definitive hosts are mosquitoes in the genus *Anopheles*. Zoonotic in humans as an intermediate host (Coatney et al. 1971; Yap et al. 2021).

**Route of infection.** Introduction of sporozoites via the bite of infected *Anopheles* mosquito (Coatney et al. 1971).

**Site in human host.** Liver (initial infection), blood (Coatney et al. 1971).

***Plasmodiumcynomolgi* Mayer, 1907**

**Geographic distribution.** Southeast Asia (Coatney et al. 1971).

**Natural hosts.** Intermediate hosts are macaques. Arthropod vector and definitive hosts are mosquitoes in the genus *Anopheles*. Zoonotic in humans as an intermediate host (Coatney et al. 1971; Hartmeyer et al. 2019; Yap et al. 2021).

**Route of infection.** Introduction of sporozoites via the bite of infected *Anopheles* mosquito (Coatney et al. 1971).

**Site in human host.** Liver (initial infection, chronic sequestration), blood (Coatney et al. 1971).

***Plasmodiumfalciparum* (Welch, 1897)**

*Oscillariamalariae* Laveran, 1881

*Haemamoebapraecox* Feletti & Grassi, 1890

*Haemamoebaimmaculata* Grassi, 1891

*Haemamoebalaverani* Labbe, 1894

*Haemosporidiumsedecimanae* Lewkowicz, 1897

*Haemosporidiumvigesimotertianae* Lewkowicz, 1897

**Geographic distribution.** Circumtropical; sub-Saharan Africa, Asia, Latin America, Caribbean (Snow et al. 2005; Weiss et al. 2019).

**Natural hosts.** Intermediate hosts are humans. Arthropod vector and definitive hosts are mosquitoes in the genus *Anopheles* (Coatney et al. 1971).

**Route of infection.** Introduction of sporozoites via the bite of infected *Anopheles* mosquito (Coatney et al. 1971).

**Site in human host.** Liver (initial infection), blood, CNS (cerebral malaria) (Coatney et al. 1971; Idro et al. 2010).

***Plasmodiuminui* Helberstaedter & Von Prowazek, 1907**

**Geographic distribution.** Southeast Asia (Coatney et al. 1971).

**Natural hosts.** Intermediate hosts are Old World monkeys. Arthropod vector and definitive hosts are mosquitoes in the genus *Anopheles*. Zoonotic in humans as an intermediate host (Coatney et al. 1971; Yap et al. 2021).

**Route of infection.** Introduction of sporozoites via the bite of infected *Anopheles* mosquito (Coatney et al. 1971).

**Site in human host.** Liver (initial infection), blood (Coatney et al. 1971; Liew et al. 2021).

***Plasmodiumknowlesi* Sinton & Mulligan, 1932**

**Geographic distribution.** Southeast Asia (Coatney et al. 1971).

**Natural hosts.** Intermediate hosts are macaques. Arthropod vector and definitive hosts are mosquitoes in the genus *Anopheles*. Zoonotic in humans as an intermediate host (Coatney et al. 1971; Singh and Daneshvar 2013; Yap et al. 2021).

**Route of infection.** Introduction of sporozoites via the bite of infected *Anopheles* mosquito (Coatney et al. 1971).

**Site in human host.** Liver (initial infection), blood (Coatney et al. 1971).

***Plasmodiummalariae* (Grassi & Feletti, 1890)**

*Plasmodiumquartanae* Celli & Sanfelice, 1891

*Haematosporidiumtertianae* Labbe, 1894

**Geographic distribution.** Sub-Saharan Africa, Southeast Asia, Indonesia, Pacific Islands, Amazonian South America (Coatney et al. 1971; Collins and Jeffery 2007).

**Natural hosts.** Intermediate hosts are humans; non-human primates in Latin America are reservoir hosts. Arthropod vector and definitive hosts are mosquitoes in the genus *Anopheles* (Coatney et al. 1971; Collins and Jeffery 2007).

**Route of infection.** Introduction of sporozoites via the bite of infected *Anopheles* mosquito (Coatney et al. 1971; Collins and Jeffery 2007).

**Site in human host.** Liver (initial infection), blood (Coatney et al. 1971; Collins and Jeffery 2007).

***Plasmodiumovalecurtisi* Sutherland et al., 2010**

**Geographic distribution.** Western sub-Saharan Africa, Southeast Asia (Coatney et al. 1971).

**Natural hosts.** Intermediate hosts are humans. Arthropod vector and definitive hosts are mosquitoes in the genus *Anopheles* (Coatney et al. 1971; Collins and Jeffery 2005; Oguike et al. 2011).

**Route of infection.** Introduction of sporozoites via the bite of infected *Anopheles* mosquito (Coatney et al. 1971; Collins and Jeffery 2005).

**Site in human host.** Liver (initial infection, chronic sequestration), blood (Coatney et al. 1971; Collins and Jeffery 2005).

**Notes.** With the separation of *P.ovale* into the subspecies *P.ovalecurtisi* and *P.o.wallikeri*, the epidemiology of the two species in not well understood. Both subspecies are sympatric at the country level in several African countries, but their individual distributions in Southeast Asia are not well defined (Oguike et al. 2011).

***Plasmodiumovalewallikeri* Sutherland et al., 2010**

**Geographic distribution.** Western sub-Saharan Africa, Southeast Asia (Coatney et al. 1971).

**Natural hosts.** Intermediate hosts are humans. Arthropod vector and definitive hosts are mosquitoes in the genus *Anopheles* (Coatney et al. 1971; Collins and Jeffery 2005; Oguike et al. 2011).

**Route of infection.** Introduction of sporozoites via the bite of infected *Anopheles* mosquito (Coatney et al. 1971; Collins and Jeffery 2005).

**Site in human host.** Liver (initial infection, chronic sequestration), blood (Coatney et al. 1971; Collins and Jeffery 2005).

**Notes.** With the separation of *P.ovale* into the subspecies *P.ovalecurtisi* and *P.o.wallikeri*, the epidemiology of the two species in not well understood. Both subspecies are sympatric at the country level in several African countries, but their individual distributions in Southeast Asia are not well defined (Oguike et al. 2011).

***Plasmodiumschwetzi* Brumpt, 1938**

**Geographic distribution.** Tropical Africa (Coatney et al. 1971).

**Natural hosts.** Intermediate hosts are gorillas and chimpanzees. Arthropod vector and definitive hosts are mosquitoes in the genus *Anopheles*. Zoonotic in humans as intermediate hosts (Contacos et al. 1970; Coatney et al. 1971).

**Route of infection.** Introduction of sporozoites via the bite of infected *Anopheles* mosquito (Coatney et al. 1971).

**Site in human host.** Liver (initial infection, presumed chronic sequestration), blood (Coatney et al. 1971).

***Plasmodiumsimiovale* Dissanaike, Nelson & Garnham, 1965**

**Geographic distribution.** Sri Lanka, Malaysia (Coatney et al. 1971).

**Natural hosts.** Intermediate hosts is the toque macaque (*Macacasinica*). Arthropod vector and definitive hosts are mosquitoes in the genus *Anopheles*. Zoonotic in humans as intermediate hosts (Coatney et al. 1971; Yap et al. 2021).

**Route of infection.** Introduction of sporozoites via the bite of infected *Anopheles* mosquito (Coatney et al. 1971).

**Site in human host.** Liver (initial infection, chronic sequestration), blood (Coatney et al. 1971; Yap et al. 2021).

***Plasmodiumsimium* Fonseca, 1951**

**Geographic distribution.** Brazil (Coatney et al. 1971).

**Natural hosts.** Intermediate hosts are howler monkeys and capuchins. Arthropod vector and definitive hosts are mosquitoes in the genus *Anopheles*. Zoonotic in humans as intermediate hosts (Coatney et al. 1971; Brasil et al. 2017).

**Route of infection.** Introduction of sporozoites via the bite of infected *Anopheles* mosquito (Coatney et al. 1971).

**Site in human host.** Liver (initial infection, chronic sequestration), blood (Coatney et al. 1971; Brasil et al. 2017).

**Notes.** It has been suggested *P.simium* is conspecific with *P.vivax*, and that *P.vivax* adapted to non-human primates in Latin America after being introduced by humans (Daron et al. 2020).

***Plasmodiumvivax* (Grassi & Feletii, 1890)**

*Haemamoebamalariae* Feletti & Grassi, 1890, in part

*Haemosporidiumtertianae* Lewkowicz, 1897

*Plasmodiumcamarense* Ziemann, 1915

**Geographic distribution.** Africa (east, Horn of Africa, and Madagascar), Central and South America, Central Asia, Indian Subcontinent, Southeast Asia, Korean Peninsula (Coatney et al. 1971; Howes et al. 2016).

**Natural hosts.** Intermediate hosts are humans; non-human primates can serve as reservoir hosts. Arthropod vector and definitive hosts are mosquitoes in the genus *Anopheles* (Coatney et al. 1971).

**Route of infection.** Introduction of sporozoites via the bite of infected *Anopheles* mosquito (Coatney et al. 1971).

**Site in human host.** Liver (initial infection, chronic sequestration), blood, CNS (Coatney et al. 1971; Sarkar and Bhattacharya 2008; Mukhtar et al. 2019).

●●●● **Piroplasmorida Wenyon, 1926**

●●●●● **Babesiidae Poche, 1913**

**Genus *Babesia* Starcovivi, 1893**

***Babesiadivergens* M’Fadyean & Stockman, 1911**

**Geographic distribution.** Europe (Ord and Lobo 2015)

**Natural hosts.** Intermediate hosts are primarily cattle. Arthropod vector and definitive hosts are ticks in the genus *Ixodes*, primarily *I.ricinus*. Zoonotic in humans as intermediate hosts (Ord and Lobo 2015; Kukina et al. 2018, 2019).

**Route of infection.** Introduction of sporozoites via the bite of the *Ixodes* tick vector; person-to-person transmission via blood and solid organ transplants (Ord and Lobo 2015; Kukina et al. 2018, 2019).

**Site in human host.** Blood (Ord and Lobo 2015; Kukina et al. 2018, 2019).

***Babesiaduncani* Conrad et al., 2006**

*Babesia* strain WA1

*Babesia* strain WA2

*Babesia* strain CA5

*Babesia* strain CA6

**Geographic distribution.** Western and northern North America (Conrad et al. 2006; Ord and Lobo 2015).

**Natural hosts.** Intermediate hosts are suspected as being mule deer (*Odocoileushemionus*). Arthropod vector and definitive host is believed to be the tick *Dermacentoralbipictus*. Zoonotic in humans (Swei et al. 2019).

**Route of infection.** Introduction of sporozoites via the bite of the presumptive tick vector; possibly also via blood transfusion and solid organ transplants (Conrad et al. 2006).

**Site in human host.** Blood.

***Babesiamicroti* (França, 1910)**

**Geographic distribution.** Northeastern North America, Australia (Senanayake et al. 2012; Ord and Lobo 2015; Westblade et al. 2017).

**Natural hosts.** Intermediate hosts are rodents, primarily white-footed mice (*Peromyscusleucopus*), and deer, primarily white-tailed deer (*Odocoileusvirginianus*). Arthropod vector and definitive hosts are ticks in the genus *Ixodes*, primarily *I.scapularis*. Zoonotic in humans as intermediate hosts (Westblade et al. 2017).

**Route of infection.** Introduction of sporozoites via the bite of infected tick, blood transfusion, and solid organ transplants (Ord and Lobo 2015; Westblade et al. 2017).

**Site in human host.** Blood (Ord and Lobo 2015; Westblade et al. 2017).

***Babesiamotasi* Wenyoun, 1926**

*Babesia* sp. Strain KO-1

*Babesiaovis*-like

**Geographic distribution.** Eurasia (Kim et al. 2007; Hong et al. 2019).

**Natural hosts.** Intermediate hosts are sheep. Definitive hosts are ticks in the genus *Haemaphysalis*. Zoonotic in humans as intermediate hosts (Kim et al. 2007; Hong et al. 2019; Wang et al. 2020).

**Route of infection.** Introduction of sporozoites via the bite of infected tick (Kim et al. 2007; Hong et al. 2019; Wang et al. 2020).

**Site in human host.** Blood (Hong et al. 2019).

***Babesiaodocoilei* Emerson & Wright, 1970**

**Geographic distribution.** Eastern North America (Pattullo et al. 2013).

**Natural hosts.** Intermediate hosts are cervids, primarily white-tailed deer (*Odocoileusvirginianus*). Arthropod vector and definitive host is the tick *Ixodesscapularis*; birds may serve as reservoir hosts for larvae or nymphs. Zoonotic in humans as intermediate hosts (Pattullo et al. 2013; Scott et al. 2021).

**Route of infection.** Introduction of sporozoites via the bite of infected tick (Scott et al. 2021).

**Site in human host.** Blood (Scott et al. 2021).

***Babesiavenatorum* Herwaldt et al., 2003**

*Babesia* strain EU1

**Geographic distribution.** Europe (Herwaldt et al. 2003).

**Natural hosts.** Intermediate hosts are red deer (*Cervuselaphus*); roe deer, sheep, dogs, cats, and other mammals may serve as reservoir hosts. Arthropod vectors and definitive hosts are ticks in the genus *Ixodes*, primarily *I.ricinus*. Zoonotic in humans as intermediate hosts (Herwaldt et al. 2003; Gray et al. 2019).

**Route of infection.** Introduction of sporozoites via the bite of infected *Ixodes* tick; possibly also via blood transfusion and solid organ transplants (Herwaldt et al. 2003).

**Site in human host.** Blood (Herwaldt et al. 2003).

***Babesia* sp. Strain MO-1**

*Babesiadivergens*-like

**Geographic distribution.** Central and Pacific Northwest USA (Burgess et al. 2017).

**Natural hosts.** Unknown; presumed intermediate hosts are unknown and presumed arthropod vector and definitive hosts are ticks in the genus *Ixodes*. Zoonotic in humans as intermediate hosts (Burgess et al. 2017; Herc et al. 2018).

**Route of infection.** Introduction of sporozoites via the bite of infected tick; possibly also via blood transfusion and solid organ transplantation (Burgess et al. 2017; Herc et al. 2018).

**Site in human host.** Blood (Burgess et al. 2017; Herc et al. 2018).

**Notes.** This strain was originally described from Missouri, USA. It has since been described from the states of Washington, Kentucky, and Arkansas (Burgess et al. 2017). Genetically it is very similar to the Palearctic *B.divergens* and is often referred to as *Babesiadivergens*-like (Herc et al. 2018).

**Babesia sp. crassa -like**

**Geographic distribution.** Undefined; human cases known from China and Slovenia (Jia et al. 2018; Strasek-Smrdel et al. 2020).

**Natural hosts.** Unknown; presumed intermediate hosts are sheep, goats, presumed arthropod vector and definitive hosts are ticks in the genera *Haemaphysalis* and/or *Ixodes*. Zoonotic in humans as intermediate hosts (Jia et al. 2018; Strasek-Smrdel et al. 2020).

**Route of infection.** Introduction of sporozoites via the bite of infected tick; possibly also via blood transfusion and solid organ transplantation (Jia et al. 2018; Strasek-Smrdel et al. 2020).

**Site in human host.** Blood (Jia et al. 2018; Strasek-Smrdel et al. 2020).

●●●●● **Anthemosomatidae Levine, 1981**

**Genus *Anthemosoma* Landau et al., 1969**

***Anthemosomagarnhami* Landau et al., 1969**

**Geographic distribution.** Sub-Saharan Africa (Chavatte et al. 2018).

**Natural hosts.** Intermediate hosts are rodents, including spiny mice (*Acomys*). Arthropod vector and definitive hosts are unknown but presumed to be ixodid ticks. Zoonotic in humans (Chavatte et al. 2018; Stead et al. 2021).

**Route of infection.** Presumably by introduction of sporozoites via the bite of an infected tick (Stead et al. 2021).

**Site in human host.** Blood (Stead et al. 2021).

●●● **Conoidasida Levine, 1988**

●●●● **Coccidia Leuckart, 1879**

●●●●● **Eimeriorina Léger, 1911**

●●●●●● **Eimeriidae Minchin, 1903**

**Genus *Cyclospora* Schneider, 1881**

***Cyclosporacayetanensis* Ortega et al., 1994**

**Geographic distribution.** Nearly worldwide, hot spots of endemicity include Latin America, the Caribbean, Middle East, and Southeast Asia (Ortega and Sanchez 2010; Almeria et al. 2019).

**Natural host.** Human (Ortega and Sanchez 2010; Almeria et al. 2019).

**Route of infection.** Ingestion of sporulated oocysts in fecally contaminated produce and water (Ortega and Sanchez 2010; Cama and Mathison 2015; Almeria et al. 2019).

**Site in human host.** Small intestine (Ortega and Sanchez 2010; Almeria et al. 2019).

●●●●●● **Sarcocystidae Poche, 1913**

**Genus *Cystoisospora* Frenkel, 1977**

***Cystoisosporabelli* (Wenyon, 1923)**

**Geographic distribution.** Nearly worldwide, more prevalent in tropics and subtropics (Cama and Mathison 2015; Dubey and Almeria 2019).

**Natural host.** Human (Cama and Mathison 2015; Dubey and Almeria 2019).

**Route of infection.** Ingestion of sporulated oocysts in fecally contaminated water, food, fomites (Cama and Mathison 2015; Dubey and Almeria 2019)

**Site in human host.** Small intestine (Cama and Mathison 2015; Dubey and Almeria 2019; Rowan et al. 2020)

**Genus *Sarcocystis* Lankester, 1882**

***Sarcocystisheydorni* Dubey et al., 2015**

**Geographic distribution.** Europe, China (Dubey et al. 2015).

**Natural hosts.** Cattle are intermediate hosts. Humans are the definitive hosts (Dubey 2015; Dubey et al. 2015).

**Route of infection.** Ingestion of cysts (bradyzoites) in undercooked beef (Dubey 2015; Dubey et al. 2015).

**Site in human host.** Small intestine (Dubey 2015; Dubey et al. 2015)

***Sarcocystishominis* (Railiet & Lucet, 1891)**

*Sarcocystisbovihominis* Heydorn et al., 1975

**Geographic distribution.** Nearly worldwide, more prevalent in tropics and subtropics (Dubey 2015).

**Natural hosts.** Intermediate hosts are cattle. Definitive hosts are humans and non-human primates (Fayer 2004; Dubey 2015; Fayer et al. 2015).

**Route of infection.** Ingestion of cysts (bradyzoites) in undercooked beef (Fayer 2004; Dubey 2015; Fayer et al. 2015).

**Site in human host.** Small intestine (Fayer 2004; Dubey 2015; Fayer et al. 2015).

***Sarcocystisnesbitti* Mandour, 1969**

**Geographic distribution.** Southeast Asia (Abubakar et al. 2013; Fayer et al. 2015).

**Natural hosts.** Unknown. Natural intermediate host is unknown, non-human primates can serve as intermediate or reservoir hosts. Definitive hosts are presumed to be snakes or other reptiles. Zoonotic in humans as incidental intermediate hosts (Abubakar et al. 2013).

**Route of infection.** Ingestion of fully-sporulated oocysts in contaminated food, water, fomites (Abubakar et al. 2013; Fayer et al. 2015).

**Site in human host.** Skeletal muscle (Abubakar et al. 2013; Fayer et al. 2015).

***Sarcocystissuihominis* Heydorn, 1977**

**Geographic distribution.** Nearly worldwide, more prevalent in tropics and subtropics (Dubey 2015).

**Natural hosts.** Intermediate hosts are pigs. Definitive hosts are humans and non-human primates (Fayer 2004; Dubey 2015; Fayer et al. 2015).

**Route of infection.** Ingestion of cysts (bradyzoites) in undercooked pork (Fayer 2004; Dubey 2015; Fayer et al. 2015).

**Site in human host.** Small intestine (Fayer 2004; Dubey 2015; Fayer et al. 2015).

**Genus *Toxoplasma* Nicolle & Manceaux, 1909**

***Toxoplasmagondii* (Nicolle & Manceaus, 1908)**

**Geographic distribution.** Worldwide (Robert-Gangneux and Darde 2012).

**Natural hosts.** Wild and domestic felids. Many mammals and birds can serve as reservoir hosts; zoonotic in humans as dead-end hosts (Attias et al. 2020).

**Route of infection.** Ingestion of fully sporulated oocysts in food, water, fomites contaminated with cat feces; ingestion of cysts (bradyzoites) in infected paratenic hosts; blood transfusion or solid organ transplantation; transplacentally from mother to fetus (Montoya and Remington 2008; Robert-Gangneux and Darde 2012; Attias et al. 2020).

**Site in human host.** Disseminated infection, common sites are skeletal muscle, myocardium, brain, and eyes; can be transmitted transplacentally from mother to fetus (Montoya and Remington 2008; Robert-Gangneux and Darde 2012).

●●●● **Gregarinasina Dufour, 1828**

●●●●● **Cryptogregarinorida Cavalier-Smith, 2014, emend. Adl et al., 2019**

●●●●●● **Cryptosporidiidae Leger, 1911**

**Genus *Cryptosporidium* Tyzzer, 1910**

***Cryptosporidiumandersoni* Lindsay et al., 2000**

**Geographic distribution.** Worldwide (Feng et al. 2011).

**Natural hosts.** Cattle, camels, sheep, goats, horses. Zoonotic in humans (Feng et al. 2011; Liu et al. 2015).

**Route of infection.** Ingestion of sporulated oocysts in water, food, fomites contaminated with animal feces (Jiang et al. 2014).

**Site in human host.** Unknown, presumed stomach.

***Cryptosporidiumbovis* Barker & Carbonell, 1974**

**Geographic distribution.** Worldwide (Feng et al. 2007).

**Natural hosts.** Cattle. Zoonotic in humans (Ng et al. 2012; Das et al. 2019).

**Route of infection.** Ingestion of sporulated oocysts in water, food, fomites contaminated with cattle feces (Ng et al. 2012; Das et al. 2019).

**Site in human host.** Duodenum and small intestine.

***Cryptosporidiumcanis* Fayer et al., 2001**

**Geographic distribution.** Worldwide (Fayer et al. 2001).

**Natural hosts.** Dogs. Zoonotic in humans (Fayer et al. 2001; Ryan et al. 2016).

**Route of infection.** Ingestion of sporulated oocysts in water, food, fomites contaminated with dog feces (Fayer et al. 2001; Ryan et al. 2016).

**Site in human host.** Duodenum and small intestine

***Cryptosporidiumcuniculus* Inman & Takeuchi, 1979**

**Geographic distribution.** Undefined, most human cases from Europe and Australia (Koehler et al. 2014; Puleston et al. 2014).

**Natural hosts.** Rabbits, kangaroos. Zoonotic in humans (Koehler et al. 2014; Puleston et al. 2014).

**Route of infection.** Ingestion of sporulated oocysts water, food, fomites contaminated with rabbit/animal feces (Koehler et al. 2014; Puleston et al. 2014; Ryan et al. 2016).

**Site in human host.** Duodenum and small intestine.

***Cryptosporidiumditrichi* Čondlová et al., 2018**

**Geographic distribution.** Europe (Condlova et al. 2018).

**Natural hosts.** Field mice (*Apodemus* spp.). Zoonotic in humans (Condlova et al. 2018; Beser et al. 2020).

**Route of infection.** Ingestion of sporulated oocysts in water, food, fomites contaminated with rodent feces (Beser et al. 2020; Condlova et al. 2018).

**Site in human host.** Duodenum and small intestine

***Cryptosporidiumerinacei* Kváč et al., 2014**

*Cryptosporidium* hedgehog genotype

**Geographic distribution.** Europe (Kváč et al. 2014).

**Natural hosts.** Hedgehogs, horses. Zoonotic in humans (Kváč et al. 2014; Zahedi et al. 2016).

**Route of infection.** Ingestion of sporulated oocysts in water, food, fomites contaminated with animal feces (Kváč et al. 2014; Zahedi et al. 2016).

**Site in human host.** Duodenum and small intestine

***Cryptosporidiumfayeri* Ryan et al., 2008**

**Geographic distribution.** Australia (Ryan et al. 2008; Waldron et al. 2010).

**Natural hosts.** Marsupials. Zoonotic in humans (Waldron et al. 2010).

**Route of infection.** Ingestion of sporulated oocysts in water, food, fomites contaminated with marsupial feces (Waldron et al. 2010).

**Site in human host.** Duodenum and small intestine

***Cryptosporidiumfelis* Iseki, 1979**

**Geographic distribution.** Worldwide (Jiang et al. 2020).

**Natural hosts.** Cats, foxes, horses, cattle. Zoonotic in humans (Caccio et al. 2002; Jiang et al. 2020).

**Route of infection.** Ingestion of sporulated oocysts in fecally contaminated water, food, fomites contaminated with cat/animal feces (Caccio et al. 2002; Jiang et al. 2020).

**Site in human host.** Duodenum and small intestine

***Cryptosporidiumhominis* Morgan-Ryan et al., 2002**

**Geographic distribution.** Worldwide (Cama and Mathison 2015).

**Natural hosts.** Humans, non-human primates (Cama and Mathison 2015; Ryan et al. 2016; Chen et al. 2019).

**Route of infection.** Ingestion of sporulated oocysts in water, food, fomites contaminated with human feces (Cama and Mathison 2015).

**Site in human host.** Duodenum and small intestine, disseminated infections in immunocompromised patients (Bouzid et al. 2013; Cama and Mathison 2015).

***Cryptosporidiummeleagridis* Slavin, 1955**

**Geographic distribution.** Worldwide

**Natural hosts.** Birds. Zoonotic in humans (Akiyoshi et al. 2003).

**Route of infection.** Ingestion of sporulated oocysts in fecally contaminated water, food, fomites contaminated with bird feces (Akiyoshi et al. 2003).

**Site in human host.** Duodenum and small intestine, disseminated infections in immunocompromised patients (Akiyoshi et al. 2003; Kopacz et al. 2019).

***Cryptosporidiummuris* Tyzzer, 1910**

**Geographic distribution.** Worldwide (Chappell et al. 2015).

**Natural hosts.** Rodents, ruminants, horses, non-human primates, carnivores. Zoonotic in humans (Chappell et al. 2015).

**Route of infection.** Ingestion of sporulated oocysts in water, food, fomites contaminated with animal feces (Chappell et al. 2015).

**Site in human host.** Unknown, presumed stomach (Chappell et al. 2015).

***Cryptosporidiumoccultus* Kváč et al., 2018**

*Cryptosporidiumparvum* VF383

**Geographic distribution.** Worldwide (Kvac et al. 2018).

**Natural hosts.** Rodents. Zoonotic in humans (Kvac et al. 2018; Xu et al. 2020).

**Route of infection.** Ingestion of sporulated oocysts in water, food, fomites contaminated with rodent feces (Kvac et al. 2018; Xu et al. 2020).

**Site in human host.** Duodenum and small intestine

***Cryptosporidiumparvum* Tyzzer, 1912**

**Geographic distribution.** Worldwide (Cama and Mathison 2015; Ryan et al. 2016).

**Natural hosts.** Cattle, sheep, goats, and other ruminants, rodents. Zoonotic in humans (Cama and Mathison 2015; Ryan et al. 2016).

**Route of infection.** Ingestion of sporulated oocysts in water, food, fomites contaminated with human and animal feces (Cama and Mathison 2015; Ryan et al. 2016).

**Site in human host.** Duodenum and small intestine, disseminated infections in immunocompromised patients (Cama and Mathison 2015; Ryan et al. 2016).

***Cryptosporidiumryanae* Fayer et al., 2008**

*Cryptosporidium* deer-like genotype

**Geographic distribution.** XX (Fayer et al. 2008).

**Natural hosts.** Cattle. Zoonotic in humans (Chako et al. 2010; Das et al. 2019).

**Route of infection.** Ingestion of sporulated oocysts in water, food, fomites contaminated with cattle feces (Das et al. 2019).

**Site in human host.** Duodenum and small intestine.

***Cryptosporidiumscrofarum* Kváč et al., 2013**

*Cryptosporidium* pig genotype II

**Geographic distribution.** Worldwide (Kváč et al. 2013).

**Natural hosts.** Pigs. Zoonotic in humans (Ryan et al. 2016).

**Route of infection.** Ingestion of sporulated oocysts in water, food, fomites contaminated with pig feces (Ryan et al. 2016).

**Site in human host.** Duodenum and small intestine.

***Cryptosporidiumsuis* Ryan et al., 2004**

**Geographic distribution.** Worldwide (Ryan et al. 2004).

**Natural hosts.** Pigs. Zoonotic in humans (Xiao et al. 2002).

**Route of infection.** Ingestion of sporulated oocysts in water, food, fomites contaminated with pig feces (Xiao et al. 2002; Nemejc et al. 2013)

**Site in human host.** Duodenum and small intestine

***Cryptosporidiumtyzzeri* Ren et al., 2012**

*Cryptosporidium* mouse genotype I

**Geographic distribution.** Presumed worldwide (Raskova et al. 2013).

**Natural hosts.** Rodents, horses may serve as reservoir hosts. Zoonotic in humans (Raskova et al. 2013; Wagnerová et al. 2015).

**Route of infection.** Ingestion of sporulated oocysts in water, food, fomites contaminated with rodent feces (Raskova et al. 2013).

**Site in human host.** Duodenum and small intestine

***Cryptosporidiumubiquitum* Fayer et al., 2010**

*Cryptosporidium* cervine genotype

**Geographic distribution.** Worldwide (Li et al. 2014).

**Natural hosts.** Many mammal species, including ruminants, rodents, and carnivores; presumed zoonotic in humans (Li et al. 2014).

**Route of infection.** Ingestion of sporulated oocysts in water, food, fomites contaminated with animal feces (Li et al. 2014).

**Site in human host.** Duodenum and small intestine

***Cryptosporidiumviatorum* Elwin et al., 2012**

**Geographic distribution.** Worldwide (Koehler et al. 2018).

**Natural hosts.** Rodents. Zoonotic in humans (Koehler et al. 2018).

**Route of infection.** Ingestion of sporulated oocysts in water, food, fomites contaminated with rodent and human feces (Koehler et al. 2018).

**Site in human host.** Duodenum and small intestine.

##### ●● Ciliophora Doflein, 1901

●●● **Postodesmatophora Gerassimova & Seravin, 1976**

●●●● **SAL (Spirotrichea-Lamellicorticata-Armophorea) Gentekaki et al., 2014**

●●●●● **Litostomatea Small & Lynn, 1981**

●●●●●● **Trichostomatia Bütschli, 1889**

●●●●●●● **Balantidiidae Reichenow in Doflein & Reichenow, 1929**

**Genus *Balantioides* Alexeieff, 1931**

*Neobalantidium* Pomajbíková et al., 2013

***Balantioidescoli* (Malmsten, 1857)**

**Geographic distribution.** Worldwide; high areas of endemicity include the Altiplano region of Bolivia, Philippines, New Guinea, Middle East (Schuster and Ramirez-Avila 2008).

**Natural hosts.** Pigs, rodents. Zoonotic in humans (Schuster and Ramirez-Avila 2008).

**Route of infection.** Ingestion of mature cysts in fecally contaminated water, food, fomites (Schuster and Ramirez-Avila 2008).

**Site in human host.** Large intestine (Schuster and Ramirez-Avila 2008).

### Excavates Cavalier-Smith, 2002, emend. Simpson, 2003.

#### ● Metamonada Grassé, 1952

##### ●● Fornicata Simpson, 2003

●●● **Diplomonadida Wenyon, 1926**

●●●● **Hexamitidae Kent, 1880**

**Genus *Enteromonas* da Fonseca, 1915**

***Enteromonashominis* da Fonseca, 1915**

*Tricercomonasintestinalis* Wenyon et O’Connor, 1917

**Geographic distribution.** Worldwide (Ash and Orihel 2007).

**Natural host.** Humans (Ash and Orihel 2007).

**Route of infection.** Ingestion of mature cysts in fecally contaminated water, food, fomites (Spriegel et al. 1989; Ash and Orihel 2007).

**Site in human host.** Large intestine (Ash and Orihel 2007).

**Genus *Giardia* Künstler, 1882**

***Giardiaduodenalis* Stiles, 1902**

*Cercomonasintestinalis* Lambl, 1859

*Giardialamblia* Kofoid & Christiansen, 1915

*Giardiaenterica* Kofoid & Christiansen, 1920

**Geographic distribution.** Worldwide (Adam 2001; Cama and Mathison 2015).

**Natural hosts.** Two genetic assemblages implicated in human disease: A and B. Both have been found in humans and numerous non-human mammals and birds (Ryan and Caccio 2013; Heyworth 2016).

**Route of infection.** Ingestion of mature cysts in fecally contaminated water, food, fomites; sexual transmission via oral-anal route (Adam 2001; Escobedo et al. 2014; Cama and Mathison 2015).

**Site in human host.** Duodenum, small intestine (Adam 2001; Cama and Mathison 2015).

●●● **Retortamonadida Grassé, 1952**

●●●● **Retortamonadidae Wenrich, 1932**

**Genus *Chilomastix* Alexeieff, 1912**

***Chilomastixmesnili* (Wenyon, 1910)**

**Geographic distribution.** Worldwide (Ash and Orihel 2007).

**Natural hosts.** Humans, non-human primates (Ash and Orihel 2007).

**Route of infection.** Ingestion of mature cysts in fecally contaminated water, food, fomites (Morimoto et al. 1996; Ash and Orihel 2007).

**Site in human host.** Large intestine (Ash and Orihel 2007).

**Genus *Retortamonas* Grassi, 1879**

***Retortamonasintestinalis* (Wenyon et O’Connoer, 1917)**

**Geographic distribution.** Worldwide (Ash and Orihel 2007).

**Natural host.** Humans (Ash and Orihel 2007).

**Route of infection.** Ingestion of mature cysts in fecally contaminated water, food, fomites (Mendez et al. 2002; Ash and Orihel 2007).

**Site in human host.** Large intestine (Ash and Orihel 2007).

##### ●● Parabasalia Honigberg, 1973

●●● **Trichonomadida Kirby, 1947**

●●●● **Monocercomonadidae Kirby, 1944**

**Genus *Dientamoeba* Jepps & Dobell, 1918**

***Dientamoebafragilis* Jepps & Dobell, 1918**

**Geographic distribution.** Worldwide (Stark et al. 2016).

**Natural hosts.** Humans, non-human primates, pigs (Stark et al. 2016).

**Route of infection.** Ingestion of trophozoites in fecally contaminated water, food, fomites. A putative cyst stage has been described, but it is not yet widely accepted among protozoologists (Munasinghe et al. 2013; Stark et al. 2014, 2016).

**Site in human host.** Large intestine (Stark et al. 2016).

●●●● **Trichomonadidae Chalmers & Pekkola, 1918, emend. Hampl et al., 2004**

**Genus *Pentatrichomonas* Mensil, 1914**

***Pentatrichomonashominis* (Davaine, 1860)**

**Geographic distribution.** Worldwide (Ash and Orihel 2007).

**Natural hosts.** Humans, non-human primates, dogs, cats, foxes, rabbits, cattle, sheep, goats, deer (Li et al. 2016, 2017, 2018).

**Route of infection.** Ingestion of trophozoites in fecally contaminated fomites (Ash and Orihel 2007).

**Site in human host.** Large intestine (Ash and Orihel 2007).

**Genus *Trichomonas* Donné, 1837**

***Trichomonastenax* (Müller, 1773)**

**Geographic distribution.** Worldwide (Dubar et al. 2020).

**Natural host.** Humans (Dubar et al. 2020).

**Route of infection.** Direct person-to-person contact (Dubar et al. 2020).

**Site in human host.** Oral cavity; ectopic colonization of pleural cavity (Bellanger et al. 2008; Marty et al. 2017; Dubar et al. 2020).

***Trichomonasvaginalis* (Donné, 1836)**

**Geographic distribution.** Worldwide (Schwebke and Burgess 2004; Kissinger 2015).

**Natural host.** Humans (Schwebke and Burgess 2004; Kissinger 2015).

**Route of infection.** Sexual contact (Schwebke and Burgess 2004; Kissinger 2015).

**Site in human host.** Urogenital tract; rare ectopic colonization of CSF (Schwebke and Burgess 2004; Hamilton et al. 2018).

**Genus *Tritrichomonas* Kofoid, 1920**

***Tritrichomonasfoetus* Reidmüller, 1928**

*Trichomonassuis* Gruby & Delafond, 1843

**Geographic distribution.** Worldwide (Suzuki et al. 2016).

**Natural host.** Cattle, pigs, cats. Zoonotic and opportunistic in immunocompromised humans (Maritz et al. 2014; Yao and Köster 2015; Suzuki et al. 2016).

**Route of infection.** Unknown.

**Site in human host.** Respiratory tract, CNS, urogenital tract (Duboucher et al. 2006; Zalonis et al. 2011; Suzuki et al. 2016).

**Genus *Tetratrichomonas* Parisi, 1910**

**Tetratrichomonas sp. cf.gallinarum**

**Geographic distribution.** Unknown, presumed worldwide

**Natural host.** Unknown, presumed birds. Zoonotic in humans (Mantini et al. 2009; Maritz et al. 2014).

**Route of infection.** Unknown

**Site in human host.** Respiratory tract, possibly associated with bacterial infection (Mantini et al. 2009; Maritz et al. 2014).

**Notes.** This species has yet to be formally described. It was first isolated from pleural fluid in a patient with empyema. Bacterial cultures of the pleural fluid grew two *Streptococcus* species and *Prevotella* (Mantini et al. 2009). The protozoan may have been associated with bacteria and not an agent of human disease.

***Tetratrichomonas* sp. (undescribed species)**

*Tetratrichomonasempyemagena* Lopez-Escamilla et al., 2013

**Geographic distribution.** Unknown, presumed worldwide

**Natural hosts.** Unknown

**Route of infection.** Unknown

**Site in human host.** Respiratory tract (Lopez-Escamilla et al. 2013)

**Notes.** This species was reported from two patients from Mexico(Lopez-Escamilla et al. 2013). The species was originally named *T.empyemagena*, but because it was not described under the criteria of the ICZN, it name should be considered *nomen nudum*. This isolate might be conspecific with that described by Mantini et al. (2009).

●●●● **Simplicimonadidae Čepička et al., 2010**

**Genus *Simplicimonas* Čepička et al., 2010**

***Simplicimonassimilis* Čepička et al., 2010**

**Geographic distribution.** Undefined, possibly worldwide (Dimasuay et al. 2013).

**Natural hosts.** Several vertebrates; previously recorded from lizards, cattle, and chickens (Dimasuay et al. 2013).

**Route of infection.** Unknown.

**Site in human host.** Intestinal tract (Greigert et al. 2018).

**Notes.***Simplicimonassimilis* was detected by molecular methods in four human patients in Madagascar (Greigert et al. 2018). The authors acknowledged that the parasite was not detected by microscopy and the finding may be based on spurious passage after consuming insects or another host, and further studies are required to determine if humans can serve as a reliable host (Greigert et al. 2018).

#### ● Discoba Simpson in Hampl et al., 2009

##### ●● Heterolobosea Page & Blanton, 1985

●●● **Tetramitia Cavalier-Smith, 1993**

●●●● **Vahlkampfiidae Jollos, 1917**

**Genus *Naegleria* Alexeieff, 1912**

***Naegleriafowleri* Carter, 1970**

**Geographic distribution.** Worldwide (Gharpure et al. 2020).

**Natural host.** None (environmental); humans are incidental hosts (Visvesvara 2013).

**Route of infection.** Exposure to trophozoites and cysts in contaminated freshwater via the nasal cavity; improper use of nasal irrigation devices (Yoder et al. 2012; Visvesvara 2013).

**Site in human host.** CNS (Visvesvara 2013; Yoder et al. 2012; Mathison and Pritt 2018).

**Genus *Paravahlkampfia* Brown et de Jonckheere, 1999**

***Paravahlkampfiafrancinae* Visvesvara et al., 2009**

**Geographic distribution.** Unknown (single case from Ohio, USA) (Visvesvara et al. 2009).

**Natural host.** None (environmental); humans are incidental hosts (Visvesvara et al. 2009).

**Route of infection.** Unknown; presumed environmental exposure.

**Site in human host.** CNS (Visvesvara et al. 2009).

**Genus *Tetramitus* Perty, 1852**

***Tetramitusentericus* (Page, 1974)**

**Geographic distribution.** Worldwide (Brown and De Jonckheere 1999).

**Natural host.** None (environmental); humans are incidental hosts (Walochnik et al. 2000).

**Route of infection.** Presumed environmental exposure.

**Site in human host.** Eye (Walochnik et al. 2000).

***Tetramitusjugosus* (Page, 1967)**

**Geographic distribution.** Worldwide (Enzien et al. 1989).

**Natural host.** None (environmental); humans are incidental hosts (Dua et al. 1998).

**Route of infection.** Presumed environmental exposure (Dua et al. 1998).

**Site in human host.** Eye (Dua et al. 1998).

**Genus *Vahlkampfia* Chatton et Lalung-Bonnaire, 1912**

**Geographic distribution.** Worldwide (Walochnik et al. 2000).

**Natural host.** None (environmental); humans are incidental hosts (Walochnik et al. 2000).

**Route of infection.** Unknown; presumed environmental exposure.

**Site in human host.** Eye (Walochnik et al. 2000).

**Notes.** Most reports of *Vahlampfia* from human clinical specimens are not characterized at the species level and may represent species currently assigned to other genera such as *Tetramitus*, *Allovahlkampfia*, and *Paravahlkampfia*.

●●●● **Acrasidae Poche, 1913**

**Genus *Allovahlkampfia* Walochnik & Mulec, 2009**

***Allovahlkampfiaspelaea* Walochnik & Mulec, 2009**

**Geographic distribution.** Not fully known; described from caves in Slovenia with single human case from Egypt (Walochnik 2009; Tolba et al. 2016).

**Natural host.** None (environmental); humans are incidental hosts (Tolba et al. 2016).

**Route of infection.** Unknown; presumed environmental exposure.

**Site in human host.** Eye (Tolba et al. 2016).

##### ●● Euglenozoa Cavalier-Smith, 1981, emend. Simpson, 1997

●●● **Kinetoplastea Honigberg, 1963**

●●●● **Metakinetoplastina Vickerman in Moreira et al., 2004**

●●●●● **Trypanosomatida Kent 1880, emend. Vickerman in Moreira et al. 2004**

●●●●●● **Trypanosomatidae Doflein, 1901**

**Genus *Crithidia* Léger, 1902**

**Geographic distribution.** Worldwide (McGhee and Cosgrove 1980).

**Natural hosts.** Arthropods, mainly insects. Zoonotic in humans (McGhee and Cosgrove 1980).

**Route of infection.** Introduction of promastigotes via the bite of infected arthropod vector (Ghobakhloo et al. 2019; Maruyama et al. 2019).

**Site in human host.** Skin, bone marrow (Ghobakhloo et al. 2019; Maruyama et al. 2019).

**Notes.***Crithidia* isolates from human clinical specimens have yet to be characterized at the species level. To date, cases are known from Brazil (Maruyama et al. 2019) and Iran (Ghobakhloo et al. 2019). The vectors in the human cases are also unknown.

**Genus *Endotrypanum* Mesnil & Brimont, 1908**

***Endotrypanumcolombiensis* (Kreuter et al., 1991)**

**Geographic distribution.** Colombia (Kreutzer et al. 1991).

**Natural hosts.** Mammalian hosts are sloths; zoonotic in humans. Arthropod vectors are sand flies in the genus *Lutzomyia* (Kreutzer et al. 1991).

**Route of infection.** Introduction of promastigotes via the bite of infected *Lutzomyia* sand fly (Kreutzer et al. 1991).

**Site in human host.** Skin (Kreutzer et al. 1991)

***Endotrypanumequatoriensis* (Grimaldi-Junior et al., 1992)**

**Geographic distribution.** Colombia, Ecuador (Grimaldi Junior et al. 1992).

**Natural hosts.** Mammalian hosts are sloths and squirrels; zoonotic in humans. Arthropod vectors are sand flies in the genus *Lutzomyia* (Grimaldi Junior et al. 1992).

**Route of infection.** Introduction of promastigotes via the bite of infected *Lutzomyia* sand fly (Grimaldi Junior et al. 1992).

**Site in human host.** Skin (Grimaldi Junior et al. 1992).

**Genus *Leishmania* Ross, 1903**

*Euleishmania* Cupolillo et al., 2000

**SubgenusLeishmania Ross, 1903**

***Leishmaniai* (*L* .) *aethiopica*Bray et al., 1973**

**Geographic distribution.** Africa (Ethiopia, Kenya, Uganda), Yemen (van Henten et al. 2018).

**Natural hosts.** Mammalian hosts include humans, rock hyraxes, and other wild mammals. Arthropod vectors are sand flies in the genus *Phlebotomus*, mainly *P.longipes* and *P.pedifer* (Bray et al. 1973).

**Route of infection.** Introduction of promastigotes via the bite of infected *Phlebotomus* sand fly (van Henten et al. 2018).

**Site in human host.** Skin (van Henten et al. 2018).

**Leishmania (L.) amazonensis Lainson & Shaw, 1972**

*Leishmaniagarnhami* Scorza et al., 1978

**Geographic distribution.** Amazonian South America (Brazil, Venezuela, Bolivia) (Torres-Guerrero et al. 2017).

**Natural hosts.** Mammals and birds, including humans, rodents, dogs, chickens. Arthropod vectors are sand flies in the genus *Lutzomyia* (Torres-Guerrero et al. 2017; Valdivia et al. 2017).

**Route of infection.** Introduction of promastigotes via the bite of infected *Lutzomyia* sand fly (Torres-Guerrero et al. 2017; Valdivia et al. 2017).

**Site in human host.** Skin, mucosal membranes of nose, throat, and mouth (Torres-Guerrero et al. 2017).

**Leishmania (L.) donovani (Laveran & Mesnil, 1903)**

*Leishmaniaarchibaldi* Castellani & Chalmers, 1919

**Geographic distribution.** Indian subcontinent, China, Central Africa; hot spots of endemicity include India, Bangladesh, Nepal, Sudan (Lukes et al. 2007; Ready 2014).

**Natural hosts.** Mammalian hosts include humans, dogs, foxes, marsupials, and rodents. Arthropod vectors are sand flies in the genera *Phlebotomus* (Old World) and *Lutzomyia* (New World) (Lukes et al. 2007; Ready 2014).

**Route of infection.** Introduction of promastigotes via the bite of infected sand fly (Torres-Guerrero et al. 2017).

**Site in human host.** Skin, spleen, liver, small intestine, lymph nodes (Torres-Guerrero et al. 2017).

**Leishmania (L.) infantum Nicolle, 1908**

*Leishmaniachagasi* da Cunha & Chagas, 1937

**Geographic distribution.** Mediterranean Europe and Africa, southeastern Europe, Middle East, Central Asia, Central and South America (Mexico, Venezuela, Brazil, Bolivia) (Pratlong et al. 2013; Steverding 2017).

**Natural hosts.** Mammalian hosts include dogs, cats, foxes, lagomorphs, rodents, marsupials, and others; zoonotic in humans. Arthropod vectors are sand flies in the genera *Phlebotomus* (Old World) and *Lutzomyia* (New World) (Millan et al. 2014).

**Route of infection.** Introduction of promastigotes via the bite of infected sand fly (Torres-Guerrero et al. 2017).

**Site in human host.** Skin, spleen, liver, small intestine, lymph nodes (Torres-Guerrero et al. 2017).

**Leishmania (L.) major Yakimoff & Schokhor, 1914**

**Geographic distribution.** Northern and Central Africa, Middle East, India, China (Guerrant et al. 2006; Steverding 2017).

**Natural hosts.** Humans; gerbils and birds may serve as reservoir hosts; dogs can become infected but are not adequate reservoir hosts for human disease. Arthropod vectors are sand flies in the genus *Phlebotomus* (Al-Bajalan et al. 2018).

**Route of infection.** Introduction of promastigotes via the bite of infected *Phlebotomus* sand fly (Torres-Guerrero et al. 2017).

**Site in human host.** Skin (Torres-Guerrero et al. 2017).

***Leishmania* (*L.*) *mexicana* Biagi, 1953, emend. Garnham, 1962**

*Leishmaniapifanoi* Medina & Romero, 1962

**Geographic distribution.** Texas, Mexico, Belize, Guatemala, Brazil, Ecuador, Peru, Venezuela (Steverding 2017).

**Natural hosts.** Mammalian hosts include humans, dogs, rodents, and bats. Arthropod vectors are sand flies in the genus *Lutzomyia* (Berzunza-Cruz et al. 2015).

**Route of infection.** Introduction of promastigotes via the bite of infected sand fly (Steverding 2017; Torres-Guerrero et al. 2017).

**Site in human host.** Skin (Steverding 2017; Torres-Guerrero et al. 2017).

**Leishmania (L.) tropica Wright, 1903**

**Geographic distribution.** North and Central Africa, Middle East, Central Asia, India (Steverding 2017).

**Natural hosts.** Mammalian hosts include humans, rock hyraxes, gundi, and dogs (although dogs are not an adequate reservoir host for human disease). Arthropod vectors are sand flies in the genus *Phlebotomus* (Ajaoud et al. 2013).

**Route of infection.** Introduction of promastigotes via the bite of infected sand fly (Steverding 2017; Torres-Guerrero et al. 2017).

**Site in human host.** Skin (Steverding 2017; Torres-Guerrero et al. 2017).

**Leishmania (L.) venezuelensis (Bonfante-Garrido, 1980)**

**Geographic distribution.** Venezuela, northern South America (Steverding 2017).

**Natural hosts.** Mammalian hosts include humans, cats. Arthropod vectors are sand flies in the genus *Lutzomyia* (Rivas et al. 2018).

**Route of infection.** Introduction of promastigotes via the bite of infected *Lutzomyia* sand fly (Steverding 2017; Torres-Guerrero et al. 2017).

**Site in human host.** Skin (Steverding 2017; Torres-Guerrero et al. 2017).

***Leishmania* (*L* .) *waltoni*Shaw et al., 2015**

**Geographic distribution.** Dominican Republic (Shaw et al. 2015).

**Natural hosts.** Mammalian hosts unknown, rats may serve as reservoir hosts; presumed zoonotic in humans. Arthropod vectors are sand flies in the genus *Lutzomyia* (Shaw et al. 2015).

**Route of infection.** Introduction of promastigotes via the bite of infected *Lutzomyia* sand fly (Shaw et al. 2015).

**Site in human host.** Skin (Shaw et al. 2015).

**SubgenusMundinia Shaw et al. in Espinosa et al., 2016**

***Leishmania* (*M.*) *martiniquensis*Desbois et al., 2014**

**Geographic distribution.** Martinique, Thailand (Desbois et al. 2014; Pothirat et al. 2014).

**Natural hosts.** Mammalian hosts are humans. Rats may serve as reservoir hosts. Arthropod vectors are sand flies, possibly in the genera *Sergentomyia* (Old World) and *Lutzomyia* (New World) (Leelayoova et al. 2017).

**Route of infection.** Introduction of promastigotes via the bite of infected sand fly

**Site in human host.** Skin, mucosal membranes of nose, throat, and mouth (Desbois et al. 2014; Liautaud et al. 2015).

**Leishmania (M.) orientalis Jariyapan et al., 2018**

*Leishmaniasiamensis* Sukmee et al., 2008

**Geographic distribution.** Thailand (Sukmee et al. 2008; Jariyapan et al. 2018).

**Natural hosts.** Mammalian hosts are humans. Arthropod vectors are sand flies. (Sukmee et al. 2008; Jariyapan et al. 2018).

**Route of infection.** Introduction of promastigotes via the bite of infected sand fly (genus unknown).

**Site in human host.** Skin (Sukmee et al. 2008; Jariyapan et al. 2018).

**SubgenusSauroleishmania Ranque, 1973**

***Leishmania* (*S.*) *tarentolae* Weynon, 1921**

**Geographic distribution.** North Africa, southern Europe, Middle East (Klatt et al. 2019).

**Natural hosts.** Definitive hosts are lizards. Arthropod vectors are sand flies in the genus *Sergentomyia*. Zoonotic in humans (Klatt et al. 2019).

**Route of infection.** Introduction of promastigotes in bite of infected *Sergentomyia* sand fly (Klatt et al. 2019; Pombi et al. 2020).

**Site in human host.** Blood (Pombi et al. 2020).

**Notes.** The single human case was diagnosed by molecular detection (only) in blood (Pombi et al. 2020).

**SubgenusViannia Lainson & Shaw, 1987**

**Leishmania (V.) braziliensis Vianna, 1911**

**Geographic distribution.** Amazonian South America (Brazil, Bolivia, Venezuela, Peru), Guatemala (Castro et al. 2007; Steverding 2017).

**Natural hosts.** Mammalian hosts include humans, dogs, rodents, and marsupials. Arthropod vectors are sand flies in the genus *Lutzomyia* (Castro et al. 2007).

**Route of infection.** Introduction of promastigotes via the bite of infected *Lutzomyia* sand fly (Steverding 2017; Torres-Guerrero et al. 2017).

**Site in human host.** Skin, mucosal membranes of nose, throat, and mouth (Steverding 2017; Torres-Guerrero et al. 2017).

**Leishmania (V.) guyanensis Floch, 1954**

**Geographic distribution.** Northern South America (French Guiana, Suriname, Brazil, Bolivia) (Guerra et al. 2011; Steverding 2017).

**Natural hosts.** Mammalian hosts include sloths, anteaters, rodents, marsupials, and humans. Arthropod vectors are sand flies in the genus *Lutzomyia* (Guerra et al. 2011).

**Route of infection.** Introduction of promastigotes via the bite of infected *Lutzomyia* sand fly (Steverding 2017; Torres-Guerrero et al. 2017).

**Site in human host.** Skin, mucosal membranes of nose, throat, and mouth (Steverding 2017; Torres-Guerrero et al. 2017).

***Leishmania* (*V.*) *lainsoni* Silveira et al., 1987**

**Geographic distribution.** Brazil, Bolivia, Ecuador, Peru, Suriname, French Guiana (Kato et al. 2016; Mans et al. 2017; Steverding 2017).

**Natural hosts.** Mammalian host is lowland paca; zoonotic in humans. Arthropod vectors are sand flies in the genus *Lutzomyia* (Mans et al. 2017).

**Route of infection.** Introduction of promastigotes via the bite of infected *Lutzomyia* sand fly (Steverding 2017; Torres-Guerrero et al. 2017).

**Site in human host.** Skin (Steverding 2017; Torres-Guerrero et al. 2017).

***Leishmania* (*V* .) *lindenbergi* Silveira et al., 2002**

**Geographic distribution.** Brazil (Cantanhêde et al. 2019).

**Natural hosts.** Mammalian hosts unknown; presumed zoonotic in humans. Arthropod vectors are sand flies in the genus *Lutzomyia* (Cantanhêde et al. 2019).

**Route of infection.** Introduction of promastigotes via the bite of infected *Lutzomyia* sand fly (Steverding 2017; Torres-Guerrero et al. 2017).

**Site in human host.** Skin (Steverding 2017; Torres-Guerrero et al. 2017).

***Leishmania* (*V.*) *naiffi* Lainson & Shaw, 1989**

**Geographic distribution.** Brazil, Bolivia, Peru (Lainson and Shaw 1989; Fagundes-Silva et al. 2015).

**Natural hosts.** Mammalian hosts are armadillos; zoonotic in humans. Arthropod vectors are sand flies in the genus *Lutzomyia* (Lainson and Shaw 1989; Fagundes-Silva et al. 2015).

**Route of infection.** Introduction of promastigotes via the bite of infected *Lutzomyia* sand fly (Steverding 2017; Torres-Guerrero et al. 2017).

**Site in human host.** Skin (Steverding 2017; Torres-Guerrero et al. 2017).

***Leishmania* (*V.*) *panamensis* Lainson & Shaw, 1972**

**Geographic distribution.** Central and South America (Steverding 2017; Torres-Guerrero et al. 2017).

**Natural hosts.** Mammalian hosts include sloths, procyonids, rodents, humans, and non-human primates. Arthropod vectors are sand flies in the genus *Lutzomyia* (Steverding 2017; Torres-Guerrero et al. 2017).

**Route of infection.** Introduction of promastigotes via the bite of infected *Lutzomyia* sand fly (Steverding 2017; Torres-Guerrero et al. 2017).

**Site in human host.** Skin, mucosal membranes of nose, throat, and mouth (Steverding 2017; Torres-Guerrero et al. 2017).

***Leishmania* (*V.*) *peruviana* Velez, 1913**

**Geographic distribution.** Peru, Bolivia (Steverding 2017; Torres-Guerrero et al. 2017).

**Natural hosts.** Mammalian hosts include humans, rodents, marsupials, and domestic dogs. Arthropod vectors are sand flies in the genera *Lutzomyia* and possibly *Pintomyia* (Kato et al. 2021).

**Route of infection.** Introduction of promastigotes via the bite of infected sand fly (Steverding 2017; Torres-Guerrero et al. 2017).

**Site in human host.** Skin, mucosal membranes of nose, throat, and mouth (Steverding 2017; Torres-Guerrero et al. 2017).

**Leishmania (V.) shawi Lainson et al., 1989**

**Geographic distribution.** Brazil (Lainson et al. 1989)

**Natural hosts.** Mammalian hosts include non-human primates, sloths, coati; zoonotic in humans. Arthropod vectors are sand flies in the genus *Lutzomyia* (Lainson et al. 1989).

**Route of infection.** Introduction of promastigotes via the bite of infected *Lutzomyia* sand fly (Steverding 2017; Torres-Guerrero et al. 2017).

**Site in human host.** Skin (Steverding 2017; Torres-Guerrero et al. 2017).

**Genus *Leptomonas* Kent, 1880**

***Leptomonasseymouri* (Wallace, 1877)**

**Geographic distribution.** Worldwide (Kraeva et al. 2015).

**Natural hosts.** Insects. Zoonotic in humans (Ghosh et al. 2012; Kraeva et al. 2015; Thakur et al. 2020).

**Route of infection.** Presumed introduction of promastigotes via the bite of infected *Phlebotomus* sand flies (Ghosh et al. 2012; Kraeva et al. 2015; Thakur et al. 2020).

**Site in human host.** Skin, spleen, bone marrow (Ghosh et al. 2012; Kraeva et al. 2015; Thakur et al. 2020)

**Notes.** Human cases of *L.seymouri* to date have been found in Southeast Asia associated with co-infection with *Leishmaniadonovani*, suggesting incidental human infection after the bite of an infected sand fly vector.

**Genus *Trypanosoma* Gruby, 1843**

**SubgenusHerpetosoma Doflein, 1901**

***Trypanosoma* (*H.*) *lewisi* Laveran & Mesnil, 1901**

**Geographic distribution.** Cosmopolitan (Desquesnes et al. 2002).

**Natural hosts.** Mammalian hosts are rats (*Rattus* spp.); zoonotic in humans. Arthropod vectors are rat fleas *Nosophyllusfasciatus* and *Xenopsyllacheopis* (Desquesnes et al. 2002).

**Route of infection.** Ingestion of infected fleas or their feces (Desquesnes et al. 2002).

**Site in human host.** Blood (Verma et al. 2011; de Sousa 2014).

***Trypanosoma* (*H.*) *rangeli* Tejera, 1920**

*Trypanosomasaimirii* Rodhain, 1941

**Geographic distribution.** South America (Guhl and Vallejo 2003).

**Natural hosts.** Mammalian hosts include coati, sloths, tamandua, and opossums; zoonotic in humans. Arthropod vectors are triatomine bugs, most-notably members of the genus *Rhodnius* (Miles et al. 1983; Guhl and Vallejo 2003; Ferreira L de et al. 2015).

**Route of infection.** Introduction of trypomastigotes via the bite of infected triatomine bug (Guhl and Vallejo 2003; Ferreira L de et al. 2015).

**Site in human host.** Blood (Guhl and Vallejo 2003; Ferreira L de et al. 2015).

**SubgenusSchizotrypanum Chagas, 1909**

**Trypanosoma (S.) cruzi Chagas, 1909**

**Geographic distribution.** Southern United States, Central and South America (Bern et al. 2011; Kirchhoff 2011; Conners et al. 2016).

**Natural hosts.** Mammalian hosts include humans and many domestic and wild mammals. Arthropod vectors are triatomine bugs, most-notably members of the genera *Triatoma*, *Panstrongylus*, and *Rhodnius* (Kirchhoff 2011).

**Route of infection.** Introduction of metacyclic trypomastigotes in the feces of infected triatomine bug when rubbed into wounds or mucus membranes; ingestion of food contaminated with bugs or their feces; transplacentally from mother to fetus; via blood transfusion and organ transplantation (Coura 2015; Conners et al. 2016; Lidani et al. 2019).

**Site in human host.** Blood (acute infection, reactivation); heart muscle, esophagus, large intestine, peripheral nervous system, CNS, other organs (chronic infection) (Coura 2015; Conners et al. 2016; Lidani et al. 2019).

**SubgenusTrypanozoon Lühe, 1906**

***Trypanosoma* (*T.*) *brucei gambiense* (Dutton, 1902)**

**Geographic distribution.** West-central Africa; hot spots of endemicity include Angola, Democratic Republic of Congo, South Sudan, Central African Republic, Uganda (Brun et al. 2010; Franco et al. 2014).

**Natural hosts.** Mammalian hosts include humans, non-human primates, and ungulates. Arthropod vectors are tsetse flies in the genus *Glossina* (Brun et al. 2010; Franco et al. 2014).

**Route of infection.** Introduction of trypomastigotes via the bite of infected *Glossina* tsetse fly (Brun et al. 2010).

**Site in human host.** Blood, lymphatics, CNS (Brun et al. 2010).

***Trypanosoma* (*T.*) *brucei rhodesiense* (Stephen & Fantham, 1910)**

**Geographic distribution.** East and southeast Africa; hot spots of endemicity include Malawi, Zambia, Tanzania, Botswana, Kenya (Brun et al. 2010; Franco et al. 2014).

**Natural hosts.** Mammalian hosts include livestock and wild ungulates; zoonotic in humans. Arthropod vectors are tsetse flies in the genus *Glossina* (Brun et al. 2010; Franco et al. 2014).

**Route of infection.** Introduction of trypomastigotes via the bite of infected *Glossina* tsetse fly (Brun et al. 2010).

**Site in human host.** Blood, lymphatics, CNS (Brun et al. 2010).

***Trypanosoma* (*T.*) *evansi* Balbiani, 1888**

**Geographic distribution.** Equatorial regions of Africa, Asia, Latin America (Van Vinh Chau et al. 2016).

**Natural hosts.** Mammalian hosts include horses, camels, bovids; zoonotic in humans. Arthropod vectors are biting flies in the genera *Stomoxys* and *Tabanus* (Van Vinh Chau et al. 2016).

**Route of infection.** Introduction of trypomastigotes via the bite of infected flies; possibly direct contact with blood/wounds of infected animals (Joshi et al. 2005; Powar et al. 2006; Van Vinh Chau et al. 2016).

**Site in human host.** Blood (Joshi et al. 2005; Powar et al. 2006; Van Vinh Chau et al. 2016).

### Opisthokonta Cavalier-Smith, 1987, emend. Adl et al., 2005

#### ● Holozoa Lang et al., 2002

##### ●● Metazoa Haeckel, 1874, emend. Adl et al. 2005

●●● **Protostomia Grobben, 1908**

●●●● **Spiralia Edgecombe et al., 2011**

●●●●● **Acanthocephala Koelreuther, 1771**

●●●●●● **Oligacanthorhynchida Petrotschenko, 1956**

●●●●●●● **Oligacanthorhynchidae Southwell & Macfie, 1925**

**Genus *Macracanthorhynchus* Travassos, 1917**

***Macracanthorhynchushirudinaceus* (Pallas, 1781)**

*Taeniahaeruca* Pallas, 1776

*Echinorhynchusgigas* Bloch, 1782

**Geographic distribution.** Worldwide (Mathison et al. 2016).

**Natural hosts.** Intermediate hosts are insects, primarily scarabaeoid beetles. Definitive hosts are pigs; dogs and other mammals can serve as reservoir hosts. Zoonotic in humans (Mathison et al. 2016).

**Route of infection.** Ingestion of cystacanths in infected insect intermediate host (Mathison et al. 2016).

**Site in human host.** Small intestine (Mathison et al. 2016).

***Macracanthorhynchusingens* (von Linstow, 1879)**

**Geographic distribution.** Eastern North America (Elkins and Nickol 1983; Richardson et al. 2017).

**Natural hosts.** Intermediate hosts are millipedes. Definitive hosts are raccoons (*Procyonlotor*) and American black bear (*Ursusamericanus*); many other mammals, especially carnivores, can serve as definitive hosts. Snakes and other animals may serve as paratenic hosts. Zoonotic in humans (Elkins and Nickol 1983; Richardson et al. 2017).

**Route of infection.** Ingestion of cystacanths in infected arthropod intermediate host or paratenic hosts (Mathison et al. 2016; Chancey et al. 2021).

**Site in human host.** Small intestine (Mathison et al. 2016; Chancey et al. 2021).

●●●●●● **Moniliformida Schmidt, 1972**

●●●●●●● **Moniliformidae Van Cleave, 1924**

**Genus *Moniliformis* Travassos, 1915**

***Moniliformismoniliformis* (Bremser, 1811)**

*Echinorhynchusgrassi* Railliet, 1893

*Echinorhynchuscanis* Porta, 1914

*Echinorhynchusbelgicus* Railliet, 1918

*Moniliformismoniliformissiciliensis* Meyer, 1932

*Moniliformismoniliformisagypticus* Meyer, 1932

*Moniliformisdubius* Meyer, 1932

**Geographic distribution.** Worldwide (Mathison et al. 2016).

**Natural hosts.** Intermediate hosts are insects, primarily beetles and cockroaches. Definitive hosts are primarily rodents and carnivores. Zoonotic in humans (Mathison et al. 2016).

**Route of infection.** Ingestion of cystacanths in infected insect intermediate host (Mathison et al. 2016).

**Site in human host.** Small intestine (Mathison et al. 2016).

●●●●●● **Echinorhynchida Petrotschenko, 1956**

●●●●●●● **Echinorhynchidae Cobbold, 1876**

**Genus *Acanthocephalus* Koelreuther, 1771**

***Acanthocephalusrauschi* (Schmidt, 1969)**

**Geographic distribution.** Alaska (Schmidt 1969).

**Natural hosts.** Alaskan grayling (*Thymallusarcticus*). Otters can serve as paratenic hosts. Zoonotic in humans (Schmidt 1969).

**Route of infection.** Unknown; presumed ingestion of cystacanths in infected intermediate host.

**Site in human host.** Peritoneum (Schmidt 1969, 1971).

**Genus *Pseudoacanthocephalus* Petrotschenko, 1958**

***Pseudoacanthocephalusbufonis* (Shipley, 1903)**

*Acanthocephalussinensis* Van Cleave, 1937

**Geographic distribution.** Southeast Asia, Hawaii (Barton and Pichelin 1999; Bush et al. 2009).

**Natural hosts.** Toads. Zoonotic in humans (Bush et al. 2009).

**Route of infection.** Unknown; presumed ingestion of cystacanths in infected intermediate host.

**Site in human host.** Small intestine (Schmidt 1971).

●●●●●● **Polymorphida Petrotschenko, 1956**

●●●●●●● **Polymorphidae Meyer, 1931**

**Genus *Bolbosoma* Porta, 1908**

***Bolbosomanipponicum* Yamaguti, 1939**

**Geographic distribution.** North Atlantic (Yamaguti 1963).

**Natural hosts.** Marine crustaceans serve as intermediate hosts. Fish and cephalopods may serve as paratenic hosts. Whales and seals serve as definitive hosts. Zoonotic in humans (Yamaguti 1963).

**Route of infection.** Unknown; presumed ingestion of cystacanths in infected fish paratenic hosts.

**Site in human host.** Small intestine (Yamamoto et al. 2018).

**Bolbosoma sp. cf. capitatum (von Linstow, 1880)**

**Geographic distribution.** Worldwide (Amin and Margolis 1998).

**Natural hosts.** Marine crustaceans serve as intermediate hosts. Whales serve as definitive hosts. Zoonotic in humans (Amin and Margolis 1998).

**Route of infection.** Unknown; presumed ingestion of cystacanths in infected fish paratenic hosts.

**Site in human host.** Small intestine (Arizono et al. 2012).

**Notes.** The single case was reported from Japan as Bolbosomacf.capitatim based on molecular and histological examination (Arizono et al. 2012).

**Genus *Corynosoma* Lühe, 1904**

***Corynosomastrumosum* (Rudolphi, 1802)**

**Geographic distribution.** Worldwide (Aznar et al. 2006; Leidenberger et al. 2020).

**Natural hosts.** Intermediate hosts are marine amphipods. Several groups of fish may serve as paratenic hosts. Definitive hosts are seals, walrus, beluga whale. Zoonotic in humans (Aznar et al. 2006; Leidenberger et al. 2020; Nickol et al. 2002).

**Route of infection.** Ingestion of cystacanths in infected fish paratenic host (Schmidt 1971).

**Site in human host.** Small intestine (Schmidt 1971).

***Corynosomavillosum* Van Cleave, 1953**

**Geographic distribution.** North Pacific (Moles 1982).

**Natural hosts.** Intermediate hosts are marine amphipods. Several groups of fish may serve as paratenic hosts. Definitive hosts are pinnipeds and whales. Zoonotic in humans (Moles 1982).

**Route of infection.** Ingestion of cystacanths in infected fish paratenic host (Fujita et al. 2016).

**Site in human host.** Small intestine (Fujita et al. 2016).

**Corynosoma sp. cf. validum Van Cleave, 1953**

**Geographic distribution.** North Pacific (Ionita et al. 2008).

**Natural hosts.** Intermediate hosts are marine amphipods. Several groups of fish may serve as paratenic hosts. Definitive host is the northern fur seal (*Callorhinusursinus*), and other pinnipeds. Zoonotic in humans (Ionita et al. 2008).

**Route of infection.** Ingestion of cystacanths in infected fish paratenic host (Takahashi et al. 2016).

**Site in human host.** Small intestine (Takahashi et al. 2016).

**Notes.** The single case was reported from Japan as Corynosomacf.validum based on DNA sequencing (Takahashi et al. 2016).

●●●●● **Annelida Lamarck, 1802**

●●●●●● **Hirudinea Lamarck, 1818**

●●●●●●● **Rhynchobdellida Blanchard, 1894**

●●●●●●●● **Glossiphoniidae Vaillant, 1890**

**Genus *Haementeria* de Filippi, 1849**

***Haementeriaacuecueyetzin* Oceguera-Figueroa, 2008**

**Geographic distribution.** Mexico (Oceguera-Figueroa 2008).

**Natural hosts.** Various vertebrates. Zoonotic on humans (Oceguera-Figueroa 2008; Charruau et al. 2020).

**Route of infection.** Exposure to fresh water (Oceguera-Figueroa 2008; Charruau et al. 2020).

**Site in human host.** Skin (Oceguera-Figueroa 2008).

**Vectored pathogens.** None.

***Haementeriaghilianii* de Filippi, 1849**

**Geographic distribution.** Amazon South America (Sawyer et al. 1981).

**Natural hosts.** Various mammals. Zoonotic on humans (Sawyer et al. 1981; Farrar 2014).

**Route of infection.** Exposure to fresh water (Farrar 2014).

**Site in human host.** Skin (Farrar 2014).

**Vectored pathogens.** None.

**Genus *Parabdella* Autrum, 1936**

***Parabdellaquadrioculata* (Moore, 1930)**

**Geographic distribution.** East Asia (Moore 1930).

**Natural hosts.** Freshwater turtles and frogs. Zoonotic on humans (Moore 1930).

**Route of infection.** Exposure to fresh water (Yamauchi et al. 2013).

**Site in human host.** Skin (Yamauchi et al. 2013).

**Vectored pathogens.** None.

**Genus *Placobdella* Blanchard, 1893**

***Placobdellacostata* (Müller, 1846)**

*Placobdellacatenigera* Moquin-Tandon, 1846

**Geographic distribution.** Mediterranean Region, Central Europe (Wilkialis 1973).

**Natural hosts.** Freshwater turtles. Zoonotic on humans (Wilkialis 1973).

**Route of infection.** Exposure to fresh water (Aloto 2018).

**Site in human host.** Skin (Aloto 2018).

**Vectored pathogens.** None.

●●●●●●● **Hirudiniformes Caballero, 1952**

●●●●●●●● **Xerobdellidae Moore, 1946**

**Genus *Diestecostoma* Vaillant, 1890**

*Heterobdella* Baird, 1869

*Hygrobdella* Caballero, 1940

***Diestecostomamexicana* (Baird, 1869)**

**Geographic distribution.** Central America (Oceguera-Figueroa and León-Règagnon 2014).

**Natural hosts.** Various mammals. Zoonotic on humans (Farrar 2014).

**Route of infection.** Environmental exposure (Farrar 2014).

**Site in human host.** Skin (Farrar 2014).

**Vectored pathogens.** None.

●●●●●●●● **Haemadipsidae Blanchard, 1896**

**Genus *Haemadipsa* Tennent, 1859**

***Haemadipsahainana* Song, 1977**

**Geographic distribution.** Southeast Asia (Tessler et al. 2018).

**Natural hosts.** Various mammals and birds. Zoonotic on humans (Tessler et al. 2018).

**Route of infection.** Environmental exposure (Tessler et al. 2018).

**Site in human host.** Skin (Tessler et al. 2018).

**Vectored pathogens.** None.

***Haemadipsainterrupta* Moore, 1935**

**Geographic distribution.** Southeast Asia (Tessler et al. 2018).

**Natural hosts.** Various mammals and birds. Zoonotic on humans (Tessler et al. 2018).

**Route of infection.** Environmental exposure (Tessler et al. 2018).

**Site in human host.** Skin (Tessler et al. 2018).

**Vectored pathogens.** None.

***Haemadipsajaponica* Whitman, 1886**

**Geographic distribution.** East Asia (Aizawa and Morishima 2018).

**Natural hosts.** Various mammals, birds, amphibians. Zoonotic on humans (Hanya et al. 2019).

**Route of infection.** Environmental exposure (Ji-Tuan 1997).

**Site in human host.** Skin, mucous membranes or the rectum and genital tract (Ji-Tuan 1997).

**Vectored pathogens.** None.

***Haemadipsapicta* Moore, 1929**

**Geographic distribution.** Southeast Asia (Lai et al. 2011).

**Natural hosts.** Various large mammals, including humans (Lai et al. 2011).

**Route of infection.** Environmental exposure (Fogden and Proctor 1985; Lai et al. 2011).

**Site in human host.** Skin (Fogden and Proctor 1985; Lai et al. 2011).

**Vectored pathogens.** None.

***Haemadipsarjukjuana* Oka, 1910**

**Geographic distribution.** East Asia (Lai et al. 2011; Won et al. 2014).

**Natural hosts.** Various mammals, including humans, and birds (Lai et al. 2011).

**Route of infection.** Environmental exposure (Lai et al. 2011; Won et al. 2014).

**Site in human host.** Skin (Lai et al. 2011; Won et al. 2014).

**Vectored pathogens.** None

***Haemadipsasylvestris* Blanchard, 1894**

**Geographic distribution.** Southeast Asia (Farrar 2014).

**Natural hosts.** Various mammals. Zoonotic on humans (Farrar 2014).

**Route of infection.** Environmental exposure (Farrar 2014).

**Site in human host.** Skin (Farrar 2014).

**Vectored pathogens.** None.

***Haemadipsatrimaculosa* Ngamprasertwong et al., 2007**

**Geographic distribution.** Southeast Asia (Ngamprasertwong et al. 2007; Tessler et al. 2018).

**Natural hosts.** Various mammals and birds. Zoonotic on humans (Ngamprasertwong et al. 2007).

**Route of infection.** Environmental exposure (Tessler et al. 2018).

**Site in human host.** Skin (Tessler et al. 2018).

**Vectored pathogens.** None

***Haemadipsazeylanica* (Moquin-Tandon, 1826)**

**Geographic distribution.** Japan, Southeast Asia (Fogden and Proctor 1985).

**Natural hosts.** Various amphibians and mammals. Zoonotic on humans (Fogden and Proctor 1985).

**Route of infection.** Environmental exposure (Fogden and Proctor 1985).

**Site in human host.** Skin (Fogden and Proctor 1985).

**Vectored pathogens.** None.

**Genus *Phytobdella* Blanchard, 1894**

***Phytobdellacatenifera* Moore, 1942**

**Geographic distribution.** Malaysia (Farrar 2014).

**Natural hosts.** Reptiles. Zoonotic on humans (Farrar 2014).

**Route of infection.** Environmental exposure (Farrar 2014).

**Site in human host.** Skin (Farrar 2014).

**Vectored pathogens.** None.

●●●●●●●● **Hirudinidae Whitman, 1886**

**Genus *Hirudo* Linnaeus, 1758**

***Hirudomedicinalis* Linnaeus, 1758**

**Geographic distribution.** Europe, Asia (Kutschera and Elliott 2014).

**Natural hosts.** Immature stages usually on amphibians. Adults on mammals. Zoonotic on humans (Kutschera and Elliott 2014).

**Route of infection.** Exposure to fresh water (Kutschera and Elliott 2014).

**Site in human host.** Skin (Kutschera and Elliott 2014).

**Vectored pathogens.***Aeromonas* spp. (Graf 1999).

***Hirudonipponia* Whitman, 1886**

**Geographic distribution.** Russia, Japan, Southeast Asia (Yang 1996).

**Natural hosts.** Immature stages usually on amphibians. Adults on mammals. Zoonotic on humans (Yang 1996).

**Route of infection.** Exposure to fresh water (Oda et al. 1984).

**Site in human host.** Skin, Eye (Oda et al. 1984).

**Vectored pathogens.** None.

***Hirudoorientalis* Utevsky & Trontelj, 2005**

**Geographic distribution.** Central Asia, Eastern Europe (Utevsky and Trontelj 2005).

**Natural hosts.** Immature stages usually on amphibians. Adults on mammals. Zoonotic on humans (Utevsky and Trontelj 2005).

**Route of infection.** Exposure to fresh water (Utevsky and Trontelj 2005).

**Site in human host.** Skin, Eye (Utevsky and Trontelj 2005).

**Vectored pathogens.***Aeromonas* spp. (Laufer et al. 2008).

***Hirudotroctina* Johnson, 1816**

**Geographic distribution.** North Africa, Iberian Peninsula (Marrone and Canale 2019).

**Natural hosts.** Immature stages usually on amphibians. Adults on mammals. Zoonotic on humans (Trontelj and Utevsky 2005).

**Route of infection.** Exposure to fresh water (Trontelj and Utevsky 2005).

**Site in human host.** Skin, Eye (Trontelj and Utevsky 2005).

**Vectored pathogens.** None.

***Hirudoverbena* Carena, 1820**

**Geographic distribution.** Mediterranean Europe (Marrone and Canale 2019).

**Natural hosts.** Immature stages usually on amphibians. Adults on mammals. Zoonotic on humans (Kutschera and Elliott 2014).

**Route of infection.** Exposure to fresh water (Kutschera and Elliott 2014).

**Site in human host.** Skin (Kutschera and Elliott 2014).

**Vectored pathogens.***Aeromonas* spp. (Laufer et al. 2008).

**Genus *Hirudinaria* Whitman, 1886**

***Hirudinariamanillensis* (Lesson, 1842)**

*Hirudomultistriata* Schmarda, 1861

*Hirudoluzoniae* Kinberg, 1866

*Hirudomaculosa* Grube, 1868

*Hirudomaculata* Baird, 1869

*Limnatisgranulosa* Blanchard, 1893

*Hirudoboyntoni* Wharton, 1913

**Geographic distribution.** Southeast Asia (Jeratthitikul et al. 2020).

**Natural hosts.** Mammals, including buffalo, cattle. Zoonotic in humans (Jeratthitikul et al. 2020).

**Route of infection.** Exposure to fresh water (Hii et al. 1978).

**Site in human host.** Upper respiratory tract (Hii et al. 1978).

**Vectored pathogens.** None

**Genus *Limnatis* Moquin-Tandon, 1827**

***Limnatisnilotica* (Savigny, 1822)**

**Geographic distribution.** Southern Europe, North Africa, Middle East (Arfuso et al. 2019).

**Natural hosts.** Mammals, including sheep, cattle, dogs, and donkeys. Zoonotic on humans (Arfuso et al. 2019).

**Route of infection.** Exposure to fresh water

**Site in human host.** Upper respiratory tract (Almallah 1968; Ağin et al. 2008).

**Vectored pathogens.** None.

**Genus *Poecilobdella* Blanchard, 1893**

***Poecilobdellagranulosa* (Savigny, 1822)**

**Geographic distribution.** India, Sri Lanka, Southeast Asia (Nesemann and Sharma 2001).

**Natural hosts.** Amphibians, Mammals. Zoonotic in humans (Nesemann and Sharma 2001).

**Route of infection.** Exposure to fresh water (Zainuddin et al. 2016).

**Site in human host.** Skin, upper respiratory tract (Zainuddin et al. 2016).

**Vectored pathogens.** None.

***Poecilobdellaviridis* Moore in Harding & Moore, 1927**

**Geographic distribution.** India, Sri Lanka (Kulkarni et al. 1977).

**Natural hosts.** Amphibians, Mammals. Zoonotic in humans (Kulkarni et al. 1977).

**Route of infection.** Exposure to fresh water (Zainuddin et al. 2016).

**Site in human host.** Upper respiratory tract (Zainuddin et al. 2016).

**Vectored pathogens.** None.

●●●●●●●● **Macrobdellidae Richardson, 1969**

**Genus *Macrobdella* Verrill, 1872**

***Macrobdelladecora* (Say, 1824)**

**Geographic distribution.** Eastern North America, Mexico (Phillips et al. 2016, 2019).

**Natural hosts.** Various vertebrate animals. Zoonotic on humans (Phillips et al. 2016, 2019).

**Route of infection.** Exposure to fresh water (Phillips et al. 2016, 2019).

**Site in human host.** Skin (Phillips et al. 2016, 2019).

**Vectored pathogens.** None.

***Macrobdelladiplotertia* Meyer, 1975**

**Geographic distribution.** East-central United States (Phillips et al. 2016, 2019).

**Natural hosts.** Various vertebrate animals. Zoonotic on humans (Phillips et al. 2016, 2019).

**Route of infection.** Exposure to fresh water (Phillips et al. 2016, 2019).

**Site in human host.** Skin (Phillips et al. 2016, 2019).

**Vectored pathogens.** None.

***Macrobdelladitetra* Moore, 1953**

**Geographic distribution.** Southeastern United States (Phillips et al. 2016, 2019).

**Natural hosts.** Various vertebrate animals. Zoonotic on humans(Phillips et al. 2016, 2019).

**Route of infection.** Exposure to fresh water (Phillips et al. 2016, 2019).

**Site in human host.** Skin (Phillips et al. 2016, 2019).

**Vectored pathogens.** None.

***Macrobdellamimicus* Phillips, 2019**

**Geographic distribution.** Northeastern United States (Phillips et al. 2019).

**Natural hosts.** Various vertebrate animals. Zoonotic on humans (Phillips et al. 2019).

**Route of infection.** Exposure to fresh water (Phillips et al. 2019).

**Site in human host.** Skin (Phillips et al. 2019).

**Vectored pathogens.** None.

***Macrobdellasestertia* Whitman, 1886**

**Geographic distribution.** Northeastern United States (Phillips et al. 2016, 2019).

**Natural hosts.** Various vertebrate animals. Zoonotic on humans (Phillips et al. 2016, 2019).

**Route of infection.** Exposure to fresh water (Phillips et al. 2016, 2019).

**Site in human host.** Skin (Phillips et al. 2016, 2019).

**Vectored pathogens.** None.

●●●●●●●● **Praeobdellidae Sawyer, 1986**

**Genus *Dinobdella* Moore in Harding & Moore, 1927**

***Dinobdellaferox* (Blanchard, 1896)**

**Geographic distribution.** India, Southeast Asia (Lai 2019).

**Natural hosts.** Various mammals. Zoonotic on humans (Lai 2019).

**Route of infection.** Exposure to fresh water (Makiya et al. 1988).

**Site in human host.** Nasal mucosa (Makiya et al. 1988).

**Vectored pathogens.** None.

**Genus *Myxobdella* Oka, 1917**

***Myxobdellaafricana* Moore, 1939**

**Geographic distribution.** Sub-Saharan Africa (Cundall et al. 1986; Estambale et al. 1992).

**Natural hosts.** Mammals, including dogs. Zoonotic in humans (Cundall et al. 1986; Estambale et al. 1992).

**Route of infection.** Exposure to fresh water (Cundall et al. 1986; Estambale et al. 1992).

**Site in human host.** Upper respiratory tract (Cundall et al. 1986; Estambale et al. 1992).

**Vectored pathogens.** None.

**Genus *Tyrannobdella* Philipps et al., 2010**

***Tyrannobdellarex* Phillips et al., 2010**

**Geographic distribution.** Amazon South America (Phillips et al. 2010).

**Natural hosts.** Unknown; presumed zoonotic on humans (Phillips et al. 2010).

**Route of infection.** Exposure to fresh water (Phillips et al. 2010).

**Site in human host.** Nasopharynx (Phillips et al. 2010).

**Vectored pathogens.** None.

●●●●● **Cestoda Rudolphi, 1809**

●●●●●● **Eucestoda Southwell, 1930**

●●●●●●● **DiphyllobothriideaKuchta et al., 2008**

●●●●●●●● **Diphyllobothriidae Lühe, 1910**

**Genus *Adenocephalus* Nybelin, 1931**

***Adenocephaluspacificus* Nybelin, 1931**

*Adenocephalusseptentrionalis* Nybelin, 1931

*Diphyllobothriumarctocephali* Drummond, 1937

*Diphyllobothriumkrotovi* Delyamure, 1955

*Dibothriocephalusatlanticum* Delyamure & Parukhin, 1968

**Geographic distribution.** Coastal Pacific waters and southern Africa (Hernandez-Orts et al. 2015; Kuchta et al. 2015).

**Natural hosts.** The first arthropod intermediate host is unknown, but presumed to be marine copepods. The second intermediate hosts are various marine fish. Definitive hosts are eared seals and sea lions; dogs and jackals can serve as reservoir hosts. Zoonotic in humans (Hernandez-Orts et al. 2015; Kuchta et al. 2015).

**Route of infection.** Ingestion of plerocercoids in infected fish (Kuchta et al. 2015).

**Site in human host.** Small intestine (Kuchta et al. 2015).

**Genus *Dibothriocephalus* Lühe, 1899**

***Dibothriocephalusdalliae* (Rausch, 1956)**

**Geographic distribution.** Northern Pacific (Alaska and northeastern Siberia) (Scholz and Kuchta 2016).

**Natural hosts.** First intermediate hosts are freshwater copepods. Second intermediate hosts are freshwater fish, primarily Alaska blackfish (*Dalliapectoralis*). Definitive hosts are Arctic fox, with domestic dogs as reservoir hosts. Zoonotic in humans (Scholz and Kuchta 2016).

**Route of infection.** Ingestion of plerocercoids in infected fish (Scholz and Kuchta 2016).

**Site in human host.** Small intestine (Scholz and Kuchta 2016).

***Dibothriocephalusdendriticus* (Nitzsch, 1824)**

*Dibothriocephalusfissiceps* Creplin, 1829

*Dibothriocephaluscordiceps* Leidy, 1872

*Dibothriocephalusexile* Linton, 1892

*Sparganumsebago* Ward, 1910

*Dibothriocephalusminor* Cholodkovsky, 1916

*Diphyllobothriumcanadense* Cooper, 1921

*Diphyllobothriumstrictum* Talysin, 1932

*Diphyllobothriumobdoriense* Piotnikoff, 1933

*Diphyllobothriumlaruei* Vergeer, 1934

*Diphyllobothriumnenzi* Petrov, 1938

*Diphyllobothriumoblongatum* Thomas, 1946

*Diphyllobothriummedium* Fahmy, 1954

*Diphyllobothriummicrocordiceps* Szidat & Soria, 1957

*Diphyllobothriumnorvegicum* Vik, 1957

**Geographic distribution.** Holarctic (Scholz and Kuchta 2016).

**Natural hosts.** The first intermediate hosts are freshwater copepods. The second intermediate and paratenic hosts are many different freshwater fish. Definitive hosts are fish-eating birds, primarily gulls, and mammals, including wild and domestic canids, bears, and otters. Zoonotic in humans (Scholz and Kuchta 2016).

**Route of infection.** Ingestion of plerocercoids in infected fish (Kuchta et al. 2013).

**Site in human host.** Small intestine (Kuchta et al. 2013).

***Dibothriocephalushians* Diesing, 1850**

**Geographic distribution.** Mediterranean and Circumboreal (Scholz and Kuchta 2016).

**Natural hosts.** The first intermediate hosts are presumed to be marine copepods. The second intermediate hosts are unknown, but presumed to be marine fish. Definitive hosts are seals. Zoonotic in humans (Scholz and Kuchta 2016).

**Route of infection.** Ingestion of plerocercoids in infected fish (Scholz and Kuchta 2016).

**Site in human host.** Small intestine (Scholz and Kuchta 2016).

***Dibothriocephaluslatus* (Linnaeus, 1758)**

*Diphyllobothriumamericanum* Hall & Wigdor, 1918

*Diphyllobothriumtungussicum* Podyapolskaya & Gnedina, 1932

*Diphyllobothriumskrjabini* Plotnikoff, 1933

**Geographic distribution.** Holarctic, South America (Scholz and Kuchta 2016).

**Natural hosts.** The first intermediate hosts are freshwater copepods. The second intermediate and paratenic hosts are freshwater fish, including perch, pike, burbot, walleye, and pikeperch. Definitive hosts are fish-eating carnivores, including wild and domestic dogs and cats, bears, and mustelids. Zoonotic in humans (Scholz and Kuchta 2016).

**Route of infection.** Ingestion of plerocercoids in infected fish (Scholz et al. 2009).

**Site in human host.** Small intestine (Scholz et al. 2009).

***Dibothriocephalusnihonkaiensis* (Yamane et al., 1886)**

*Diphyllobothriumgiljacicum* Rutkevich, 1937

*Diphyllobothriumklebanovskii* Muratov & Posokov, 1988

**Geographic distribution.** Northern Pacific (Scholz and Kuchta 2016; Ikuno et al. 2018).

**Natural hosts.** First intermediate hosts are marine copepods. Second intermediate hosts are salmonid fish. Definitive hosts are bears, wild canids, and mink. Zoonotic in humans.

**Route of infection.** Ingestion of plerocercoids in infected fish (Scholz and Kuchta 2016).

**Site in human host.** Small intestine (Scholz and Kuchta 2016).

***Dibothriocephalusursi* (Rausch, 1954)**

*Diphyllobothriumgonodo* Yamaguti, 1942

**Geographic distribution.** Northern North America (Scholz and Kuchta 2016).

**Natural hosts.** First intermediate hosts are marine copepods. Second intermediate hosts are salmonid fish, primarily sockeye salmon (*Oncorhynchusnerka*). Definitive hosts are bears. Zoonotic in humans (Scholz and Kuchta 2016).

**Route of infection.** Ingestion of plerocercoids in infected fish (Scholz and Kuchta 2016).

**Site in human host.** Small intestine (Scholz and Kuchta 2016).

**Genus *Diphyllobothrium* Cobbold, 1858**

***Diphyllobothriumbalaenopterae* (Lönnberg, 1892)**

*Krabbeagrandis* Blanchard, 1894

*Diplogonoporusfukuokaensis* Kamo & Miyazaki, 1970

**Geographic distribution.** Worldwide (Scholz and Kuchta 2016).

**Natural hosts.** First intermediate hosts are marine copepods. Second intermediate hosts are marine fish, including Japanese anchovy, Japanese sardine, and skipjack tuna. Definitive hosts are baleen whales. Zoonotic in humans (Scholz and Kuchta 2016).

**Route of infection.** Ingestion of plerocercoids in infected fish (Scholz and Kuchta 2016).

**Site in human host.** Small intestine (Scholz and Kuchta 2016).

***Diphyllobothriumstemmacephalum* Cobbold, 1858**

*Diphyllobothriumponticum* Delyamure, 1971

*Diphyllobothriumyonagoense* Yamane et al., 1981

**Geographic distribution.** Northern Hemisphere (Scholz and Kuchta 2016).

**Natural hosts.** First intermediate hosts are presumed to be marine copepods. Second intermediate hosts are unknown, but presumed to be marine fish. Definitive hosts are dolphins and porpoises. Zoonotic in humans (Scholz and Kuchta 2016).

**Route of infection.** Ingestion of plerocercoids in infected fish (Scholz and Kuchta 2016).

**Site in human host.** Small intestine (Scholz and Kuchta 2016).

***Diphyllobothrium* , incertae sedis**:

***Diphyllobothriumcordatum* (Leuckart, 1863)**

**Geographic distribution.** Circumpolar (Scholz and Kuchta 2016).

**Natural hosts.** First intermediate hosts are presumed to be marine copepods. Second intermediate hosts are unknown, but presumed to be marine fish. Definitive hosts are seals and walrus. Zoonotic in humans (Scholz and Kuchta 2016).

**Route of infection.** Ingestion of plerocercoids in infected fish (Scholz and Kuchta 2016).

**Site in human host.** Small intestine (Scholz and Kuchta 2016).

***Diphyllobothriumlanceolatum* (Krabbe, 1865)**

**Geographic distribution.** Circumpolar (Scholz and Kuchta 2016).

**Natural hosts.** First intermediate hosts are marine copepods. Second intermediate hosts are marine fish, including sardine cisco (*Coregonussardinella*). Definitive hosts are seals. Zoonotic in humans (Scholz and Kuchta 2016).

**Route of infection.** Ingestion of plerocercoids in infected fish (Scholz and Kuchta 2016).

**Site in human host.** Small intestine (Scholz and Kuchta 2016).

**Genus *Spirometra* Faust et al., 1929**

***Spirometradecepiens* (Diesing, 1850)**

**Geographic distribution.** North and South America, Southeast Asia (Jeon et al. 2015; Jeon et al. 2018b).

**Natural hosts.** First intermediate hosts are believed to be freshwater copepods. Second intermediate hosts are believed to be freshwater fish and/or amphibians. Definitive hosts are wild and domestic cats; dogs may serve as reservoir hosts. Zoonotic in humans as dead-end hosts that harbor plerocercoid larvae (Jeon et al. 2015; Jeon et al. 2018b).

**Route of infection.** Ingestion of plerocercoids in infected paratenic or intermediate host; ingestion of water containing copepods infected with procercoids (Jeon et al. 2015; Jeon et al. 2018b).

**Site in human host.** Skin and soft tissues, pleural and peritoneal cavities, abdominal viscera, eye, CNS (Jeon et al. 2015; Jeon et al. 2018b).

***Spirometraerinaceieuropaei* (Rudolphi, 1819)**

**Geographic distribution.** Europe, Asia, and Australia (Tang et al. 2017; Kuchta et al. 2021).

**Natural hosts.** First intermediate hosts are freshwater copepods. Second intermediate hosts are amphibians, reptiles and mammals. Definitive hosts are believed to be wild and domestic canids and felids. Zoonotic in humans as dead-end hosts that harbor plerocercoid larvae (Tang et al. 2017; Kuchta et al. 2021).

**Route of infection.** Ingestion of plerocercoids in infected paratenic or intermediate host; ingestion of water containing copepods infected with procercoids (Lee et al. 1984; Tang et al. 2017).

**Site in human host.** Skin and soft tissues, pleural and peritoneal cavities, abdominal viscera, eye, CNS as plerocercoid larvae (spargana); rarely as adults in small intestine (Lee et al. 1984; Tang et al. 2017).

***Spirometramansoni* (Joyeux & Houdemer, 1928)**

**Geographic distribution.** Southeast Asia (Hong et al. 2016).

**Natural hosts.** First intermediate hosts are freshwater copepods. Second intermediate hosts are amphibians, while reptiles, birds, and pigs may serve as paratenic hosts. Definitive hosts are felids and canids. Zoonotic in humans as dead-end hosts that harbor plerocercoid larvae (Hong et al. 2016).

**Route of infection.** Ingestion of plerocercoids in infected paratenic or intermediate host; ingestion of water containing copepods infected with procercoids (Galan-Puchades 2019).

**Site in human host.** Skin and soft tissues, pleural and peritoneal cavities, abdominal viscera, eye, CNS (Galan-Puchades 2019).

***Spirometramansonoides* (Mueller, 1935)**

**Geographic distribution.** North America (McHale et al. 2020).

**Natural hosts.** First intermediate hosts are freshwater copepods. Second intermediate hosts are amphibians, reptiles, birds, small mammals. Definitive hosts are felids and canids. Zoonotic in humans as dead-end hosts that harbor plerocercoid larvae (McHale et al. 2020).

**Route of infection.** Ingestion of plerocercoids in infected paratenic or intermediate host; ingestion of water containing copepods infected with procercoids (Landero et al. 1991).

**Site in human host.** Skin and soft tissues, pleural and peritoneal cavities, abdominal viscera, eye, CNS (Landero et al. 1991).

***Spirometraranarum* (Gastaldi, 1854)**

**Geographic distribution.** Africa, Southeast Asia (Jeon et al. 2018a; Saksirisampant et al. 2020).

**Natural hosts.** First intermediate hosts are freshwater copepods. Second intermediate hosts are amphibians and reptiles. Definitive hosts are wild and domestic felids and canids. Zoonotic in humans as dead-end hosts that harbor plerocercoid larvae (Jeon et al. 2018a; Saksirisampant et al. 2020).

**Route of infection.** Ingestion of infected intermediate or paratenic host (Saksirisampant et al. 2020).

**Site in human host.** Skin and soft tissues, pleural and peritoneal cavities, abdominal viscera, eye, CNS (Saksirisampant et al. 2020).

***Spirometra* spp.**

**Geographic distribution.** East Africa (Eberhard et al. 2015).

**Natural hosts.** Unknown (Eberhard et al. 2015).

**Route of infection.** Either ingestion of plerocercoids in undercooked intermediate or paratenic hosts, or water containing copepods infected with procercoids (Eberhard et al. 2015).

**Site in human host.** Skin and soft tissues (Eberhard et al. 2015).

**Notes.** Several infections with *Spirometra* sp. have been confirmed in South Sudan and sporadically in other East African countries. Molecular data have confirmed distinctiveness from other known zoonotic *Spirometra* spp., but no specific identification has been determined (Eberhard et al. 2015).

**Diphyllobothriidae, incertae sedis**:

***Sparganumproliferum* (Ilima, 1905)**

**Geographic distribution.** Undefined (Mueller and Strano 1974).

**Natural hosts.** Unknown, currently known only from humans in which it is believed to be zoonotic (Mueller and Strano 1974).

**Route of infection.** Unknown, but presumed to be ingestion of infected intermediate or paratenic host (Mueller and Strano 1974).

**Site in human host.** Skin and soft tissues, pleural and peritoneal cavities, abdominal viscera, eye, CNS (Mueller and Strano 1974).

**Notes.** The name *Sparganumproliferum* is the name given to an enigmatic parasite of unknown affinities. To date, it is only known from its larval form from humans.

●●●●●●● **Cyclophyllidea van Beneden in Braun, 1990**

●●●●●●●● **Dipylidiidae Stiles, 1896**

**Genus *Dipylidium* Leuckart, 1863**

*Microtaenia* Sedgwick in Claus & Sedwick, 1884

***Dipylidiumcaninum* (Linnaeus, 1758)**

**Geographic distribution.** Worldwide (Sapp and Bradbury 2020).

**Natural hosts.** Intermediate hosts are insects, primarily fleas (*Ctenocephalides*, *Pulex*) and lice (*Trichodectes*). Definitive hosts are wild and domestic felids and canids. Zoonotic in humans (Sapp and Bradbury 2020).

**Route of infection.** Ingestion of cysticercoids in infected arthropod intermediate host (Cabello et al. 2011; Sapp and Bradbury 2020).

**Site in human host.** Small intestine (Cabello et al. 2011; Sapp and Bradbury 2020).

●●●●●●●● **Hymenolepididae Ariola, 1899**

**Genus *Hymenolepis* Weinland, 1858**

***Hymenolepisdiminuta* (Rudolphi, 1819)**

*Taeniaflavopunctata* Weinland, 1858

**Geographic distribution.** Worldwide (Arai 1980).

**Natural hosts.** Intermediate hosts are insects, commonly granary beetles. Definitive hosts are rodents (primarily rats). Zoonotic in humans (Arai 1980).

**Route of infection.** Ingestion of cysticercoids infected insect intermediate host (Tiwari et al. 2014).

**Site in human host.** Small intestine (Tiwari et al. 2014).

***Hymenolepishibernia* Montgomery et al., 1987**

**Geographic distribution.** Europe, Asia (Sargison et al. 2018).

**Natural hosts.** Intermediate hosts are beetles. Definitive hosts are mice in the genus *Apodemus*. Zoonotic in humans (Sargison et al. 2018).

**Route of infection.** Presumed ingestion of cysticercoids in infected insect intermediate host (Nkouawa et al. 2016).

**Site in human host.** Small intestine (Nkouawa et al. 2016).

**Genus *Rodentolepis* Spasskii, 1954**

***Rodentolepismicrostoma* (Dujardin, 1845)**

**Geographic distribution.** Worldwide (Cunningham and Olson 2010).

**Natural hosts.** Intermediate hosts are insects, commonly granary beetles and fleas. Definitive hosts are fleas. Zoonotic in humans (Andreassen et al. 2004; Cunningham and Olson 2010).

**Route of infection.** Presumably, ingestion of cysticercoids in infected insect intermediate host (Macnish et al. 2003).

**Site in human host.** Small intestine (Macnish et al. 2003).

**Notes.** The few cases of *H.microstoma* in humans have been diagnosed by molecular detection in stool (Macnish et al. 2003).

***Rodentolepisnana* (Bilharz, 1851)**

*Hymenolepisfraterna* Stiles, 1906

**Geographic distribution.** Worldwide (Sataeva et al. 2018).

**Natural hosts.** Intermediate hosts are insects, commonly granary beetles and fleas. Definitive hosts are rodents. Zoonotic in humans, although human-to-human transmission can occur (Sataeva et al. 2018).

**Route of infection.** Ingestion of cysticercoids in infected insect intermediate host; ingestion of eggs on fecally contaminated fomites (Thompson 2015; Sataeva et al. 2018).

**Site in human host.** Small intestine (Thompson 2015; Sataeva et al. 2018).

●●●●●●●● **Taeniidae Ludwig, 1886**

**Genus *Echinococcus* Rudolphi, 1801**

***Echinococcuscanadensis* Webster & Cameron, 1961**

*Echinococcusgranulosusborealis* Williams & Sweatman, 1863

*Echinococcus* G6, camel-pig strain

*Echinococcus* G7, camel-pig strain

*Echinococcus* G8, American cervid strain

*Echinococcus* G10, Fennoscandian strain

**Geographic distribution.** North America, Europe, Middle East, China, east Africa (Romig et al. 2015; Hua et al. 2019).

**Natural hosts.** Intermediate hosts are camels, pigs, goats, and deer. Definitive hosts are wild and domestic canids. Zoonotic in humans as dead-end hosts harboring larval cysts (Romig et al. 2015; Hua et al. 2019).

**Route of infection.** Ingestion of eggs in food, water, fomites contaminated with dog feces (Romig et al. 2015).

**Site in human host.** Disseminated infection, common sites of colonization are liver, lung, brain, bone (Romig et al. 2015).

***Echinococcusequinus* Willams & Sweatman, 1963**

*Echinococcus* G4, horse strain

**Geographic distribution.** Europe, North Africa, Middle East (Romig et al. 2015).

**Natural hosts.** Intermediate hosts are horses. Definitive hosts are wild and domestic canids. Zoonotic in humans as dead-end hosts harboring larval cysts (Romig et al. 2015).

**Route of infection.** Ingestion of eggs in food, water, fomites contaminated with canid feces (Romig et al. 2015; Kim et al. 2020).

**Site in human host.** Disseminated infection, common sites of colonization are liver, lung, brain, bone (Kim et al. 2020).

***Echinococcusgranulosus* (Batsch, 1796)**

*Echinococcusminimus* Cameron, 1926

*Echinococcuslongimanubrius* Cameron, 1926

*Echinococcuscameroni* Ortlepp, 1934

*Echinococcuslycaontis* Ortlepp, 1934

*Echinococcusintermedius* Lopez-Neyra & Soler Planas, 1943

*Echinococcuspatagonicus* Szidat, 1960

*Echinococcuscepanzoi* Szidat, 1971

*Echinococcus* G1, sheep-buffalo strain

*Echinococcus* G2, sheep-buffalo strain

*Echinococcus* G3, sheep-buffalo strain

**Geographic distribution.** Worldwide (Eckert and Deplazes 2004; Romig et al. 2015).

**Natural hosts.** Intermediate hosts are primarily sheep, goats, cattle, and buffalo. Definitive hosts are wild and domestic canids. Zoonotic in humans as dead-end hosts harboring larval cysts (Eckert and Deplazes 2004; Romig et al. 2015).

**Route of infection.** Ingestion of eggs in food, water, fomites contaminated with canid feces (Eckert and Deplazes 2004; Romig et al. 2015).

**Site in human host.** Disseminated infection, common sites of colonization are liver, lung, brain, bone (Eckert and Deplazes 2004; Romig et al. 2015).

***Echinococcusmultilocularis* (Leuckart, 1863)**

*Echinococcussibiricensis* Rausch & Schiller, 1964

*Echinococcusrussicensis* Tang et al., 2007

**Geographic distribution.** Europe, Asia, North America (Eckert and Deplazes 2004; Conraths and Deplazes 2015).

**Natural hosts.** Intermediate hosts are rodents, primarily voles. Definitive hosts are wild canids, primarily red fox (*Vulpesvulpes*); raccoon dogs, domestic dogs, and domestic cats may serve as reservoir hosts. Zoonotic in humans as dead-end hosts harboring larval cysts (Conraths and Deplazes 2015; Eckert and Deplazes 2004).

**Route of infection.** Ingestion of eggs in food, water, fomites contaminated with canid feces (Eckert and Deplazes 2004; Conraths and Deplazes 2015).

**Site in human host.** Liver, with dissemination to other organs, such as lung, heart, brain, bone (Eckert and Deplazes 2004; Conraths and Deplazes 2015).

***Echinococcusoligarthra* Diesing, 1863**

*Echinococcuscruzi* Brumpt & Joyeux, 1924

*Echinococcuspampeanus* Szidat, 1967

**Geographic distribution.** South America (D’Alessandro and Rausch 2008).

**Natural hosts.** Intermediate hosts are rodents and marsupials. Definitive hosts are wild felids. Zoonotic in humans as dead-end hosts harboring larval cysts (D’Alessandro and Rausch 2008).

**Route of infection.** Ingestion of eggs in food, water, fomites contaminated with felid feces (D’Alessandro and Rausch 2008).

**Site in human host.** Liver, peritoneal and pleural cavities, with dissemination to other organs (D’Alessandro and Rausch 2008).

***Echinococcusortleppi* Lopez-Neyra & Soler Planas, 1943**

*Echinococcus* G5, cattle strain

**Geographic distribution.** Africa, Europe, India, South America (Romig et al. 2015).

**Natural hosts.** Intermediate hosts are primarily bovids. Definitive hosts are wild and domestic canids. Zoonotic in humans as dead-end hosts harboring larval cysts (Romig et al. 2015).

**Route of infection.** Ingestion of eggs in food, water, fomites contaminated with canid feces (Romig et al. 2015).

**Site in human host.** Disseminated infection, common sites of colonization are liver, lung, brain, bone (Romig et al. 2015).

***Echinococcusvogeli* Rausch & Berstein, 1972**

**Geographic distribution.** South America (D’Alessandro and Rausch 2008).

**Natural hosts.** Intermediate hosts are rodents, primarily paca (*Cuniculuspaca*). Definitive hosts are bush dogs (*Speothosvenaticus*). Zoonotic in humans as dead-end hosts harboring larval cysts (D’Alessandro and Rausch 2008).

**Route of infection.** Ingestion of eggs in food, water, fomites contaminated with bush dog feces (D’Alessandro and Rausch 2008).

**Site in human host.** Liver, peritoneal and pleural cavities, with dissemination to other organs (D’Alessandro and Rausch 2008).

**Genus *Hydatigera* Lamarck, 1816**

***Hydatigerataeniaeformis* (Batsch, 1786)**

*Cysticercusfasciolaris* Rudolphi, 1808

**Geographic distribution.** Worldwide (Nakao et al. 2013; Guo 2020).

**Natural hosts.** Intermediate hosts are primarily rodents and lagomorphs. Definitive hosts are felids. Zoonotic in humans (Nakao et al. 2013; Guo 2020).

**Route of infection.** Unknown

**Site in human host.** Small intestine, liver (Stĕrba and Barus 1976; Guo 2020).

**Genus *Taenia* Linnaeus, 1758**

***Taeniabrauni* Setti, 1897**

**Geographic distribution.** Africa (Deplazes et al. 2019).

**Natural hosts.** Intermediate hosts are rodents and non-human primates. Definitive hosts are canids and genets. Zoonotic in humans as dead-end hosts harboring larval cysts (Deplazes et al. 2019).

**Route of infection.** Ingestion of eggs in food, water, fomites contaminated with canid feces (Lescano and Zunt 2013).

**Site in human host.** Skin and soft tissues, eyes (Vanderick et al. 1964; Lescano and Zunt 2013; Deplazes et al. 2019).

**Notes.***Taeniabrauni* is often considered a subspecies or synonym of *T.serialis*.

***Taeniacrassiceps* (Zeder, 1800)**

*Taeniahyperborea* von Linstow, 1905

**Geographic distribution.** Northern Hemisphere (Ntoukas et al. 2013).

**Natural hosts.** Intermediate hosts are rodents. Definitive hosts are canids, primarily foxes Zoonotic in humans as dead-end hosts harboring larval cysts (Freeman 2011; Ntoukas et al. 2013).

**Route of infection.** Ingestion of eggs in food, water, fomites contaminated with canid feces (Ntoukas et al. 2013; Tappe et al. 2016a).

**Site in human host.** Skin and soft tissues, eyes, CNS (Ntoukas et al. 2013; Tappe et al. 2016a).

***Taeniaglomeratus* (Railliett & Henry, 1915)**

**Geographic distribution.** Africa (Deplazes et al. 2019).

**Natural hosts.** Intermediate hosts are rodents. Definitive hosts are canids and genets. Zoonotic in humans as dead-end hosts harboring larval cysts (Lescano and Zunt 2013; Deplazes et al. 2019).

**Route of infection.** Ingestion of eggs in food, water, fomites contaminated with canid feces (Lescano and Zunt 2013; Deplazes et al. 2019).

**Site in human host.** Eyes (Lescano and Zunt 2013; Deplazes et al. 2019).

**Notes.***Taeniaglomeratus* is often considered a subspecies or synonym of *T.serialis*.

***Taeniamartis* (Zeder, 1803)**

**Geographic distribution.** North America, Europe (Brunet et al. 2015).

**Natural hosts.** Intermediate hosts include rodents. Definitive hosts are mustelids and foxes. Zoonotic in humans has a dead-end host harboring larval cysts (Eberwein et al. 2013; Brunet et al. 2015; Deplazes et al. 2019).

**Route of infection.** Ingestion of eggs in food, water, fomites contaminated with feces of definitive host (Brunet et al. 2015; Deplazes et al. 2019).

**Site in human host.** Skin and soft tissues, CNS, eyes (Eberwein et al. 2013; Brunet et al. 2015; Deplazes et al. 2019).

***Taeniamulticeps* Leske, 1790**

**Geographic distribution.** Worldwide (Varcasia et al. 2015).

**Natural hosts.** Intermediate hosts are ungulates, primarily sheep. Definitive hosts are canids, primarily foxes. Zoonotic in humans as dead-end hosts harboring larval cysts (Varcasia et al. 2015; Deplazes et al. 2019).

**Route of infection.** Ingestion of eggs in food, water, fomites contaminated with canid feces (Deplazes et al. 2019).

**Site in human host.** Skin and soft tissues, CNS (Deplazes et al. 2019).

***Taeniasaginata* Goeze, 1782**

*Taeniaconfusa* Ward, 1896

*Taeniaafricana* von Linstow, 1900

*Taeniahominis* von Linstow, 1904

**Geographic distribution.** Worldwide (Hoberg 2002; Braae et al. 2018).

**Natural hosts.** Intermediate hosts are cattle. Definitive hosts are humans (Braae et al. 2018; Hoberg 2002).

**Route of infection.** Ingestion of cysticercoids in infected cattle (Braae et al. 2018).

**Site in human host.** Small intestine (Braae et al. 2018).

***Taeniaserialis* Gervais, 1847**

*Multicepsradians* Joyeux et al., 1922

**Geographic distribution.** Worldwide; highest prevalence in Africa (Schneider-Crease et al. 2017).

**Natural hosts.** Intermediate hosts include rabbits, rodents, cattle, sheep, goats. Definitive hosts are wild and domestic canids. Zoonotic in humans as dead-end hosts harboring larval cysts (Schneider-Crease et al. 2017).

**Route of infection.** Ingestion of eggs in food, water, fomites contaminated with canid feces (Tappe et al. 2016a).

**Site in human host.** CNS (Tappe et al. 2016a).

***Taeniasolium* Linnaeus, 1758**

*Cysticercuscellulosae* Gmelin, 1790

**Geographic distribution.** Worldwide (Garcia et al. 2003; Nakao et al. 2002; Coral-Almeida et al. 2015).

**Natural hosts.** Intermediate hosts are pigs. Definitive hosts are humans, although in cases of cysticercosis humans function as dead-end intermediate hosts (Garcia et al. 2003; Coral-Almeida et al. 2015).

**Route of infection.** Ingestion of cysticercoids in infected pigs (taeniasis); ingestion of eggs in food, water, fomites contaminated with human feces (cysticercosis) (Garcia et al. 2003).

**Site in human host.** Small intestine (taeniasis); disseminated infection with predilection for CNS (cysticercosis) (Garcia et al. 2003).

***Taeniasuihominis* Mathison et al., 2021**

*Taeniaasiatica* Eom & Rim, 1993

*Taeniasaginataasiatica* Eom & Rim, 1993

*Taeniaasiaticus* Eom et al., 2020

**Geographic distribution.** East Asia (Ale et al. 2014).

**Natural hosts.** Intermediate hosts are pigs. Definitive hosts are humans (Eom and Rim 1993; Ale et al. 2014).

**Route of infection.** Ingestion of cysticerci in infected pigs (Ale et al. 2014; Eom and Rim 1993).

**Site in human host.** Small intestine (Eom and Rim 1993; Ale et al. 2014).

**Genus *Versteria*Nakao et al., 2013**

**Geographic distribution.** Europe, Central Asia, North America (Nakao et al. 2013; Deplazes et al. 2019).

**Natural hosts.** Intermediate hosts are rodents; non-human primates can be incidental hosts. Definitive hosts are mustelids. Zoonotic in humans (Nakao et al. 2013; Deplazes et al. 2019).

**Route of infection.** Presumably by the ingestion of eggs in food, water, fomites contaminated with feces of definitive host (Deplazes et al. 2019).

**Site in human hose.** Various organs, disseminated infection (Barkati et al. 2018; Lehman et al. 2019).

**Notes.** Human and primate *Versteria* infections have not been identified to species level but bear close genetic resemblance to isolates from North American mink and are somewhat similar to isolates of *V.mustelae* (Gmelin, 1790) from European mink (Lee et al. 2016; Lehman et al. 2019).

●●●●●●●● **Anoplocephalidae Cholodkovsky, 1902**

**Genus *Bertiella* Stiles & Hassall, 1902**

*Bertia* Blanchard, 1891

***Bertiellamucronotata* (Meyner, 1895)**

**Geographic distribution.** South America, Caribbean (Sapp and Bradbury 2020).

**Natural hosts.** First intermediate hosts are oribatid mites. Definitive hosts are New World monkeys. Zoonotic in humans (Sapp and Bradbury 2020).

**Route of infection.** Ingestion of cysticercoids in infected mite intermediate host (Sapp and Bradbury 2020).

**Site in human host.** Small intestine (Sapp and Bradbury 2020).

***Bertiellastuderi* (Blanchard, 1891)**

**Geographic distribution.** Africa, Asia (Sapp and Bradbury 2020).

**Natural hosts.** First intermediate hosts are oribatid mites. Definitive hosts are Old World monkeys and non-human apes. Zoonotic in humans (Sapp and Bradbury 2020).

**Route of infection.** Ingestion of cysticercoids in infected mite intermediate host (Sapp and Bradbury 2020).

**Site in human host.** Small intestine (Sapp and Bradbury 2020).

**Genus *Inermicapsifer* Janicki, 1910**

***Inermicapsifermadagascariensis* (Davaine, 1870)**

*Acanthocephalaarvicanthidis* Kofend, 1917

*Raillietinacubensis* Kouri, 1938

**Geographic distribution.** Sub-Saharan Africa, South America, West Indies, Southeast Asia (Goldsmid and Muir 1972; Sapp and Bradbury 2020).

**Natural hosts.** Intermediate hosts are not known, but presumed to be terrestrial arthropods. Definitive hosts are rodents and hyraxes. Zoonotic in humans (Goldsmid and Muir 1972; Sapp and Bradbury 2020).

**Route of infection.** Ingestion of cysticercoids in infected arthropod intermediate host (Sapp and Bradbury 2020).

**Site in human host.** Small intestine (Sapp and Bradbury 2020).

**Genus *Mathevotaenia* Akhumyan, 1946**

***Mathevotaeniasymmetrica* (Baylis, 1927)**

**Geographic distribution.** Europe, Asia (Lamon and Greer 1986).

**Natural hosts.** Intermediate hosts are suspected as being arthropods. Definitive hosts are rodents. Zoonotic in humans (Lamon and Greer 1986).

**Route of infection.** Presumably, ingestion of cysticercoids in infected arthropod intermediate host (Lamon and Greer 1986).

**Site in human host.** Small intestine (Lamon and Greer 1986).

**Genus *Moniezia* Blanchard, 1891**

***Monieziaexpansa* (Rudolphi, 1810)**

**Geographic distribution.** Worldwide (single human case from Egypt) (Elliott 1986; el-Shazly et al. 2004).

**Natural hosts.** Intermediate hosts are mites. Definitive hosts are sheep, goats, cattle. Zoonotic in humans (Elliott 1986; el-Shazly et al. 2004).

**Route of infection.** Presumably, ingestion of cysticercoids in infected arthropod intermediate host (el-Shazly et al. 2004).

**Site in human host.** Small intestine (el-Shazly et al. 2004).

●●●●●●●● **Davaineidae Braun, 1900**

**Genus *Raillietina* Fuhrman, 1920**

***Raillietinacelebensis* (Janicki, 1902)**

*Taeniaasiatica* von Linstow, 1891

*Taeniaformosana* Akashi, 1916

*Raillietinafunerebris* Meggitt & Subramanian, 1927

*Raillietinagarrisoni* Tubangui, 1931

*Raillietinasinensis* Hsu, 1935

*Raillietinamurium* Joyeux & Baer, 1938

**Geographic distribution.** Asia, Australia (Baer and Sandars 1956; Sapp and Bradbury 2020).

**Natural hosts.** Intermediate hosts are invertebrates, including insects (primarily ants and beetles) and terrestrial mollusks. Definitive hosts are rodents and shrews. Zoonotic in humans (Baer and Sandars 1956; Sapp and Bradbury 2020).

**Route of infection.** Ingestion of cysticercoids in infected arthropod intermediate host (Baer and Sandars 1956; Sapp and Bradbury 2020).

**Site in human host.** Small intestine (Baer and Sandars 1956; Sapp and Bradbury 2020).

***Raillietinademerariensis* (Daniels, 1895)**

*Raillietinaquitensis* Leon, 1935

*Raillietinabrumpti* Doilfus, 1939

*Raillietinaequatoriensis* Dollfus, 1939

*Raillietinaleoni* Dollfus, 1939

*Raillietinaluisaleoni* Dollfus, 1939

*Raillietinahalli* Perez-Vigueras, 1934

**Geographic distribution.** South America, Caribbean (Baer and Sandars 1956; Sapp and Bradbury 2020).

**Natural hosts.** Intermediate hosts are invertebrates, including insects (primarily ants and beetles) and terrestrial mollusks. Definitive hosts are rodents; non-human primates may serve as reservoir hosts. Zoonotic in humans (Baer and Sandars 1956; Sapp and Bradbury 2020).

**Route of infection.** Ingestion of cysticercoids in infected arthropod intermediate host (Baer and Sandars 1956; Sapp and Bradbury 2020).

**Site in human host.** Small intestine (Baer and Sandars 1956; Sapp and Bradbury 2020).

***Raillietinasiriraji* Chandler & Pradatsundarasar, 1957**

**Geographic distribution.** Thailand (Sapp and Bradbury 2020).

**Natural hosts.** Intermediate hosts are invertebrates, including insects (primarily ants and beetles). Definitive hosts are rodents. Zoonotic in humans (Sapp and Bradbury 2020).

**Route of infection.** Ingestion of cysticercoids in infected arthropod intermediate host (Sapp and Bradbury 2020).

**Site in human host.** Small intestine (Sapp and Bradbury 2020).

●●●●●●●● **Mesocestoididae Fuhrmann, 1907**

**Genus *Mesocestoides* Valliant, 1863**

***Mesocestoideslineatus* (Goeze, 1782)**

**Geographic distribution.** Europe and Asia (Sapp and Bradbury 2020).

**Natural hosts.** The complete life cycle and presumed number of hosts is not completely understood. The presumed first intermediate host is believed to be an arthropod. The second intermediate hosts are a variety of vertebrates, including small mammals, birds, reptiles, and amphibians. Definitive hosts are carnivores, including foxes, wolves, raccoon dogs, and European badgers. Zoonotic in humans (Padgett and Boyce 2004; Sapp and Bradbury 2020).

**Route of infection.** Unknown; presumed to be ingestion of tetrathyridia in meat and viscera of infected second intermediate hosts (Sapp and Bradbury 2020).

**Site in human host.** Small intestine (Sapp and Bradbury 2020).

***Mesocestoidesvariabilis* Mueller, 1928**

**Geographic distribution.** North America (Sapp and Bradbury 2020).

**Natural hosts.** The complete life cycle and presumed number of hosts is not completely understood. The presumed first intermediate host is believed to be an arthropod. The second intermediate hosts are a variety of vertebrates, including small mammals, birds, reptiles, and amphibians. Definitive hosts include wild and domestic canids and felids, mustelids, and opossums. Zoonotic in humans (Padgett and Boyce 2004; Sapp and Bradbury 2020).

**Route of infection.** Unknown; presumed to be ingestion of tetrathyridia in meat and viscera of infected second intermediate hosts (Sapp and Bradbury 2020).

**Site in human host.** Small intestine (Sapp and Bradbury 2020).

●●●●● **Trematoda Rudolphi, 1808**

●●●●●● **Digenea Carus, 1863**

●●●●●●● **Diplostomida Olson et al., 2003**

●●●●●●●● **Diplostomata Olson et al., 2003**

●●●●●●●●● **Brachylaeimidae Joyeux & Foley, 1930**

**Genus *Brachylaeima* Dujardin, 1843**

***Brachylaeimacribbi* Butcher & Grove, 2001**

**Geographic distribution.** Australia (Chai and Jung 2020).

**Natural hosts.** First intermediate hosts are terrestrial snails in the genus *Theba*. Second intermediate hosts are land snails in the genus *Cernualla*. Definitive hosts include a variety of birds, mammals, and reptiles. Zoonotic in humans (Chai and Jung 2020).

**Route of infection.** Ingestion of metacercariae in infected snail intermediate hosts (Chai and Jung 2020).

**Site in human host.** Small intestine (Chai and Jung 2020).

●●●●●●●●● **Cyathocotylidae Mühling, 1898**

**Genus *Prohemistomum* Odhner, 1913**

***Prohemistomumvivax* (Sonsino, 1892)**

**Geographic distribution.** Europe, North Africa, Middle East (Nasr 1941; Chai and Jung 2020).

**Natural hosts.** First intermediate hosts are freshwater snails in the genus *Cleopatra*. Second intermediate hosts are various brackish water and freshwater fish. Definitive hosts are felids, canids, and birds. Zoonotic in humans (Nasr 1941; Chai and Jung 2020).

**Route of infection.** Ingestion of metacercariae in infected fish intermediate hosts (Nasr 1941; Chai and Jung 2020)

**Site in human host.** Small intestine (Nasr 1941; Chai and Jung 2020).

●●●●●●●●● **Diplostomidae Poirier, 1886**

**Genus *Alaria* Schrank, 1788**

***Alariaamericana* Hall & Wigdor, 1918**

**Geographic distribution.** North America (Möhl et al. 2009).

**Natural hosts.** First intermediate hosts are snails in the genera *Planorbis*, *Heliosoma*, *Lymnea*, and *Anisus*. Second intermediate hosts are amphibians; many vertebrate animals can serve as paratenic hosts. Definitive hosts are carnivores. Zoonotic in humans (Möhl et al. 2009).

**Route of infection.** Ingestion of metacercariae in undercooked game meat (Möhl et al. 2009).

**Site in human host.** Subcutaneous tissues, eyes, lungs, disseminated infections (Freeman et al. 1976; Möhl et al. 2009).

**Genus *Fibricola* Dubois, 1932**

***Fibricolacratera* (Barker & Noll, 1915)**

**Geographic distribution.** North America (Hoffman 1955; Chai and Jung 2020).

**Natural hosts.** First intermediate hosts are freshwater snails in the genus *Physa*. Second intermediate hosts are amphibians. Definitive hosts are rodents and shrews. Zoonotic in humans (Hoffman 1955; Chai and Jung 2020).

**Route of infection.** Ingestion of metacercariae in infected amphibian intermediate hosts (Chai and Jung 2020).

**Site in human host.** Small intestine (Chai and Jung 2020).

**Genus *Neodiplostomum* Railliet, 1919**

***Neodiplostomumseoulense* Seo et al., 1964**

**Geographic distribution.** China, South Korea (Chai and Jung 2020).

**Natural hosts.** First intermediate hosts are freshwater snails in the genera *Hippeutis* and *Segmentina*. Second intermediate hosts are amphibians; snakes can serve as paratenic hosts. Definitive hosts are rodents and humans (Chai and Jung 2020).

**Route of infection.** Ingestion of metacercariae in infected reptile and amphibian intermediate and paratenic hosts (Chai and Jung 2020).

**Site in human host.** Small intestine (Chai and Jung 2020).

●●●●●●●●● **Strigeidae Railliet, 1919**

**Genus *Cotylurus* Szidat, 1928**

***Cotylurusjaponicus* Ishii, 1932**

**Geographic distribution.** Central and Southeast Asia, Russia, Japan (Chai and Jung 2020).

**Natural hosts.** First intermediate hosts are unknown but hypothesized to be freshwater snails. Second intermediate hosts are unknown but presumed to be freshwater snails. Definitive hosts are ducks. Zoonotic in humans (Chai and Jung 2020).

**Route of infection.** Ingestion of metacercariae in infected insect intermediate hosts (Chai and Jung 2020).

**Site in human host.** Small intestine (Chai and Jung 2020).

●●●●●●●●● **Schistosomatidae Stiles & Hassall, 1898**

**Genus *Schistosoma* Weinland, 1858**

***Schistosomabovis* (Bilharz, 1852)**

**Geographic distribution.** Southern Europe, Middle East, South Asia, Africa (Christensen et al. 1983).

**Natural hosts.** First intermediate hosts are snails in the genus *Bulinus*. Definitive hosts are ruminants, primarily cattle. Zoonotic in humans (Christensen et al. 1983).

**Route of infection.** Penetration of skin by free-swimming cercariae

**Site in human host.** Mesenteric veins of the large intestine (Chunge et al. 1986; Mouchet et al. 1988)

**Notes.** Human infections with hybrids of *S.bovis* and *S.haematobium* have been confirmed using molecular methods (Oleaga et al. 2019).

***Schistosomaguineensis* Pagès et al., 2003**

*Schistosomaintercalatum*, Guinea strain

**Geographic distribution.** West Africa (Webster et al. 2006).

**Natural hosts.** First intermediate hosts are snails in the genus *Bulinus*. Definitive hosts are humans, rodents, ruminants (Webster et al. 2006).

**Route of infection.** Penetration of skin by free-swimming cercariae (Webster et al. 2006).

**Site in human host.** Mesenteric veins of the large intestine (Webster et al. 2006).

***Schistosomahaematobium* (Bilharz, 1852)**

**Geographic distribution.** Africa, isolated regions of the Middle East (Colley et al. 2014; Gautret et al. 2015).

**Natural hosts.** First intermediate hosts are snails in the genus *Bulinus*. Definitive hosts are humans (Colley et al. 2014).

**Route of infection.** Penetration of skin by free-swimming cercariae (Colley et al. 2014).

**Site in human host.** Venous plexus of the urinary bladder (Colley et al. 2014).

**Notes.** Human infection of hybrids of *S.haematobium* and *S.bovis* have been documented in Corsica, France (Oleaga et al. 2019).

***Schistosomaintercalatum* Fisher, 1934**

**Geographic distribution.** Democratic Republic of the Congo (Webster et al. 2006; Colley et al. 2014).

**Natural hosts.** First intermediate hosts are snails in the genus *Bulinus*. Definitive hosts are humans, rodents, ruminants (Colley et al. 2014).

**Route of infection.** Penetration of skin by free-swimming cercariae (Colley et al. 2014).

**Site in human host.** Mesenteric veins of the large intestine (Colley et al. 2014).

***Schistosomajaponicum* (Katsurada, 1904)**

**Geographic distribution.** China, Philippines, Indonesia (Sulawesi) (Colley et al. 2014).

**Natural hosts.** First intermediate hosts are snails in the genus *Oncomelania*. Definitive hosts include a variety of mammals, including canids, felids, pigs, ruminants, rodents, and humans (Colley et al. 2014).

**Route of infection.** Penetration of skin by free-swimming cercariae (Colley et al. 2014).

**Site in human host.** Mesenteric veins of the small and large intestine (Colley et al. 2014).

***Schistosomamansoni* Sambon, 1907**

**Geographic distribution.** Sub-Saharan Africa, South America, Caribbean, parts of the Arabian Peninsula (Colley et al. 2014).

**Natural hosts.** First intermediate hosts are snails in the genus *Biomphalaria*. Definitive hosts are humans, occasionally non-human primates (Colley et al. 2014).

**Route of infection.** Penetration of skin by free-swimming cercariae (Colley et al. 2014).

**Site in human host.** Mesenteric veins of the large intestine (Colley et al. 2014).

***Schistosomamattheei* Veglia & LeRoux, 1929**

**Geographic distribution.** Africa, Middle East (Christensen et al. 1983).

**Natural hosts.** First intermediate hosts are snails in the genus *Bulinus*. Definitive hosts are wild and domestic ruminants. Zoonotic in humans (Christensen et al. 1983).

**Route of infection.** Penetration of skin by free-swimming cercariae (Christensen et al. 1983).

**Site in human host.** Presumed to be venous plexus of the urinary bladder (Wolmarans et al. 1990; Cnops et al. 2020).

**Notes.** Reports of patent, egg-producing human infections with *S.mattheei* are most likely a result of hybridization of this species with *S.haematobium*. Dead-end acute infections with *S.mattheei* × *S.haematobium* have been confirmed using molecular methods (Kruger and Evans 2009; Cnops et al. 2020).

***Schistosomamekongi* Voge et al., 1978**

**Geographic distribution.** Mekong River Valley of Cambodia and Laos (Colley et al. 2014).

**Natural hosts.** First intermediate host is the snail *Neotriculaaperta*. Definitive hosts include a variety of mammals, including canids, felids, pigs, ruminants, rodents, and humans (Colley et al. 2014).

**Route of infection.** Penetration of skin by free-swimming cercariae (Colley et al. 2014).

**Site in human host.** Mesenteric veins of the large intestine (Colley et al. 2014).

**Cercarial dermatitis**

Many species of avian, and some mammalian, schistosomes cause a limited cutaneous reaction in humans referred to as cercarial dermatitis (or “swimmer’s itch”). The identity of causative agents is typically inferred via environmental investigations following outbreaks and may or may not be identified to species level; only the major genera are listed here:

**Genus *Anserobilharzia* Brandt et al., 2013**

**Geographic distribution.** Northern Hemisphere (Brant et al. 2013).

**Natural hosts.** Intermediate hosts are freshwater snails in the family Planorbidae. Definitive hosts are charadriiform birds. Zoonotic in humans (Brant et al. 2013; Horák et al. 2015).

**Route of infection.** Penetration of skin by free-swimming cercariae (Horák et al. 2015).

**Site in human host.** Skin (Horák et al. 2015).

**Genus *Austrobilharzia* Johnston, 1917**

**Geographic distribution.** Northern Hemisphere, Australia (Farley 1971).

**Natural hosts.** Intermediate hosts are freshwater snails in the family Planorbidae. Definitive hosts are charadriiform birds. Zoonotic in humans (Farley 1971).

**Route of infection.** Penetration of skin by free-swimming cercariae (Horák et al. 2015).

**Site in human host.** Skin (Horák et al. 2015).

**Genus *Bilharziella* Looss, 1899**

**Geographic distribution.** Europe (Prüter et al. 2017).

**Natural hosts.** Intermediate hosts are freshwater snails in the family Planorbidae. Definitive hosts are waterfowl and other wading birds. Zoonotic in humans (Prüter et al. 2017).

**Route of infection.** Penetration of skin by free-swimming cercariae (Horák et al. 2015).

**Site in human host.** Skin (Horák et al. 2015).

**Genus *Bivitellobilharzia* Vogel & Minning, 1940**

**Geographic distribution.** Africa, Asia (Devkota et al. 2014).

**Natural hosts.** Snail intermediate hosts are unknown. Definitive hosts are elephants and rhinoceroses. Zoonotic in humans (Devkota et al. 2014).

**Route of infection.** Penetration of skin by free-swimming cercariae (Horák et al. 2015).

**Site in human host.** Skin (Horák et al. 2015).

**Genus *Gigantobilharzia* Odhner, 1910**

**Geographic distribution.** North America (McDonald 1981).

**Natural hosts.** Intermediate hosts are freshwater snails in the family Physidae. Definitive hosts are passerine birds. Zoonotic in humans (McDonald 1981).

**Route of infection.** Penetration of skin by free-swimming cercariae (Horák et al. 2015).

**Site in human host.** Skin (Horák et al. 2015).

**Genus *Heterobilharzia* Price, 1929**

**Geographic distribution.** North America (Graham et al. 2021).

**Natural hosts.** Intermediate hosts are freshwater snails in the family Lymnaeidae. Definitive hosts are various mammals, including carnivores and marsupials. Zoonotic in humans (Graham et al. 2021).

**Route of infection.** Penetration of skin by free-swimming cercariae (Horák et al. 2015).

**Site in human host.** Skin (Horák et al. 2015).

**Genus *Ornithobilharzia* Odhner, 1912**

**Geographic distribution.** Worldwide (Farley 1971)

**Natural hosts.** Intermediate hosts are marine snails in the family Batillaridae. Definitive hosts are various birds and mammals. Zoonotic in humans (Farley 1971).

**Route of infection.** Penetration of skin by free-swimming cercariae (Horák et al. 2015).

**Site in human host.** Skin (Horák et al. 2015).

**Genus *Trichobilharzia* Skrjabin & Zakharow, 1920**

**Geographic distribution.** Worldwide (Horák et al. 2002).

**Natural hosts.** Intermediate hosts are freshwater snails in the families Lymnaeidae and Physidae. Definitive hosts are waterfowl. Zoonotic in humans (Horák et al. 2002).

**Route of infection.** Penetration of skin by free-swimming cercariae (Horák et al. 2015).

**Site in human host.** Skin (Horák et al. 2015).

●●●●●●● **Plagiorchiida La Rue, 1957**

●●●●●●●● **Bucephalata La Rue, 1926**

●●●●●●●●● **Gymnophallidae Odhner, 1905**

**Genus *Gymnophalloides* Fujita, 1925**

***Gymnophalloidesseoi* Chai et al., 2003**

**Geographic distribution.** South Korea (Lee and Chai 2001; Chai and Jung 2020).

**Natural hosts.** First intermediate hosts are unknown. Second intermediate hosts are oysters in the genus *Crassostrea*. Definitive hosts are oystercatchers (*Haematopus*). Zoonotic in humans (Lee and Chai 2001; Chai and Jung 2020).

**Route of infection.** Ingestion of metacercariae in infected oyster intermediate hosts (Lee and Chai 2001; Chai and Jung 2020),

**Site in human host.** Small intestine (Lee and Chai 2001; Chai and Jung 2020).

●●●●●●●● **Echinostomata La Rue, 1926**

●●●●●●●●● **Himasthlidae Odhner, 1910**

**Genus *Acanthoparyphium* Dietz, 1909**

***Acanthoparyphiumtyosenense* Yamaguti, 1939**

**Geographic distribution.** Korea, Japan (Toledo and Esteban 2016; Chai and Jung 2020).

**Natural hosts.** First intermediate hosts are marine gastropods. The second intermediate hosts are brackish water bivalves. Definitive hosts are ducks. Zoonotic in humans (Toledo and Esteban 2016; Chai and Jung 2020).

**Route of infection.** Ingestion of metacercariae in infected bivalves (Toledo and Esteban 2016; Chai and Jung 2020).

**Site in human host.** Small intestine (Toledo and Esteban 2016; Chai and Jung 2020).

**Genus *Himasthla* Dietz, 1909**

***Himasthlamuehlensi* Vogel, 1933**

**Geographic distribution.** North America, Colombia (Toledo and Esteban 2016; Chai and Jung 2020).

**Natural hosts.** First intermediate hosts are unknown, presumed to be marine gastropods. Second intermediate hosts are presumed to be marine clams and mussels. Definitive hosts are birds. Zoonotic in humans (Toledo and Esteban 2016; Chai and Jung 2020).

**Route of infection.** Ingestion of metacercariae in infected mollusk intermediate hosts (Toledo and Esteban 2016; Chai and Jung 2020).

**Site in human host.** Small intestine (Toledo and Esteban 2016; Chai and Jung 2020).

●●●●●●●●● **Echinochasmidae Odhner, 1910**

**Genus *Echinochasmus* Dietz, 1909**

***Echinochasmuscaninus* (Verma, 1935)**

**Geographic distribution.** Southeast Asia, India (Toledo and Esteban 2016; Chai and Jung 2020).

**Natural hosts.** First intermediate hosts are unknown. Second intermediate hosts are freshwater fish in the genus *Macropodus*. Definitive hosts are wild and domestic canids. Zoonotic in humans (Toledo and Esteban 2016; Chai and Jung 2020).

**Route of infection.** Ingestion of metacercariae in infected fish intermediate host (Toledo and Esteban 2016; Chai and Jung 2020).

**Site in human host.** Small intestine (Toledo and Esteban 2016; Chai and Jung 2020).

***Echinochasmusfujianensis* Chen, in Chen et al., 1992**

**Geographic distribution.** China (Toledo and Esteban 2016; Chai and Jung 2020).

**Natural hosts.** First intermediate hosts are freshwater snails in the genus *Bellamya* Second intermediate hosts are freshwater fish in the genera *Pseudorasbora* and *Cyprinus*. Definitive hosts are wild and domestic canids and felids, pigs, and rodents. Zoonotic in humans (Toledo and Esteban 2016; Chai and Jung 2020).

**Route of infection.** Ingestion of metacercariae in infected fish intermediate host (Toledo and Esteban 2016; Chai and Jung 2020).

**Site in human host.** Small intestine (Toledo and Esteban 2016; Chai and Jung 2020).

***Echinochastmusjaponicus* Tanabe, 1926**

**Geographic distribution.** Southeast Asia, Japan (Toledo and Esteban 2016; Chai and Jung 2020).

**Natural hosts.** First intermediate hosts are freshwater snails, including the genus *Parafossarulis*. Second intermediate hosts are various freshwater fish. Definitive hosts are felids, insectivores, and birds. Zoonotic in humans (Toledo and Esteban 2016; Chai and Jung 2020).

**Route of infection.** Ingestion of metacercariae in infected fish intermediate host (Toledo and Esteban 2016; Chai and Jung 2020).

**Site in human host.** Small intestine (Toledo and Esteban 2016; Chai and Jung 2020).

***Echinochasmusjiufoensis* Liang & Ke, 1988**

**Geographic distribution.** China (Toledo and Esteban 2016; Chai and Jung 2020).

**Natural hosts.** First intermediate hosts are unknown. Second intermediate hosts are presumed to be freshwater fish or mollusks. Definitive hosts are felids, canids, and pigs. Zoonotic in humans (Toledo and Esteban 2016; Chai and Jung 2020).

**Route of infection.** Ingestion of metacercariae in infected intermediate host (Toledo and Esteban 2016; Chai and Jung 2020).

**Site in human host.** Small intestine (Toledo and Esteban 2016; Chai and Jung 2020).

***Echinochasmusliliputanus* (Looss, 1896)**

**Geographic distribution.** China, Middle East, North Africa (Toledo and Esteban 2016; Chai and Jung 2020).

**Natural hosts.** First intermediate hosts are freshwater snails in the genus *Parafossarulus*. Second intermediate hosts are freshwater fish in the genera *Pseudorasbora* and *Carassius*. Definitive hosts are felids, canids, raccoons, and badgers. Zoonotic in humans (Toledo and Esteban 2016; Chai and Jung 2020).

**Route of infection.** Ingestion of metacercariae in infected intermediate fish host or water contaminated with metacercariae (Toledo and Esteban 2016; Chai and Jung 2020).

**Site in human host.** Small intestine (Toledo and Esteban 2016; Chai and Jung 2020).

***Echinochasmusperfoliatus* (Ratz, 1908)**

**Geographic distribution.** Southeast Asia, Russia, Europe, North Africa (Toledo and Esteban 2016; Chai and Jung 2020).

**Natural hosts.** First intermediate hosts are various freshwater snails (*Parafossarulus*, *Bithynia*, *Lymnaea*). Second intermediate hosts are various freshwater fish (*Zacco*, *Carassius*, *Pseudorasbora*). Definitive hosts are felids, canids, pigs, and birds. Zoonotic in humans (Toledo and Esteban 2016; Chai and Jung 2020).

**Route of infection.** Ingestion of metacercariae in infected fish intermediate host (Toledo and Esteban 2016; Chai and Jung 2020).

**Site in human host.** Small intestine (Toledo and Esteban 2016; Chai and Jung 2020).

●●●●●●●●● **Echinostomatidae Looss, 1899**

**Genus *Artyfechinostomum* Lane, 1915**

***Artyfechinostomummalayanum* (Leiper, 1911)**

**Geographic distribution.** Southeast Asia (Toledo and Esteban 2016; Chai and Jung 2020).

**Natural hosts.** First intermediate hosts are freshwater snails in the genera *Indoplanorbis* and *Gyraulus*. Second intermediate hosts are several freshwater snails. Definitive hosts include pigs, rodents, shrews, felids, canids. Zoonotic in humans (Toledo and Esteban 2016; Chai and Jung 2020).

**Route of infection.** Ingestion of metacercariae in infected snail intermediate hosts (Toledo and Esteban 2016; Chai and Jung 2020).

**Site in human host.** Small intestine (Toledo and Esteban 2016; Chai and Jung 2020).

***Artyfechinostomumoraoni* Bandyopadhyay et al., 1989**

**Geographic distribution.** India (Toledo and Esteban 2016; Chai and Jung 2020).

**Natural hosts.** First intermediate hosts are snails in the genus *Lymnaea*. Second intermediate hosts and definitive hosts are unknown; presumed zoonotic in humans (Toledo and Esteban 2016; Chai and Jung 2020).

**Route of infection.** Ingestion of metacercariae in infected intermediate hosts (Toledo and Esteban 2016; Chai and Jung 2020).

**Site in human host.** Small intestine (Toledo and Esteban 2016; Chai and Jung 2020).

***Artyfechinostomumsufratyfex* Lane, 1915**

*Cercariamehrai* Faruqui, 1930

**Geographic distribution.** India, Vietnam (Toledo and Esteban 2016; Chai and Jung 2020).

**Natural hosts.** First intermediate hosts are snails in the genera *Indoplanorbis* and *Lymnaea*. Second intermediate hosts include freshwater snails, fish, and amphibians. Definitive hosts are pigs, canids, and rodents. Zoonotic in humans (Toledo and Esteban 2016; Chai and Jung 2020).

**Route of infection.** Ingestion of metacercariae in infected intermediate hosts (Toledo and Esteban 2016; Chai and Jung 2020).

**Site in human host.** Small intestine (Toledo and Esteban 2016; Chai and Jung 2020).

**Genus *Echinoparyphium* Dietz, 1909**

***Echinoparyphiumrecurvatum* (von Linstow, 1873)**

**Geographic distribution.** North America, Europe, North Africa, Middle East, Central and Southeast Asia (Toledo and Esteban 2016; Chai and Jung 2020).

**Natural hosts.** First intermediate hosts are snails in the genera *Physa* and *Lymnaea*. Second intermediate hosts include freshwater snails and amphibians. Definitive hosts are canids and rodents. Zoonotic in humans (Toledo and Esteban 2016; Chai and Jung 2020).

**Route of infection.** Ingestion of metacercariae in infected intermediate hosts (Toledo and Esteban 2016; Chai and Jung 2020).

**Site in human host.** Small intestine (Toledo and Esteban 2016; Chai and Jung 2020).

**Genus *Echinostoma* Rudolphi, 1809**

***Echinostomaaegyptica* Khalil & Abaza, 1924**

**Geographic distribution.** North Africa, Middle East, Southeast Asia, Japan (Toledo and Esteban 2016; Chai and Jung 2020).

**Natural hosts.** First and second intermediate hosts are unknown. Definitive hosts are rats. Zoonotic in humans (Toledo and Esteban 2016; Chai and Jung 2020).

**Route of infection.** Ingestion of metacercariae in infected intermediate hosts (Toledo and Esteban 2016; Chai and Jung 2020).

**Site in human host.** Small intestine (Toledo and Esteban 2016; Chai and Jung 2020).

***Echinostomaangustitestes* Wang, 1977**

**Geographic distribution.** China (Toledo and Esteban 2016; Chai and Jung 2020).

**Natural hosts.** First intermediate hosts are presumed to be freshwater snails. Second intermediate hosts are freshwater fish. Definitive hosts are dogs and livestock. Zoonotic in humans (Toledo and Esteban 2016; Chai and Jung 2020).

**Route of infection.** Ingestion of metacercariae in infected fish intermediate hosts (Toledo and Esteban 2016; Chai and Jung 2020).

**Site in human host.** Small intestine (Toledo and Esteban 2016; Chai and Jung 2020).

***Echinostomacinetorchis* Ando & Ozaki, 1923**

**Geographic distribution.** Southeast Asia, Japan (Toledo and Esteban 2016; Chai and Jung 2020).

**Natural hosts.** First intermediate hosts are snails in the genera *Hippeutis* and *Segmentina*. Second intermediate hosts include freshwater snails, fish (*Misgurnus*), and amphibians. Definitive hosts are rats. Zoonotic in humans (Toledo and Esteban 2016; Chai and Jung 2020).

**Route of infection.** Ingestion of metacercariae in infected intermediate hosts (Toledo and Esteban 2016; Chai and Jung 2020).

**Site in human host.** Small intestine (Toledo and Esteban 2016; Chai and Jung 2020).

***Echinostomailocanum* (Garrison, 1908)**

**Geographic distribution.** Central and Southeast Asia (Toledo and Esteban 2016; Chai and Jung 2020).

**Natural hosts.** First intermediate hosts are snails in the genera *Gyraulus* and *Hippeutis*. Second intermediate hosts include freshwater snails in the genera *Pila* and *Vivaparus*. Definitive hosts are rats and canids. Zoonotic in humans (Toledo and Esteban 2016; Chai and Jung 2020).

**Route of infection.** Ingestion of metacercariae in infected snail intermediate hosts(Toledo and Esteban 2016; Chai and Jung 2020).

**Site in human host.** Small intestine (Toledo and Esteban 2016; Chai and Jung 2020).

***Echinostomalindoense* Sandground & Bonne, 1940**

**Geographic distribution.** Europe, Southeast Asia, South America (Toledo and Esteban 2016; Chai and Jung 2020).

**Natural hosts.** First intermediate hosts are several genera of freshwater snails. Second intermediate hosts include freshwater snails and mussels. Definitive hosts include a variety of mammals and birds. Zoonotic in humans (Toledo and Esteban 2016; Chai and Jung 2020).

**Route of infection.** Ingestion of metacercariae in infected mollusk intermediate hosts (Toledo and Esteban 2016; Chai and Jung 2020).

**Site in human host.** Small intestine (Toledo and Esteban 2016; Chai and Jung 2020).

***Echinostomamacrorchis* Ando & Ozaki, 1923**

**Geographic distribution.** Southeast Asia, Japan (Toledo and Esteban 2016; Chai and Jung 2020).

**Natural hosts.** First intermediate hosts are snails in the genera *Segmentina* and *Gyraulus*. Second intermediate hosts include freshwater snails (*Segmentina*) and amphibians. Definitive hosts are rodents and wading birds. Zoonotic in humans (Toledo and Esteban 2016; Chai and Jung 2020).

**Route of infection.** Ingestion of metacercariae in infected intermediate hosts (Toledo and Esteban 2016; Chai and Jung 2020).

**Site in human host.** Small intestine(Toledo and Esteban 2016; Chai and Jung 2020).

***Echinostomaparaensei* Lie & Basch, 1967**

**Geographic distribution.** Australia, Brazil (Toledo and Esteban 2016; Chai and Jung 2020).

**Natural hosts.** First and second intermediate hosts are snails in the genus *Biomphalaria*. Definitive hosts are rats. Zoonotic in humans (Toledo and Esteban 2016; Chai and Jung 2020).

**Route of infection.** Ingestion of metacercariae in infected snail intermediate hosts (Toledo and Esteban 2016; Chai and Jung 2020).

**Site in human host.** Small intestine (Toledo and Esteban 2016; Chai and Jung 2020).

***Echinostomarevolutum* (Froelich, 1802)**

**Geographic distribution.** Europe, Russia, Middle East, Central and Southeast Asia, North America (Toledo and Esteban 2016; Chai and Jung 2020).

**Natural hosts.** First intermediate hosts include several genera of freshwater snails. Second intermediate hosts include freshwater snails and clams, and amphibians. Definitive hosts are felids, canids, rodents, and birds. Zoonotic in humans (Toledo and Esteban 2016; Chai and Jung 2020).

**Route of infection.** Ingestion of metacercariae in infected intermediate hosts (Toledo and Esteban 2016; Chai and Jung 2020).

**Site in human host.** Small intestine (Toledo and Esteban 2016; Chai and Jung 2020).

**Notes.** Numerous nominal species are considered part of the “*E.revolutum* complex” or the “37-collar-spined *Echinostoma* complex”; taxonomic resolution of species within this group is an area of ongoing investigation.

**Genus *Hypoderaeum* Dietz, 1909**

***Hypoderaeumconoideum* (Bloch, 1782)**

**Geographic distribution.** Southeast Asia, Japan, Europe, North and Central America (Toledo and Esteban 2016; Chai and Jung 2020).

**Natural hosts.** First intermediate hosts include several genera of freshwater snails. Second intermediate hosts include freshwater snails and amphibians. Definitive hosts are birds. Zoonotic in humans (Toledo and Esteban 2016; Chai and Jung 2020).

**Route of infection.** Ingestion of metacercariae infected intermediate hosts (Toledo and Esteban 2016; Chai and Jung 2020).

**Site in human host.** Small intestine (Toledo and Esteban 2016; Chai and Jung 2020).

**Genus *Isthmiophora* Lühe, 1909**

***Isthmiophorahortensis* (Asada, 1926)**

**Geographic distribution.** China, Japan, Korea (Toledo and Esteban 2016; Chai and Jung 2020).

**Natural hosts.** First intermediate hosts include freshwater snails in the genera *Lymnaea* and *Radix*. Second intermediate hosts include several genera of freshwater fish. Definitive hosts are felids, canids, and rodents. Zoonotic in humans (Toledo and Esteban 2016; Chai and Jung 2020).

**Route of infection.** Ingestion of metacercariae in infected fish intermediate hosts (Toledo and Esteban 2016; Chai and Jung 2020).

**Site in human host.** Small intestine (Toledo and Esteban 2016; Chai and Jung 2020).

***Isthmiophoramelis* (Schrank, 1788)**

**Geographic distribution.** Europe, USA, Taiwan (Toledo and Esteban 2016; Chai and Jung 2020).

**Natural hosts.** First intermediate hosts freshwater snails in the genus *Lymnaea*. Second intermediate hosts are freshwater fish and amphibians. Definitive hosts are canids, mustelids, hedgehogs, and rodents. Zoonotic in humans (Toledo and Esteban 2016; Chai and Jung 2020).

**Route of infection.** Ingestion of metacercariae in infected intermediate hosts (Toledo and Esteban 2016; Chai and Jung 2020).

**Site in human host.** Small intestine (Toledo and Esteban 2016; Chai and Jung 2020).

●●●●●●●●● **Fasciolidae Railliet, 1895**

**Genus *Fasciola* Linnaeus, 1758**

***Fasciolagigantica* Cobbold, 1855**

**Geographic distribution.** Africa, Middle East, Southeast Asia, Japan (Tolan 2011; Phalee et al. 2015).

**Natural hosts.** First intermediate hosts are freshwater snails in the genus *Lymnaea*. Second intermediate hosts are aquatic plants. Definitive hosts are primarily sheep, also cattle, buffalo, goats, pigs. Zoonotic in humans (olan 2011; Phalee et al. 2015).

**Route of infection.** Ingestion of metacercariae on contaminated aquatic vegetation (olan 2011; Phalee et al. 2015).

**Site in human host.** Biliary ducts; ectopic migration to skin, brain (olan 2011; Phalee et al. 2015).

***Fasciolahepatica* Linnaeus, 1758**

**Geographic distribution.** Worldwide; hot spots of endemicity for human infection include the Bolivian Altiplano, Ecuador, Peru, Cuba, Portugal, Spain, Turkey, North Africa (Nile Delta), Iran, Vietnam (Tolan 2011).

**Natural hosts.** First intermediate hosts are freshwater snails, particularly members of the genera *Galba*, *Fossaria*, and *Pseudosuccinea*. Second intermediate hosts are aquatic plants. Definitive hosts include primarily cattle and buffalo, also sheep, goats, and deer. Zoonotic in humans (Tolan 2011).

**Route of infection.** Ingestion of metacercariae on contaminated aquatic vegetation (Tolan 2011).

**Site in human host.** Biliary ducts; ectopic migration to skin, brain (Tolan 2011).

**Genus *Fasciolopsis* Looss, 1899**

***Fasciolopsisbuski* (Lankester in Küchenmeister, 1857)**

*Distomarathouisi* Poirier, 1887

*Fasciolopsisfuelleborni* Rodenwadt, 1909

*Fasciolopsisgoddardi* Ward, 1910

**Geographic distribution.** Central and Southeast Asia (Sah et al. 2019; Chai and Jung 2020).

**Natural hosts.** First intermediate hosts are several genera of freshwater snails, especially the genera *Segmentina*, *Hippeutis*, and *Gyraulus*. Second intermediate hosts are freshwater plants. Definitive hosts are pigs, rabbits, and canids. Zoonotic in humans (Ma et al. 2017; Sah et al. 2019; Chai and Jung 2020).

**Route of infection.** Ingestion of metacercariae on contaminated freshwater vegetation (Sah et al. 2019; Chai and Jung 2020).

**Site in human host.** Small intestine (Sah et al. 2019; Chai and Jung 2020).

●●●●●●●●● **Philophthalmidae Looss, 1899**

**Genus *Philophthalmus* Looss, 1899**

***Philophthalmusgralli* Matthis & Leger, 1910**

*Philophthalmusanatinus* Sugimoto, 1928

*Philophthalmusnyrocae* Yamaguti, 1934

**Geographic distribution.** East and Southeast Asia, North America, South America (Nollen and Kanev 1995).

**Natural hosts.** Intermediate hosts are snails in the genus *Melanoides*. Definitive hosts are birds, primarily water birds and fowl. Zoonotic in humans (Nollen and Kanev 1995).

**Route of infection.** Ingestion of metacercariae in water or on submerged substrates (e.g., mollusk shells, plants); also direct exposure of eyes to free-swimming cercariae in water (Nollen and Kanev 1995).

**Site in human host.** Eyes (Nollen and Kanev 1995).

***Philophthalmuspalpebrarum* Looss, 1899**

**Geographic distribution.** Middle East, North Africa (Nollen and Kanev 1995).

**Natural hosts.** Intermediate hosts are snails in the genus *Melanoides*. Definitive hosts are birds, primarily water birds and fowl. Zoonotic in humans (Nollen and Kanev 1995).

**Route of infection.** Ingestion of metacercariae in water or on submerged substrates (e.g., mollusk shells, plants); also direct exposure of eyes to free-swimming cercariae in water (Nollen and Kanev 1995).

**Site in human host.** Eyes (Nollen and Kanev 1995).

***Philophthalmuslacrymosus* Braun, 1902**

**Geographic distribution.** Central and South America (Nollen and Kanev 1995).

**Natural hosts.** Intermediate hosts are snails in the genus *Melanoides*. Definitive hosts are birds, primarily water birds and fowl. Zoonotic in humans (Nollen and Kanev 1995).

**Route of infection.** Ingestion of metacercariae in water or on submerged substrates (e.g., mollusk shells, plants); also direct exposure of eyes to free-swimming cercariae in water (Nollen and Kanev 1995).

**Site in human host.** Eyes (Nollen and Kanev 1995).

●●●●●●●● **Hemiurata Skrjabin & Guschanskaja, 1954**

●●●●●●●●● **Isoparorchiidae Travassos, 1922**

**Genus *Isoparorchis* Southwell, 1913**

*Leptolecithum* Kobayashi, 1915

***Isoparorchishypselobagri* (Billet, 1898)**

**Geographic distribution.** Central and Southeast Asia, Russia, Australia (Chai and Jung 2020).

**Natural hosts.** First intermediate hosts are freshwater snails in the genus *Melanoides*. Second intermediate hosts are catfish. Definitive hosts are predator fish. Zoonotic in humans (Chai and Jung 2020).

**Route of infection.** Ingestion of metacercariae in infected fish intermediate hosts (Chai and Jung 2020).

**Site in human host.** Small intestine (Chai and Jung 2020).

●●●●●●●● **Opisthorchiata La Rue, 1957**

●●●●●●●●● **Heterophyidae Leiper, 1909**

**Genus *Acanthotrema* Travassos, 1928**

***Acanthotremafelis* Sohn et al., 2003**

**Geographic distribution.** Korea (Chai and Jung 2020).

**Natural hosts.** First intermediate hosts are unknown. Second intermediate hosts are brackish water fish in the genus *Acanthogobius*. Definitive hosts are felids. Zoonotic in humans (Chai and Jung 2020).

**Route of infection.** Ingestion of metacercariae in infected fish intermediate host (Chai and Jung 2020).

**Site in human host.** Small intestine (Chai and Jung 2020).

**Genus *Apophallus* Lühe, 1909**

***Apophallusdonicus* (Skrjabin & Lindtrop, 1919)**

**Geographic distribution.** North America (Chai and Jung 2020).

**Natural hosts.** First intermediate hosts are freshwater snails in the genus *Flumenicola*. Second intermediate hosts are various freshwater fish. Definitive hosts are carnivores and lagomorphs. Zoonotic in humans (Chai and Jung 2020).

**Route of infection.** Ingestion of metacercariae in infected fish intermediate host (Chai and Jung 2020).

**Site in human host.** Small intestine (Chai and Jung 2020).

**Genus *Ascocotyle* Looss, 1899**

***Ascocotylelonga* Ransom, 1920**

**Geographic distribution.** North America, Europe (Chai and Jung 2020).

**Natural hosts.** First intermediate hosts are brackish water snails in the genus *Helecobia*. Second intermediate hosts are brackish water fish in the genus *Mugil*. Definitive hosts are canids. Zoonotic in humans (Chai and Jung 2020).

**Route of infection.** Ingestion of metacercariae in infected fish (Chai and Jung 2020).

**Site in human host.** Small intestine (Chai and Jung 2020).

**Genus *Centrocestus* Looss, 1899**

***Centrocestusarmatus* (Tanabe, 1922)**

**Geographic distribution.** Korea, Japan (Chai and Jung 2020).

**Natural hosts.** First intermediate hosts are freshwater snails in the genus *Semisulcospira*. Second intermediate hosts are freshwater fish in the genus *Zacco*. Definitive hosts are wild and domestic canids and felids, rabbits, rats, and birds. Zoonotic in humans (Chai and Jung 2020).

**Route of infection.** Ingestion of metacercariae in infected fish (Chai and Jung 2020).

**Site in human host.** Small intestine (Chai and Jung 2020).

***Centrocestuscuspidatus* (Looss, 1896)**

**Geographic distribution.** Southeast Asia, North Africa, Middle East (Chai and Jung 2020).

**Natural hosts.** First intermediate hosts are freshwater snails in the genus *Oncomelania*. Second intermediate hosts are freshwater fish in the genus *Gambusia*. Definitive hosts are carnivores, rodents, and birds. Zoonotic in humans (Chai and Jung 2020).

**Route of infection.** Ingestion of metacercariae in infected fish (Chai and Jung 2020).

**Site in human host.** Small intestine (Chai and Jung 2020).

***Centrocestusformosanus* Nishigori, 1924**

*Centrocestuscaninus* Leiper, 1913

**Geographic distribution.** Southeast Asia, Central and South America, North Africa, Middle East (Chai and Jung 2020).

**Natural hosts.** First intermediate hosts are freshwater snails in the genus *Stenomelania*. Second intermediate hosts are freshwater fish, including *Cyclocheilichthys* and *Puntius*. Definitive hosts are wild and domestic canids, chickens, and ducks. Zoonotic in humans (Chai and Jung 2020).

**Route of infection.** Ingestion of metacercariae in infected fish (Chai and Jung 2020).

**Site in human host.** Small intestine (Chai and Jung 2020).

***Centrocestuskurokawai* (Kurowawa, 1935)**

**Geographic distribution.** Japan (Chai and Jung 2020).

**Natural hosts.** First intermediate hosts are unknown. Second intermediate hosts are unknown but presumed to be freshwater fish. Definitive hosts unknown; presumed zoonotic in humans (Chai and Jung 2020).

**Route of infection.** Ingestion of metacercariae in infected intermediate host (Chai and Jung 2020).

**Site in human host.** Small intestine (Chai and Jung 2020).

**Genus *Cryptocotyle* Lühe, 1899**

***Cryptocotylelingua* (Creplin, 1825)**

**Geographic distribution.** North America, Europe, Russia, Japan (Chai and Jung 2020).

**Natural hosts.** First intermediate hosts are brackish water snails in the genus *Littorina*. Second intermediate hosts are brackish and freshwater fish in the genus *Gobius*. Definitive hosts are carnivores, rodents, and birds. Zoonotic in humans (Chai and Jung 2020).

**Route of infection.** Ingestion of metacercariae in infected fish (Chai and Jung 2020).

**Site in human host.** Small intestine (Chai and Jung 2020).

**Genus *Haplorchis* Looss, 1899**

***Haplorchispumilio* (Looss, 1896)**

**Geographic distribution.** Central and Southeast Asia, Australia, Middle East, North Africa, Central and South America (Chai and Jung 2020).

**Natural hosts.** First intermediate hosts are snails in the genus *Melania*. Second intermediate hosts are various freshwater fish. Definitive hosts are carnivores. Zoonotic in humans (Chai and Jung 2020).

**Route of infection.** Ingestion of metacercariae in infected freshwater fish (Chai and Jung 2020).

**Site in human host.** Small intestine (Chai and Jung 2020).

***Haplorchistaichui* (Nishigori, 1924)**

*Monorchotremamicrorchia* Katsuta, 1932

**Geographic distribution.** Central and Southeast Asia, Hawaii, Middle East (Chai and Jung 2020).

**Natural hosts.** First intermediate hosts are freshwater snails in the genera *Melania* and *Melanoides*. Second intermediate hosts are various freshwater fish. Definitive hosts are carnivores and birds. Zoonotic in humans (Chai and Jung 2020).

**Route of infection.** Ingestion of metacercariae in infected freshwater fish (Chai and Jung 2020).

**Site in human host.** Small intestine (Chai and Jung 2020).

***Haplorchisvanissimus* Africa, 1938**

**Geographic distribution.** Australia, Philippines (Chai and Jung 2020).

**Natural hosts.** First intermediate hosts are unknown. Second intermediate hosts are unknown but presumed to be freshwater fish. Definitive hosts are wild and domestic canids and felids and birds. Zoonotic in humans (Chai and Jung 2020).

**Route of infection.** Ingestion of metacercariae in infected fish (Chai and Jung 2020).

**Site in human host.** Small intestine (Chai and Jung 2020).

***Haplorchisyokogawai* (Katsuta, 1932)**

**Geographic distribution.** Central and Southeast Asia, Australia, Middle East, North Africa (Chai and Jung 2020).

**Natural hosts.** First intermediate hosts include several genera of freshwater snails. Second intermediate hosts are various freshwater fish. Definitive hosts are mammals, including cattle and carnivores. Zoonotic in humans (Chai and Jung 2020).

**Route of infection.** Ingestion of metacercariae in infected freshwater fish (Chai and Jung 2020).

**Site in human host.** Small intestine (Chai and Jung 2020).

**Genus *Heterophyes* Cobbold, 1866**

***Heterophyesheterophyes* (Siebold, 1853)**

*Heterophyesaegyptiaca* Cobbold, 1866

**Geographic distribution.** Central and Southeast Asia, Japan, Middle East, North Africa (Chai and Jung 2020).

**Natural hosts.** First intermediate hosts are various freshwater snails. Second intermediate hosts are various freshwater and brackish water fish. Definitive hosts are carnivores. Zoonotic in humans (Chai and Jung 2020).

**Route of infection.** Ingestion of metacercariae in infected fish (Chai and Jung 2020).

**Site in human host.** Small intestine (Chai and Jung 2020).

***Heterophyesnocens* Onji & Nishio, 1916**

*Heterophyeskatsuradai* Ozaki & Asada, 1926

**Geographic distribution.** Korea, Japan China (Chai and Jung 2020).

**Natural hosts.** First intermediate hosts are freshwater snails in the genus *Cerithidea*. Second intermediate hosts are freshwater fish in the genus *Acanthogobius*. Definitive hosts are wild and domestic and felids. Zoonotic in humans (Chai and Jung 2020).

**Route of infection.** Ingestion of metacercariae in infected fish (Chai and Jung 2020).

**Site in human host.** Small intestine (Chai and Jung 2020).

**Genus *Heterophyopsis* Tubangui & Africa, 1938**

***Heterophyopsiscontinua* (Onki & Nishio, 1916)**

**Geographic distribution.** Korea, Japan (Chai and Jung 2020).

**Natural hosts.** First intermediate hosts are unknown. Second intermediate hosts are various freshwater fish. Definitive hosts are carnivores and birds. Zoonotic in humans(Chai and Jung 2020).

**Route of infection.** Ingestion of metacercariae in infected fish (Chai and Jung 2020).

**Site in human host.** Small intestine (Chai and Jung 2020).

**Genus *Metagonimus* Katsurada, 1912**

***Metagonimuskatsuradai* Izumi, 1935**

**Geographic distribution.** Japan, Russia (Chai and Jung 2020).

**Natural hosts.** First intermediate hosts are freshwater snails in the genus *Semisulcospira*. Second intermediate hosts are freshwater fish in the genus *Tanakia*. Definitive hosts are carnivores. Zoonotic in humans (Chai and Jung 2020).

**Route of infection.** Ingestion of metacercariae in infected fish (Chai and Jung 2020).

**Site in human host.** Small intestine (Chai and Jung 2020).

***Metagonimusminutus* Katsuta, 1932**

**Geographic distribution.** Taiwan (Chai and Jung 2020).

**Natural hosts.** First intermediate hosts are unknown. Second intermediate hosts are brackish water fish in the genus *Mugil*. Definitive hosts are unknown; presumed zoonotic in humans (Chai and Jung 2020).

**Route of infection.** Ingestion of metacercariae in infected fish (Chai and Jung 2020).

**Site in human host.** Small intestine (Chai and Jung 2020).

***Metagonimusmiyatai* Saito et al., 1997**

**Geographic distribution.** Korea, Japan (Chai and Jung 2020).

**Natural hosts.** First intermediate hosts are freshwater snails in the genus *Semisulcospira*. Second intermediate hosts are various freshwater fish. Definitive hosts are carnivores, rodents, and birds. Zoonotic in humans (Chai and Jung 2020).

**Route of infection.** Ingestion of metacercariae in infected fish (Chai and Jung 2020).

**Site in human host.** Small intestine (Chai and Jung 2020).

***Metagonimustakahashii* Takahashi, 1929**

**Geographic distribution.** Korea, Japan (Chai and Jung 2020).

**Natural hosts.** First intermediate hosts are freshwater snails in the genera *Semisulcospira* and *Koreanomelania*. Second intermediate hosts are various freshwater fish. Definitive hosts are carnivores and birds. Zoonotic in humans (Chai and Jung 2020).

**Route of infection.** Ingestion of metacercariae in infected fish (Chai and Jung 2020).

**Site in human host.** Small intestine (Chai and Jung 2020).

***Metagonimusyokogawai* (Katsurada, 1912)**

**Geographic distribution.** Central and Southeast Asia, Russia, Japan, Europe (Chai and Jung 2020).

**Natural hosts.** First intermediate hosts are freshwater snails in the genus *Semisulcospira*. Second intermediate hosts are various freshwater fish. Definitive hosts are carnivores, rodents, and birds. Zoonotic in humans (Chai and Jung 2020).

**Route of infection.** Ingestion of metacercariae in infected fish (Chai and Jung 2020).

**Site in human host.** Small intestine (Chai and Jung 2020).

**Genus *Procerovum* Onji & Nishio, 1916**

***Procerovumcalderoni* (Africa & Garcia, 1935)**

**Geographic distribution.** Egypt, Southeast Asia (Chai and Jung 2020).

**Natural hosts.** First intermediate hosts are brackish water snails in the genus *Sermyla*. Second intermediate hosts are freshwater fish in the genus *Ophiocephalis*. Definitive hosts are wild and domestic canids and felids. Zoonotic in humans (Chai and Jung 2020).

**Route of infection.** Ingestion of metacercariae in infected fish (Chai and Jung 2020).

**Site in human host.** Small intestine (Chai and Jung 2020).

***Procerovumvarium* Onji & Nishio, 1916**

**Geographic distribution.** Korea, Japan (Chai and Jung 2020).

**Natural hosts.** First intermediate hosts are brackish water snails in the genus *Melanoides*. Second intermediate hosts are brackish water fish in the genus *Mugil*. Definitive hosts are wild and domestic canids and felids. Zoonotic in humans (Chai and Jung 2020).

**Route of infection.** Ingestion of metacercariae in infected fish (Chai and Jung 2020).

**Site in human host.** Small intestine (Chai and Jung 2020).

**Genus *Pygidiopsis* Looss, 1907**

***Pygidiopsisgenata* Looss, 1907**

**Geographic distribution.** North Africa, Middle East, Eastern Europe, Philippines (Chai and Jung 2020).

**Natural hosts.** First intermediate hosts are freshwater snails in the genera *Melanoides* and *Melanopsis*. Second intermediate hosts are various freshwater fish. Definitive hosts are carnivores, rodents, shrews, and birds. Zoonotic in humans (Chai and Jung 2020).

**Route of infection.** Ingestion of metacercariae in infected fish (Chai and Jung 2020).

**Site in human host.** Small intestine (Chai and Jung 2020).

***Pygidiopsissumma* Onji & Nishio, 1916**

**Geographic distribution.** Korea, Japan, Vietnam (Chai and Jung 2020).

**Natural hosts.** First intermediate hosts are unknown. Second intermediate hosts are brackish water fish in the genera *Mugil* and *Acnathogonius*. Definitive hosts are wild and domestic felids. Zoonotic in humans (Chai and Jung 2020).

**Route of infection.** Ingestion of metacercariae in infected fish (Chai and Jung 2020).

**Site in human host.** Small intestine (Chai and Jung 2020).

**Genus *Stellantchasmus* Onji & Nishio, 1916**

***Stellantchasmusfalcatus* Onji & Nishio, 1916**

**Geographic distribution.** Central and Southeast Asia, Japan, Middle East, Australia, Hawaii (Chai and Jung 2020).

**Natural hosts.** First intermediate hosts are freshwater snails in the genera *Stenomelania* and *Thiara*. Second intermediate hosts are mullets. Definitive hosts are wild and domestic canids and felids, rabbits, rats, and birds. Zoonotic in humans (Chai and Jung 2020).

**Route of infection.** Ingestion of metacercariae in infected fish (Chai and Jung 2020).

**Site in human host.** Small intestine (Chai and Jung 2020).

**Genus *Stictodora* Looss, 1899**

***Stictodorafuscata* (Onji & Nishio, 1916)**

**Geographic distribution.** Korea, Japan, Kuwait (Chai and Jung 2020).

**Natural hosts.** First intermediate hosts are unknown. Second intermediate hosts are brackish water fish in the genera *Acanthogobius* and *Pseudorasbora*. Definitive hosts are wild and domestic canids and felids. Zoonotic in humans (Chai and Jung 2020).

**Route of infection.** Ingestion of metacercariae in infected fish (Chai and Jung 2020).

**Site in human host.** Small intestine (Chai and Jung 2020).

***Stictodoralari* Yamaguti, 1939**

**Geographic distribution.** Southeast Asia, Japan, Russia, Australia (Chai and Jung 2020).

**Natural hosts.** First intermediate hosts are brackish water snails, including the genus *Velacumantus*. Second intermediate hosts are various brackish water fish. Definitive hosts are wild and domestic canids and felids. Zoonotic in humans (Chai and Jung 2020).

**Route of infection.** Ingestion of metacercariae in infected fish (Chai and Jung 2020).

**Site in human host.** Small intestine (Chai and Jung 2020).

●●●●●●●●● **Opisthorchiidae Looss, 1899**

**Genus *Amphimerus* Barker, 1911**

**Geographic distribution.** North, Central, and South America (Calvopiña et al. 2011, 2015).

**Natural hosts.** Intermediate hosts are various freshwater snails. Second intermediate hosts are freshwater fish. Natural hosts are a variety of mammals, birds, and reptiles, including domestic dogs and cats. Zoonotic in humans (Calvopiña et al. 2011, 2015).

**Route of infection.** Ingestion of metacercariae in infected fish (Calvopiña et al. 2011).

**Site in human host.** Liver (Calvopiña et al. 2011).

**Notes.** The natural hosts and species identification have not been confirmed from human isolates, most of which have been documented in Ecuador.

**Genus *Clonorchis* Looss, 1907**

***Clonorchissinensis* (Cobbold, 1875)**

**Geographic distribution.** East Asia; hot spots of endemicity for human disease include Korea, China, Taiwan, and Vietnam (Qian et al. 2012).

**Natural hosts.** First intermediate hosts are various genera of freshwater snails. Second intermediate host are a variety of freshwater fish, primarily cyprinids. Definitive hosts are fish-eating mammals, including wild and domestic canids and felids, mustelids, and pigs. Zoonotic in humans (Qian et al. 2012).

**Route of infection.** Ingestion of metacercariae in infected fish (Qian et al. 2012).

**Site in human host.** Biliary ducts (Qian et al. 2012).

**Genus *Metorchis* Looss, 1899**

***Metorchisbilis* (Braun, 1790)**

*Distomaalbidus* Braun, 1893

*Distomacrassiusculus* Rudolphi, 1809

**Geographic distribution.** Central and Eastern Europe, Russia (Mordvinov et al. 2012).

**Natural hosts.** First intermediate hosts include snails of the genus *Bithynia*. Second intermediate hosts are typically cyprinid fishes (e.g., carps, minnows). Natural definitive hosts are various fish-eating mammals and birds. Zoonotic in humans (Mordvinov et al. 2012).

**Route of infection.** Ingestion of metacercariae in infected fish (Mordvinov et al. 2012).

**Site in human host.** Biliary ducts (Mordvinov et al. 2012).

***Metorchisconjunctus* (Cobbold, 1860)**

**Geographic distribution.** Northern North America (Behr et al. 1998).

**Natural hosts.** First intermediate hosts are freshwater snails in the genus *Amnicola*. Second intermediate hosts are freshwater fish, primarily members of the genus *Catostomus*. Definitive hosts are carnivores. Zoonotic in humans (Behr et al. 1998).

**Route of infection.** Ingestion of metacercariae in infected fish (MacLean et al. 1996; Behr et al. 1998).

**Site in human host.** Biliary ducts (MacLean et al. 1996; Behr et al. 1998).

***Metorchisorientalis* Tanabe, 1921**

**Geographic distribution.** East Asia (Na et al. 2016).

**Natural hosts.** First intermediate hosts include snails of the genera *Bithynia* and *Parafossarulus*. Second intermediate hosts are typically cyprinid fishes (e.g., carps, minnows). Natural definitive hosts are various fish-eating mammals and birds. Zoonotic in humans (Na et al. 2016).

**Route of infection.** Ingestion of metacercariae in infected fish (Na et al. 2016).

**Site in human host.** Biliary ducts (Na et al. 2016).

***Metorchistaiwanensis* Morishita & Tsuchimochi, 1952**

**Geographic distribution.** East Asia (Zhan et al. 2017).

**Natural hosts.** First intermediate hosts include snails of the genus *Bithynia*. Second intermediate hosts are typically cyprinid fishes (e.g., carps, minnows). Natural definitive hosts are various fish-eating mammals and birds. Zoonotic in humans (Zhan et al. 2017).

**Route of infection.** Ingestion of metacercariae in infected fish (Zhan et al. 2017).

**Site in human host.** Biliary ducts (Zhan et al. 2017).

**Genus *Opisthorchis* Blanchard, 1895**

***Opisthorchisfelineus* (Rivolta, 1884)**

**Geographic distribution.** Eastern Europe, Russia, Central Asia (Pakharukova and Mordvinov 2016).

**Natural hosts.** First intermediate hosts are freshwater snails in the genus *Bithynia*. Second intermediate hosts are various freshwater fish. Definitive hosts are mammals, primarily carnivores. Zoonotic in humans (Pakharukova and Mordvinov 2016).

**Route of infection.** Ingestion of metacercariae in infected fish (Pakharukova and Mordvinov 2016).

**Site in human host.** Biliary ducts (Pakharukova and Mordvinov 2016).

***Opisthorchisviverrini* (Poirier, 1886)**

**Geographic distribution.** Southeast Asia, with high prevalence of human infection in Thailand, Laos, Vietnam, and Cambodia (Kaewpitoon et al. 2008; Suwannatrai et al. 2018).

**Natural hosts.** First intermediate hosts are freshwater snails in the genus *Bithynia*. Second intermediate hosts are various freshwater fish. Definitive hosts are wild and domestic canids and felids. Zoonotic in humans (Kaewpitoon et al. 2008; Suwannatrai et al. 2018).

**Route of infection.** Ingestion of metacercariae in infected fish (Kaewpitoon et al. 2008).

**Site in human host.** Biliary ducts (Kaewpitoon et al. 2008).

**Genus *Pseudamphistomum* Lühe, 1908**

***Pseudamphistomumtruncatum* (Rudolphi, 1819)**

**Geographic distribution.** Europe, Asia (Neimanis et al. 2016).

**Natural hosts.** First intermediate hosts are freshwater and brackish snails. Second intermediate host are freshwater, brackish, and marine fish, primarily cyprinids. Definitive hosts are carnivores, including cats, pinnipeds, and mustelids. Zoonotic in humans (Neimanis et al. 2016).

**Route of infection.** Ingestion of metacercariae in infected fish (Neimanis et al. 2016).

**Site in human host.** Biliary ducts (Neimanis et al. 2016).

●●●●●●●● **Pronocephalata Olson et al., 2003**

●●●●●●●●● **Paramphistomidae Fischoeder, 1901**

**Genus *Fischoederius* Stiles & Goldberger, 1910**

***Fischoederiuselongatus* (Poirier, 1883)**

**Geographic distribution.** Europe, Russia, Central and Southeast Asia, Japan (Chai and Jung 2020).

**Natural hosts.** First intermediate hosts are freshwater snails in the genus *Lymnaea*. Second intermediate hosts are freshwater plants. Definitive hosts are cattle, deer, goats, and sheep. Zoonotic in humans (Chai and Jung 2020).

**Route of infection.** Ingestion of metacercariae on contaminated aquatic vegetation (Chai and Jung 2020)

**Site in human host.** Small intestine (Chai and Jung 2020).

**Genus *Gastrodiscoides* Leiper, 1913**

***Gastrodiscoideshominis* (Lewis & McConnell, 1876)**

**Geographic distribution.** Europe, Central and Southeast Asia(Mas-Coma 2006; Chai and Jung 2020).

**Natural hosts.** First intermediate hosts are freshwater snails in the genus *Helicorbis*. Second intermediate hosts are freshwater plants, amphibians, and crayfish. Definitive hosts are pigs and humans (Mas-Coma 2006; Chai and Jung 2020).

**Route of infection.** Ingestion of metacercariae in infected intermediate hosts (Mas-Coma 2006; Chai and Jung 2020).

**Site in human host.** Small intestine (Mas-Coma 2006; Chai and Jung 2020).

**Genus *Watsonius* Stiles & Goldberger, 1910**

***Watsoniuswatsoni* (Conyngham, 1904)**

**Geographic distribution.** Sub-Saharan Africa, Vietnam (Chai and Jung 2020).

**Natural hosts.** First intermediate hosts are freshwater snails in the genus *Physa*. Second intermediate hosts are presumed to be freshwater vegetation. Definitive hosts are non-human primates. Zoonotic in humans (Chai and Jung 2020).

**Route of infection.** Ingestion of metacercariae on contaminated aquatic vegetation (Chai and Jung 2020).

**Site in human host.** Small intestine (Chai and Jung 2020).

●●●●●●●● **Xiphidiata Olson et al., 2003**

●●●●●●●●● **Dicrocoeliidae Looss, 1899**

**Genus *Dicrocoelium* Dujardin, 1845**

***Dicrocoeliumdendriticum* (Rudolphi, 1819)**

*Dicrocoeliumlanceolatum* Stiles & Hassall, 1898

**Geographic distribution.** North America, Africa, Asia, Europe, Middle East (Cengiz et al. 2010).

**Natural hosts.** First intermediate hosts are various genera of terrestrial snails. Second intermediate hosts are ants (primarily genus *Formica*). Definitive hosts are ungulates, including cattle, sheep, and goats. Zoonotic in humans (Cengiz et al. 2010).

**Route of infection.** Ingestion of metacercariae in infected ants (Cengiz et al. 2010).

**Site in human host.** Bile ducts, gall bladder (Cengiz et al. 2010).

***Dicrocoeliumhospes* Looss, 1907**

**Geographic distribution.** West Africa (Odei 1966).

**Natural hosts.** First intermediate hosts are snails in the genera *Achatina* and *Limicolaria*. Second intermediate hosts are ants in the genera *Dorylus* and *Crematogaster*. Definitive hosts are ungulates, including cattle and sheep. Zoonotic in humans (Odei 1966).

**Route of infection.** Ingestion of infected intermediate hosts (Odei 1966).

**Site in human host.** Bile ducts (Odei 1966)

**Notes.** It is uncertain whether *D.hospes* causes true infection in humans. Most reported cases of the finding of eggs in stool are believed to be spurious after the consumption of infected beef liver (Wolfe 2007). The two cases from Ghana believed to be true infections are in children who denied eating beef liver (Odei 1966). True infection should be confirmed by the examination of follow-up stool specimens (Wolfe 2007).

●●●●●●●●● **Lecithodendriidae Lühe, 1901**

**Genus *Caprimolgorchis* Jha, 1943**

***Caprimolgorchismolenkampi* Lie Kian Joe, 1961**

**Geographic distribution.** Southeast Asia (Chai and Jung 2020).

**Natural hosts.** First intermediate hosts are unknown, but hypothesized to be freshwater snails in the genera *Bithynia* or *Zebrina*. Second intermediate hosts are dragonflies and damselflies. Definitive hosts are rodents and bats. Zoonotic in humans (Chai and Jung 2020).

**Route of infection.** Ingestion of infected insects (Chai and Jung 2020).

**Site in human host.** Small intestine (Chai and Jung 2020).

●●●●●●●●● **Microphalidae Ward, 1901**

**Genus *Gynaecotyla* Yamaguti, 1939**

***Gynaecotylasquatarolae* (Yamaguti, 1934)**

**Geographic distribution.** Japan, South Korea, Taiwan (Chai and Jung 2020).

**Natural hosts.** First intermediate hosts are brackish water snails in the genus *Batillaria*. Second intermediate hosts are brackish water crabs in the genus *Macrophthalmus*. Definitive hosts are shorebirds. Zoonotic in humans (Chai and Jung 2020).

**Route of infection.** Ingestion of metacercariae in infected crabs (Chai and Jung 2020).

**Site in human host.** Small intestine (Chai and Jung 2020).

**Genus *Microphallus* Ward, 1901**

***Microphallusbrevicaeca* (Africa & Garcia, 1935)**

**Geographic distribution.** Papua New Guinea, Philippines (Chai and Jung 2020).

**Natural hosts.** First intermediate hosts are unknown. Second intermediate hosts are brackish water crustaceans in the genera *Carcinus* and *Macrobrachium*. Definitive hosts are birds and mammals, especially non-human primates. Zoonotic in humans (Chai and Jung 2020).

**Route of infection.** Ingestion of metacercariae in infected crustaceans (Chai and Jung 2020).

**Site in human host.** Small intestine (Chai and Jung 2020).

●●●●●●●●● **Phaeneropsolidae Mehra, 1935**

**Genus *Phaneropsolus* Looss, 1899**

***Phaneropsolusbonnei* Lie Kian Joe, 1951**

**Geographic distribution.** Central and Southeast Asia (Chai and Jung 2020).

**Natural hosts.** First intermediate hosts are unknown, but hypothesized to be freshwater snails in the genus *Bithynia*. Second intermediate hosts are dragonflies and damselflies. Definitive hosts are non-human primates. Zoonotic in humans (Chai and Jung 2020).

**Route of infection.** Ingestion of infected insects (Chai and Jung 2020).

**Site in human host.** Small intestine (Chai and Jung 2020).

***Phaneropsolusspinicirrus* Kaewkes et al., 1991**

**Geographic distribution.** Thailand (Chai and Jung 2020).

**Natural hosts.** First intermediate hosts are unknown. Second intermediate hosts are unknown, but presumed to be dragonflies and damselflies. Definitive hosts are unknown; presumed zoonotic in humans (Chai and Jung 2020).

**Route of infection.** Ingestion of infected intermediate hosts (Chai and Jung 2020).

**Site in human host.** Small intestine (Chai and Jung 2020).

●●●●●●●●● **Plagiorchiidae Lühe, 1901**

**Genus *Plagiorchis* Lühe, 1899**

***Plagiorchisjavensis* Sandground, 1940**

**Geographic distribution.** Indonesia (Chai and Jung 2020).

**Natural hosts.** First intermediate hosts are unknown. Second intermediate hosts include snails, insects, and possibly fish. Definitive hosts are birds and bats. Zoonotic in humans (Chai and Jung 2020).

**Route of infection.** Ingestion of metacercariae in infected intermediate hosts (Chai and Jung 2020).

**Site in human host.** Small intestine (Chai and Jung 2020).

***Plagiorchismuris* (Tanabe, 1922)**

**Geographic distribution.** Europe, Central and Southeast Asia, Japan, North and Central America (Chai and Jung 2020).

**Natural hosts.** First intermediate hosts are freshwater snails in the genus *Lymnaea*. Second intermediate hosts are freshwater insects, crustaceans, and fish. Definitive hosts are canids, felids, raccoons, rodents, bats, and birds. Zoonotic in humans (Chai and Jung 2020).

**Route of infection.** Ingestion of metacercariae in infected intermediate hosts (Chai and Jung 2020).

**Site in human host.** Small intestine (Chai and Jung 2020).

***Plagiorchisphilippinensis* Africa & Garcia, 1937**

**Geographic distribution.** Philippines (Chai and Jung 2020).

**Natural hosts.** First intermediate hosts are unknown. Second intermediate hosts are presumed to be aquatic insects. Definitive hosts rodents and possibly birds. Zoonotic in humans (Chai and Jung 2020).

**Route of infection.** Ingestion of metacercariae in infected intermediate hosts (Chai and Jung 2020).

**Site in human host.** Small intestine (Chai and Jung 2020).

***Plagiorchisvespertilionis* (Müller, 1784)**

**Geographic distribution.** Europe, North Africa, Central and Southeast Asia, Japan, Madagascar, North America (Chai and Jung 2020).

**Natural hosts.** First intermediate hosts are freshwater snails in the genus *Lymnaea*. Second intermediate hosts are freshwater insects. Definitive hosts are rodents and bats. Zoonotic in humans (Chai and Jung 2020).

**Route of infection.** Ingestion of metacercariae in infected insect intermediate hosts (Chai and Jung 2020).

**Site in human host.** Small intestine (Chai and Jung 2020).

●●●●●●●● **Troglotremata Schell, 1890**

●●●●●●●●● **Nanophyetidae Dollfus, 1939**

**Genus *Nanophyetus* Chapin, 1928**

***Nanophyetussalmincola* (Chapin, 1926)**

**Geographic distribution.** Northern North America (Chai and Jung 2020).

**Natural hosts.** First intermediate hosts are snails in the genus *Oxytrema*. Second intermediate hosts are several general of freshwater fish, especially salmonids. Definitive hosts canids, felids, raccoons, and birds. Zoonotic in humans (Chai and Jung 2020).

**Route of infection.** Ingestion of metacercariae in infected intermediate hosts (Chai and Jung 2020).

**Site in human host.** Small intestine (Chai and Jung 2020).

***Nanophyetusschikhobalowi* Skrjabin & Podiapolskaia, 1931**

**Geographic distribution.** Northern Eurasia (Voronova et al. 2017; Voronova and Chelomina 2018).

**Natural hosts.** First intermediate hosts are snails in the genus *Oxytrema*. Second intermediate hosts are several general of freshwater fish, especially salmonids. Definitive hosts canids, felids, raccoons, and birds. Zoonotic in humans (Voronova et al. 2017; Voronova and Chelomina 2018).

**Route of infection.** Ingestion of metacercariae in infected intermediate hosts (Voronova et al. 2017; Voronova and Chelomina 2018).

**Site in human host.** Small intestine (Voronova et al. 2017; Voronova and Chelomina 2018).

●●●●●●●●● **Paragonimidae Dollfus, 1939**

**Genus *Paragonimus* Braun, 1899**

***Paragonimusafricanus* Volker & Vogel, 1965**

**Geographic distribution.** West Africa (Blair et al. 1999; Cumberlidge et al. 2018).

**Natural hosts.** First intermediate hosts are presumed to be freshwater or brackish snails, but the specific species have yet to be identified. Second intermediate hosts are freshwater crabs in the family Potamidae. Definitive hosts are carnivores and non-human primates. Zoonotic in humans (Blair et al. 1999; Cumberlidge et al. 2018).

**Route of infection.** Ingestion of metacercariae in infected intermediate or paratenic hosts (Blair et al. 1999; Cumberlidge et al. 2018).

**Site in human host.** Lungs, possibly ectopic infection at other sites (Blair et al. 1999; Cumberlidge et al. 2018).

***Paragonimusheterotremus* Chen & Hsia, 1964**

*Paragonimustuanshansis* Chung et al., 1964

*Paragonimuspseudoheterotremus* Waikagul et al., 2007

**Geographic distribution.** Southeast Asia (Yoshida et al. 2019).

**Natural hosts.** First intermediate hosts are freshwater snails in the superfamily Rissooidea. Second intermediate hosts are freshwater crabs in the families Potamidae and Parathelphusidae. Definitive hosts are carnivores and non-human primates. Wild boar, rodents, and deer can serve as paratenic hosts. Zoonotic in humans (Yoshida et al. 2019).

**Route of infection.** Ingestion of metacercariae in infected intermediate or paratenic hosts (Yoshida et al. 2019).

**Site in human host.** Lungs, possibly ectopic infection at other sites (Yoshida et al. 2019).

**Notes.***Paragonimusheterotremus* is often referred to as a species complex; the status of many members as distinct and valid species is an area of ongoing investigation.

***Paragonimuskellicotti* Ward, 1908**

**Geographic distribution.** Eastern and Midwestern North America (Procop 2009).

**Natural hosts.** First intermediate hosts are freshwater snails in the family Pomatiopsidae. Second intermediate hosts are freshwater crayfish, primarily of the family Astacidae. Definitive hosts are carnivores and marsupials. Zoonotic in humans (Procop 2009).

**Route of infection.** Ingestion of metacercariae in infected intermediate and paratenic hosts (Procop 2009).

**Site in human host.** Lungs, possibly ectopic infection at other sites (Procop 2009).

***Paragonimusmexicanus* Miyazaki & Ishii, 1968**

*Paragonimusperuvianus* Miyazaki et al., 1969

*Paragonimusecuadorensis* Voekler & Arzube, 1979

**Geographic distribution.** Central and South America (Blair et al. 1999).

**Natural hosts.** First intermediate hosts are freshwater and brackish snails in the families Hydrobiidae and Pomatiopsidae. Second intermediate hosts are freshwater crabs in the families Pseudothelphusidae and Trichodactylidae. Definitive hosts are carnivores and marsupials. Zoonotic in humans (Blair et al. 1999).

**Route of infection.** Ingestion of metacercariae in infected crustaceans (Blair et al. 1999).

**Site in human host.** Lungs, possibly ectopic infection at other sites (Blair et al. 1999).

***Paragonimusohirai* Miyazaki, 1939**

*Paragonimusiloktsuensis* Chen, 1940

*Paragonimussadoensis* Miyazaki et al., 1968

**Geographic distribution.** East Asia (Blair et al. 1999; Yoshida et al. 2019).

**Natural hosts.** First intermediate hosts are freshwater and brackish snails in the families Assimineidae and Pomatiopsidae. Second intermediate hosts are freshwater crabs in the family Potamidae and brackish crabs in the family Grapsidae. Definitive hosts are carnivores. Zoonotic in humans (Blair et al. 1999; Yoshida et al. 2019).

**Route of infection.** Ingestion of metacercariae in infected intermediate or paratenic hosts (Blair et al. 1999; Yoshida et al. 2019).

**Site in human host.** Lungs, possibly ectopic infection at other sites (Blair et al. 1999; Yoshida et al. 2019).

**Notes.***Paragonimusohirai* is often referred to as a species complex; the status of many members as distinct and valid species is an area of ongoing investigation.

***Paragonimusskrjabini* Chen, 1959**

*Paragonimusmiyazakii* Kamo et al., 1961

*Paragonimusszechuanensis* Chung & Tsao, 1962

*Paragonimushueitungensis* Chung et al., 1975

*Paragonimusveocularis* Chen & Li, 1979

**Geographic distribution.** East and Southeast Asia, Indian subcontinent (Blair et al. 1999; Yoshida et al. 2019).

**Natural hosts.** First intermediate hosts are freshwater and brackish snails in the superfamily Rissooidea. Second intermediate hosts are freshwater crabs in the families Potamidae and Parathelphusidae. Definitive hosts are carnivores. Wild boar, rodents, and deer may serve as paratenic hosts. Zoonotic in humans (Blair et al. 1999; Yoshida et al. 2019).

**Route of infection.** Ingestion of metacercariae in infected intermediate and paratenic hosts (Blair et al. 1999; Yoshida et al. 2019).

**Site in human host.** Lungs, possibly ectopic infection at other sites (Blair et al. 1999; Yoshida et al. 2019).

**Notes.***Paragonimusskrjabini* is often referred to as a species complex; the status of many members as distinct and valid species is an area of ongoing investigation.

***Paragonimusuterobilateralis* Volker & Vogel, 1965**

**Geographic distribution.** West Africa (Blair et al. 1999; Cumberlidge et al. 2018).

**Natural hosts.** First intermediate hosts are presumed to be freshwater or brackish snails, but the specific species have yet to be identified. Second intermediate hosts are freshwater crabs in the family Potamidae. Definitive hosts are carnivores. Zoonotic in humans (Blair et al. 1999; Cumberlidge et al. 2018).

**Route of infection.** Ingestion of metacercariae in infected intermediate or paratenic hosts (Blair et al. 1999; Cumberlidge et al. 2018).

**Site in human host.** Lungs, possibly ectopic infection at other sites (Blair et al. 1999; Cumberlidge et al. 2018).

***Paragonimuswestermani* (Kerbert, 1878)**

*Distomapulmonalis* Baelz, 1880

*Distomaringeri* Cobbold, 1880

*Paragonimusedwardsi* Gulati, 1926

*Paragonimusmacacae* Sandosham, 1953

*Paragonimusasymmetricus* Chen, 1977

*Paragonimusfilipinus* Miyazaki, 1978

*Paragonimusphilippinensis* Ito et al., 1978

**Geographic distribution.** East and Southeast Asia, Indian subcontinent, Siberia (Blair et al. 1999; YBlair 2019; oshida et al. 2019).

**Natural hosts.** First intermediate hosts are freshwater and brackish snails in the superfamily Cerithioidea. Second intermediate hosts are freshwater crabs in the families Potamidae and Parathelphusidae. Definitive hosts are carnivores and non-human primates. Wild boar, rodents, and deer may serve as paratenic hosts. Zoonotic in humans (Blair et al. 1999; Blair 2019; Yoshida et al. 2019).

**Route of infection.** Ingestion of metacercariae in infected intermediate or paratenic hosts (Blair et al. 1999; Blair 2019; Yoshida et al. 2019).

**Site in human host.** Lungs, possibly ectopic infection at other sites (Blair et al. 1999; Blair 2019; Yoshida et al. 2019).

**Notes.***Paragonimuswestermani* is often referred to as a species complex; the status of many members as distinct and valid species is an area of ongoing investigation.

●●●●●●●●● **Orchipedidae Skrjabin, 1913**

Achillurbainiidae Dollfus, 1939

**Genus *Achillurbainia* Dolffus, 1939**

*Poikilorchis* Fain & Vandepitte, 1957

***Achillurbainiacongolensis* (Fain & Vandepitte, 1957)**

**Geographic distribution.** Sub-Saharan Africa (Fain and Vandepitte 1957; Nieuwenhuyse and Gatti 1968).

**Natural hosts.** Unknown; presumed zoonotic in humans (Fain and Vandepitte 1957; Nieuwenhuyse and Gatti 1968).

**Route of infection.** Unknown.

**Site in human host.** Subcutaneous cysts (usually near the ear), mastoid, middle ear (Fain and Vandepitte 1957; Nieuwenhuyse and Gatti 1968).

***Achillurbainianouveli* Dolffus, 1939**

**Geographic distribution.** Southeast Asia (human case from China) (Kannangara 1971).

**Natural hosts.** First intermediate hosts are unknown. Second intermediate hosts are freshwater crabs (*Paratelphusa*). Definitive hosts are leopards. Zoonotic in humans (Kannangara 1971; Kwo and Lim 1968).

**Route of infection.** Unknown, presumed ingestion of metacercariae in undercooked crabs (Kannangara 1971).

**Site in human host.** Subcutaneous cysts (near the ear) (Kannangara 1971).

***Achillurbainiarecondita* Travassos, 1942**

**Geographic distribution.** Central and South America (Beaver et al. 1977).

**Natural hosts.** Intermediate hosts unknown. Definitive hosts are opossums (*Didelphismarsupialis*). Zoonotic in humans (Beaver et al. 1977).

**Route of infection.** Unknown.

**Site in human host.** Peritoneal cavity (Beaver et al. 1977).

●●●● **Ecdysozoa Anguinaldo et al., 1997**

●●●●● **Panarthropoda Nielsen, 1995**

●●●●●● **Arthropoda von Siebold, 1848**

●●●●●●● **Chelicerata Heymons, 1901**

●●●●●●●● **Euchelicerata Weygoldt & Paulus, 1979**

●●●●●●●●● **Arachnida Lamarck, 1801**

●●●●●●●●●● **Acari Leach, 1817**

●●●●●●●●●●● **Acariformes Zakhvatkin, 1952**

●●●●●●●●●●●● **Sarcoptiformes Reuter, 1909**

●●●●●●●●●●●●● **Astigmatina Canestrini, 1891**

●●●●●●●●●●●●●● **Acaridia Latreille, 1802**

●●●●●●●●●●●●●●● **Histiostomatidae Berlese, 1897**

**Histiostomatidae, incertae sedis**

**Geographic distribution.** Unknown (single human case from Saudi Arabia) (Al-Arfaj et al. 2007).

**Natural hosts.** Unknown

**Route of infection.** Unknown, presumed exposure to fresh water (Al-Arfaj et al. 2007).

**Site in human host.** Ear (Al-Arfaj et al. 2007).

**Vectored pathogens.** None

**Notes.** Histostomatid mites were isolated from the ear of a Saudi man who travelled to the United States in 2007. The mites were reported as being an undescribed species of Histiostomatidae close to *Loxantoetus* (Al-Arfaj et al. 2007). At the time of this writing, the species had still not been described (Gary Mullen, pers. comm. 2020).

●●●●●●●●●●●●●●● **Acaridae Latreille, 1802**

**Genus *Sancassania* Oudemans, 1916**

***Sancassaniaberlesei* (Michael, 1903)**

**Geographic distribution.** Worldwide (Timms et al. 1981).

**Natural hosts.** None; cause incidental infestations in humans (Timms et al. 1981).

**Route of infection.** Contamination from the environment.

**Site in human host.** Mastoid cavity, ear (Cho et al. 1999; Paleri and Ruckley 2001).

**Vectored pathogens.** None.

**Genus *Cosmoglyphus* Oudemans, 1932**

**Geographic distribution.** Worldwide (Lombert et al. 1982).

**Natural hosts.** None; cause incidental infestations in humans (Lombert et al. 1982).

**Route of infection.** Contamination from the environment.

**Site in human host.** Ear (Pal et al. 2018).

**Vectored pathogens.** None.

**Notes.** Isolates of *Cosmoglyphus* from human clinical specimens have not been characterized at the species level.

**Genus *Rhizoglyphus* Claparédè, 1869**

**Geographic distribution.** Worldwide (Fan and Zhang 2004).

**Natural hosts.** None; cause incidental infestations in humans (Fan and Zhang 2004).

**Route of infection.** Contamination from the environment

**Site in human host.** Ear (Kiakojouri et al. 2018).

**Vectored pathogens.** None

**Notes.***Rhizoglyphus* from human clinical specimens have not been characterized at the species level.

●●●●●●●●●●●●●●● **Chorotoglyphidae Berlese, 1897**

**Genus *Chorotoglyphus* Berlese, 1884**

***Chorotoglyphusarcuatus* (Troupeau, 1879)**

**Geographic distribution.** Worldwide (Abi-Akl 2017).

**Natural hosts.** None; cause incidental infections in humans (Abi-Akl 2017).

**Route of infection.** Contamination from the environment.

**Site in human host.** Ear (Abi-Akl 2017).

**Vectored pathogens.** None.

**Notes.** The species-level identification of *C.arcuatus* isolated from the ear of a man in Lebanon was considered tentative based on morphologic characteristics (Abi-Akl 2017).

●●●●●●●●●●●●●● **Psoroptidia Yunker, 1955**

●●●●●●●●●●●●●●● **Pyroglyphidae Cunliffe, 1958**

**Genus *Dermatophagoides* Bogdanov, 1864**

***Dermatophagoidesfarinae* Hughes, 1961**

**Geographic distribution.** Worldwide, more prevalent in North America (Hart and Fain 1988).

**Natural hosts.** None; cause incidental infections in humans (Hart and Fain 1988).

**Route of infection.** Contamination from the environment.

**Site in human host.** Ear (Liao and Chang 2012).

**Vectored pathogens.** None.

**Notes.** The finding of *D.farinae* in stool (Kapoor et al. 2019) probably represents spurious passage after the incidental ingestion of mites in contaminated foodstuffs or contamination of the stool specimen. Dust mites in urine also probably represent contamination of the urine specimens (Dini and Frean 2005; Siebers 2014).

●●●●●●●●●●●●●●● **Psoroptidae Canestrini, 1892**

**Genus *Otodectes* Canestrini, 1894**

***Otodectescynotis* (Hering, 1838)**

**Geographic distribution.** Worldwide (Sweatman 1958).

**Natural hosts.** Carnivores, primarily dogs, cats, and ferrets. Zoonotic in humans (Sweatman 1958).

**Route of infection.** Contamination from the environment.

**Site in human host.** Ear (Van de Heyning and Thienpont 1977).

**Vectored pathogens.** None.

**Genus *Psoroptes* Gervais, 1841**

***Psoroptesovis* (Hering, 1838)**

**Geographic distribution.** Worldwide (Mazyad et al. 2001; Ken et al. 2014).

**Natural hosts.** Mammals, primarily sheep, but also horses, cattle, goats. Zoonotic on humans (Mazyad et al. 2001; Ken et al. 2014).

**Route of infection.** Contact with infected animal hosts (Mazyad et al. 2001; Ken et al. 2014).

**Site in human host.** Skin (Mazyad et al. 2001; Ken et al. 2014).

**Vectored pathogens.** None.

●●●●●●●●●●●●●●● **Sarcoptidae Murray, 1877**

**Genus *Notoedres* Railliet, 1893**

***Notoedrescati* (Hering, 1838)**

**Geographic distribution.** Worldwide (Sivajothi et al. 2015).

**Natural hosts.** Cats and other felids. Zoonotic on humans (Sivajothi et al. 2015).

**Route of infection.** Contact with infected felid hosts (Chakrabarti 1986).

**Site in human host.** Skin (Chakrabarti 1986; Beck and Pfister 2006).

**Vectored pathogens.** None.

**Genus *Sarcoptes* Latreille, 1802**

***Sarcoptesscabiei* (Linnaeus, 1758)**

**Geographic distribution.** Worldwide (Arlian and Morgan 2017; Chandler and Fuller 2019).

**Natural hosts.** Humans (other animals have their own varieties and subspecies) (Arlian and Morgan 2017; Chandler and Fuller 2019).

**Route of infection.** Direct person-to-person contact or contaminated fomites (Arlian and Morgan 2017; Chandler and Fuller 2019).

**Site in human host.** Skin (Arlian and Morgan 2017; Chandler and Fuller 2019).

**Vectored pathogens.** None.

**Genus *Trixacarus* Sellnick, 1944**

***Trixacaruscaviae* Fain et al., 1972**

**Geographic distribution.** Worldwide (Dorrestein and Van Bronswijk 1979).

**Natural hosts.** Domestic Guinea pigs. Zoonotic on humans (Dorrestein and Van Bronswijk 1979).

**Route of infection.** Contact with infected Guinea pigs (Dorrestein and Van Bronswijk 1979).

**Site in human host.** Skin (Dorrestein and Van Bronswijk 1979).

**Vectored pathogens.** None.

●●●●●●●●●●●● **Trombidiformes Reuter, 1909**

●●●●●●●●●●●●● **Prostigmata Kramer, 1877**

●●●●●●●●●●●●●● **Trombidoidea Leach, 1815**

●●●●●●●●●●●●●●● **Trombiculidae Ewing, 1929**

**Genus *Eutrombicula* Ewing, 1938**

***Eutrombiculaalfreddugesi* (Oudemans, 1910)**

**Geographic distribution.** Western Hemisphere (Diaz 2009).

**Natural hosts.** Mammals and birds. Zoonotic on humans as incidental hosts (Diaz 2009).

**Route of infection.** Exposure to larvae in the environment (Diaz 2009).

**Site in human host.** Skin (Diaz 2009).

**Vectored pathogens.** None.

***Eutrombiculasarcia* (Womersley, 1944)**

**Geographic distribution.** Asia, Australia (Diaz 2009).

**Natural hosts.** Mammals and birds. Zoonotic on humans as incidental hosts (Diaz 2009).

**Route of infection.** Exposure to larvae in the environment (Diaz 2009).

**Site in human host.** Skin.

**Vectored pathogens.** None.

**Genus *Leptotrombidium* Nagayo et al., 1916**

***Leptotrombidiumakamushi* (Brumpt, 1910)**

**Geographic distribution.** Japan (Diaz 2009; Elliott et al. 2019).

**Natural hosts.** Rodents and insectivores. Zoonotic on humans as incidental hosts (Diaz 2009; Elliott et al. 2019).

**Route of infection.** Exposure to larvae in the environment (Diaz 2009; Elliott et al. 2019).

**Site in human host.** Skin.

**Vectored pathogens.***Orientiatsutsugamushi* (Oriental scrub typhus) (Diaz 2009; Elliott et al. 2019).

***Leptotrombidiumarenicola* (Traub, 1960)**

**Geographic distribution.** Malaysia (Diaz 2009; Elliott et al. 2019).

**Natural hosts.** Rodents and insectivores. Zoonotic on humans as incidental hosts (Diaz 2009; Elliott et al. 2019).

**Route of infection.** Exposure to larvae in the environment (Diaz 2009; Elliott et al. 2019).

**Site in human host.** Skin.

**Vectored pathogens.***Orientiatsutsugamushi* (Oriental scrub typhus) (Diaz 2009; Elliott et al. 2019).

***Leptotrombidiumdeliense* (Walch, 1922)**

**Geographic distribution.** Southeast Asia, Japan, Philippines, Australia, Pacific Islands (Lv et al. 2018).

**Natural hosts.** Rodents and insectivores. Zoonotic on humans as incidental hosts (Lv et al. 2018).

**Route of infection.** Exposure to larvae in the environment (Lv et al. 2018).

**Site in human host.** Skin.

**Vectored pathogens.***Orientiatsutsugamushi* (Oriental scrub typhus) (Lv et al. 2018).

***Leptotrombidiumfletcheri* (Womersley & Heaslip, 1943)**

**Geographic distribution.** Malaysia (Diaz 2009; Elliott et al. 2019).

**Natural hosts.** Rodents and insectivores. Zoonotic on humans as incidental hosts (Diaz 2009; Elliott et al. 2019)

**Route of infection.** Exposure to larvae in the environment (Diaz 2009; Elliott et al. 2019).

**Site in human host.** Skin.

**Vectored pathogens.***Orientiatsutsugamushi* (Oriental scrub typhus) (Diaz 2009; Elliott et al. 2019).

***Leptotrombidiumpallidum* (Nagayo et al., 1919)**

**Geographic distribution.** Japan (Diaz 2009; Elliott et al. 2019).

**Natural hosts.** Rodents and insectivores. Zoonotic on humans as incidental hosts (Diaz 2009; Elliott et al. 2019).

**Route of infection.** Exposure to larvae in the environment (Diaz 2009; Elliott et al. 2019).

**Site in human host.** Skin

**Vectored pathogens.***Orientiatsutsugamushi* (Oriental scrub typhus) (Diaz 2009; Elliott et al. 2019).

***Leptotrombidiumpavlovskyi* (Schluger, 1948)**

**Geographic distribution.** Eastern Russia (Diaz 2009; Elliott et al. 2019).

**Natural hosts.** Rodents and insectivores. Zoonotic on humans as incidental hosts (Diaz 2009; Elliott et al. 2019).

**Route of infection.** Exposure to larvae in the environment (Diaz 2009; Elliott et al. 2019).

**Site in human host.** Skin

**Vectored pathogens.***Orientiatsutsugamushi* (Oriental scrub typhus) (Diaz 2009; Elliott et al. 2019).

***Leptotrombidiumscutellaris* (Nagayo et al., 1921)**

**Geographic distribution.** Japan (Diaz 2009; Elliott et al. 2019).

**Natural hosts.** Rodents and insectivores. Zoonotic on humans as incidental hosts (Diaz 2009; Elliott et al. 2019).

**Route of infection.** Exposure to larvae in the environment (Diaz 2009; Elliott et al. 2019).

**Site in human host.** Skin

**Vectored pathogens.***Orientiatsutsugamushi* (Oriental scrub typhus) (Diaz 2009; Elliott et al. 2019).

**Genus *Neotrombicula* Hirst, 1925**

***Neotrombiculaautumnalis* (Shaw, 1790)**

**Geographic distribution.** Europe (Schöler et al. 2006).

**Natural hosts.** Mammals and birds. Zoonotic on humans as incidental hosts (Schöler et al. 2006).

**Route of infection.** Exposure to larvae in the environment (Diaz 2009).

**Site in human host.** Skin.

**Vectored pathogens.** None.

**Genus *Schoengastiella* Hirst, 1915**

***Schoengastiellaligula* Radford, 1946**

**Geographic distribution.** South Asia (Tilak et al. 2011; Luce-Fedrow et al. 2018).

**Natural hosts.** Rodents and insectivores. Zoonotic on humans as incidental hosts (Tilak et al. 2011; Luce-Fedrow et al. 2018).

**Route of infection.** Exposure to larvae in the environment (Tilak et al. 2011; Luce-Fedrow et al. 2018).

**Site in human host.** Skin

**Vectored pathogens.***Orientiatsutsugamushi* (Oriental scrub typhus) (Tilak et al. 2011; Luce-Fedrow et al. 2018).

●●●●●●●●●●●●●● **Cheyletoidea Leach, 1815**

●●●●●●●●●●●●●●● **Cheyletidae Leach, 1815**

**Genus *Cheyletiella* Canestrini, 1886**

***Cheyletiellablakei* Smikey, 1970**

**Geographic distribution.** Worldwide (Keh and Lane 1987; Wagner and Stallmeister 2000).

**Natural hosts.** Cats. Zoonotic on humans as incidental hosts (Keh and Lane 1987; Wagner and Stallmeister 2000).

**Route of infection.** Exposure to infected cats (Keh and Lane 1987; Wagner and Stallmeister 2000).

**Site in human host.** Skin (Keh and Lane 1987; Wagner and Stallmeister 2000).

**Vectored pathogens.** None.

***Cheyletiellaparasitovorax* Mégnin, 1877**

**Geographic distribution.** Worldwide (Harcourt-Brown 2002).

**Natural hosts.** Rabbits. Zoonotic on humans as incidental hosts (Harcourt-Brown 2002).

**Route of infection.** Exposure to infected rabbits (Milman and Dik 2017).

**Site in human host.** Skin (Milman and Dik 2017).

**Vectored pathogens.** None.

***Cheyletiellayasguri* Smiley, 1965**

**Geographic distribution.** Worldwide (Foxx and Ewing 1969).

**Natural hosts.** Dogs. Zoonotic on humans as incidental hosts (Foxx and Ewing 1969).

**Route of infection.** Exposure to infected dogs (Powell et al. 1977).

**Site in human host.** Skin (Powell et al. 1977).

**Vectored pathogens.** None.

●●●●●●●●●●●●●●● **Demodecidae Nicolet, 1855**

**Genus *Demodex* Owen, 1843**

***Demodexbrevis* Akbulatova, 1963**

**Geographic distribution.** Worldwide (Rather and Hassan 2014).

**Natural hosts.** Humans (Rather and Hassan 2014).

**Route of infection.** Direct person-to-person contact (Rather and Hassan 2014).

**Site in human host.** Skin, sebaceous glands (Rather and Hassan 2014).

**Vectored pathogens.** None.

***Demodexfolliculorum* Simon, 1842**

**Geographic distribution.** Worldwide (Rather and Hassan 2014).

**Natural hosts.** Humans (Rather and Hassan 2014).

**Route of infection.** Direct person-to-person contact (Rather and Hassan 2014).

**Site in human host.** Skin, hair follicles (Rather and Hassan 2014).

**Vectored pathogens.** None.

●●●●●●●●●●●●●● **Tarsonemoidea Kramer, 1877**

●●●●●●●●●●●●●●● **Pyemotidae Oudemans, 1937**

**Genus *Pyemotes* Amerling, 1861**

***Pyemotesherfsi* (Oudemans, 1936)**

**Geographic distribution.** North America, Europe, North Africa, India, Australia (Broce et al. 2006).

**Natural hosts.** Insects. Zoonotic on humans as incidental hosts (Broce et al. 2006).

**Route of infection.** Environmental exposure (Broce et al. 2006).

**Site in human host.** Skin (Broce et al. 2006).

**Vectored pathogens.** None.

***Pyemotestritici* (LaGrèze-Fossat & Montagné, 1851)**

**Geographic distribution.** Worldwide (Yu et al. 2010).

**Natural hosts.** Insects. Zoonotic on humans as incidental hosts (Yu et al. 2010).

**Route of infection.** Environmental exposure (Rosen et al. 2002).

**Site in human host.** Skin (Rosen et al. 2002).

**Vectored pathogens.** None.

***Pyemotesventricosus* (Newport, 1850)**

**Geographic distribution.** Southern Europe (Moser 1975).

**Natural hosts.** Insects. Zoonotic on humans as incidental hosts (Moser 1975).

**Route of infection.** Environmental exposure (Diaz 2009).

**Site in human host.** Skin (Diaz 2009).

**Vectored pathogens.** None.

●●●●●●●●●●● **Parasitiformes Leach, 1815**

●●●●●●●●●●●● **Ixodoidea Leach, 1815**

●●●●●●●●●●●●● **Ixodidae Koch, 1844**

**Genus *Amblyomma* Koch, 1844**

***Amblyommaamericanum* (Linnaeus, 1758)**

*Ixodesunipunctata* Packard, 1869

**Geographic distribution.** Eastern North America (Cooley 1944; Guglielmone and Robbins 2018).

**Natural hosts.** Many birds and mammals, including humans (Cooley 1944; Guglielmone and Robbins 2018).

**Route of infection.** Exposure to larval, nymph, or adult ticks in the environment.

**Site in human host.** Skin.

**Vectored pathogens.***Ehrlichiachaffeensis* (human monocytic ehrlichiosis), *E.ewingii* (human granulocytic ehrlichiosis), *Francisellatularensis* (tularemia), Heartland virus, Bourbon virus; also implicated in alpha-gal syndrome and Southern Tick-Associated Rash Illness (STARI) (Madison-Antenucci et al. 2020).

***Amblyommaaureolatum* (Pallas, 1772)**

*Amblyommastriatum* Koch, 1844

**Geographic distribution.** South America (Guglielmone and Robbins 2018).

**Natural hosts.** Mammals, including carnivores and rodents, and birds. Zoonotic on humans (Guglielmone and Robbins 2018).

**Route of infection.** Exposure to larval, nymph, or adult ticks in the environment.

**Site in human host.** Skin.

**Vectored pathogens.***Rickettsiarickettsii* (Rocky Mountain Spotted Fever) (Szabó et al. 2013).

***Amblyommababirussae* Schülze, 1933**

**Geographic distribution.** Indonesia (Guglielmone and Robbins 2018).

**Natural hosts.** Mammals, including pigs, bovids, deer, carnivores, and rodents. Zoonotic on humans (Guglielmone and Robbins 2018).

**Route of infection.** Exposure to larval, nymph, or adult ticks in the environment.

**Site in human host.** Skin.

**Vectored pathogens.** None.

***Amblyommabrasiliense* Aragão, 1908**

**Geographic distribution.** South America (Guglielmone and Robbins 2018).

**Natural hosts.** Several mammals and birds. Zoonotic on humans (Guglielmone and Robbins 2018).

**Route of infection.** Exposure to larval, nymph, or adult ticks in the environment.

**Site in human host.** Skin.

**Vectored pathogens.** None.

***Amblyommabreviscutatum* Neumann, 1899**

*Amblyommacyprium* Koch & Neumann, 1899

*Amblyommacypriumaeratipes* Schülze, 1932

**Geographic distribution.** Southeast Asia, Australia, Pacific Islands (Guglielmone and Robbins 2018).

**Natural hosts.** Immature stages primarily on rodents and small birds. Adults are primarily on mammals, including bovids, deer, pigs, horses. Zoonotic on humans (Guglielmone and Robbins 2018).

**Route of infection.** Exposure to larval, nymph, or adult ticks in the environment.

**Site in human host.** Skin.

**Vectored pathogens.** None.

***Amblyommacajennense* (Fabricius, 1787)**

**Geographic distribution.** Southern United States, Central and South America, Caribbean (Cooley 1944; Martins et al. 2016; Guglielmone and Robbins 2018).

**Natural hosts.** Many mammals, including humans, and birds (Cooley 1944; Guglielmone and Robbins 2018).

**Route of infection.** Exposure to larval, nymph, or adult ticks in the environment.

**Site in human host.** Skin.

**Vectored pathogens.** None.

***Amblyommacalcartum* Neumann, 1899**

**Geographic distribution.** Central and South America, Caribbean (Guglielmone and Robbins 2018).

**Natural hosts.** Mammals, primarily anteaters; also birds. Zoonotic on humans (Guglielmone and Robbins 2018).

**Route of infection.** Exposure to larval, nymph, or adult ticks in the environment.

**Site in human host.** Skin.

**Vectored pathogens.** None.

***Amblyommaclypeolatum* Neumann, 1899**

**Geographic distribution.** India, Sri Lanka, Myanmar (Liyanaarachchi et al. 2015).

**Natural hosts.** Reptiles. Zoonotic on humans (Liyanaarachchi et al. 2015; Guglielmone and Robbins 2018).

**Route of infection.** Exposure to larval, nymph, or adult ticks in the environment.

**Site in human host.** Skin.

**Vectored pathogens.** None.

***Amblyommacoelebs* Neumann, 1899**

**Geographic distribution.** Central and South America (Guglielmone and Robbins 2018).

**Natural hosts.** Several groups of mammals and birds. Zoonotic on humans (Guglielmone and Robbins 2018).

**Route of infection.** Exposure to larval, nymph, or adult ticks in the environment.

**Site in human host.** Skin.

**Vectored pathogens.** None.

***Amblyommacohaerens* Dönitz, 1909**

**Geographic distribution.** Sub-Saharan Africa (Guglielmone and Robbins 2018).

**Natural hosts.** Primarily bovids, but also other mammals and birds. Zoonotic on humans (Guglielmone and Robbins 2018).

**Route of infection.** Exposure to larval, nymph, or adult ticks in the environment.

**Site in human host.** Skin.

**Vectored pathogens.** None.

***Amblyommacordiferum* Neumann, 1899**

**Geographic distribution.** Southeast Asia (Audy 1960).

**Natural hosts.** Immature stages are primarily on rodents. Adults are on reptiles and ungulates. Zoonotic on humans (Audy 1960).

**Route of infection.** Exposure to larval, nymph, or adult ticks in the environment.

**Site in human host.** Skin.

**Vectored pathogens.** None.

**Notes.** The single record of *A.cordiferum* from a human in Malaysia (Audy 1960) is considered a tentative identification (Guglielmone and Robbins 2018).

***Amblyommadissimile* Koch, 1844**

**Geographic distribution.** North, Central, and South America and Caribbean (Cooley 1944; Guglielmone and Robbins 2018).

**Natural hosts.** Primarily reptiles and amphibians, but also a variety of mammals and birds. Zoonotic on humans (Cooley 1944; Guglielmone and Robbins 2018).

**Route of infection.** Exposure to larval, nymph, or adult ticks in the environment.

**Site in human host.** Skin.

**Vectored pathogens.** None.

***Amblyommadubitatum* Neumann, 1899**

*Amblyommacooperi* Nuttall & Warburton, 1908

**Geographic distribution.** South America (Guglielmone and Robbins 2018).

**Natural hosts.** Primarily rodents, but also other mammals and birds. Zoonotic on humans (Guglielmone and Robbins 2018).

**Route of infection.** Exposure to larval, nymph, or adult ticks in the environment.

**Site in human host.** Skin.

**Vectored pathogens.** None.

***Amblyommafalsomarmoreum* Tonelli-Rondelli, 1935**

**Geographic distribution.** Sub-Saharan Africa (Guglielmone and Robbins 2018).

**Natural hosts.** Reptiles, occasionally other mammals. Zoonotic on humans (Guglielmone and Robbins 2018).

**Route of infection.** Exposure to larval, nymph, or adult ticks in the environment.

**Site in human host.** Skin.

**Vectored pathogens.** None.

***Amblyommafuscum* Neumann, 1907**

**Geographic distribution.** Brazil (Guglielmone and Robbins 2018).

**Natural hosts.** Immature stages on mammals, including rodents, carnivores, and marsupials. Adults usually on reptiles and amphibians. Zoonotic on humans (Guglielmone and Robbins 2018).

**Route of infection.** Exposure to larval, nymph, or adult ticks in the environment.

**Site in human host.** Skin.

**Vectored pathogens.** None.

***Amblyommagemma* Dönitz, 1909**

**Geographic distribution.** Sub-Saharan Africa (Guglielmone and Robbins 2018).

**Natural hosts.** Immature stages on small mammals and birds. Adults primarily on bovids, pigs, camels, and horses, but also other mammals, large birds, and reptiles. Zoonotic on humans (Guglielmone and Robbins 2018).

**Route of infection.** Exposure to larval, nymph, or adult ticks in the environment.

**Site in human host.** Skin.

**Vectored pathogens.** None.

***Amblyommageomydae* (Cantor, 1847)**

*Amblyommamalayanum* Neumann, 1906

**Geographic distribution.** Southeast Asia (Guglielmone and Robbins 2018).

**Natural hosts.** Immature stages on a variety of small mammals, birds, and reptiles. Adults primarily on tortoises, but also several mammals, including pigs, bovids, deer. Zoonotic on humans (Guglielmone and Robbins 2018).

**Route of infection.** Exposure to larval, nymph, or adult ticks in the environment.

**Site in human host.** Skin.

**Vectored pathogens.** None.

***Amblyommahadanii* Nava et al., 2014**

**Geographic distribution.** Argentina (Guglielmone and Robbins 2018).

**Natural hosts.** Primarily bovids, tapirs, horses, also carnivores. Zoonotic on humans (Guglielmone and Robbins 2018).

**Route of infection.** Exposure to larval, nymph, or adult ticks in the environment.

**Site in human host.** Skin.

**Vectored pathogens.** None.

***Amblyommahebraeum* Koch, 1844**

**Geographic distribution.** Sub-Saharan Africa (Guglielmone and Robbins 2018).

**Natural hosts.** Mammals, including bovids, goats, sheep, other ruminants, and humans; occasionally birds and reptiles (Guglielmone and Robbins 2018).

**Route of infection.** Exposure to larval, nymph, or adult ticks in the environment.

**Site in human host.** Skin.

**Vectored pathogens.***Rickettsiaafricae* (African tick bite fever) (Jongejan et al. 2020).

***Amblyommaincisum* Neumann, 1906**

**Geographic distribution.** South America (Guglielmone and Robbins 2018).

**Natural hosts.** Mammals, primarily tapirs, but also rodents, deer, and carnivores. Zoonotic on humans (Guglielmone and Robbins 2018).

**Route of infection.** Exposure to larval, nymph, or adult ticks in the environment.

**Site in human host.** Skin.

**Vectored pathogens.** None.

***Amblyommainoratum* (Banks, 1909)**

**Geographic distribution.** North and Central America (Guglielmone and Robbins 2018).

**Natural hosts.** Several groups of mammals and birds. Zoonotic on humans (Guglielmone and Robbins 2018).

**Route of infection.** Exposure to larval, nymph, or adult ticks in the environment.

**Site in human host.** Skin.

**Vectored pathogens.** None.

***Amblyommaintegrum* Karsch, 1879**

**Geographic distribution.** India, Sri Lanka (Guglielmone and Robbins 2018).

**Natural hosts.** Primarily bovids, but also other mammals and birds. Zoonotic on humans (Guglielmone and Robbins 2018).

**Route of infection.** Exposure to larval, nymph, or adult ticks in the environment.

**Site in human host.** Skin, ears (Dilrukshi et al. 2004).

**Vectored pathogens.** None.

***Amblyommajavanense* (Supino, 1897)**

*Aponommasublaeve* Neumann, 1899

**Geographic distribution.** Central, Southeastern Asia (Guglielmone and Robbins 2018).

**Natural hosts.** Mammals, primarily pangolins, but also rodents, and carnivores, and reptiles. Zoonotic on humans (Guglielmone and Robbins 2018).

**Route of infection.** Exposure to larval, nymph, or adult ticks in the environment.

**Site in human host.** Skin.

**Vectored pathogens.** None.

***Amblyommalatepunctatum* Tonelli-Rondelli, 1939**

**Geographic distribution.** South America (Guglielmone and Robbins 2018).

**Natural hosts.** Mammals, primarily tapirs and peccaries, also marsupials and rodents. Zoonotic on humans (Guglielmone and Robbins 2018).

**Route of infection.** Exposure to larval, nymph, or adult ticks in the environment.

**Site in human host.** Skin.

**Vectored pathogens.** None.

***Amblyommalatum* Koch, 1844**

**Geographic distribution.** Sub-Saharan Africa (Guglielmone and Robbins 2018).

**Natural hosts.** Primarily reptiles, also amphibians, rodents, and shrews. Zoonotic on humans (Guglielmone and Robbins 2018).

**Route of infection.** Exposure to larval, nymph, or adult ticks in the environment.

**Site in human host.** Skin.

**Vectored pathogens.** None.

***Ambylommalepidum* Dönitz, 1909**

**Geographic distribution.** Sub-Saharan Africa, Middle East (Guglielmone and Robbins 2018).

**Natural hosts.** Primarily bovids, but also other mammals and birds. Zoonotic on humans (Guglielmone and Robbins 2018).

**Route of infection.** Exposure to larval, nymph, or adult ticks in the environment.

**Site in human host.** Skin.

**Vectored pathogens.** None.

***Amblyommalimbatum* Neumann, 1899**

**Geographic distribution.** Australia (Guglielmone and Robbins 2018).

**Natural hosts.** Reptiles. Zoonotic on humans (Guglielmone and Robbins 2018).

**Route of infection.** Exposure to larval, nymph, or adult ticks in the environment.

**Site in human host.** Skin.

**Vectored pathogens.** None.

***Amblyommaloculosum* Neumann, 1907**

**Geographic distribution.** Sub-Saharan Africa, Australia, several islands in the Indian and South Pacific Oceans (Guglielmone and Robbins 2018).

**Natural hosts.** Primarily birds and reptiles, occasionally bovids. Zoonotic on humans (Guglielmone and Robbins 2018).

**Route of infection.** Exposure to larval, nymph, or adult ticks in the environment.

**Site in human host.** Skin.

**Vectored pathogens.** None.

***Amblyommalongirostre* (Koch, 1844)**

**Geographic distribution.** Central and South America (Guglielmone and Robbins 2018).

**Natural hosts.** Primarily birds (immature stages) and New World porcupines (adults), but also a variety of mammals and birds. Zoonotic on humans (Guglielmone and Robbins 2018).

**Route of infection.** Exposure to larval, nymph, or adult ticks in the environment.

**Site in human host.** Skin.

**Vectored pathogens.** None.

***Amblyommamaculatum* Koch, 1844**

**Geographic distribution.** Southern USA, Central and South America (Cooley 1944; Guglielmone and Robbins 2018; Lado et al. 2018).

**Natural hosts.** Many reptiles, birds, and mammals, including humans (Cooley 1944; Guglielmone and Robbins 2018).

**Route of infection.** Exposure to larval, nymph, or adult ticks in the environment.

**Site in human host.** Skin.

**Vectored pathogens.***Rickettsiaparkeri* (tidewater spotted fever) (Sumner et al. 2007; Lee et al. 2019).

***Amblyommamarmoreum* Koch, 1844**

**Geographic distribution.** Sub-Saharan Africa (Guglielmone and Robbins 2018).

**Natural hosts.** Immature stages primarily on birds and mammals. Adults on tortoises. Zoonotic on humans (Guglielmone and Robbins 2018).

**Route of infection.** Exposure to larval, nymph, or adult ticks in the environment.

**Site in human host.** Skin.

**Vectored pathogens.** None.

***Amblyommamixtum* Koch, 1844**

**Geographic distribution.** Southern USA, Central and South America (Cooley 1944; Guglielmone and Robbins 2018).

**Natural hosts.** Variety of mammals, including humans, also birds, amphibians, reptiles (Cooley 1944; Guglielmone and Robbins 2018).

**Route of infection.** Exposure to larval, nymph, or adult ticks in the environment.

**Site in human host.** Skin.

**Vectored pathogens.***Rickettsiarickettsii* (Rocky Mountain spotted fever) (Bermudez and Troyo 2018).

***Amblyommamoreliae* (Koch, 1867)**

**Geographic distribution.** Australia (Roberts 1970).

**Natural hosts.** Reptiles. Zoonotic on humans (Roberts 1970).

**Route of infection.** Exposure to larval, nymph, or adult ticks in the environment.

**Site in human host.** Skin.

**Vectored pathogens.** None.

***Amblyommanaponense* (Packard, 1869)**

*Amblyommamantiquirense* Aragão, 1908

**Geographic distribution.** Central and South America (Guglielmone and Robbins 2018).

**Natural hosts.** Primarily mammals, including peccaries, carnivores, anteaters, marsupials, and rodents, and birds. Zoonotic on humans (Guglielmone and Robbins 2018).

**Route of infection.** Exposure to larval, nymph, or adult ticks in the environment.

**Site in human host.** Skin.

**Vectored pathogens.** None.

***Amblyommaneumanni* Ribaga, 1902**

*Amblyommafurcula* Dönitz, 1909

**Geographic distribution.** Argentina, Colombia (Guglielmone and Robbins 2018).

**Natural hosts.** Mammals, including bovids, deer, carnivores, pigs, peccaries, and humans (Guglielmone and Robbins 2018).

**Route of infection.** Exposure to larval, nymph, or adult ticks in the environment.

**Site in human host.** Skin.

**Vectored pathogens.** None.

***Amblyommanuttalli* Dönitz, 1909**

**Geographic distribution.** Sub-Saharan Africa (Guglielmone and Robbins 2018).

**Natural hosts.** Immature stages on a variety of mammals, birds, and reptiles. Adults primarily on tortoises. Zoonotic on humans (Guglielmone and Robbins 2018).

**Route of infection.** Exposure to larval, nymph, or adult ticks in the environment.

**Site in human host.** Skin.

**Vectored pathogens.** None.

***Ambylommaoblongoguttatum* Koch, 1844**

**Geographic distribution.** Central and South America (Guglielmone and Robbins 2018).

**Natural hosts.** A variety of mammals, including deer, rodents, carnivores, and marsupials, and birds. Zoonotic on humans (Guglielmone and Robbins 2018).

**Route of infection.** Exposure to larval, nymph, or adult ticks in the environment.

**Site in human host.** Skin.

**Vectored pathogens.** None.

***Amblyommaovale* Koch, 1844**

*Amblyommafossum* Neumann, 1899

**Geographic distribution.** Southern USA, Central and South America (Cooley 1944; Guglielmone and Robbins 2018).

**Natural hosts.** Mammals, primarily carnivores, rodents, tapirs, and humans. Occasionally marsupials, birds (Cooley 1944; Guglielmone and Robbins 2018).

**Route of infection.** Exposure to larval, nymph, or adult ticks in the environment.

**Site in human host.** Skin.

**Vectored pathogens.** None.

***Amblyommapacae* Aragão, 1911**

**Geographic distribution.** Central and South America (Guglielmone and Robbins 2018).

**Natural hosts.** Primarily mammals, including rodents and marsupials, but also carnivores and birds. Zoonotic on humans (Guglielmone and Robbins 2018).

**Route of infection.** Exposure to larval, nymph, or adult ticks in the environment.

**Site in human host.** Skin.

**Vectored pathogens.** None.

***Ambylommaparkeri* Fonseca & Aragão, 1951**

**Geographic distribution.** Brazil (Guglielmone and Robbins 2018).

**Natural hosts.** Primarily rodents, also carnivores, marsupials, monkeys, and birds. Zoonotic on humans (Guglielmone and Robbins 2018).

**Route of infection.** Exposure to larval, nymph, or adult ticks in the environment.

**Site in human host.** Skin.

**Vectored pathogens.** None.

***Amblyommaparvum* Aragão, 1908**

**Geographic distribution.** Central and South America (Guglielmone and Robbins 2018).

**Natural hosts.** Wide variety of mammals, including humans, and birds (Guglielmone and Robbins 2018).

**Route of infection.** Exposure to larval, nymph, or adult ticks in the environment.

**Site in human host.** Skin.

**Vectored pathogens.** None.

***Amblyommapaulopunctatum* Neumann, 1899**

**Geographic distribution.** Sub-Saharan Africa (Guglielmone and Robbins 2018).

**Natural hosts.** Mammals, primarily pigs, but also hippopotamuses, carnivores, and rodents, and birds. Zoonotic on humans (Guglielmone and Robbins 2018).

**Route of infection.** Exposure to larval, nymph, or adult ticks in the environment.

**Site in human host.** Skin.

**Vectored pathogens.** None.

***Amblyommapecarium* Dunn, 1933**

**Geographic distribution.** Central and South America (Guglielmone and Robbins 2018).

**Natural hosts.** Peccaries, also deer. Zoonotic on humans (Guglielmone and Robbins 2018).

**Route of infection.** Exposure to larval, nymph, or adult ticks in the environment.

**Site in human host.** Skin.

**Vectored pathogens.** None.

***Amblyommapersonatum* Neumann, 1901**

**Geographic distribution.** Sub-Saharan Africa (Guglielmone and Robbins 2018).

**Natural hosts.** Rhinoceroses, bovids. Zoonotic on humans(Guglielmone and Robbins 2018).

**Route of infection.** Exposure to larval, nymph, or adult ticks in the environment.

**Site in human host.** Skin.

**Vectored pathogens.** None.

***Amblyommapostoculatum* Neumann, 1899**

**Geographic distribution.** Australia (Roberts 1970).

**Natural hosts.** Marsupials. Zoonotic on humans (Roberts 1970).

**Route of infection.** Exposure to larval, nymph, or adult ticks in the environment.

**Site in human host.** Skin.

**Vectored pathogens.** None.

***Amblyommapseudoconcolor* Aragão, 1908**

**Geographic distribution.** South America (Guglielmone and Robbins 2018).

**Natural hosts.** Primarily armadillos, also marsupials and birds. Zoonotic on humans (Guglielmone and Robbins 2018).

**Route of infection.** Exposure to larval, nymph, or adult ticks in the environment.

**Site in human host.** Skin.

**Vectored pathogens.** None.

***Amblyommapseudoparvum* Guglielmone et al., 1990**

**Geographic distribution.** Argentina (Guglielmone and Robbins 2018).

**Natural hosts.** Caviomorph rodents. Zoonotic on humans (Guglielmone and Robbins 2018).

**Route of infection.** Exposure to larval, nymph, or adult ticks in the environment.

**Site in human host.** Skin.

**Vectored pathogens.** None.

***Amblyommaromitii* Tonelli-Rondelli, 1939**

*Amblyommatasquei* Floch & Adonnenc, 1940

**Geographic distribution.** South America (Guglielmone and Robbins 2018).

**Natural hosts.** Rodents, marsupials. Zoonotic on humans (Guglielmone and Robbins 2018).

**Route of infection.** Exposure to larval, nymph, or adult ticks in the environment.

**Site in human host.** Skin.

**Vectored pathogens.** None.

***Amblyommarotundum* Koch, 1844**

**Geographic distribution.** Southern USA, Central and South America, Pacific Islands (Cooley 1944; Guglielmone and Robbins 2018).

**Natural hosts.** Reptiles and amphibians, occasionally birds. Zoonotic on humans (Guglielmone and Robbins 2018).

**Route of infection.** Exposure to larval, nymph, or adult ticks in the environment.

**Site in human host.** Skin.

**Vectored pathogens.** None.

***Amblyommasabanerae* Stoll, 1890**

**Geographic distribution.** Central and South America (Guglielmone and Robbins 2018).

**Natural hosts.** Primarily reptiles, including tortoises, also amphibians, rodents, and marsupials. Zoonotic on humans (Guglielmone and Robbins 2018).

**Route of infection.** Exposure to larval, nymph, or adult ticks in the environment.

**Site in human host.** Skin.

**Vectored pathogens.** None.

***Amblyommascalpturatum* Neumann, 1906**

**Geographic distribution.** Central and South America (Guglielmone and Robbins 2018).

**Natural hosts.** Mammals, including tapirs, pigs, anteaters, peccaries, carnivores, and rodents. Zoonotic on humans (Guglielmone and Robbins 2018).

**Route of infection.** Exposure to larval, nymph, or adult ticks in the environment.

**Site in human host.** Skin.

**Vectored pathogens.** None.

***Amblyommasculptum* (Berlese, 1888)**

**Geographic distribution.** South America (Martins et al. 2016; Guglielmone and Robbins 2018).

**Natural hosts.** Several mammals, including capybaras and humans, and birds (Osava et al. 2016; Guglielmone and Robbins 2018).

**Route of infection.** Exposure to larval, nymph, or adult ticks in the environment

**Site in human host.** Skin.

**Vectored pathogens.***Rickettsiarickettsii* (Rocky Mountain Spotted Fever) (Ramírez-Hernández et al. 2020).

***Amblyommasparsum* Neumann, 1899**

**Geographic distribution.** Sub-Saharan Africa (Guglielmone and Robbins 2018).

**Natural hosts.** Several groups of mammals, birds, and reptiles. Zoonotic on humans (Guglielmone and Robbins 2018).

**Route of infection.** Exposure to larval, nymph, or adult ticks in the environment.

**Site in human host.** Skin.

**Vectored pathogens.** None.

***Amblyommatapirellum* Dunn, 1933**

**Geographic distribution.** Central and South America (Guglielmone and Robbins 2018).

**Natural hosts.** Tapirs, peccaries, marsupials, rodents. Zoonotic on humans (Guglielmone and Robbins 2018).

**Route of infection.** Exposure to larval, nymph, or adult ticks in the environment.

**Site in human host.** Skin.

**Vectored pathogens.** None.

***Amblyommatenellum* Koch, 1844**

*Amblyommaimitator* Kohls, 1958

**Geographic distribution.** Southern USA, Central America (Guglielmone and Robbins 2018).

**Natural hosts.** Mammals, including bovids, peccaries, horses, marsupials, carnivores, and rodents, and birds. Zoonotic on humans (Guglielmone and Robbins 2018).

**Route of infection.** Exposure to larval, nymph, or adult ticks in the environment.

**Site in human host.** Skin.

**Vectored pathogens.** None.

***Amblyommatestudinarum* Koch, 1844**

**Geographic distribution.** Central and Southeast Asia, Japan (Guglielmone and Robbins 2018).

**Natural hosts.** Several groups of mammals, including humans, birds, and reptiles (Guglielmone and Robbins 2018),

**Route of infection.** Exposure to larval, nymph, or adult ticks in the environment

**Site in human host.** Skin, ear (Nakao et al. 2017).

**Vectored pathogens.** Dabie bandavirus (severe fever with thrombocytopenia syndrome Virus, SFTSV) (Sato et al. 2021).

***Amblyommatholloni* Neumann, 1899**

**Geographic distribution.** Sub-Saharan Africa (Guglielmone and Robbins 2018).

**Natural hosts.** Several groups of mammals, primarily elephants, and birds and reptiles. Zoonotic on humans (Guglielmone and Robbins 2018).

**Route of infection.** Exposure to larval, nymph, or adult ticks in the environment.

**Site in human host.** Skin.

**Vectored pathogens.** None.

***Amblyommatigrinum* Koch, 1844**

**Geographic distribution.** South America (Guglielmone and Robbins 2018).

**Natural hosts.** Primarily carnivores, also rodents and ungulates. Zoonotic on humans (Guglielmone and Robbins 2018).

**Route of infection.** Exposure to larval, nymph, or adult ticks in the environment.

**Site in human host.** Skin.

**Vectored pathogens.** None.

***Amblyommatonelliae* Nava et al., 2014**

**Geographic distribution.** South America (Guglielmone and Robbins 2018).

**Natural hosts.** Mammals, primarily bovids and equids. Zoonotic on humans (Guglielmone and Robbins 2018).

**Route of infection.** Exposure to larval, nymph, or adult ticks in the environment.

**Site in human host.** Skin.

**Vectored pathogens.***Rickettsiaricketsii* (Rocky Mountain spotted fever) (Tarragona et al. 2016).

***Amblyommatriguttatum* Koch, 1844**

**Geographic distribution.** Australia (Guglielmone and Robbins 2018).

**Natural hosts.** Primarily marsupials, also horses, bovids, carnivores, lagomorphs, rodents, humans, and reptiles (Guglielmone and Robbins 2018).

**Route of infection.** Exposure to larval, nymph, or adult ticks in the environment.

**Site in human host.** Skin.

**Vectored pathogens.** None.

***Amblyommatriste* Koch, 1844**

**Geographic distribution.** Southern USA, Central and South America (Cooley 1944; Guglielmone and Robbins 2018).

**Natural hosts.** Several groups of mammals, primarily bovids, deer, and carnivores, and birds. Zoonotic on humans (Cooley 1944; Guglielmone and Robbins 2018).

**Route of infection.** Exposure to larval, nymph, or adult ticks in the environment.

**Site in human host.** Skin.

**Vectored pathogens.***Rickettsiaparkeri* (Tidewater spotted fever) (Silveira et al. 2007).

***Amblyommatuberculatum* Marx in Hubbard, 1894**

**Geographic distribution.** Southeastern USA (Cooley 1944; Guglielmone and Robbins 2018).

**Natural hosts.** Tortoises. Zoonotic on humans (Cooley 1944; Guglielmone and Robbins 2018).

**Route of infection.** Exposure to larval, nymph, or adult ticks in the environment.

**Site in human host.** Skin.

**Vectored pathogens.** None.

***Amblyommavariegatum* (Fabricius, 1794)**

**Geographic distribution.** Africa, Middle East, Caribbean (Guglielmone and Robbins 2018).

**Natural hosts.** Several groups of mammals, primarily bovids, also humans, birds, and reptiles (Guglielmone and Robbins 2018).

**Route of infection.** Exposure to larval, nymph, or adult ticks in the environment.

**Site in human host.** Skin.

**Vectored pathogens.***Rickettsiaafricae* (African tick bite fever) (Dantas-Torres et al. 2012).

***Amblyommavarium* Koch, 1844**

**Geographic distribution.** Central and South America (Guglielmone and Robbins 2018).

**Natural hosts.** Mammals, primarily sloths, also marsupials, tapirs, carnivores, and rodents, also reptiles and birds. Zoonotic on humans (Guglielmone and Robbins 2018).

**Route of infection.** Exposure to larval, nymph, or adult ticks in the environment.

**Site in human host.** Skin.

**Vectored pathogens.** None.

**Genus *Bothriocroton* Keirans et al., 1994**

***Bothriocrotonauruginans* (Schülze, 1938)**

**Geographic distribution.** Australia (Guglielmone and Robbins 2018).

**Natural hosts.** Marsupials, occasionally carnivores. Zoonotic on humans (Guglielmone and Robbins 2018).

**Route of infection.** Exposure to larval, nymph, or adult ticks in the environment.

**Site in human host.** Skin.

**Vectored pathogens.** None.

***Bothriocrotonhydrosauri* (Denny, 1843)**

**Geographic distribution.** Australia (Guglielmone and Robbins 2018).

**Natural hosts.** Reptiles, occasionally bovids. Zoonotic on humans (Guglielmone and Robbins 2018).

**Route of infection.** Exposure to larval, nymph, or adult ticks in the environment.

**Site in human host.** Skin.

**Vectored pathogens.** None.

**Genus *Dermacentor* Koch, 1844**

***Dermacentoralbipictus* (Packard, 1869)**

*Ixodesnigrolineatus* Packard, 1869

**Geographic distribution.** North and Central America (Guglielmone and Robbins 2018).

**Natural hosts.** Mammals, primarily cattle, deer, and horses. Zoonotic on humans (Guglielmone and Robbins 2018).

**Route of infection.** Exposure to larval, nymph, or adult ticks in the environment.

**Site in human host.** Skin.

**Vectored pathogens.***Babesiaduncani* (babesiosis) (Swei et al. 2019).

***Dermacentorandersoni* Stiles, 1908**

*Dermacentorvenustus* Marx in Neumann 1897

**Geographic distribution.** Rocky Mountain region of North America (Guglielmone and Robbins 2018).

**Natural hosts.** Mammals, including humans (Eisen 2007; Guglielmone and Robbins 2018).

**Route of infection.** Exposure to larval, nymph, or adult ticks in the environment

**Site in human host.** Skin

**Vectored pathogens.***Rickettsiarickettsii* (Rocky Mountain spotted fever), *Francisellatularensis* (tularemia), Colorado tick fever virus; also implicated in tick paralysis (Eisen 2007).

***Dermacentoratrosignatus* Neumann, 1906**

**Geographic distribution.** Southeast Asia (Guglielmone and Robbins 2018).

**Natural hosts.** Pigs. Zoonotic on humans (Guglielmone and Robbins 2018).

**Route of infection.** Exposure to larval, nymph, or adult ticks in the environment.

**Site in human host.** Skin, ear (Mariana et al. 2008).

**Vectored pathogens.** None.

***Dermacentorauratus* Supino, 1897**

**Geographic distribution.** Southeast Asia (Guglielmone and Robbins 2018).

**Natural hosts.** Primarily pigs, but also a variety of other mammals, birds, and reptiles. Zoonotic on humans (Guglielmone and Robbins 2018).

**Route of infection.** Exposure to larval, nymph, or adult ticks in the environment.

**Site in human host.** Skin, ear (Ariyarathne et al. 2011).

**Vectored pathogens.***Rickettsiasibirica* (Siberian tick typhus) (Dantas-Torres et al. 2012).

***Dermacentorbellulus* (Schülze, 1933)**

**Geographic distribution.** Southeast Asia (Guglielmone and Robbins 2018).

**Natural hosts.** Immature stages primarily on rodents, also tree shrews, carnivores, lagomorphs, and birds. Adults are primarily on pigs, also carnivores. Zoonotic on humans (Guglielmone and Robbins 2018).

**Route of infection.** Exposure to larval, nymph, or adult ticks in the environment.

**Site in human host.** Skin.

**Vectored pathogens.** None.

***Dermacentorcircumguttatus* Neumann, 1897**

**Geographic distribution.** Sub-Saharan Africa (Guglielmone and Robbins 2018).

**Natural hosts.** Primarily elephants, also duikers, pigs. Zoonotic on humans (Guglielmone and Robbins 2018).

**Route of infection.** Exposure to larval, nymph, or adult ticks in the environment.

**Site in human host.** Skin.

**Vectored pathogens.** None.

***Dermacentorcompactus* Neumann, 1901**

**Geographic distribution.** Southeast Asia (Guglielmone and Robbins 2018).

**Natural hosts.** Primarily pigs, but also a variety of other mammals and reptiles. Zoonotic on humans (Guglielmone and Robbins 2018).

**Route of infection.** Exposure to larval, nymph, or adult ticks in the environment.

**Site in human host.** Skin, ear (Mariana et al. 2008).

**Vectored pathogens.** None.

***Dermacentorhunteri* Bishopp, 1912**

**Geographic distribution.** Southeast Asia (Guglielmone and Robbins 2018).

**Natural hosts.** Primarily bovids, also deer, lagomorphs, and rodents. Zoonotic on humans (Guglielmone and Robbins 2018).

**Route of infection.** Exposure to larval, nymph, or adult ticks in the environment.

**Site in human host.** Skin.

**Vectored pathogens.** None.

***Dermacentorimitans* Warburton, 1933**

**Geographic distribution.** Central and South America (Guglielmone and Robbins 2018).

**Natural hosts.** Peccaries, deer. Zoonotic on humans (Guglielmone and Robbins 2018).

**Route of infection.** Exposure to larval, nymph, or adult ticks in the environment.

**Site in human host.** Skin.

**Vectored pathogens.** None.

***Dermacentorlatus* Cooley, 1937**

**Geographic distribution.** Central America (Guglielmone and Robbins 2018).

**Natural hosts.** Primarily tapirs, also carnivores. Zoonotic on humans (Guglielmone and Robbins 2018).

**Route of infection.** Exposure to larval, nymph, or adult ticks in the environment.

**Site in human host.** Skin.

**Vectored pathogens.** None.

***Dermacentorlimbooliati* Apanaskevich & Apanaskevich, 2015**

**Geographic distribution.** Malaysia, Vietnam (Apanaskevich and Apanaskevich 2015).

**Natural hosts.** Pigs. Zoonotic on humans (Apanaskevich and Apanaskevich 2015).

**Route of infection.** Exposure to larval, nymph, or adult ticks in the environment.

**Site in human host.** Skin.

**Vectored pathogens.** None.

***Dermacentormarginatus* (Sulzer, 1776)**

**Geographic distribution.** Europe, western and central Asia, northern Africa (Rubel et al. 2016; Guglielmone and Robbins 2018).

**Natural hosts.** Many mammals, including humans, and birds (Guglielmone and Robbins 2018).

**Route of infection.** Exposure to larval, nymph, or adult ticks in the environment.

**Site in human host.** Skin.

**Vectored pathogens.***Rickettsiaraoultii*, Omsk hemorrhagic fever virus (Dantas-Torres et al. 2012).

***Dermacentornitens* Neumann, 1897**

**Geographic distribution.** Southern USA, Central and South America, Caribbean (Guglielmone and Robbins 2018).

**Natural hosts.** Horses, occasionally other mammals, reptiles, and amphibians. Zoonotic on humans (Guglielmone and Robbins 2018).

**Route of infection.** Exposure to larval, nymph, or adult ticks in the environment.

**Site in human host.** Skin.

**Vectored pathogens.** None.

***Dermacentorniveus* Neumann, 1897**

**Geographic distribution.** Central Asia, China, Russia (Guglielmone and Robbins 2018).

**Natural hosts.** Mammals, including ungulates, rodents, lagomorphs, hedgehogs, and carnivores. Zoonotic on humans (Guglielmone and Robbins 2018).

**Route of infection.** Exposure to larval, nymph, or adult ticks in the environment.

**Site in human host.** Skin.

**Vectored pathogens.** None.

***Dermacentornuttalli* Olenev, 1929**

**Geographic distribution.** Eastern Russia, Mongolia, China (Guglielmone and Robbins 2018).

**Natural hosts.** Immature stages on rodents, lagomorphs, hedgehogs. Adults primarily on ungulates and horses. Zoonotic on humans (Guglielmone and Robbins 2018).

**Route of infection.** Exposure to larval, nymph, or adult ticks in the environment.

**Site in human host.** Skin.

**Vectored pathogens.***Rickettsiasibirica* (Siberian tick typhus), *Francisellatularensis* (tularemia), Crimean-Congo hemorrhagic fever virus (Gui et al. 2021; Kulakova et al. 2014).

***Dermacentoroccidentalis* Marx in Curtice, 1892**

**Geographic distribution.** Pacific coastal areas of Mexico and USA (Guglielmone and Robbins 2018).

**Natural hosts.** Several groups of mammals. Immature stages primarily on rodents and lagomorphs. Adults on cattle, deer, and humans (Guglielmone and Robbins 2018).

**Route of infection.** Exposure to larval, nymph, or adult ticks in the environment.

**Site in human host.** Skin.

**Vectored pathogens.***Rickettsiaphlipii* (Pacific Coast tick fever) (Padgett et al. 2016).

***Dermacentorparumapertus* Neumann, 1901**

**Geographic distribution.** North America (Guglielmone and Robbins 2018).

**Natural hosts.** Lagomorphs, rodents, bovids, deer, carnivores, and birds. Zoonotic on humans (Guglielmone and Robbins 2018).

**Route of infection.** Exposure to larval, nymph, or adult ticks in the environment.

**Site in human host.** Skin.

**Vectored pathogens.** None.

***Dermacentorraskemensis* Pomerantsev, 1946**

**Geographic distribution.** Central and Southeast Asia (Guglielmone and Robbins 2018).

**Natural hosts.** Bovids, carnivores, rodents, lagomorphs. Zoonotic on humans (Guglielmone and Robbins 2018).

**Route of infection.** Exposure to larval, nymph, or adult ticks in the environment.

**Site in human host.** Skin.

**Vectored pathogens.** None.

***Dermacentorreticulatus* (Fabricius, 1794)**

*Dermacentorpictus* Hermann, 1804

**Geographic distribution.** Europe and western Asia (Rubel et al. 2016; Guglielmone and Robbins 2018).

**Natural hosts.** Immature stages primarily on rodents, lagomorphs, hedgehogs, rarely birds and reptiles, and amphibians. Adults primarily on canids, also ungulates, and humans (Guglielmone and Robbins 2018).

**Route of infection.** Exposure to larval, nymph, or adult ticks in the environment.

**Site in human host.** Skin.

**Vectored pathogens.***Rickettsiaslovaca* (tick-borne lymphandenopathy, TIBOLA), *Rickettsiaraoultii*, Omsk hemorrhagic fever virus (Földvári et al. 2016).

***Dermacentorrhinocerinus* (Denny, 1843)**

**Geographic distribution.** Sub-Saharan Africa (Guglielmone and Robbins 2018).

**Natural hosts.** Primarily rhinoceroses, occasionally other mammals and reptiles. Zoonotic on humans (Guglielmone and Robbins 2018).

**Route of infection.** Exposure to larval, nymph, or adult ticks in the environment.

**Site in human host.** Skin.

**Vectored pathogens.** None.

***Dermacentorsilvarum* Olenev, 1931**

*Dermacentorasiaticus* Emel’yanova & Kozlovskaya, 1967

**Geographic distribution.** Eastern Russia, China, Mongolia (Guglielmone and Robbins 2018).

**Natural hosts.** Mammals, including bovids, deer, sheep, dogs, and humans (Guglielmone and Robbins 2018).

**Route of infection.** Exposure to larval, nymph, or adult ticks in the environment.

**Site in human host.** Skin.

**Vectored pathogens.***Rickettsiasibirica* (Siberian tick typhus), *Rickettsiaraoultii* (Cao et al. 2008).

***Dermacentorsimilis* Lado, Glon, et Klompen, 2021**

**Geographic distribution.** North America, west of the Rocky Mountains (Lado et al. 2021).

**Natural hosts.** Several groups of mammals, including humans, and birds (Guglielmone and Robbins 2018).

**Route of infection.** Exposure to larval, nymph, or adult ticks in the environment.

**Site in human host.** Skin.

**Vectored pathogens.***Rickettsiarickettsii* (Rocky Mountain spotted fever), *Francisellatularensis* (tularemia); also implicated in tick paralysis (Wikswo et al. 2014; Moraru 2019; Mullen and Durden 2019).

***Dermacentorsteini* (Schülze, 1933)**

**Geographic distribution.** Southeast Asia (Guglielmone and Robbins 2018).

**Natural hosts.** Primarily pigs, but also a variety of other mammals and reptiles. Zoonotic on humans (Guglielmone and Robbins 2018).

**Route of infection.** Exposure to larval, nymph, or adult ticks in the environment.

**Site in human host.** Skin, ear (Mariana et al. 2008).

**Vectored pathogens.** None.

***Dermacentortamokensis* Apanaskevich & Apanaskevich, 2016**

**Geographic distribution.** Southeast Asia (Apanaskevich and Apanaskevich 2016; Guglielmone and Robbins 2018).

**Natural hosts.** Pigs. Zoonotic on humans (Guglielmone and Robbins 2018)

**Route of infection.** Exposure to larval, nymph, or adult ticks in the environment.

**Site in human host.** Skin.

**Vectored pathogens.** None.

***Dermacentorvariabilis* (Say, 1821)**

*Dermacentorelectus* Koch, 1844

**Geographic distribution.** Eastern North America (Guglielmone and Robbins 2018; Lado et al. 2021).

**Natural hosts.** Several groups of mammals, including humans, and birds. (Guglielmone and Robbins 2018).

**Route of infection.** Exposure to larval, nymph, or adult ticks in the environment.

**Site in human host.** Skin, ear (Grady et al. 2011).

**Vectored pathogens.***Rickettsiarickettsii* (Rocky Mountain spotted fever), *Francisellatularensis* (tularemia); also implicated in tick paralysis (Ammerman et al. 2004; Dergousoff and Chilton 2012; Moraru 2019; Mullen and Durden 2019).

**Genus *Haemaphysalis* Koch, 1844**

***Haemaphysalisaculeata* Lavarra, 1904**

**Geographic distribution.** India, Sri Lanka (Guglielmone and Robbins 2018).

**Natural hosts.** Chevrotains, bovids, deer. Zoonotic on humans (Guglielmone and Robbins 2018).

**Route of infection.** Exposure to larval, nymph, or adult ticks in the environment.

**Site in human host.** Skin.

**Vectored pathogens.** Kyasanur Forest Disease virus (Sharma et al. 2019).

***Haemaphysalisanomala* Warburton, 1913**

**Geographic distribution.** Southeast Asia (Guglielmone and Robbins 2018),

**Natural hosts.** Immature stages on rodents and small birds. Adults on bovids, deer, carnivores. Zoonotic on humans (Guglielmone and Robbins 2018).

**Route of infection.** Exposure to larval, nymph, or adult ticks in the environment.

**Site in human host.** Skin.

**Vectored pathogens.** None.

***Haemaphysalisaponommoides* Warburton, 1913**

**Geographic distribution.** China, India, Nepal (Guglielmone and Robbins 2018).

**Natural hosts.** Immature stages on rodents, shrews, birds. Adults on mammals, primarily bovids. Zoonotic on humans (Guglielmone and Robbins 2018).

**Route of infection.** Exposure to larval, nymph, or adult ticks in the environment.

**Site in human host.** Skin.

**Vectored pathogens.** None.

***Haemaphysalisbancrofti* Nuttall & Warburton, 1915**

**Geographic distribution.** Australia, Indonesia, Papua New Guinea (Guglielmone and Robbins 2018).

**Natural hosts.** Primarily marsupials, but also a variety of other mammals and birds. Zoonotic on humans (Guglielmone and Robbins 2018).

**Route of infection.** Exposure to larval, nymph, or adult ticks in the environment.

**Site in human host.** Skin.

**Vectored pathogens.** None.

***Haemaphysalisbirmaniae* Supino, 1897**

**Geographic distribution.** Southeast Asia (Guglielmone and Robbins 2018).

**Natural hosts.** Bovids, deer, pigs, carnivores, and rodents. Zoonotic on humans (Guglielmone and Robbins 2018).

**Route of infection.** Exposure to larval, nymph, or adult ticks in the environment.

**Site in human host.** Skin.

**Vectored pathogens.** None.

***Haemaphysalisbispinosa* Neumann, 1897**

**Geographic distribution.** Central and Southeast Asia (Guglielmone and Robbins 2018).

**Natural hosts.** Mammals, primarily bovids and carnivores, and birds. Zoonotic on humans (Guglielmone and Robbins 2018).

**Route of infection.** Exposure to larval, nymph, or adult ticks in the environment.

**Site in human host.** Skin.

**Vectored pathogens.** Kyasanur Forest disease virus (Sharma et al. 2019).

***Haemaphysaliscampanulata* Warburton, 1908**

**Geographic distribution.** Central and Southeast Asia, Japan (Guglielmone and Robbins 2018).

**Natural hosts.** Immature stages primarily on rodents. Adults on mammals, primarily carnivores. Zoonotic on humans (Guglielmone and Robbins 2018).

**Route of infection.** Exposure to larval, nymph, or adult ticks in the environment.

**Site in human host.** Skin.

**Vectored pathogens.** None.

***Haemaphysaliscaucasica* Olenev, 1928**

**Geographic distribution.** Eastern Europe, Central Asia (Guglielmone and Robbins 2018).

**Natural hosts.** Mammals, including lagomorphs, carnivores, rodents, and bovids, and birds. Zoonotic on humans (Guglielmone and Robbins 2018).

**Route of infection.** Exposure to larval, nymph, or adult ticks in the environment.

**Site in human host.** Skin.

**Vectored pathogens.** None.

***Haemaphysaliscelebensis* Hoogstral et al., 1965**

**Geographic distribution.** Indonesia (Guglielmone and Robbins 2018).

**Natural hosts.** Primarily pigs, bovids, deer, and horses. Zoonotic on humans (Guglielmone and Robbins 2018).

**Route of infection.** Exposure to larval, nymph, or adult ticks in the environment.

**Site in human host.** Skin.

**Vectored pathogens.** None.

***Haemaphysalischordeilis* (Packard, 1869)**

**Geographic distribution.** North America (Guglielmone and Robbins 2018).

**Natural hosts.** Primarily birds, also mammals including bovids, horses, rodents. Zoonotic on humans (Guglielmone and Robbins 2018).

**Route of infection.** Exposure to larval, nymph, or adult ticks in the environment.

**Site in human host.** Skin.

**Vectored pathogens.** None.

***Haemaphysaliscolasbelcouri* (Santos Dias, 1958)**

**Geographic distribution.** China, Vietnam, Laos (Guglielmone and Robbins 2018).

**Natural hosts.** Primarily bovids and deer. Zoonotic on humans (Guglielmone and Robbins 2018).

**Route of infection.** Exposure to larval, nymph, or adult ticks in the environment.

**Site in human host.** Skin.

**Vectored pathogens.** None.

***Haemaphysalisconcinna* Koch, 1844**

**Geographic distribution.** Palearctic (Guglielmone and Robbins 2018; Kiewra et al. 2019).

**Natural hosts.** Small mammals, birds, and reptiles. Zoonotic on humans (Guglielmone and Robbins 2018; Kiewra et al. 2019).

**Route of infection.** Exposure to larval, nymph, or adult ticks in the environment.

**Site in human host.** Skin.

**Vectored pathogens.***Francisellatularensis* (tularemia), tick-borne encephalitis viruses (TBE) (Kiewra et al. 2019).

***Haemaphysaliscornigera* Neumann, 1897**

**Geographic distribution.** Southeast Asia, Japan (Guglielmone and Robbins 2018).

**Natural hosts.** Mammals, including bovids, deer, carnivores, shrews, and tree shrews. Zoonotic on humans (Guglielmone and Robbins 2018).

**Route of infection.** Exposure to larval, nymph, or adult ticks in the environment.

**Site in human host.** Skin.

**Vectored pathogens.***Rickettsiajaponica* (Japanese spotted fever) (Li et al. 2019).

***Haemaphysaliscuspidata* Warburton, 1910**

**Geographic distribution.** India, Sri Lanka (Guglielmone and Robbins 2018).

**Natural hosts.** Many groups of mammals, including ungulates, carnivores, rodents, lagomorphs, shrews, and non-human primates, and birds. Zoonotic on humans (Guglielmone and Robbins 2018).

**Route of infection.** Exposure to larval, nymph, or adult ticks in the environment.

**Site in human host.** Skin.

**Vectored pathogens.** Kyasanur Forest disease virus (Sharma et al. 2019).

***Haemaphysalisdarjeeling* Hoogstraal & Dhanda, 1970**

**Geographic distribution.** Southeast Asia (Guglielmone and Robbins 2018).

**Natural hosts.** Mammals, including bovids, deer, pigs, and carnivores. Zoonotic on humans (Guglielmone and Robbins 2018).

**Route of infection.** Exposure to larval, nymph, or adult ticks in the environment.

**Site in human host.** Skin.

**Vectored pathogens.** None.

***Haemaphysalisdoenitzi* Warburton & Nuttall, 1909**

**Geographic distribution.** Southeast Asia, Japan, Russia, Australia (Guglielmone and Robbins 2018).

**Natural hosts.** Primarily birds, occasionally lagomorphs and reptiles. Zoonotic on humans (Guglielmone and Robbins 2018).

**Route of infection.** Exposure to larval, nymph, or adult ticks in the environment.

**Site in human host.** Skin.

**Vectored pathogens.** None.

***Haemaphysaliselliptica* (Koch, 1844)**

**Geographic distribution.** Sub-Saharan Africa (Guglielmone and Robbins 2018).

**Natural hosts.** Primarily carnivores, also rodents, elephant shrews, and birds. Zoonotic on humans (Guglielmone and Robbins 2018).

**Route of infection.** Exposure to larval, nymph, or adult ticks in the environment.

**Site in human host.** Skin.

**Vectored pathogens.** None.

***Haemaphysaliselongata* Neumann, 1897**

**Geographic distribution.** Madagascar (Guglielmone and Robbins 2018).

**Natural hosts.** Primarily tenrecs, also carnivores and hedgehogs. Zoonotic on humans (Guglielmone and Robbins 2018).

**Route of infection.** Exposure to larval, nymph, or adult ticks in the environment.

**Site in human host.** Skin.

**Vectored pathogens.** None.

***Haemaphysaliserinacei* Pavesi, 1884**

*Haemaphysalisnumidiana* Neumann, 1905

*Haemaphysaliserinaceitaurica* Pospelova-Shtrom, 1939

**Geographic distribution.** Europe, northern Africa, Russia (Guglielmone and Robbins 2018).

**Natural hosts.** Mammals, primarily hedgehogs and carnivores, also lagomorphs and bovids, birds, and reptiles. Zoonotic on humans (Guglielmone and Robbins 2018).

**Route of infection.** Exposure to larval, nymph, or adult ticks in the environment.

**Site in human host.** Skin.

**Vectored pathogens.** None.

***Haemaphysalisflava* Neumann, 1897**

**Geographic distribution.** East Asia (Guglielmone and Robbins 2018).

**Natural hosts.** Several groups of mammals and birds. Zoonotic on humans (Guglielmone and Robbins 2018).

**Route of infection.** Exposure to larval, nymph, or adult ticks in the environment.

**Site in human host.** Skin.

**Vectored pathogens.***Rickettsiajaponica* (Japanese spotted fever) (Li et al. 2019).

***Haemaphysalisheinrichi* Schülze, 1939**

**Geographic distribution.** Southeast Asia (Guglielmone and Robbins 2018).

**Natural hosts.** Mammals, including bovids, carnivores, rodents, and shrews. Zoonotic on humans (Guglielmone and Robbins 2018).

**Route of infection.** Exposure to larval, nymph, or adult ticks in the environment.

**Site in human host.** Skin.

**Vectored pathogens.** None.

***Haemaphysalishirsuta* Hoogstraal et al., 1966**

**Geographic distribution.** Indonesia (Guglielmone and Robbins 2018).

**Natural hosts.** Mammals, including bovids, pigs, carnivores, and rodents. Zoonotic on humans (Guglielmone and Robbins 2018).

**Route of infection.** Exposure to larval, nymph, or adult ticks in the environment.

**Site in human host.** Skin.

**Vectored pathogens.** None.

***Haemaphysalishoodi* Warburton & Nuttall, 1909**

**Geographic distribution.** Sub-Saharan Africa (Guglielmone and Robbins 2018).

**Natural hosts.** Primarily birds, also mammals including bovids, non-human primates, rodents, carnivores, and lagomorphs. Zoonotic on humans (Guglielmone and Robbins 2018).

**Route of infection.** Exposure to larval, nymph, or adult ticks in the environment.

**Site in human host.** Skin.

**Vectored pathogens.** None.

***Haemaphysalishumerosa* Warburton & Nuttall, 1909**

**Geographic distribution.** Indonesia, Australia (Guglielmone and Robbins 2018).

**Natural hosts.** Primarily marsupials, also monotremes, rodents, and birds. Zoonotic on humans (Guglielmone and Robbins 2018).

**Route of infection.** Exposure to larval, nymph, or adult ticks in the environment.

**Site in human host.** Skin.

**Vectored pathogens.** None.

***Haemaphysalishylobatis* Schülze, 1933**

**Geographic distribution.** Southeast Asia, Japan (Guglielmone and Robbins 2018).

**Natural hosts.** Several groups of mammals and birds. Zoonotic on humans (Guglielmone and Robbins 2018).

**Route of infection.** Exposure to larval, nymph, or adult ticks in the environment.

**Site in human host.** Skin.

**Vectored pathogens.** None.

***Haemaphysalishystricis* Supino, 1897**

*Haemaphysalisnishiyamai* Sugimoto, 1935

*Haemaphysalistrispinosa* Tounamoff, 1941

**Geographic distribution.** Southeast Asia, Japan (Guglielmone and Robbins 2018).

**Natural hosts.** Several groups of mammals and birds. Zoonotic on humans (Guglielmone and Robbins 2018).

**Route of infection.** Exposure to larval, nymph, or adult ticks in the environment.

**Site in human host.** Skin.

**Vectored pathogens.***Rickettsiajaponica* (Japanese spotted fever) (Li et al. 2019).

***Haemaphysalisindoflava* Dhanda & Bhat, 1968**

**Geographic distribution.** Southeast Asia, Japan (Guglielmone and Robbins 2018).

**Natural hosts.** Bovids, pigs, carnivores. Zoonotic on humans (Guglielmone and Robbins 2018).

**Route of infection.** Exposure to larval, nymph, or adult ticks in the environment.

**Site in human host.** Skin.

**Vectored pathogens.** None.

***Haemaphysalisinermis* Birula, 1895**

**Geographic distribution.** Southern Europe, Middle East (Guglielmone and Robbins 2018).

**Natural hosts.** Primarily birds, also rodents, shrews, and reptiles. Zoonotic on humans (Guglielmone and Robbins 2018).

**Route of infection.** Exposure to larval, nymph, or adult ticks in the environment.

**Site in human host.** Skin.

**Vectored pathogens.** None.

***Haemaphysalisintermedia* Warburton & Nuttall, 1909**

**Geographic distribution.** Central Asia (Guglielmone and Robbins 2018).

**Natural hosts.** Several groups of mammals and birds. Zoonotic on humans (Guglielmone and Robbins 2018).

**Route of infection.** Exposure to larval, nymph, or adult ticks in the environment.

**Site in human host.** Skin.

**Vectored pathogens.** None.

***Haemaphysalisjaponica* Warburton, 1908**

*Haemaphysalisjaponicadouglasi* Nuttall & Warburton, 1915

**Geographic distribution.** Southeast Asia, Japan, Russia (Guglielmone and Robbins 2018).

**Natural hosts.** Mammals, including deer, carnivores, lagomorphs, pigs, horses, bovids, and hedgehogs, and birds. Zoonotic on humans (Guglielmone and Robbins 2018).

**Route of infection.** Exposure to larval, nymph, or adult ticks in the environment.

**Site in human host.** Skin.

**Vectored pathogens.** None.

***Haemaphysalisjuxtakochi* Cooley, 1946**

*Haemaphysaliskohlsi* Aragão & Fonseca, 1951

**Geographic distribution.** USA, Central and South America (Guglielmone and Robbins 2018).

**Natural hosts.** Mammals, including deer, peccaries, bovids, horses, carnivores, marsupials, rodents, and lagomorphs, and birds. Zoonotic on humans (Guglielmone and Robbins 2018).

**Route of infection.** Exposure to larval, nymph, or adult ticks in the environment.

**Site in human host.** Skin.

**Vectored pathogens.** None.

***Haemaphysaliskitaokai* Hoogstraal, 1969**

**Geographic distribution.** Southeast Asia, Japan (Guglielmone and Robbins 2018).

**Natural hosts.** Mammals, including bovids, deer, horses, and rodents, and birds. Zoonotic on humans (Guglielmone and Robbins 2018).

**Route of infection.** Exposure to larval, nymph, or adult ticks in the environment.

**Site in human host.** Skin.

**Vectored pathogens.** None.

***Haemaphysaliskoningsbergeri* Warburton & Nuttall, 1909**

**Geographic distribution.** Southeast Asia (Guglielmone and Robbins 2018).

**Natural hosts.** Mammals, primarily carnivores and rodents. Zoonotic on humans (Guglielmone and Robbins 2018).

**Route of infection.** Exposure to larval, nymph, or adult ticks in the environment.

**Site in human host.** Skin.

**Vectored pathogens.** None.

***Haemaphysalislagrangei* Larrousse, 1925**

*Haemaphysalishystricisindochinensis* Phan Trong, 1977

**Geographic distribution.** Southeast Asia (Guglielmone and Robbins 2018).

**Natural hosts.** Several groups of mammals and birds. Zoonotic on humans (Guglielmone and Robbins 2018).

**Route of infection.** Exposure to larval, nymph, or adult ticks in the environment.

**Site in human host.** Skin.

**Vectored pathogens.** None.

***Haemaphysalisleachi* (Audouin, 1826)**

**Geographic distribution.** Africa (Apanaskevich et al. 2007; Guglielmone and Robbins 2018).

**Natural hosts.** Mammals, primarily carnivores, bovids, pigs, non-human primates, hedgehogs, and rodents, and birds. Zoonotic on humans (Apanaskevich et al. 2007; Guglielmone and Robbins 2018).

**Route of infection.** Exposure to larval, nymph, or adult ticks in the environment.

**Site in human host.** Skin.

**Vectored pathogens.***Rickettsiaconorii* (boutonneuse fever) (Apanaskevich et al. 2007).

***Haemaphysalisleporispalustris* Packard, 1869**

**Geographic distribution.** North, Central, and South America (Guglielmone and Robbins 2018; Sánchez-Montes et al. 2020).

**Natural hosts.** Primarily rabbits, also other mammals and birds. Zoonotic on humans (Guglielmone and Robbins 2018).

**Route of infection.** Exposure to larval, nymph, or adult ticks in the environment.

**Site in human host.** Skin.

**Vectored pathogens.** None.

***Haemaphysalislongicornis* Neumann, 1901**

**Geographic distribution.** East Asia, Australia, New Zealand, Pacific Islands, eastern North America (Beard et al. 2018; Guglielmone and Robbins 2018; Wormser et al. 2020).

**Natural hosts.** Mammals, including sheep, deer, and horses, and birds. Zoonotic on humans (Guglielmone and Robbins 2018).

**Route of infection.** Exposure to larval, nymph, or adult ticks in the environment.

**Site in human host.** Skin, ear (Choi et al. 2018).

**Vectored pathogens.** Dabie bandavirus (severe fever with thrombocytopenia syndrome vrus, SFTSV), *Rickettsiajaponica* (Japanese spotted fever) (Li et al. 2019; Sato et al. 2021).

***Haemaphysalismageshimaensis* Saito & Hoogstraal, 1973**

**Geographic distribution.** Southeast Asia, Japan (Guglielmone and Robbins 2018).

**Natural hosts.** Mammals, including bovids, deer, carnivores, pigs, rodents, and birds. Zoonotic on humans (Guglielmone and Robbins 2018).

**Route of infection.** Exposure to larval, nymph, or adult ticks in the environment.

**Site in human host.** Skin.

**Vectored pathogens.** None.

***Haemaphysalismegaspinosa*** Saito, 1969

**Geographic distribution.** Southeast Asia, Japan (Guglielmone and Robbins 2018).

**Natural hosts.** Deer, carnivores, horses. Zoonotic on humans (Guglielmone and Robbins 2018).

**Route of infection.** Exposure to larval, nymph, or adult ticks in the environment.

**Site in human host.** Skin.

**Vectored pathogens.** None.

***Haemaphysalismjoebergi* Warburton, 1926**

**Geographic distribution.** Indonesia (Guglielmone and Robbins 2018).

**Natural hosts.** Deer, bovids. Zoonotic on humans (Guglielmone and Robbins 2018).

**Route of infection.** Exposure to larval, nymph, or adult ticks in the environment.

**Site in human host.** Skin.

**Vectored pathogens.** None.

***Haemaphysalismontgomeryi* Nuttall, 1912**

**Geographic distribution.** Central and Southeast Asia, Japan (Guglielmone and Robbins 2018).

**Natural hosts.** Mammals, including bovids, rodents, camels, deer, horses, and hedgehogs, and birds. Zoonotic on humans (Guglielmone and Robbins 2018).

**Route of infection.** Exposure to larval, nymph, or adult ticks in the environment.

**Site in human host.** Skin.

**Vectored pathogens.** None.

***Haemaphysalisnadchatrami* Hoogstraal et al., 1965**

**Geographic distribution.** Southeast Asia (Guglielmone and Robbins 2018).

**Natural hosts.** Rodents, bovids, deer, pigs, carnivores, horses, tapirs. Zoonotic on humans (Guglielmone and Robbins 2018).

**Route of infection.** Exposure to larval, nymph, or adult ticks in the environment.

**Site in human host.** Skin.

**Vectored pathogens.** None.

***Haemaphysalisnepalensis* Hoogstraal, 1962**

**Geographic distribution.** Central and Southeast Asia (Guglielmone and Robbins 2018).

**Natural hosts.** Bovids, carnivores, rodents. Zoonotic on humans (Guglielmone and Robbins 2018).

**Route of infection.** Exposure to larval, nymph, or adult ticks in the environment.

**Site in human host.** Skin.

**Vectored pathogens.** None.

***Haemaphysalisnovaeguineae* Hirst, 1914**

**Geographic distribution.** Australia, Papua New Guinea (Guglielmone and Robbins 2018).

**Natural hosts.** Mammals, including bovids, pigs, marsupials, carnivores, horses, and rodents, and birds. Zoonotic on humans (Guglielmone and Robbins 2018).

**Route of infection.** Exposure to larval, nymph, or adult ticks in the environment.

**Site in human host.** Skin.

**Vectored pathogens.** None.

***Haemaphysalisobesa* Larrousse, 1925**

**Geographic distribution.** Southeast Asia (Guglielmone and Robbins 2018).

**Natural hosts.** Several groups of mammals. Zoonotic on humans (Guglielmone and Robbins 2018).

**Route of infection.** Exposure to larval, nymph, or adult ticks in the environment.

**Site in human host.** Skin.

**Vectored pathogens.** None.

***Haemaphysalispapuana* Thorell, 1883**

**Geographic distribution.** Southeast Asia (Guglielmone and Robbins 2018).

**Natural hosts.** Mammals, including pigs, deer, musk deer, carnivores, and rodents, and birds. Zoonotic on humans (Guglielmone and Robbins 2018).

**Route of infection.** Exposure to larval, nymph, or adult ticks in the environment.

**Site in human host.** Skin.

**Vectored pathogens.** None.

***Haemaphysalisparaleachi* Camicas et al. 1983**

**Geographic distribution.** Sub-Saharan Africa (Guglielmone and Robbins 2018).

**Natural hosts.** Carnivores, rodents, bovids, non-human primates. Zoonotic on humans (Guglielmone and Robbins 2018).

**Route of infection.** Exposure to larval, nymph, or adult ticks in the environment.

**Site in human host.** Skin.

**Vectored pathogens.** None.

***Haemaphysalisparmata* Neumann, 1905**

**Geographic distribution.** Sub-Saharan Africa (Guglielmone and Robbins 2018).

**Natural hosts.** Mammals, primarily bovids, rarely reptiles and birds. Zoonotic on humans (Guglielmone and Robbins 2018).

**Route of infection.** Exposure to larval, nymph, or adult ticks in the environment.

**Site in human host.** Skin.

**Vectored pathogens.** None.

***Haemaphysalisparva* (Neumann, 1897)**

*Haemaphysalisotophila* Schülze, 1919

**Geographic distribution.** Europe, northern Africa, western Asia (Guglielmone and Robbins 2018).

**Natural hosts.** Mammals, including hedgehogs, carnivores, lagomorphs, rodents, and humans, birds, and reptiles (Guglielmone and Robbins 2018).

**Route of infection.** Exposure to larval, nymph, or adult ticks in the environment.

**Site in human host.** Skin.

**Vectored pathogens.** None.

***Haemaphysalispunctata* Canestrini & Fanzago, 1878**

*Haemaphysalispunctataautumnalis* Schülze, 1919

**Geographic distribution.** Europe, northern Africa, western and central Asia (Guglielmone and Robbins 2018).

**Natural hosts.** Several groups of mammals, including humans, birds, and reptiles (Guglielmone and Robbins 2018).

**Route of infection.** Exposure to larval, nymph, or adult ticks in the environment.

**Site in human host.** Skin.

**Vectored pathogens.** None.

***Haemaphysalisqinghaiensis* Teng, 1980**

**Geographic distribution.** China (Guglielmone and Robbins 2018).

**Natural hosts.** Bovids, horses, lagomorphs. Zoonotic on humans (Guglielmone and Robbins 2018).

**Route of infection.** Exposure to larval, nymph, or adult ticks in the environment.

**Site in human host.** Skin.

**Vectored pathogens.** None.

***Haemaphysalisramachandrai* Dhanda et al., 1970**

**Geographic distribution.** India, Nepal (Guglielmone and Robbins 2018).

**Natural hosts.** Deer, bovids, carnivores. Zoonotic on humans (Guglielmone and Robbins 2018).

**Route of infection.** Exposure to larval, nymph, or adult ticks in the environment.

**Site in human host.** Skin.

**Vectored pathogens.** None.

***Haemaphysalisroubaudi* Toumanoff, 1940**

**Geographic distribution.** Vietnam (Guglielmone and Robbins 2018).

**Natural hosts.** Deer. Zoonotic on humans (Guglielmone and Robbins 2018).

**Route of infection.** Exposure to larval, nymph, or adult ticks in the environment.

**Site in human host.** Skin.

**Vectored pathogens.** None.

***Haemaphysalissemermis* Neumann, 1901**

**Geographic distribution.** Southeast Asia (Guglielmone and Robbins 2018).

**Natural hosts.** Chevrotains, deer, pigs, carnivores, tapirs, rodents, tree shrews. Zoonotic on humans (Guglielmone and Robbins 2018).

**Route of infection.** Exposure to larval, nymph, or adult ticks in the environment.

**Site in human host.** Skin.

**Vectored pathogens.** None.

***Haemaphysalisshimoga* Trapido & Hoogstraal, 1964**

*Haemaphysaliscornigeravietnama* Phan Trong, 1977

**Geographic distribution.** Southeast Asia (Guglielmone and Robbins 2018).

**Natural hosts.** Mammals, including bovids, pigs, deer, and rodents. Zoonotic on humans (Guglielmone and Robbins 2018).

**Route of infection.** Exposure to larval, nymph, or adult ticks in the environment.

**Site in human host.** Skin.

**Vectored pathogens.** None.

***Haemaphysalissilacea* Robinson, 1912**

**Geographic distribution.** Sub-Saharan Africa (Guglielmone and Robbins 2018)

**Natural hosts.** Mammals, including bovids, elephant shrews, rhinoceroses, carnivores, lagomorphs, and rodents, and birds. Zoonotic on humans (Guglielmone and Robbins 2018).

**Route of infection.** Exposure to larval, nymph, or adult ticks in the environment.

**Site in human host.** Skin.

**Vectored pathogens.** None.

***Haemaphysalisspinigera* Neumann, 1897**

**Geographic distribution.** Southeast Asia (Guglielmone and Robbins 2018).

**Natural hosts.** Several groups of mammals, including humans, and birds (Guglielmone and Robbins 2018).

**Route of infection.** Exposure to larval, nymph, or adult ticks in the environment.

**Site in human host.** Skin.

**Vectored pathogens.** Kyasanur Forest Disease virus (Varma et al. 1960; Sharma et al. 2019).

***Haemaphysalissulcata* Canestrini & Fanzago, 1878**

*Haemaphysalischolodkovskyi* Olenev. 1928

**Geographic distribution.** Europe, North Africa, Asia (Guglielmone and Robbins 2018).

**Natural hosts.** Mammals, primarily bovids, but also carnivores, rodents, lagomorphs, bats, hedgehogs, and humans, and reptiles (Guglielmone and Robbins 2018).

**Route of infection.** Exposure to larval, nymph, or adult ticks in the environment.

**Site in human host.** Skin.

**Vectored pathogens.** None.

***Haemaphysalisturturis* Nuttall & Warburton, 1915**

**Geographic distribution.** India, Sri Lanka (Guglielmone and Robbins 2018).

**Natural hosts.** Several groups of mammals, birds, and reptiles. Zoonotic on humans (Guglielmone and Robbins 2018).

**Route of infection.** Exposure to larval, nymph, or adult ticks in the environment.

**Site in human host.** Skin.

**Vectored pathogens.** Kyasanur Forest disease virus (Sharma et al. 2019).

***Haemaphysaliswellingtoni* Nuttall & Warburton, 1908**

**Geographic distribution.** Southeast Asia (Guglielmone and Robbins 2018).

**Natural hosts.** Primarily birds, occasionally mammals. Zoonotic on humans (Guglielmone and Robbins 2018).

**Route of infection.** Exposure to larval, nymph, or adult ticks in the environment.

**Site in human host.** Skin.

**Vectored pathogens.** Kyasanur Forest disease virus (Sharma et al. 2019).

**Genus *Hyalomma* Koch, 1844**

***Hyalommaaegyptium* (Linnaeus, 1758)**

**Geographic distribution.** Europe, North Africa, Middle East, Central Asia (Guglielmone and Robbins 2018).

**Natural hosts.** Primarily tortoises, but also other reptiles and mammals. Zoonotic on humans (Guglielmone and Robbins 2018).

**Route of infection.** Exposure to larval, nymph, or adult ticks in the environment.

**Site in human host.** Skin.

**Vectored pathogens.***Borreliaturcica* (tick-borne relapsing fever) (Güner et al. 2004).

***Hyalommaalbiparmatum* Schülze, 1919**

**Geographic distribution.** East Africa (Apanaskevich and Horak 2008; Guglielmone and Robbins 2018).

**Natural hosts.** Many mammals, primarily wild ungulates, also lagomorphs and birds. Zoonotic on humans (Apanaskevich and Horak 2008; Guglielmone and Robbins 2018).

**Route of infection.** Exposure to larval, nymph, or adult ticks in the environment.

**Site in human host.** Skin.

**Vectored pathogens.***Rickettsiaconorii* (boutonneuse fever) (Apanaskevich and Horak 2008).

***Hyalommaanatolicum* Koch, 1844**

**Geographic distribution.** North Africa, Middle East, Central Asia (Vatansever 2017a; Guglielmone and Robbins 2018).

**Natural hosts.** Mammals, including cattle, horses, camels, sheep, goats, and humans, and birds (Guglielmone and Robbins 2018).

**Route of infection.** Exposure to larval, nymph, or adult ticks in the environment.

**Site in human host.** Skin.

**Vectored pathogens.** Crimean-Congo hemorrhagic fever virus (Kassari et al. 2020).

***Hyalommaasiaticum* Schülze & Schlottke, 1930**

*Hyalommaasiaticumkozlovi* Olenev, 1931

**Geographic distribution.** Central Asia, Middle East (Guglielmone and Robbins 2018; Vatansever 2017b).

**Natural hosts.** Mammals, including bovids, camels, rodents, lagomorphs, and hedgehogs, birds, and reptiles. Zoonotic on humans (Guglielmone and Robbins 2018).

**Route of infection.** Exposure to larval, nymph, or adult ticks in the environment.

**Site in human host.** Skin.

**Vectored pathogens.***Rickettsiasibiricamongolitimonae*, *Coxiellaburnetti* (Q fever), and possibly Crimean-Congo Hemorrhagic Fever virus (Ramos et al. 2013; Batu et al. 2020; Kassari et al. 2020).

***Hyalommabrevipunctatum* Sharif, 1928**

**Geographic distribution.** Southeast and Central Asia (Guglielmone and Robbins 2018).

**Natural hosts.** Mammals, including bovids, deer, camels, horses, rodents, carnivores, shrews, and birds. Zoonotic on humans (Guglielmone and Robbins 2018).

**Route of infection.** Exposure to larval, nymph, or adult ticks in the environment.

**Site in human host.** Skin.

**Vectored pathogens.** None.

***Hyalommadromedarii* Koch, 1844**

*Ixodescamelinus* Fischer von Waldheim, 1823

*Ixodesarenicola* Eichwald, 1830

*Ixodestrilineatus* Lucas, 1844

*Ixodescinctus* Lucas, 1844

*Hyalommayakimovi* Olenev, 1931

*Hyalommayakimovipersiacum* Olenev, 1931

*Hyalommadelphy* Schülze and Gossel in Schülze, 1936

**Geographic distribution.** Africa, Middle East, Central and Southeast Asia (Guglielmone and Robbins 2018).

**Natural hosts.** Primarily camels, but also other mammals, birds, and reptiles. Zoonotic on humans (Guglielmone and Robbins 2018).

**Route of infection.** Exposure to larval, nymph, or adult ticks in the environment.

**Site in human host.** Skin.

**Vectored pathogens.** Crimean-Congo hemorrhagic fever virus (Champour et al. 2016).

***Hyalommaexcavatum* Koch, 1844**

**Geographic distribution.** North Africa, Mediterranean Europe, Middle East, Central Asia (Guglielmone and Robbins 2018).

**Natural hosts.** Mammals, including bovids, camels, lagomorphs, rodents, hedgehogs, and humans, birds, and reptiles (Guglielmone and Robbins 2018).

**Route of infection.** Exposure to larval, nymph, or adult ticks in the environment.

**Site in human host.** Skin.

**Vectored pathogens.** None.

***Hyalommaglabrum* Delpy, 1949**

**Geographic distribution.** South Africa (Guglielmone and Robbins 2018).

**Natural hosts.** Immature stages on lagomorphs, carnivores, hyrax, rodents, and birds. Adults on bovids, occasionally carnivores. Zoonotic on humans (Guglielmone and Robbins 2018).

**Route of infection.** Exposure to larval, nymph, or adult ticks in the environment.

**Site in human host.** Skin.

**Vectored pathogens.** None.

***Hyalommahussaini* Sharif, 1928**

**Geographic distribution.** India, Pakistan, Myanmar (Guglielmone and Robbins 2018).

**Natural hosts.** Immature stages on rodents and shrews. Adults on bovids, camels, pigs, carnivores, and horses. Zoonotic on humans (Guglielmone and Robbins 2018).

**Route of infection.** Exposure to larval, nymph, or adult ticks in the environment.

**Site in human host.** Skin.

**Vectored pathogens.** None.

***Hyalommaimpeltatum* Schülze & Schlottke, 1930**

**Geographic distribution.** Africa, Middle East, Central Asia (Guglielmone and Robbins 2018).

**Natural hosts.** Mammals, including bovids, camels, horses, rhinoceroses, carnivores, and rodents, birds, and reptiles. Zoonotic on humans (Guglielmone and Robbins 2018).

**Route of infection.** Exposure to larval, nymph, or adult ticks in the environment.

**Site in human host.** Skin.

**Vectored pathogens.** None.

***Hyalmonnaisaaci* Sharif, 1928**

**Geographic distribution.** Central and Southeast Asia (Guglielmone and Robbins 2018).

**Natural hosts.** Immature stages on lagomorphs, deer, carnivores, and birds. Adults primarily on bovids. Zoonotic on humans (Guglielmone and Robbins 2018).

**Route of infection.** Exposure to larval, nymph, or adult ticks in the environment.

**Site in human host.** Skin.

**Vectored pathogens.** None.

***Hyalommalustanicum* Koch, 1844**

**Geographic distribution.** Mediterranean Europe and Africa (Guglielmone and Robbins 2018).

**Natural hosts.** Mammals, including bovids and lagomorphs, and birds. Zoonotic on humans (Guglielmone and Robbins 2018).

**Route of infection.** Exposure to larval, nymph, or adult ticks in the environment.

**Site in human host.** Skin.

**Vectored pathogens.** None.

***Hyalommamarginatum* Koch, 1844**

**Geographic distribution.** Europe, North Africa, Middle East, Central Asia (Estrada-Peña et al. 2012; Guglielmone and Robbins 2018).

**Natural hosts.** Several groups of mammals, including humans, and birds. Immature stages are primarily on small mammals and birds. Adults primarily on ungulates (Guglielmone and Robbins 2018).

**Route of infection.** Exposure to larval, nymph, or adult ticks in the environment.

**Site in human host**: Skin.

**Vectored pathogens.** Crimean-Congo hemorrhagic fever virus (Gargili et al. 2013; Kassari et al. 2020).

***Hyalommarufipes* Koch, 1844**

**Geographic distribution.** Africa, Middle East (Guglielmone and Robbins 2018).

**Natural hosts.** Several groups of mammals and birds. Immature stages are primarily on birds and lagomorphs. Adults are primarily on bovids. Zoonotic on humans (Guglielmone and Robbins 2018).

**Route of infection.** Exposure to larval, nymph, or adult ticks in the environment.

**Site in human host.** Skin.

**Vectored pathogens.** Crimean-Congo hemorrhagic fever virus (Hoogstraal 1979).

***Hyalommaschulzei* Olenev, 1931**

**Geographic distribution.** Middle East, Central Asia (Guglielmone and Robbins 2018).

**Natural hosts.** Immature stages primarily on rodents, hedgehogs, and lagomorphs. Adults primarily on camels, occasionally bovids. Zoonotic on humans (Guglielmone and Robbins 2018).

**Route of infection.** Exposure to larval, nymph, or adult ticks in the environment.

**Site in human host.** Skin.

**Vectored pathogens.** None.

***Hyalommascupense* Schülze, 1919**

*Hyalommadetritum* Schülze, 1919

*Hyalommadetritumdardanicum* Schülze & Schlottke, 1930

*Hyalommauralense* Schülze & Schlottke, 1930

*Hyalommavolgense* Schülze & Schlottke, 1930

**Geographic distribution.** Europe, North Africa, Middle East, Central and East Asia (Guglielmone and Robbins 2018).

**Natural hosts.** Bovids, camels, horses, pigs, deer, and humans (Guglielmone and Robbins 2018).

**Route of infection.** Exposure to larval, nymph, or adult ticks in the environment.

**Site in human host.** Skin.

**Vectored pathogens.** None.

***Hyalommatruncatum* Koch, 1844**

*Hyalommatransiens* Schülze, 1919

**Geographic distribution.** Africa, Saudi Arabia, Yemen (Apanaskevich and Horak 2008; Guglielmone and Robbins 2018).

**Natural hosts.** Many groups of mammals, birds, and reptiles. Immature stages primarily on rodents and lagomorphs. Adults primarily on domestic and wild ungulates. Zoonotic on humans (Apanaskevich and Horak 2008; Guglielmone and Robbins 2018).

**Route of infection.** Exposure to larval, nymph, or adult ticks in the environment

**Site in human host.** Skin

**Vectored pathogens.** Crimean-Congo hemorrhagic fever virus, *Rickettsiaconnorii* (boutonneuse fever), *Coxiellaburnetii* (Q fever) (Apanaskevich and Horak 2008).

***Hyalommaturanicum* Pomerantzev, 1946**

**Geographic distribution.** North Africa, Middle East, Central and East Asia (Guglielmone and Robbins 2018).

**Natural hosts.** Mammals, including bovids, lagomorphs, camels, pigs, horses, humans, and birds (Guglielmone and Robbins 2018).

**Route of infection.** Exposure to larval, nymph, or adult ticks in the environment.

**Site in human host.** Skin.

**Vectored pathogens.** None.

**Genus *Ixodes* Latreille, 1795**

***Ixodesacuminatus* Neumann, 1901**

**Geographic distribution.** Europe, Middle East (Guglielmone and Robbins 2018).

**Natural hosts.** Mammals, primarily rodents, but also other mammals and birds. Zoonotic on humans (Guglielmone and Robbins 2018).

**Route of infection.** Exposure to larval, nymph, or adult ticks in the environment.

**Site in human host.** Skin.

**Vectored pathogens.** None.

***Ixodesacutitarsus* (Karsch, 1880)**

*Ixodesgigas* Warburton, 1910

**Geographic distribution.** Southeast Asia, Japan, India (Guglielmone and Robbins 2018).

**Natural hosts.** Immature stages are typically on rodents; adults on ungulates. Zoonotic on humans (Guglielmone and Robbins 2018).

**Route of infection.** Exposure to larval, nymph, or adult ticks in the environment.

**Site in human host.** Skin.

**Vectored pathogens.** None.

***Ixodesangustus* Neumann, 1899**

**Geographic distribution.** North America, Russia, Japan (Spencer 1963; Guglielmone and Robbins 2018).

**Natural hosts.** Mammals, primarily rodents, but also other mammals and birds. Zoonotic on humans (Spencer 1963; Guglielmone and Robbins 2018).

**Route of infection.** Exposure to larval, nymph, or adult ticks in the environment.

**Site in human host.** Skin.

**Vectored pathogens.** None.

***Ixodesapronophorus* Schülze, 1924**

**Geographic distribution.** Europe, Asia (Guglielmone and Robbins 2018).

**Natural hosts.** Mammals, primarily rodents, but also other mammals, reptiles, and birds. Zoonotic on humans (Guglielmone and Robbins 2018).

**Route of infection.** Exposure to larval, nymph, or adult ticks in the environment.

**Site in human host.** Skin.

**Vectored pathogens.** None.

***Ixodesasanumai* Kitaoka, 1973**

**Geographic distribution.** Japan (Okono 2010).

**Natural hosts.** Reptiles, rarely mammals and birds. Zoonotic on humans (Okono 2010).

**Route of infection.** Exposure to larval, nymph, or adult ticks in the environment.

**Site in human host.** Skin.

**Vectored pathogens.** None.

***Ixodesaustraliensis* Neumann, 1904**

**Geographic distribution.** Australia (Kwak 2018).

**Natural hosts.** Marsupials, dogs, bovids. Zoonotic on humans (Kwak 2018).

**Route of infection.** Exposure to larval, nymph, or adult ticks in the environment.

**Site in human host.** Skin.

**Vectored pathogens.** None.

***Ixodesbaergi* Cooley & Kohls, 1942**

**Geographic distribution.** North America (Walker et al. 1998).

**Natural hosts.** Passerine birds. Zoonotic on humans (Walker et al. 1998).

**Route of infection.** Exposure to larval, nymph, or adult ticks in the environment.

**Site in human host.** Skin.

**Vectored pathogens.** None.

***Ixodesbanksi* Bishopp, 1911**

**Geographic distribution.** North America (Guglielmone and Robbins 2018).

**Natural hosts.** Rodents, carnivores. Zoonotic on humans (Guglielmone and Robbins 2018).

**Route of infection.** Exposure to larval, nymph, or adult ticks in the environment.

**Site in human host.** Skin.

**Vectored pathogens.** None.

***Ixodesboliviensis* Neumann, 1904**

*Ixodesbicornis* Neumann, 1906

**Geographic distribution.** Central and South America (Guglielmone and Robbins 2018).

**Natural hosts.** Several groups of mammals and birds. Zoonotic on humans (Guglielmone and Robbins 2018).

**Route of infection.** Exposure to larval, nymph, or adult ticks in the environment.

**Site in human host.** Skin.

**Vectored pathogens.** None.

***Ixodesbrunneus* Koch, 1844**

*Ixodesricinuscalifornicus* Banks, 1908

**Geographic distribution.** Western Hemisphere (Guglielmone and Robbins 2018).

**Natural hosts.** Birds. Zoonotic on humans (Guglielmone and Robbins 2018).

**Route of infection.** Exposure to larval, nymph, or adult ticks in the environment.

**Site in human host.** Skin.

**Vectored pathogens.** None.

***Ixodescanisuga* Johnston, 1849**

**Geographic distribution.** Europe (Guglielmone and Robbins 2018).

**Natural hosts.** Carnivores; immature stages occasionally on birds. Zoonotic on humans (Guglielmone and Robbins 2018).

**Route of infection.** Exposure to larval, nymph, or adult ticks in the environment.

**Site in human host.** Skin.

**Vectored pathogens.** None.

***Ixodescavipalpus* Nuttall & Warburton, 1908**

*Ixodesrubicunduslimbatus* Neumann, 1908

**Geographic distribution.** Sub-Saharan Africa (Guglielmone and Robbins 2018).

**Natural hosts.** Ungulates, primarily cattle. Zoonotic on humans (Guglielmone and Robbins 2018).

**Route of infection.** Exposure to larval, nymph, or adult ticks in the environment.

**Site in human host.** Skin.

**Vectored pathogens.** None.

***Ixodescolumnae* Takada & Fujita, 1992**

**Geographic distribution.** Japan (Takada and Fujita 1992).

**Natural hosts.** Rodents, birds. Zoonotic on humans (Takada and Fujita 1992).

**Route of infection.** Exposure to larval, nymph, or adult ticks in the environment.

**Site in human host.** Skin.

**Vectored pathogens.** None.

***Ixodesconfusus* Roberts, 1960**

**Geographic distribution.** Australia, Papua New Guinea (Roberts 1970).

**Natural hosts.** Ungulates, equids, marsupials. Zoonotic on humans (Roberts 1970).

**Route of infection.** Exposure to larval, nymph, or adult ticks in the environment.

**Site in human host.** Skin.

**Vectored pathogens.** None.

***Ixodescookei* Packard, 1869**

*Ixodescruciarius* Fitch, 1872

**Geographic distribution.** North America (Guglielmone and Robbins 2018).

**Natural hosts.** Several groups of mammals, including humans, and birds (Guglielmone and Robbins 2018).

**Route of infection.** Exposure to larval, nymph, or adult ticks in the environment.

**Site in human host.** Skin.

**Vectored pathogens.** Powassan virus lineage I (Khan et al. 2019).

***Ixodescornuatus* Roberts, 1960**

**Geographic distribution.** Australia (Guglielmone and Robbins 2018).

**Natural hosts.** Mammals, primarily carnivores and rodents, and birds. Zoonotic on humans (Guglielmone and Robbins 2018).

**Route of infection.** Exposure to larval, nymph, or adult ticks in the environment.

**Site in human host.** Skin.

**Vectored pathogens.** None.

***Ixodescrenulatus* Koch, 1844**

**Geographic distribution.** Europe, Asia (Guglielmone and Robbins 2018).

**Natural hosts.** Mammals, primarily rodents, carnivores, lagomorphs. Zoonotic on humans (Guglielmone and Robbins 2018).

**Route of infection.** Exposure to larval, nymph, or adult ticks in the environment.

**Site in human host.** Skin.

**Vectored pathogens.** None.

***Ixodescumulatimpunctatus* Schülze, 1943**

*Ixodespseudorasus* Arthur & Burrow, 1957

**Geographic distribution.** Sub-Saharan Africa (Guglielmone and Robbins 2018).

**Natural hosts.** Several groups of mammals and birds. Zoonotic on humans (Guglielmone and Robbins 2018).

**Route of infection.** Exposure to larval, nymph, or adult ticks in the environment.

**Site in human host.** Skin.

**Vectored pathogens.** None.

***Ixodesdentatus* Marx in Neumann, 1899**

**Geographic distribution.** North America (Guglielmone and Robbins 2018).

**Natural hosts.** Immature stages on a variety of mammals and birds; adults on mammals, primarily lagomorphs and carnivores. Zoonotic on humans (Guglielmone and Robbins 2018).

**Route of infection.** Exposure to larval, nymph, or adult ticks in the environment.

**Site in human host.** Skin.

**Vectored pathogens.** None.

***Ixodeseichhorni* Nuttall, 1916**

**Geographic distribution.** Southeast Asia, Australia, Pacific Islands (Guglielmone and Robbins 2018).

**Natural hosts.** Birds. Zoonotic on humans (Guglielmone and Robbins 2018).

**Route of infection.** Exposure to larval, nymph, or adult ticks in the environment.

**Site in human host.** Skin.

**Vectored pathogens.** None.

***Ixodesfecialis* Warburton & Nuttall, 1909**

**Geographic distribution.** Australia, Papua New Guinea (Domrow and Derrick 1964).

**Natural hosts.** Marsupials. Zoonotic on humans (Domrow and Derrick 1964).

**Route of infection.** Exposure to larval, nymph, or adult ticks in the environment

**Site in human host.** Skin

**Vectored pathogens.** None

***Ixodesfestai* Rondelli, 1926**

**Geographic distribution.** Southern Europe, northern Africa (Guglielmone and Robbins 2018).

**Natural hosts.** Birds. Zoonotic on humans (Guglielmone and Robbins 2018).

**Route of infection.** Exposure to larval, nymph, or adult ticks in the environment.

**Site in human host.** Skin.

**Vectored pathogens.** None.

***Ixodesfrontalis* (Panzer, 1798)**

**Geographic distribution.** Europe, Middle East (Guglielmone and Robbins 2018).

**Natural hosts.** Birds. Zoonotic on humans (Guglielmone and Robbins 2018).

**Route of infection.** Exposure to larval, nymph, or adult ticks in the environment.

**Site in human host.** Skin.

**Vectored pathogens.** None.

***Ixodesgibbosus* Nuttall, 1916**

**Geographic distribution.** Southern, eastern Europe, Middle East (Guglielmone and Robbins 2018).

**Natural hosts.** Ungulates, primarily bovids, rarely birds. Zoonotic on humans (Guglielmone and Robbins 2018).

**Route of infection.** Exposure to larval, nymph, or adult ticks in the environment.

**Site in human host.** Skin.

**Vectored pathogens.** None.

***Ixodesgranulatus* Supino, 1897**

**Geographic distribution.** Southeast Asia (Guglielmone and Robbins 2018).

**Natural hosts.** Mammals, birds, reptiles. Zoonotic on humans (Guglielmone and Robbins 2018).

**Route of infection.** Exposure to larval, nymph, or adult ticks in the environment.

**Site in human host.** Skin.

**Vectored pathogens.** None.

***Ixodeshexagonus* Leach, 1815**

**Geographic distribution.** Europe (Guglielmone and Robbins 2018).

**Natural hosts.** Mammals, primarily hedgehogs, also birds. Zoonotic on humans (Guglielmone and Robbins 2018).

**Route of infection.** Exposure to larval, nymph, or adult ticks in the environment.

**Site in human host.** Skin.

**Vectored pathogens.** None.

***Ixodesholocyclus* Neumann, 1899**

**Geographic distribution.** East coastal Australia (Guglielmone and Robbins 2018).

**Natural hosts.** Marsupials, primarily bandicoots, also livestock, cats, dogs, and humans, and birds (Guglielmone and Robbins 2018).

**Route of infection.** Exposure to larval, nymph, or adult ticks in the environment.

**Site in human host.** Skin.

**Vectored pathogens.***Rickettsiaaustralis* (Queensland tick typhus); also implicated in tick paralysis (Hall-Mendelin et al. 2011; Stewart et al. 2017).

***Ixodeskaschmiricus* Pomerantsev, 1948**

**Geographic distribution.** Central and East Asia (Guglielmone and Robbins 2018).

**Natural hosts.** Mammals, primarily bovids, carnivores, rodents. Zoonotic on humans (Guglielmone and Robbins 2018).

**Route of infection.** Exposure to larval, nymph, or adult ticks in the environment.

**Site in human host.** Skin.

**Vectored pathogens.** None.

***Ixodeskazakstani* Olenev & Sorokoumov, 1934**

**Geographic distribution.** Central Asia (Guglielmone and Robbins 2018).

**Natural hosts.** Several mammals and birds. Zoonotic on humans (Guglielmone and Robbins 2018).

**Route of infection.** Exposure to larval, nymph, or adult ticks in the environment.

**Site in human host.** Skin.

**Vectored pathogens.** None.

***Ixodeskingi* Bishopp, 1911**

**Geographic distribution.** North America (Guglielmone and Robbins 2018).

**Natural hosts.** Mammals, including carnivores, rodents, lagomorphs. Zoonotic on humans (Guglielmone and Robbins 2018).

**Route of infection.** Exposure to larval, nymph, or adult ticks in the environment.

**Site in human host.** Skin.

**Vectored pathogens.** None.

***Ixodeskohlsi* Arthur, 1955**

**Geographic distribution.** Australia (Roberts 1970).

**Natural hosts.** Birds. Zoonotic on humans (Roberts 1970).

**Route of infection.** Exposure to larval, nymph, or adult ticks in the environment.

**Site in human host.** Skin.

**Vectored pathogens.** None.

***Ixodeslaguri* Olenev, 1929**

**Geographic distribution.** Central and Eastern Europe, Asia (Guglielmone and Robbins 2018).

**Natural hosts.** Mammals, primarily rodents, lagomorphs, shrews, hedgehogs, carnivores. Zoonotic on humans (Guglielmone and Robbins 2018).

**Route of infection.** Exposure to larval, nymph, or adult ticks in the environment.

**Site in human host.** Skin.

**Vectored pathogens.** None.

***Ixodesmarxi* Banks, 1908**

**Geographic distribution.** North America (Guglielmone and Robbins 2018).

**Natural hosts.** Mammals, primarily rodents; also birds. Zoonotic on humans (Guglielmone and Robbins 2018).

**Route of infection.** Exposure to larval, nymph, or adult ticks in the environment.

**Site in human host.** Skin.

**Vectored pathogens.** Powassan virus lineage I (Khan et al. 2019).

***Ixodesmonospinosus* Saito, 1968**

**Geographic distribution.** Japan (Guglielmone and Robbins 2018).

**Natural hosts.** Mammals, including bovids, deer, rodents, carnivores, shrews. Zoonotic on humans (Guglielmone and Robbins 2018).

**Route of infection.** Exposure to larval, nymph, or adult ticks in the environment.

**Site in human host.** Skin.

**Vectored pathogens.** None.

***Ixodesmuniensis* Arthur & Burrow, 1957**

**Geographic distribution.** Sub-Saharan Africa (Guglielmone and Robbins 2018).

**Natural hosts.** Mammals, including bovids, pigs, giraffes, hyrax, carnivores, rodents. Zoonotic on humans (Guglielmone and Robbins 2018).

**Route of infection.** Exposure to larval, nymph, or adult ticks in the environment.

**Site in human host.** Skin.

**Vectored pathogens.** None.

***Ixodesmuris* Bishopp & Smith, 1937**

**Geographic distribution.** North America (Guglielmone and Robbins 2018).

**Natural hosts.** Mammals, primarily rodents. Zoonotic on humans (Guglielmone and Robbins 2018).

**Route of infection.** Exposure to larval, nymph, or adult ticks in the environment.

**Site in human host.** Skin.

**Vectored pathogens.** None.

***Ixodesmyrmecobii* Roberts, 1962**

**Geographic distribution.** Australia (Guglielmone and Robbins 2018).

**Natural hosts.** Marsupials, rarely other mammals, birds. Zoonotic on humans (Guglielmone and Robbins 2018).

**Route of infection.** Exposure to larval, nymph, or adult ticks in the environment.

**Site in human host.** Skin.

**Vectored pathogens.** None.

***Ixodesnipponensis* Kitaoka & Saito, 1967**

**Geographic distribution.** Southeast Asia, Japan, Russia (Guglielmone and Robbins 2018).

**Natural hosts.** Mammals, primarily carnivores, bovids, deer, lagomorphs. Zoonotic on humans (Guglielmone and Robbins 2018).

**Route of infection.** Exposure to larval, nymph, or adult ticks in the environment.

**Site in human host.** Skin.

**Vectored pathogens.** None.

***Ixodesovatus* Neumann, 1899**

*Ixodesjaponensis* Neumann, 1904

*Ixodesfrequens* Ogura & Takada, 1927

*Ixodescarinatus* Kishida, 1930

*Ixodeslindbergi* Santos Dias, 1959

**Geographic distribution.** Central, Eastern, Southeastern Asia (Guglielmone and Robbins 2018).

**Natural hosts.** Mammals, including rodents, lagomorphs, carnivores, shrews, humans, and birds (Guglielmone and Robbins 2018).

**Route of infection.** Exposure to larval, nymph, or adult ticks in the environment.

**Site in human host.** Skin, ear (Iwasaki et al. 2007).

**Vectored pathogens.***Rickettsiajaponica* (Japanese spotted fever) (Li et al. 2019).

***Ixodespacificus* Cooley & Kohls, 1943**

*Ixodescalifornicus* Banks, 1904

**Geographic distribution.** Western North America (Padgett and Lane 2001; Guglielmone and Robbins 2018).

**Natural hosts.** Small mammals, birds, and reptiles. Zoonotic on humans (Padgett and Lane 2001; Guglielmone and Robbins 2018).

**Route of infection.** Exposure to larval, nymph, or adult ticks in the environment.

**Site in human host.** Skin.

**Vectored pathogens.***Borreliaburgdorferi* (Lyme disease), *B.miyamotoi* (tick-borne relapsing fever), *Anaplasmaphagocytophilum* (human granulocytic anaplasmosis) (Padgett and Lane 2001).

***Ixodespararicinus* Keirans & Clifford in Keirans et al. 1985**

**Geographic distribution.** Argentina, Colombia, Peru (Saracho-Bottero et al. 2018).

**Natural hosts.** Immature stages are primarily on small mammals and birds. Adults are on ungulates, including bovids, peccaries, and deer. Zoonotic on humans (Saracho-Bottero et al. 2018).

**Route of infection.** Exposure to larval, nymph, or adult ticks in the environment.

**Site in human host.** Skin.

**Vectored pathogens.** None.

***Ixodespavlovskyi* Pomerantzev, 1946**

**Geographic distribution.** Central Asia, Russia, Japan (Guglielmone and Robbins 2018).

**Natural hosts.** Birds and mammals, including rodents, lagomorphs, carnivores, hedgehogs, shrews, and humans (Guglielmone and Robbins 2018).

**Route of infection.** Exposure to larval, nymph, or adult ticks in the environment.

**Site in human host.** Skin.

**Vectored pathogens.** None.

***Ixodespersulcatus* (Schülze, 1930)**

**Geographic distribution.** Europe, Northern Asia (Guglielmone and Robbins 2018).

**Natural hosts.** Mammals, including dogs, deer, rodents, lagomorphs, and humans, and ground-nesting birds (Guglielmone and Robbins 2018).

**Route of infection.** Exposure to larval, nymph, or adult ticks in the environment.

**Site in human host.** Skin, ear (Kogoashiwa et al. 2009),

**Vectored pathogens.***Borreliamiyamotoi* (tick-borne relapsing fever), *B.garinii*, tick-borne encephalitis viruses (TBE) (Jaenson et al. 2016).

***Ixodespetauristae* Warburton, 1933**

**Geographic distribution.** India, Sri Lanka (Guglielmone and Robbins 2018).

**Natural hosts.** Mammals, primarily rodents. Zoonotic on humans (Guglielmone and Robbins 2018).

**Route of infection.** Exposure to larval, nymph, or adult ticks in the environment.

**Site in human host.** Skin.

**Vectored pathogens.** None.

***Ixodespilosus* Koch, 1844**

**Geographic distribution.** Southern Africa (Guglielmone and Robbins 2018).

**Natural hosts.** Mammals, primarily ungulates. Zoonotic on humans (Guglielmone and Robbins 2018).

**Route of infection.** Exposure to larval, nymph, or adult ticks in the environment.

**Site in human host.** Skin.

**Vectored pathogens.** None.

***Ixodesrageaui* Arthur, 1958**

**Geographic distribution.** Sub-Saharan Africa (Guglielmone and Robbins 2018).

**Natural hosts.** Non-human primates. Zoonotic on humans (Guglielmone and Robbins 2018).

**Route of infection.** Exposure to larval, nymph, or adult ticks in the environment.

**Site in human host.** Skin.

**Vectored pathogens.** None.

***Ixodesrasus* Neumann, 1899**

**Geographic distribution.** Sub-Saharan Africa (Guglielmone and Robbins 2018).

**Natural hosts.** Mammals, including ungulates, carnivores, rodents, and hyrax, and birds, Zoonotic on humans (Guglielmone and Robbins 2018).

**Route of infection.** Exposure to larval, nymph, or adult ticks in the environment.

**Site in human host.** Skin.

**Vectored pathogens.** None.

***Ixodesredikorzevi* Olenev, 1927**

*Ixodestheodori* Warburton, 1927

**Geographic distribution.** Europe, North Africa, Middle East, Asia (Guglielmone and Robbins 2018).

**Natural hosts.** Mammals, including rodents, hedgehogs, lagomorphs, and carnivores, birds, and reptiles. Zoonotic on humans (Guglielmone and Robbins 2018).

**Route of infection.** Exposure to larval, nymph, or adult ticks in the environment.

**Site in human host.** Skin.

**Vectored pathogens.** None.

***Ixodesricinus* (Linnaeus, 1758)**

*Acarusreduvius* Linnaeus, 1758

**Geographic distribution.** Europe, North Africa, Middle East, northern Asia (Guglielmone and Robbins 2018).

**Natural hosts.** Many mammals, including humans, and birds. Immature stages are typically on small mammals, reptiles, and birds. Adults are on ungulates and dogs (Guglielmone and Robbins 2018).

**Route of infection.** Exposure to larval, nymph, or adult ticks in the environment.

**Site in human host.** Skin.

**Vectored pathogens.***Borreliaburgdorferi* sensu lato (Lyme disease), *B.miyamotoi* (tick-borne relapsing fever), *Coxiellaburnetii* (Q fever), *Babesiadivergens* (babesiosis), tick-borne encephalitis viruses (TBE), louping ill virus (Davidson et al. 1991; Gern 2005; Rizzoli et al. 2014).

***Ixodesrubicundus* Neumann, 1904**

**Geographic distribution.** South Africa (Guglielmone and Robbins 2018)y.

**Natural hosts.** Mammals, including ungulates, rodents, lagomorphs, and carnivores. Zoonotic on humans (Guglielmone and Robbins 2018).

**Route of infection.** Exposure to larval, nymph, or adult ticks in the environment.

**Site in human host.** Skin.

**Vectored pathogens.** None.

***Ixodesrugosus* Bishopp, 1911**

**Geographic distribution.** North America (Guglielmone and Robbins 2018).

**Natural hosts.** Mammals, primarily carnivores, but also opossums and rodents. Zoonotic on humans (Guglielmone and Robbins 2018).

**Route of infection.** Exposure to larval, nymph, or adult ticks in the environment.

**Site in human host.** Skin.

**Vectored pathogens.** None.

***Ixodesscapularis* Say, 1821**

*Ixodesozarkus* Cooley, 1944

*Ixodesdammini* Spielman et al., 1979

**Geographic distribution.** Eastern and Midwestern North America (Guglielmone and Robbins 2018).

**Natural hosts.** Immature stages are primarily on rodents and other small mammals, reptiles, small birds. Adults are on ungulates (primarily deer) and dogs. Zoonotic on humans (Guglielmone and Robbins 2018).

**Route of infection.** Exposure to larval, nymph, or adult ticks in the environment.

**Site in human host.** Skin.

**Vectored pathogens.***Borreliaburgdorferi* sensu lato and *B.mayonii* (Lyme disease), *B.miyamotoi* (tick-borne relapsing fever), *Babesiamicroti* (babesiosis), *Anaplasmaphagotocytophilum* (human granulocytic anaplasmosis), deer tick virus (Powassan virus lineage II) (Nelder et al. 2016; Westblade et al. 2017; Khan et al. 2019; Wolf et al. 2020).

***Ixodesschillingsi* Neumann, 1901**

**Geographic distribution.** Sub-Saharan Africa (Guglielmone and Robbins 2018).

**Natural hosts.** Non-human primates. Zoonotic on humans (Guglielmone and Robbins 2018).

**Route of infection.** Exposure to larval, nymph, or adult ticks in the environment.

**Site in human host.** Skin.

**Vectored pathogens.** None.

***Ixodessculptus* Neumann, 1904**

**Geographic distribution.** Central Asia, Russia, Japan (Guglielmone and Robbins 2018).

**Natural hosts.** Primarily rodents and lagomorphs, rarely ungulates and birds. Zoonotic on humans (Guglielmone and Robbins 2018).

**Route of infection.** Exposure to larval, nymph, or adult ticks in the environment.

**Site in human host.** Skin.

**Vectored pathogens.** None.

***Ixodessinensis* Teng, 1977**

**Geographic distribution.** China (Guglielmone and Robbins 2018).

**Natural hosts.** Mammals, including ungulates, rodents, and lagomorphs, and birds. Zoonotic on humans (Guglielmone and Robbins 2018).

**Route of infection.** Exposure to larval, nymph, or adult ticks in the environment.

**Site in human host.** Skin.

**Vectored pathogens.** None.

***Ixodessoricis* Gregson, 1942**

**Geographic distribution.** North America (Spencer 1963).

**Natural hosts.** Shrews and rodents. Zoonotic on humans (Spencer 1963).

**Route of infection.** Exposure to larval, nymph, or adult ticks in the environment.

**Site in human host.** Skin.

**Vectored pathogens.** None.

***Ixodesspinicoxalis* Neumann, 1899**

**Geographic distribution.** Southeast Asia (Guglielmone and Robbins 2018).

**Natural hosts.** Mammals, tree shrews, rodents, and carnivores, and birds. Zoonotic on humans (Guglielmone and Robbins 2018).

**Route of infection.** Exposure to larval, nymph, or adult ticks in the environment.

**Site in human host.** Skin.

**Vectored pathogens.** None.

***Ixodesspinipalpis* Hawden et Nuttall in Nutall, 1916**

**Geographic distribution.** North America (Guglielmone and Robbins 2018).

**Natural hosts.** Mammals, primarily rodents, but also lagomorphs and carnivores, and birds. Zoonotic on humans (Guglielmone and Robbins 2018).

**Route of infection.** Exposure to larval, nymph, or adult ticks in the environment.

**Site in human host.** Skin.

**Vectored pathogens.** None.

***Ixodestancitarius* Cooley & Kohls, 1942**

**Geographic distribution.** Mexico (Guglielmone and Robbins 2018).

**Natural hosts.** Rodents. Zoonotic on humans (Guglielmone and Robbins 2018).

**Route of infection.** Exposure to larval, nymph, or adult ticks in the environment.

**Site in human host.** Skin.

**Vectored pathogens.** None.

**Notes.** The single human record from Mexico is considered tentative (Guglielmone and Robbins 2018).

***Ixodestanuki* Saito, 1964**

**Geographic distribution.** Southeast Asia, Japan (Guglielmone and Robbins 2018).

**Natural hosts.** Mammals, including carnivores, rodents, and ungulates. Zoonotic on humans (Guglielmone and Robbins 2018).

**Route of infection.** Exposure to larval, nymph, or adult ticks in the environment.

**Site in human host.** Skin.

**Vectored pathogens.** None.

***Ixodestasmani* Neumann, 1899**

**Geographic distribution.** Southeast Asia, Japan (Guglielmone and Robbins 2018).

**Natural hosts.** Mammals, including marsupials, carnivores, and rodents. Zoonotic on humans (Guglielmone and Robbins 2018).

**Route of infection.** Exposure to larval, nymph, or adult ticks in the environment.

**Site in human host.** Skin.

**Vectored pathogens.** None.

***Ixodestexanus* Banks, 1909**

**Geographic distribution.** North America (Guglielmone and Robbins 2018).

**Natural hosts.** Mammals, primarily carnivores. Zoonotic on humans (Guglielmone and Robbins 2018).

**Route of infection.** Exposure to larval, nymph, or adult ticks in the environment.

**Site in human host.** Skin.

**Vectored pathogens.** None.

***Ixodestrainguliceps* Birula, 1895**

**Geographic distribution.** Europe, northwestern Asia (Guglielmone and Robbins 2018).

**Natural hosts.** Mammals, primarily shrews, but also rodents and carnivores; birds, lizards. Zoonotic on humans (Guglielmone and Robbins 2018).

**Route of infection.** Exposure to larval, nymph, or adult ticks in the environment.

**Site in human host.** Skin.

**Vectored pathogens.** None.

***Ixodesturdus* Nakatsudi, 1942**

**Geographic distribution.** Southeast Asia, Japan (Guglielmone and Robbins 2018).

**Natural hosts.** Birds, rarely rodents. Zoonotic on humans (Guglielmone and Robbins 2018).

**Route of infection.** Exposure to larval, nymph, or adult ticks in the environment.

**Site in human host.** Skin.

**Vectored pathogens.** None.

***Ixodesuriae* White, 1852**

*Hyalommaputa* Pickard-Cambridge, 1876

**Geographic distribution.** North America, Europe, Russia, southern Africa, Australia (Guglielmone and Robbins 2018).

**Natural hosts.** Predominately birds, but also mammals, including carnivores, rodents, and humans (Guglielmone and Robbins 2018).

**Route of infection.** Exposure to larval, nymph, or adult ticks in the environment.

**Site in human host.** Skin.

**Vectored pathogens.** None.

***Ixodesvanidicus* Schülze, 1943**

**Geographic distribution.** Sub-Saharan Africa (Estrada-Pena and Jongejan 1999).

**Natural hosts.** Mammals, including carnivores and elephant shrews. Zoonotic on humans (Estrada-Pena and Jongejan 1999).

**Route of infection.** Exposure to larval, nymph, or adult ticks in the environment.

**Site in human host.** Skin.

**Vectored pathogens.** None.

***Ixodesventalloi* Gil Collado, 1936**

**Geographic distribution.** Europe, northern Africa (Guglielmone and Robbins 2018).

**Natural hosts.** Mammals, including carnivores, rodents, and lagomorphs; birds, and reptiles. Zoonotic on humans (Guglielmone and Robbins 2018).

**Route of infection.** Exposure to larval, nymph, or adult ticks in the environment.

**Site in human host.** Skin.

**Vectored pathogens.** None.

***Ixodesvespertilionis* Koch, 1844**

**Geographic distribution.** Europe, Africa, Asia (Guglielmone and Robbins 2018).

**Natural hosts.** Bats. Zoonotic on humans (Guglielmone and Robbins 2018).

**Route of infection.** Exposure to larval, nymph, or adult ticks in the environment.

**Site in human host.** Skin.

**Vectored pathogens.** None.

***Ixodeswoodi* Bishopp, 1911**

**Geographic distribution.** North America (Guglielmone and Robbins 2018).

**Natural hosts.** Mammals, primarily rodents, also carnivores. Zoonotic on humans (Guglielmone and Robbins 2018).

**Route of infection.** Exposure to larval, nymph, or adult ticks in the environment.

**Site in human host.** Skin.

**Vectored pathogens.** None.

**Genus *Nosomma* Schülze, 1919**

***Nosommamonstrosum* (Nuttall & Warburton, 1908)**

**Geographic distribution.** Central and Southeast Asia (Guglielmone and Robbins 2018).

**Natural hosts.** Immature stage primarily on rodents and shrews. Adults primarily on bovids, deer, pics, horses, and carnivores. Zoonotic on humans (Guglielmone and Robbins 2018).

**Route of infection.** Exposure to larval, nymph, or adult ticks in the environment.

**Site in human host.** Skin.

**Vectored pathogens.** None.

**Genus *Rhipicephalus* Koch, 1844**

***Rhipicephalusannulatus* (Say, 1821)**

*Rhipicephaluscalcaratus* Birula, 1895

**Geographic distribution.** North America, Africa, Europe, Middle East, Central Asia (Guglielmone and Robbins 2018).

**Natural hosts.** Primarily cattle, but also other mammals, birds, and reptiles. Zoonotic on humans (Guglielmone and Robbins 2018).

**Route of infection.** Exposure to larval, nymph, or adult ticks in the environment.

**Site in human host.** Skin.

**Vectored pathogens.** None.

***Rhipicephalusappendiculatus* Neumann, 1901**

**Geographic distribution.** Sub-Saharan Africa (Perry et al. 1990).

**Natural hosts.** Mammals, including cattle, buffalo, antelope, warthogs, equids, lagomorphs, and dogs. Zoonotic on humans (Perry et al. 1990).

**Route of infection.** Exposure to larval, nymph, or adult ticks in the environment.

**Site in human host.** Skin.

**Vectored pathogens.** None.

***Rhipicephalusarmatus* Pocock, 1900**

**Geographic distribution.** East Africa (Guglielmone and Robbins 2018).

**Natural hosts.** Carnivores, bovids, and lagomorphs. Zoonotic on humans (Guglielmone and Robbins 2018).

**Route of infection.** Exposure to larval, nymph, or adult ticks in the environment.

**Site in human host.** Skin.

**Vectored pathogens.** None.

***Rhipicephalusaurantiacus* Neumann, 1907**

**Geographic distribution.** Sub-Saharan Africa (Guglielmone and Robbins 2018).

**Natural hosts.** Bovids, pigs. Zoonotic on humans (Guglielmone and Robbins 2018).

**Route of infection.** Exposure to larval, nymph, or adult ticks in the environment.

**Site in human host.** Skin.

**Vectored pathogens.** None.

***Rhipicephalusaustralis* Fuller, 1899.**

**Geographic distribution.** Southeast Asia, Australia, Pacific Islands (Guglielmone and Robbins 2018).

**Natural hosts.** Mammals, primarily bovids. Zoonotic on humans (Guglielmone and Robbins 2018).

**Route of infection.** Exposure to larval, nymph, or adult ticks in the environment.

**Site in human host.** Skin.

**Vectored pathogens.** None.

***Rhipicephalusbequaerti* Zumpt, 1950**

**Geographic distribution.** Sub-Saharan Africa (Guglielmone and Robbins 2018).

**Natural hosts.** Bovids, pigs. Zoonotic on humans (Guglielmone and Robbins 2018).

**Route of infection.** Exposure to larval, nymph, or adult ticks in the environment.

**Site in human host.** Skin.

**Vectored pathogens.** None.

***Rhipicephalusbursa* Canestrini er Fanzago, 1878**

**Geographic distribution.** Southern Europe, North Africa, Middle East, Central and East Asia (Guglielmone and Robbins 2018).

**Natural hosts.** Mammals, primarily bovids, and birds. Zoonotic on humans (Guglielmone and Robbins 2018).

**Route of infection.** Exposure to larval, nymph, or adult ticks in the environment.

**Site in human host.** Skin.

**Vectored pathogens.** None.

***Rhipicephaluscarnivoralis* Walker, 1966**

**Geographic distribution.** East Africa (Guglielmone and Robbins 2018).

**Natural hosts.** Primarily felids, also other mammals including bovids and hyrax. Zoonotic on humans (Guglielmone and Robbins 2018).

**Route of infection.** Exposure to larval, nymph, or adult ticks in the environment.

**Site in human host.** Skin.

**Vectored pathogens.** None.

***Rhipicephaluscomplanatus* Neumann, 1911**

**Geographic distribution.** West Africa (Guglielmone and Robbins 2018).

**Natural hosts.** Pigs, bovids, carnivores, and rodents. Zoonotic on humans (Guglielmone and Robbins 2018).

**Route of infection.** Exposure to larval, nymph, or adult ticks in the environment.

**Site in human host.** Skin.

**Vectored pathogens.** None.

***Rhipicephaluscompositus* Neumann, 1897**

**Geographic distribution.** Sub-Saharan Africa (Guglielmone and Robbins 2018).

**Natural hosts.** Immature stages primarily on rodents. Adults primarily on bovids, pigs, carnivores, horses, rhinoceroses. Zoonotic on humans (Guglielmone and Robbins 2018).

**Route of infection.** Exposure to larval, nymph, or adult ticks in the environment.

**Site in human host.** Skin.

**Vectored pathogens.** None.

***Rhipicephalusdecoloratus* Koch, 1844**

**Geographic distribution.** Sub-Saharan Africa (Guglielmone and Robbins 2018).

**Natural hosts.** Primarily bovids, also other mammals, birds, and tortoises. Zoonotic on humans (Guglielmone and Robbins 2018).

**Route of infection.** Exposure to larval, nymph, or adult ticks in the environment.

**Site in human host.** Skin.

**Vectored pathogens.** None.

***Rhipicephalusdistinctus* Bedford, 1932**

**Geographic distribution.** Sub-Saharan Africa (Guglielmone and Robbins 2018).

**Natural hosts.** Primarily hyrax, also lagomorphs, carnivores, rodents, and elephant shrews. Zoonotic on humans (Guglielmone and Robbins 2018).

**Route of infection.** Exposure to larval, nymph, or adult ticks in the environment.

**Site in human host.** Skin.

**Vectored pathogens.** None.

***Rhipicephalusevertsi* Neumann, 1897**

**Geographic distribution.** Sub-Saharan Africa (Guglielmone and Robbins 2018).

**Natural hosts.** Several groups of mammals and birds. Zoonotic on humans (Guglielmone and Robbins 2018).

**Route of infection.** Exposure to larval, nymph, or adult ticks in the environment.

**Site in human host.** Skin.

**Vectored pathogens.** None.

***Rhipicephalusfollis* Dönitz, 1910**

**Geographic distribution.** Sub-Saharan Africa (Guglielmone and Robbins 2018).

**Natural hosts.** Mammals, including bovids, pigs, carnivores, horses, rhinoceroses, hyrax, and rodents. Zoonotic on humans (Guglielmone and Robbins 2018).

**Route of infection.** Exposure to larval, nymph, or adult ticks in the environment.

**Site in human host.** Skin.

**Vectored pathogens.** None.

***Rhipicephalusfulvus* Neumann, 1913**

**Geographic distribution.** Central and northern Africa (Guglielmone and Robbins 2018).

**Natural hosts.** Bovids, camels, rodents. Zoonotic on humans (Guglielmone and Robbins 2018).

**Route of infection.** Exposure to larval, nymph, or adult ticks in the environment.

**Site in human host.** Skin.

**Vectored pathogens.** None.

***Rhipicephalusgertrudae* Feldman-Muhsam, 1960**

**Geographic distribution.** Southern Africa (Guglielmone and Robbins 2018).

**Natural hosts.** Several groups of mammals, birds, and reptiles. Immature stages primarily on rodents. Adults primarily on bovids. Zoonotic on humans (Guglielmone and Robbins 2018).

**Route of infection.** Exposure to larval, nymph, or adult ticks in the environment.

**Site in human host.** Skin.

**Vectored pathogens.** None.

***Rhipicephalusglabroscutatus* Du Toit, 1941**

**Geographic distribution.** South Africa (Guglielmone and Robbins 2018).

**Natural hosts.** Immature stages primarily on carnivores, rodents, birds. Adults primarily on lagomorphs, horses, and hyrax. Zoonotic on humans (Guglielmone and Robbins 2018).

**Route of infection.** Exposure to larval, nymph, or adult ticks in the environment.

**Site in human host.** Skin.

**Vectored pathogens.** None.

***Rhipicephalusguilhoni* Morel & Vassiliades, 1963**

**Geographic distribution.** Africa (Guglielmone and Robbins 2018).

**Natural hosts.** Several groups of mammals and birds. Immature stages primarily on rodents, lagomorphs. Adults primarily on bovids. Zoonotic on humans (Guglielmone and Robbins 2018).

**Route of infection.** Exposure to larval, nymph, or adult ticks in the environment.

**Site in human host.** Skin.

**Vectored pathogens.** None.

***Rhipicephalushaemaphysaloides* Supino, 1897**

**Geographic distribution.** Central and Southeast Asia (Guglielmone and Robbins 2018).

**Natural hosts.** Mammals, including carnivores, rodents, bovids, and shrews; birds. Zoonotic on humans (Guglielmone and Robbins 2018).

**Route of infection.** Exposure to larval, nymph, or adult ticks in the environment.

**Site in human host.** Skin, ear (Dilrukshi et al. 2004).

**Vectored pathogens.** None.

***Rhipicephalushumeralis* Tonelli-Rondelli, 1926**

**Geographic distribution.** East Africa (Guglielmone and Robbins 2018).

**Natural hosts.** Various groups of mammals. Zoonotic on humans (Guglielmone and Robbins 2018).

**Route of infection.** Exposure to larval, nymph, or adult ticks in the environment.

**Site in human host.** Skin.

**Vectored pathogens.** None.

***Rhipicephalushurti* Wilson, 1954**

**Geographic distribution.** East Africa (Guglielmone and Robbins 2018).

**Natural hosts.** Mammals, including bovids, pigs, carnivores, rhinoceroses, and rodents. Zoonotic on humans (Guglielmone and Robbins 2018).

**Route of infection.** Exposure to larval, nymph, or adult ticks in the environment.

**Site in human host.** Skin.

**Vectored pathogens.** None.

***Rhipicephalusjeanneli* Neumann, 1913**

**Geographic distribution.** East Africa (Guglielmone and Robbins 2018).

**Natural hosts.** Mammals, including bovids, pigs, horses, rhinoceroses, and carnivores, and birds. Zoonotic on humans (Guglielmone and Robbins 2018).

**Route of infection.** Exposure to larval, nymph, or adult ticks in the environment.

**Site in human host.** Skin.

**Vectored pathogens.** None.

***Rhipicephaluskochi* Dönitz, 1905**

*Rhipicephalusneavi* Warburton, 1912

**Geographic distribution.** Sub-Saharan Africa (Guglielmone and Robbins 2018).

**Natural hosts.** Mammals, including bovids, elephant shrews, lagomorphs, and pigs; birds. Zoonotic on humans (Guglielmone and Robbins 2018).

**Route of infection.** Exposure to larval, nymph, or adult ticks in the environment.

**Site in human host.** Skin.

**Vectored pathogens.** None.

***Rhipicephaluslongus* Neumann, 1907**

**Geographic distribution.** Sub-Saharan Africa (Guglielmone and Robbins 2018).

**Natural hosts.** Mammals, primarily bovids and pigs. Zoonotic on humans (Guglielmone and Robbins 2018).

**Route of infection.** Exposure to larval, nymph, or adult ticks in the environment.

**Site in human host.** Skin.

**Vectored pathogens.** None.

***Rhipicephaluslunulatus* Neumann, 1907**

**Geographic distribution.** Sub-Saharan Africa (Guglielmone and Robbins 2018).

**Natural hosts.** Mammals, including bovids, lagomorphs, elephant shrews, and rodents; birds. Zoonotic on humans (Guglielmone and Robbins 2018).

**Route of infection.** Exposure to larval, nymph, or adult ticks in the environment.

**Site in human host.** Skin.

**Vectored pathogens.** None.

***Rhipicephalusmaculatus* Neumann, 1901**

**Geographic distribution.** Southern and East Africa (Guglielmone and Robbins 2018).

**Natural hosts.** Mammals, including bovids, rhinoceroses, carnivores, horses, and elephants; rarely reptiles. Zoonotic on humans (Guglielmone and Robbins 2018).

**Route of infection.** Exposure to larval, nymph, or adult ticks in the environment.

**Site in human host.** Skin.

**Vectored pathogens.** None.

***Rhipicephalusmicroplus* (Canestrini, 1888)**

**Geographic distribution.** Circumtropical (Guglielmone and Robbins 2018).

**Natural hosts.** Several groups of mammals, especially livestock; birds and reptiles. Zoonotic on humans (Guglielmone and Robbins 2018).

**Route of infection.** Exposure to larval, nymph, or adult ticks in the environment.

**Site in human host.** Skin.

**Vectored pathogens.** None.

***Rhipicephalusmuehlensi* Zumpt, 1943**

**Geographic distribution.** Africa (Guglielmone and Robbins 2018).

**Natural hosts.** Several groups of mammals. Zoonotic on humans (Guglielmone and Robbins 2018).

**Route of infection.** Exposure to larval, nymph, or adult ticks in the environment.

**Site in human host.** Skin.

**Vectored pathogens.** None.

***Rhipicephaluspilans* Schülze, 1935**

**Geographic distribution.** East Timor, Indonesia, Philippines (Guglielmone and Robbins 2018).

**Natural hosts.** Several groups of mammals. Zoonotic on humans (Guglielmone and Robbins 2018).

**Route of infection.** Exposure to larval, nymph, or adult ticks in the environment.

**Site in human host.** Skin.

**Vectored pathogens.** None.

***Rhipicephalusplanus* Neumann, 1907**

*Rhipicephalusreichenowi* Zumpt, 1943

**Geographic distribution.** Sub-Saharan Africa (Guglielmone and Robbins 2018).

**Natural hosts.** Several groups of mammals. Immature stages primarily on rodents and lagomorphs. Adults primarily on bovids and pigs. Zoonotic on humans (Guglielmone and Robbins 2018).

**Route of infection.** Exposure to larval, nymph, or adult ticks in the environment.

**Site in human host.** Skin.

**Vectored pathogens.** None.

***Rhipicephaluspraetextatus* Gerstäcker, 1873**

**Geographic distribution.** Africa, Yemen (Guglielmone and Robbins 2018).

**Natural hosts.** Several groups of mammals and birds. Immature stages primarily on rodents. Adults primarily on bovids. Zoonotic on humans (Guglielmone and Robbins 2018).

**Route of infection.** Exposure to larval, nymph, or adult ticks in the environment.

**Site in human host.** Skin.

**Vectored pathogens.** None.

***Rhipicephalusparvus* Dönitz, 1910**

**Geographic distribution.** East Africa (Guglielmone and Robbins 2018).

**Natural hosts.** Several groups of mammals, birds, and reptiles. Zoonotic on humans (Guglielmone and Robbins 2018).

**Route of infection.** Exposure to larval, nymph, or adult ticks in the environment.

**Site in human host.** Skin.

**Vectored pathogens.** None.

***Rhipicephaluspulchellus* (Gerstäcker, 1873)**

**Geographic distribution.** East Africa (Guglielmone and Robbins 2018).

**Natural hosts.** Several groups of mammals, especially bovids, and birds. Zoonotic on humans (Guglielmone and Robbins 2018).

**Route of infection.** Exposure to larval, nymph, or adult ticks in the environment.

**Site in human host.** Skin.

**Vectored pathogens.** None.

***Rhipicephaluspumilio* Schülze, 1935**

**Geographic distribution.** Central and East Asia (Guglielmone and Robbins 2018).

**Natural hosts.** Several groups of mammals, including hedgehogs, rodents, and lagomorphs, and birds. Zoonotic on humans (Guglielmone and Robbins 2018).

**Route of infection.** Exposure to larval, nymph, or adult ticks in the environment.

**Site in human host.** Skin.

**Vectored pathogens.** None.

***Rhipicephaluspusillus* Gil Collado, 1936**

**Geographic distribution.** Mediterranean Europe and Africa(Guglielmone and Robbins 2018).

**Natural hosts.** Several groups of mammals and birds. Zoonotic on humans (Guglielmone and Robbins 2018).

**Route of infection.** Exposure to larval, nymph, or adult ticks in the environment.

**Site in human host.** Skin.

**Vectored pathogens.** None.

***Rhipicephalusrossicus* Yakimov et Kohl-Yakimova, 1911**

**Geographic distribution.** Eastern Europe, Asia (Guglielmone and Robbins 2018).

**Natural hosts.** Several groups of mammals, birds, and reptiles. Zoonotic on humans (Guglielmone and Robbins 2018).

**Route of infection.** Exposure to larval, nymph, or adult ticks in the environment.

**Site in human host.** Skin.

**Vectored pathogens.** None.

***Rhipicephalussanguineus* (Latreille, 1806)**

**Geographic distribution.** Worldwide (Guglielmone and Robbins 2018).

**Natural hosts.** Several groups of mammals, especially dogs. Zoonotic on humans (Guglielmone and Robbins 2018).

**Route of infection.** Exposure to larval, nymph, or adult ticks in the environment.

**Site in human host.** Skin.

**Vectored pathogens.***Rickettsiarickettsii* (Rocky Mountain spotted fever), *R.conorii* (boutonneuse fever) (Dantas-Torres 2010; Dantas-Torres et al. 2012).

***Rhipicephalusschulzei* Olenev, 1929**

**Geographic distribution.** Central and East Asia (Guglielmone and Robbins 2018).

**Natural hosts.** Mammals, including rodents, lagomorphs, and carnivores; birds. Zoonotic on humans (Guglielmone and Robbins 2018).

**Route of infection.** Exposure to larval, nymph, or adult ticks in the environment.

**Site in human host.** Skin.

**Vectored pathogens.** None.

***Rhipicephalussenegalensis* Koch, 1844**

**Geographic distribution.** Sub-Saharan Africa (Guglielmone and Robbins 2018).

**Natural hosts.** Several groups of mammals and birds. Immature stages primarily on carnivores, elephant shrews, and rodents. Adults primarily on bovids, especially domestic cattle, and other mammals. Zoonotic on humans (Guglielmone and Robbins 2018).

**Route of infection.** Exposure to larval, nymph, or adult ticks in the environment.

**Site in human host.** Skin.

**Vectored pathogens.** None.

***Rhipicephalussimus* Koch, 1844**

**Geographic distribution.** Southern and East Africa (Guglielmone and Robbins 2018).

**Natural hosts.** Several groups of mammals. Zoonotic on humans (Guglielmone and Robbins 2018).

**Route of infection.** Exposure to larval, nymph, or adult ticks in the environment.

**Site in human host.** Skin.

**Vectored pathogens.** None.

***Rhipicephalussulcatus* Neumann, 1908**

**Geographic distribution.** Sub-Saharan Africa (Guglielmone and Robbins 2018).

**Natural hosts.** Several groups of mammals. Zoonotic on humans (Guglielmone and Robbins 2018).

**Route of infection.** Exposure to larval, nymph, or adult ticks in the environment.

**Site in human host.** Skin.

**Vectored pathogens.** None.

***Rhipicephalussupertritus* Neumann, 1907**

**Geographic distribution.** Sub-Saharan Africa (Guglielmone and Robbins 2018).

**Natural hosts.** Several groups of mammals, especially bovids. Zoonotic on humans (Guglielmone and Robbins 2018).

**Route of infection.** Exposure to larval, nymph, or adult ticks in the environment.

**Site in human host.** Skin.

**Vectored pathogens.** None.

***Rhipicephalusturanicus* Pomerantzev, 1936**

**Geographic distribution.** Hard to define but generally considered broadly distributed in Africa and Asia (Guglielmone and Robbins 2018).

**Natural hosts.** Several groups of mammals, including humans, birds, and reptiles (Guglielmone and Robbins 2018).

**Route of infection.** Exposure to larval, nymph, or adult ticks in the environment.

**Site in human host.** Skin.

**Vectored pathogens.***Rickettsiaconorii* (boutonneuse fever) (Dantas-Torres et al. 2012).

***Rhipicephaluswarburtoni* Walker et Horak in Walker et al., 2000**

**Geographic distribution.** South Africa (Guglielmone and Robbins 2018).

**Natural hosts.** Mammals, including bovids, elephant shrews, lagomorphs, carnivores, horses, and rodents; birds. Zoonotic on humans (Guglielmone and Robbins 2018).

**Route of infection.** Exposure to larval, nymph, or adult ticks in the environment.

**Site in human host.** Skin.

**Vectored pathogens.** None.

***Rhipicephaluszambeziensis* Walker et al., 1981**

**Geographic distribution.** Sub-Saharan Africa (Guglielmone and Robbins 2018).

**Natural hosts.** Many groups of mammals and birds. Immature stages primarily on lagomorphs. Adults primarily on bovids. Zoonotic on humans (Guglielmone and Robbins 2018).

**Route of infection.** Exposure to larval, nymph, or adult ticks in the environment.

**Site in human host.** Skin.

**Vectored pathogens.** None.

***Rhipicephalusziemanni* Neumann, 1904**

**Geographic distribution.** Sub-Saharan Africa (Guglielmone and Robbins 2018).

**Natural hosts.** Mammals, primarily carnivores and rodents. Zoonotic on humans (Guglielmone and Robbins 2018).

**Route of infection.** Exposure to larval, nymph, or adult ticks in the environment.

**Site in human host.** Skin.

**Vectored pathogens.** None.

***Rhipicephaluszumpti* Santos Dias, 1950**

**Geographic distribution.** Southern and Eastern Africa (Guglielmone and Robbins 2018).

**Natural hosts.** Several groups of mammals. Zoonotic on humans (Guglielmone and Robbins 2018).

**Route of infection.** Exposure to larval, nymph, or adult ticks in the environment.

**Site in human host.** Skin.

**Vectored pathogens.** None.

●●●●●●●●●●●●● **Argasidae Koch, 1844**

**Genus *Argas* Latreille, 1795**

***Argasmonolakensis* Schwan et al. 1992**

**Geographic distribution.** Mono Lake, California, USA (Schwan et al. 1992)

**Natural hosts.** Birds, primarily California gulls (*Laruscalifornicus*) (Schwan et al. 1992).

**Route of infection.** Exposure to larval, nymph, or adult ticks in the environment.

**Site in human host.** Skin.

**Vectored pathogens.** None.

***Argaspersicus* (Oken, 1818)**

**Geographic distribution.** Worldwide (Hoogstraal 1985).

**Natural hosts.** Birds. Zoonotic on humans as incidental hosts (Hoogstraal 1985).

**Route of infection.** Exposure to larval, nymph, or adult ticks in the environment.

**Site in human host.** Skin.

**Vectored pathogens.** Kyasanur Forest disease virus (Hoogstraal 1985).

**Genus *Ornithodoros* Koch, 1844**

***Ornithodoroserraticus* Lucas, 1849**

**Geographic distribution.** Mediterranean Region (Talagrand-Reboul et al. 2018).

**Natural hosts.** Many mammals, including pigs, rodents, insectivores, canids, mustelids, bats. Zoonotic on humans as incidental hosts (Talagrand-Reboul et al. 2018).

**Route of infection.** Exposure to larval, nymph, or adult ticks in the environment.

**Site in human host.** Skin.

**Vectored pathogens.***Borreliahispanica* (tick-borne relapsing fever) (Boinas et al. 2014; Talagrand-Reboul et al. 2018).

***Ornithodorosgraingeri* Heisch & Guggisberg, 1953**

**Geographic distribution.** Kenya (Heisch and Harvey 1953).

**Natural hosts.** Rodents. Zoonotic on humans as incidental hosts (Heisch and Harvey 1953; Talagrand-Reboul et al. 2018).

**Route of infection.** Exposure to larval, nymph, or adult ticks in the environment.

**Site in human host.** Skin.

**Vectored pathogens.***Borreliagraingeri* (tick-borne relapsing fever) (Talagrand-Reboul et al. 2018).

***Ornithodoroshermsi* Wheeler, 1935**

**Geographic distribution.** Western North America (Sage et al. 2017).

**Natural hosts.** Rodents. Zoonotic on humans as incidental hosts (Sage et al. 2017; Talagrand-Reboul et al. 2018).

**Route of infection.** Exposure to larval, nymph, or adult ticks in the environment.

**Site in human host.** Skin.

**Vectored pathogens.***Borreliahermsii* (tick-borne relapsing fever) (Sage et al. 2017; Talagrand-Reboul et al. 2018).

***Ornithodorosmarocanus* Velu, 1919**

**Geographic distribution.** Mediterranean Region (Vial 2009).

**Natural hosts.** Many mammals, including rodents, insectivores, canids, mustelids, bats. Zoonotic on humans as incidental hosts (Vial 2009; Talagrand-Reboul et al. 2018).

**Route of infection.** Exposure to larval, nymph, or adult ticks in the environment.

**Site in human host.** Skin.

**Vectored pathogens.***Borreliahispanica* (tick-borne relapsing fever) (Talagrand-Reboul et al. 2018).

***Ornithodorosmoubata* Murray, 1877**

**Geographic distribution.** Sub-Saharan Africa (Vial 2009).

**Natural hosts.** Pigs, poultry. Zoonotic on humans as incidental hosts (Vial 2009; Talagrand-Reboul et al. 2018).

**Route of infection.** Exposure to larval, nymph, or adult ticks in the environment.

**Site in human host.** Skin.

**Vectored pathogens.***Borreliaduttoni* (tick-borne relapsing fever) (Talagrand-Reboul et al. 2018).

***Ornithodorosparkeri* Cooley, 1936**

**Geographic distribution.** Western North America (Lopez et al. 2016).

**Natural hosts.** Rodents, horses. Zoonotic on humans as incidental hosts (Lopez et al. 2016; Talagrand-Reboul et al. 2018).

**Route of infection.** Exposure to larval, nymph, or adult ticks in the environment.

**Site in human host.** Skin.

**Vectored pathogens.***Borreliaparkeri* (tick-borne relapsing fever) (Lopez et al. 2016; Talagrand-Reboul et al. 2018).

***Ornithodorosrudis* Karsch, 1880**

*Ornithodorosvenezuelensis* Brumpt, 1921

**Geographic distribution.** Panama and South America (Faccini-Martínez Á and Botero-García 2016).

**Natural hosts.** Birds, primarily poultry. Zoonotic on humans as incidental hosts (Faccini-Martínez Á and Botero-García 2016; Talagrand-Reboul et al. 2018).

**Route of infection.** Exposure to larval, nymph, or adult ticks in the environment.

**Site in human host.** Skin.

**Vectored pathogens.***Borreliavenezuelensis* (tick-borne relapsing fever) (Faccini-Martínez Á and Botero-García 2016; Talagrand-Reboul et al. 2018).

***Ornithodorossonrai* Sautet & Witkowski, 1944**

**Geographic distribution.** Western and northern Africa (Vial 2009).

**Natural hosts.** Rodents, insectivores. Zoonotic on humans as incidental hosts (Vial 2009; Talagrand-Reboul et al. 2018).

**Route of infection.** Exposure to larval, nymph, or adult ticks in the environment.

**Site in human host.** Skin.

**Vectored pathogens.***Borreliacrocidurae* (tick-borne relapsing fever) (Talagrand-Reboul et al. 2018).

***Ornithodorostalajae* (Guérin-Méneville, 1849)**

**Geographic distribution.** Southern United States, Central and South America (Cooley 1945; Faccini-Martínez Á and Botero-García 2016).

**Natural hosts.** Many mammals. Zoonotic on humans as incidental hosts (Cooley 1945; Talagrand-Reboul et al. 2018).

**Route of infection.** Exposure to larval, nymph, or adult ticks in the environment.

**Site in human host.** Skin.

**Vectored pathogens.***Borrelliamazzottii* (tick-borne relapsing fever) (Elelu 2018; Talagrand-Reboul et al. 2018; Moraru 2019).

***Ornithodorostholozani* Laboulbène & Mégnin, 1882**

**Geographic distribution.** Middle East, North Africa, Central Asia, India (Vial 2009).

**Natural hosts.** Rodents, insectivores. Zoonotic on humans as incidental hosts (Vial 2009; Talagrand-Reboul et al. 2018).

**Route of infection.** Exposure to larval, nymph, or adult ticks in the environment.

**Site in human host.** Skin.

**Vectored pathogens.***Borreliapersica* (tick-borne relapsing fever) (Talagrand-Reboul et al. 2018).

***Ornithodorosturicata* (Dugès, 1876)**

**Geographic distribution.** Western and Midwestern North America, Mexico (Cooley 1945).

**Natural hosts.** Rodents, dogs. Zoonotic on humans as incidental hosts (Cooley 1945; Talagrand-Reboul et al. 2018).

**Route of infection.** Exposure to larval, nymph, or adult ticks in the environment.

**Site in human host.** Skin.

**Vectored pathogens.***Borreliaturicatae* (tick-borne relapsing fever) (Talagrand-Reboul et al. 2018).

***Ornithodorosverrucosus* Olenev et al., 1934**

**Geographic distribution.** Eastern Europe (Vial 2009).

**Natural hosts.** Rodents. Zoonotic on humans as incidental hosts (Vial 2009).

**Route of infection.** Exposure to larval, nymph, or adult ticks in the environment.

**Site in human host.** Skin.

**Vectored pathogens.***Borreliacaucasica* (tick-borne relapsing fever) (Talagrand-Reboul et al. 2018).

**Genus *Otobius* Banks, 1912**

***Otobiusmegnini* (Dugès, 1884)**

**Geographic distribution.** Nearly worldwide wherever livestock are raised (Cooley 1945).

**Natural hosts.** Cattle, sheep, goats, deer, other ruminants, carnivores, rabbits. Zoonotic on humans as incidental hosts (Cooley 1945).

**Route of infection.** Exposure to larval or nymph ticks in the environment.

**Site in human host.** Ears (Cooley 1945; Naudé et al. 2001; Mazlumoglu 2018).

**Vectored pathogens.** None.

●●●●●●●●●●●● **Mesostigmata Canestrini, 1891**

●●●●●●●●●●●●● **Dermanyssoidea Kolenati, 1859**

●●●●●●●●●●●●●● **Dermanyssidae Kolenati, 1859**

**Genus *Dermanyssus* Dugès, 1834**

***Dermanyssusgallinae* (DeGeer, 1778)**

**Geographic distribution.** Worldwide (Cafiero et al. 2019).

**Natural hosts.** Birds. Zoonotic on humans as incidental hosts (Cafiero et al. 2019).

**Route of infection.** Contact with infected birds (Cafiero et al. 2019).

**Site in human host.** Skin, ear (Rossiter 1997; Cafiero et al. 2019).

**Vectored pathogens.** None.

**Genus *Liponyssoides* Hirst, 1913**

***Liponyssoidessanguineus* (Hirst, 1914)**

**Geographic distribution.** Worldwide (Diaz 2009).

**Natural hosts.** Rodents. Zoonotic on humans as incidental hosts (Diaz 2009).

**Route of infection.** Contact with and living among infected rodents (Diaz 2009).

**Site in human host.** Skin (Diaz 2009).

**Vectored organism.***Rickettsiaakari* (rickettsialpox) (Saini et al. 2004).

●●●●●●●●●●●●●● **Laelapidae Berlese, 1892**

**Genus *Laelaps* Koch, 1836**

***Laelapsechidnina* Berlese, 1887**

**Geographic distribution.** Worldwide (Watson 2008).

**Natural hosts.** Rodents, primarily rats (*Rattus*). Zoonotic on humans as incidental hosts (Watson 2008).

**Route of infection.** Contact with and living among infected rodents (Watson 2008).

**Site in human host.** Skin (Watson 2008).

**Vectored pathogens.** None.

●●●●●●●●●●●●●● **Macronyssidae Oudemans, 1936**

**Genus *Ophionyssus* Mégnin, 1884**

***Ophionyssusnatricis* (Gervais, 1844)**

**Geographic distribution.** Worldwide (Wozniak and DeNardo 2000).

**Natural hosts.** Reptiles, primarily snakes, also lizards. Zoonotic on humans as incidental hosts (Schutlz 1975; Wozniak and DeNardo 2000; Amanatfard et al. 2014).

**Route of infection.** Contact with infected reptiles (Schutlz 1975; Amanatfard et al. 2014).

**Site in human host.** Skin (Schutlz 1975; Amanatfard et al. 2014).

**Vectored pathogens.** None.

**Genus *Ornithonyssus* Sambon, 1928**

***Ornithonysusbacoti* (Hirst, 1913)**

**Geographic distribution.** Tropics and subtropics worldwide (Diaz 2009).

**Natural hosts.** Rodents, primarily rats (*Rattus*). Zoonotic on humans as incidental hosts (Diaz 2009).

**Route of infection.** Contact with and living among infected rodents (Diaz 2009).

**Site in human host.** Skin (Diaz 2009).

**Vectored pathogens.** None.

***Ornithonyssusbursa* (Berlese, 1888)**

**Geographic distribution.** Tropics and subtropics worldwide (Diaz 2009).

**Natural hosts.** Birds. Zoonotic on humans as incidental hosts (Diaz 2009).

**Route of infection.** Contact with and living among infected birds (Diaz 2009).

**Site in human host.** Skin (Diaz 2009).

**Vectored pathogens.** None.

***Ornithonyssussylviarum* (Canestrini & Fanzago, 1877)**

**Geographic distribution.** Worldwide (Murillo and Mullens 2017).

**Natural hosts.** Birds. Zoonotic on humans as incidental hosts (Murillo and Mullens 2017).

**Route of infection.** Contact with and living among infected birds (Murillo and Mullens 2017).

**Site in human host.** Skin (Murillo and Mullens 2017).

**Vectored pathogens.** None.

●●●●●●● **Mandibulata Snodgrass, 1938**

●●●●●●●● **Pancrustacea Zrzavý & Štys, 1997**

Tetraconata Dohle, 2001

●●●●●●●●● **Crustacea Brünnich, 1772**

●●●●●●●●●● **Maxillopoda Dahl, 1956**

●●●●●●●●●●● **Pentastomida Diesing, 1836**

●●●●●●●●●●● **Porocephalidae Sambon, 1922**

**Genus *Armillifer* Sambon, 1922**

***Armilliferagkistrodontis* Self & Kuntz, 1966**

**Geographic distribution.** China (Chen et al. 2010; Christoffersen and Assis 2013; Ioannou and Vamvoukaki 2019).

**Natural hosts.** Intermediate hosts are rodents. Definitive hosts are snakes. Zoonotic in humans as dead-end hosts (Chen et al. 2010; Christoffersen and Assis 2013; Ioannou and Vamvoukaki 2019).

**Route of infection.** Ingestion of infected intermediate or reservoir host (Chen et al. 2010; Ioannou and Vamvoukaki 2019).

**Site in human host.** Disseminated infection, including the mesenteries, peritoneum, lungs (Chen et al. 2010; Ioannou and Vamvoukaki 2019).

***Armilliferarmillatus* Wyman, 1845**

**Geographic distribution.** Central and western Africa (Christoffersen and Assis 2013; Ioannou and Vamvoukaki 2019).

**Natural hosts.** Intermediate hosts are various mammals. Definitive hosts are pythons. Zoonotic in humans as dead-end hosts (Christoffersen and Assis 2013; Ioannou and Vamvoukaki 2019).

**Route of infection.** Ingestion of infected intermediate or reservoir host (Ioannou and Vamvoukaki 2019).

**Site in human host.** Disseminated infection, including the mesenteries, peritoneum, lungs, brain, eye. (Ioannou and Vamvoukaki 2019).

***Armillifergrandis* Hett, 1915**

**Geographic distribution.** Central and western Africa (Christoffersen and Assis 2013; Ioannou and Vamvoukaki 2019).

**Natural hosts.** Intermediate hosts are rodents. Definitive hosts are viperid snakes. Zoonotic in humans as dead-end hosts (Christoffersen and Assis 2013; Ioannou and Vamvoukaki 2019).

**Route of infection.** Ingestion of infected intermediate or reservoir host (Ioannou and Vamvoukaki 2019).

**Site in human host.** Disseminated infection, including the mesenteries, peritoneum, lungs, brain, and eye (Tappe and Büttner 2009; Ioannou and Vamvoukaki 2019).

***Armillifermoniliformis* (Diesing, 1835)**

**Geographic distribution.** Southeast Asia (Christoffersen and Assis 2013; Ioannou and Vamvoukaki 2019).

**Natural hosts.** Intermediate hosts are various mammals. Definitive hosts are snakes. Zoonotic in humans as dead-end hosts (Christoffersen and Assis 2013; Ioannou and Vamvoukaki 2019).

**Route of infection.** Ingestion of infected intermediate or reservoir host (Ioannou and Vamvoukaki 2019).

**Site in human host.** Disseminated infection, including the mesenteries, peritoneum, lungs, liver (Latif et al. 2011; Ioannou and Vamvoukaki 2019).

**Genus *Porocephalus* Humboldt, 1811**

***Porocephaluscrotali* (Humboldt, 1808)**

**Geographic distribution.** New World (Esslinger 1962; Christoffersen and Assis 2013).

**Natural hosts.** Intermediate hosts are small mammals, including rodents, monkeys. Definitive hosts are snakes, primarily rattlesnakes (*Crotalus*). Zoonotic in humans as dead-end hosts (Esslinger 1962; Christoffersen and Assis 2013).

**Route of infection.** Ingestion of infected intermediate or reservoir host (Tappe and Büttner 2009).

**Site in human host.** Disseminated infection, including the mesenteries, peritoneum, lungs (Tappe and Büttner 2009).

***Porocephalussubuliferum* (Leuckart, 1860)**

**Geographic distribution.** Africa (Sambon 1922; Christoffersen and Assis 2013).

**Natural hosts.** Intermediate and definitive hosts are snakes. Zoonotic in humans as dead-end hosts (Sambon 1922; Christoffersen and Assis 2013).

**Route of infection.** Ingestion of infected intermediate host (Sambon 1922).

**Site in human host.** Viscera (Sambon 1922; Christoffersen and Assis 2013).

***Porocephalustaiwana* Qui et al., 2005**

**Geographic distribution.** Southeast Asia (Qiu et al. 2005; Yao et al. 2008; Christoffersen and Assis 2013).

**Natural hosts.** Intermediate hosts are unknown. Definitive hosts are snakes. Zoonotic in humans as dead-end hosts (Christoffersen and Assis 2013).

**Route of infection.** Ingestion of infected intermediate or reservoir host (Qiu et al. 2005).

**Site in human host.** Disseminated disease, including greater omentum, small intestine, liver, lungs (Qiu et al. 2005; Yao et al. 2008).

●●●●●●●●●●● **Linguatulidae Haldeman, 1851**

**Genus *Linguatula* Fröhlich, 1789**

***Linguatulaserrata* Fröhlich, 1789**

**Geographic distribution.** Worldwide (Christoffersen and Assis 2013).

**Natural hosts.** Intermediate hosts are sheep, bovids, rodents. Definitive hosts are felids and canids. Zoonotic in humans as dead-end hosts (Christoffersen and Assis 2013).

**Route of infection.** Ingestion of infected intermediate or reservoir host (Tappe and Büttner 2009).

**Site in human host.** Disseminated infection, including the mesenteries, peritoneum, lungs, eyes (Tappe and Büttner 2009).

●●●●●●●●●●● **Raillietiellidae Sambon, 1922**

**Genus *Raillietiella* Sambon in Vaney & Sambon, 1910**

**Geographic distribution.** Worldwide (Christoffersen and Assis 2013).

**Natural hosts.** Intermediate hosts are insects. Definitive hosts are reptiles and amphibians. Zoonotic in humans as dead-end hosts (Christoffersen and Assis 2013).

**Route of infection.** Ingestion of infected intermediate or reservoir host (Tappe et al. 2016b).

**Site in human host.** Disseminated infection, including the mesenteries, peritoneum, lungs (Tappe et al. 2016b).

**Notes.***Raillietiella* isolates from human hosts have not been characterized at the species level. Cases of *R.hemidactyli* Hett, 1934 and *R.gehyrae* Bovien, 1927 from Vietnam were not confirmed morphologically (Tappe et al. 2016b).

●●●●●●●●●●● **Sebekidae Sambon, 1922**

**Genus *Leiperia* Sambon, 1922**

***Leiperiacincinallis* Sambon, in Vaney and Sambon 1910**

**Geographic distribution.** Africa (Christoffersen and Assis 2013).

**Natural hosts.** Intermediate hosts are freshwater fish. Definitive hosts are crocodiles and turtles. Zoonotic in humans as dead-end hosts (Christoffersen and Assis 2013).

**Route of infection.** Presumably ngestion of infected intermediate or paratenic host.

**Site in human host.** Intestine (?) (Fain 1960, 1961)

**Notes.***Leiperiacincinallis* has been reported from the stool of a European woman in Zaire (Fain 1960, 1961), but it is believed this may represent spurious passage after ingesting infected fish (Christoffersen and Assis 2015).

**Genus *Sebekia* Sambon, 1922**

**Geographic distribution.** Australia, South America, Southeast Asia, southern Africa (Christoffersen and Assis 2013).

**Natural hosts.** Intermediate hosts are freshwater fish. Definitive hosts are crocodiles and snakes (Christoffersen and Assis 2013).

**Route of infection.** Presumably by ingestion of infected intermediate or paratenic host.

**Site in human host.** Skin (Mairena et al. 1989).

**Notes.** A single human case of *Sebekia*, manifesting as dermatitis in a woman in Costa Rica (Mairena et al. 1989) was not identified to the species level.

●●●●●●●●● **Hexapoda Latreille, 1825**

●●●●●●●●●● **Insecta Linnaeus, 1758**

●●●●●●●●●●● **Hemiptera Linnaeus, 1758**

●●●●●●●●●●●● **Reduviidae Latreille, 1807**

**Genus *Meccus* Stål, 1859**

***Meccuspallidipennis* (Stål, 1872)**

**Geographic distribution.** Mexico (Franzim-Junior et al. 2018).

**Natural hosts.** Various mammals. Zoonotic on humans (Franzim-Junior et al. 2018).

**Route of infection.** Exposure to nymphs and adults in the environment.

**Site in human host.** Skin.

**Vectored pathogens.***Trypanosomacruzi* (Chagas Disease, American trypanosomiasis) (Franzim-Junior et al. 2018).

**Genus *Panstrongylus* Berg, 1879**

***Panstrongylusgeniculatus* (Latreille, 1811)**

**Geographic distribution.** Central and South America (Melo et al. 2018).

**Natural hosts.** Armadillos. Zoonotic on humans (Melo et al. 2018).

**Route of infection.** Exposure to nymphs and adults in the environment.

**Site in human host.** Skin.

**Vectored pathogens.** None.

***Panstrongylusmegistus* (Burmeister, 1835)**

**Geographic distribution.** Argentina, Bolivia, Brazil, Paraguay, Uruguay (Jurberg and Galvão 2006).

**Natural hosts.** Various mammals. Zoonotic on humans (Jurberg and Galvão 2006).

**Route of infection.** Exposure to nymphs and adults in the environment.

**Site in human host.** Skin.

**Vectored pathogens.***Trypanosomacruzi* (Chagas disease, American trypanosomiasis) (Jurberg and Galvão 2006).

**Genus *Paratriatoma* Barber, 1938**

***Paratriatomahirsuta* Barber, 1938**

**Geographic distribution.** Southwestern USA, Mexico (Bern et al. 2011).

**Natural hosts.** Rodents. Zoonotic on humans (Bern et al. 2011).

**Route of infection.** Exposure to nymphs and adults in the environment.

**Site in human host.** Skin.

**Vectored pathogens.** None.

**Genus *Rhodnius* Stål, 1859**

***Rhodniusprolixus* Stål, 1859**

**Geographic distribution.** Central and South America (Jurberg and Galvão 2006).

**Natural hosts.** Various mammals. Zoonotic on humans (Jurberg and Galvão 2006).

**Route of infection.** Exposure to nymphs and adults in the environment.

**Site in human host.** Skin.

**Vectored pathogens.***Trypanosomacruzi* (Chagas disease, American trypanosomiasis) (Jurberg and Galvão 2006).

**Genus *Triatoma* Laporte de Castelnau, 1832**

***Triatomabrasiliensis* Neiva, 1911**

**Geographic distribution.** Brazil (Jurberg and Galvão 2006).

**Natural hosts.** Various mammals. Zoonotic on humans (Jurberg and Galvão 2006).

**Route of infection.** Exposure to nymphs and adults in the environment.

**Site in human host.** Skin.

**Vectored pathogens.***Trypanosomacruzi* (Chagas disease, American trypanosomiasis) (Jurberg and Galvão 2006).

***Triatomadimidiata* (Latrielle, 1811)**

**Geographic distribution.** Central and South America (Jurberg and Galvão 2006).

**Natural hosts.** Various mammals. Zoonotic on humans (Jurberg and Galvão 2006).

**Route of infection.** Exposure to nymphs and adults in the environment.

**Site in human host.** Skin.

**Vectored pathogens.***Trypanosomacruzi* (Chagas disease, American trypanosomiasis) (Jurberg and Galvão 2006).

***Triatomagerstaeckeri* (Stål, 1859)**

**Geographic distribution.** Southwestern USA, Mexico (Wozniak et al. 2015).

**Natural hosts.** Various mammals. Zoonotic on humans (Wozniak et al. 2015).

**Route of infection.** Exposure to nymphs and adults in the environment.

**Site in human host.** Skin.

**Vectored pathogens.** None (*T.gerstaeckeri* has been found to be naturally infected with *Trypanosomacruzi* but its role as a natural vector for human disease is not fully understood) (Reisenman et al. 2010).

***Triatomainfestans* (Klug in Meyen, 1834)**

**Geographic distribution.** South America (Jurberg and Galvão 2006).

**Natural hosts.** Various mammals. Zoonotic on humans (Jurberg and Galvão 2006).

**Route of infection.** Exposure to nymphs and adults in the environment.

**Site in human host.** Skin.

**Vectored pathogens.***Trypanosomacruzi* (Chagas disease, American trypanosomiasis) (Jurberg and Galvão 2006).

***Triatomalectularia* (Stål, 1859)**

**Geographic distribution.** Southern and southeastern USA, Mexico (Bern et al. 2011).

**Natural hosts.** Various mammals. Zoonotic on humans (Bern et al. 2011).

**Route of infection.** Exposure to nymphs and adults in the environment.

**Site in human host.** Skin.

**Vectored pathogens.** None (*T.lectularia* has been found to be naturally infected with *Trypanosomacruzi* but its role as a natural vector for human disease is not fully understood) (Bern et al. 2011).

***Triatomaprotracta* (Uhler, 1894)**

**Geographic distribution.** Southwestern USA, Mexico (Bern et al. 2011).

**Natural hosts.** Woodrats (*Neotoma*). Zoonotic on humans (Bern et al. 2011).

**Route of infection.** Exposure to nymphs and adults in the environment.

**Site in human host.** Skin.

**Vectored pathogens.** None (*T.protracta* has been found to be naturally infected with *Trypanosomacruzi* but its role as a natural vector for human disease is not fully understood) (Bern et al. 2011).

***Triatomapseudomaculata* Correa & Espínola, 1964**

**Geographic distribution.** Brazil (Ribeiro et al. 2019; Soares et al. 2000).

**Natural hosts.** Various mammals. Zoonotic on humans (Soares et al. 2000).

**Route of infection.** Exposure to nymphs and adults in the environment.

**Site in human host.** Skin.

**Vectored pathogens.***Trypanosomacruzi* (Chagas disease, American trypanosomiasis) (Soares et al. 2000; Ribeiro et al. 2019).

***Triatomarecurva* (Stål, 1868)**

**Geographic distribution.** Southwestern USA, Mexico (Bern et al. 2011).

**Natural hosts.** Rodents. Zoonotic on humans (Bern et al. 2011).

**Route of infection.** Exposure to nymphs and adults in the environment.

**Site in human host.** Skin.

**Vectored pathogens.** None (*T.recurva* has been found to be naturally infected with *Trypanosomacruzi* but its role as a natural vector for human disease is not fully understood) (Bern et al. 2011).

***Triatomarubida* (Uhler, 1894)**

**Geographic distribution.** Southwestern USA, Central America (Bern et al. 2011).

**Natural hosts.** Rodents. Zoonotic on humans (Bern et al. 2011).

**Route of infection.** Exposure to nymphs and adults in the environment.

**Site in human host.** Skin.

**Vectored pathogens.** None (*T.rubida* has been found to be naturally infected with *Trypanosomacruzi* but its role as a natural vector for human disease is not fully understood) (Reisenman et al. 2010; Bern et al. 2011).

***Triatomarubrofasciata* (De Geer, 1773)**

**Geographic distribution.** Eastern North America, Southeast Asia, Pacific Islands (Bern et al. 2011; Huang et al. 2018).

**Natural hosts.** Rodents. Zoonotic on humans (Bern et al. 2011).

**Route of infection.** Exposure to nymphs and adults in the environment.

**Site in human host.** Skin.

**Vectored pathogens.** None; implicated in anaphylactic shock from bites (Huang et al. 2018).

***Triatomasanguisuga* (LeConte, 1855)**

**Geographic distribution.** Eastern United States (Bern et al. 2011).

**Natural hosts.** Several groups of mammals, birds, reptiles, and amphibians. Zoonotic on humans (Bern et al. 2011).

**Route of infection.** Exposure to nymphs and adults in the environment.

**Site in human host.** Skin.

**Vectored pathogens.** None.

***Triatomasordida* (Stål, 1859)**

**Geographic distribution.** South America (Crocco and Catalá 1997; Ribeiro et al. 2019).

**Natural hosts.** Various mammals. Zoonotic on humans (Crocco and Catalá 1997).

**Route of infection.** Exposure to nymphs and adults in the environment.

**Site in human host.** Skin

**Vectored pathogens.***Trypanosomacruzi* (Chagas Disease, American trypanosomiasis) (Ribeiro et al. 2019).

●●●●●●●●●●●● **Cimicidae Latreille, 1802**

**Genus *Cimex* Linnaeus, 1758**

***Cimexadjunctus* Barber, 1939**

**Geographic distribution.** Eastern North America (Goddard 2012).

**Natural hosts.** Bats. Zoonotic on humans as incidental hosts (Goddard 2012).

**Route of infection.** Exposure to nymphs and adults in the environment.

**Site in human host.** Skin.

**Vectored pathogens.** None.

***Cimexhemipterus* (Fabricius, 1802)**

**Geographic distribution.** Circumtropical (Usinger 1966; Mathison and Pritt 2021).

**Natural hosts.** Humans (Usinger 1966; Mathison and Pritt 2021).

**Route of infection.** Exposure to nymphs and adults in the environment.

**Site in human host.** Skin.

**Vectored pathogens.** None.

***Cimexlectularius* Linnaeus, 1758**

**Geographic distribution.** Worldwide (Usinger 1966; Mathison and Pritt 2021).

**Natural hosts.** Humans (Usinger 1966; Mathison and Pritt 2021).

**Route of infection.** Exposure to nymphs and adults in the environment.

**Site in human host.** Skin.

**Vectored pathogens.** None.

***Cimexpilosellus* (Horváth, 1910)**

**Geographic distribution.** Western North America (Goddard 2012).

**Natural hosts.** Bats. Zoonotic on humans as incidental hosts (Goddard 2012).

**Route of infection.** Exposure to nymphs and adults in the environment.

**Site in human host.** Skin.

**Vectored pathogens.** None.

***Cimexpipistrelli* Jenyns, 1839**

**Geographic distribution.** Europe and Central Asia (Goddard 2012).

**Natural hosts.** Bats. Zoonotic on humans as incidental hosts (Goddard 2012).

**Route of infection.** Exposure to nymphs and adults in the environment.

**Site in human host.** Skin.

**Vectored pathogens.** None.

**Genus *Leptocimex* Roubaud, 1913**

***Leptocimexboueti* (Brumpt, 1910)**

**Geographic distribution.** Africa (Miller 2008).

**Natural hosts.** Bats. Zoonotic on humans as incidental hosts (Miller 2008).

**Route of infection.** Exposure to nymphs and adults in the environment.

**Site in human host.** Skin.

**Vectored pathogens.** None.

**Genus *Haematosiphon* Champion, 1900**

***Haematosiphoninodora* (Dugès, 1892)**

**Geographic distribution.** North and Central America (Mullen and Durden 2019).

**Natural hosts.** Birds, primarily poultry. Zoonotic on humans as incidental hosts (Mullen and Durden 2019).

**Route of infection.** Exposure to nymphs and adults in the environment.

**Site in human host.** Skin.

**Vectored pathogens.** None.

**Genus *Hesperocimex* List, 1925**

***Hesperocimexcoloradensis* List, 1925**

**Geographic distribution.** North and Central America (Mullen and Durden 2019).

**Natural hosts.** Birds. Zoonotic on humans as incidental hosts (Mullen and Durden 2019).

**Route of infection.** Exposure to nymphs and adults in the environment.

**Site in human host.** Skin.

**Vectored pathogens.** None.

**Genus *Oeciacus* Stål, 1873**

***Oeciacusvicarius* Horváth, 1912**

**Geographic distribution.** North America (Mullen and Durden 2019).

**Natural hosts.** Birds, primarily chimney swifts (*Chaetura*). Zoonotic on humans as incidental hosts (Mullen and Durden 2019).

**Route of infection.** Exposure to nymphs and adults in the environment.

**Site in human host.** Skin.

**Vectored pathogens.** None.

●●●●●●●●●●● **Psocodea Hennig, 1966**

Anoplura Leach, 1815

Phthiraptera Haeckel, 1896

●●●●●●●●●●●● **Pediculidae Leach, 1817**

**Genus *Pediculus* Linnaeus, 1758**

***Pediculushumanuscapitis* De Geer, 1778**

**Geographic distribution.** Worldwide (Veracx and Raoult 2012; Meister and Ochsendorf 2016).

**Natural hosts.** Humans (Veracx and Raoult 2012; Bonilla et al. 2013).

**Route of infection.** Direct person-to-person contact, contaminated fomites (Veracx and Raoult 2012; Bonilla et al. 2013).

**Site in human host.** Hair shafts, commonly on the scalp (Veracx and Raoult 2012; Bonilla et al. 2013; Meister and Ochsendorf 2016).

**Vectored pathogens.** None.

***Pediculushumanushumanus* Linnaeus, 1758**

*Pediculuscorporis* De Geer, 1778

**Geographic distribution.** Worldwide (Veracx and Raoult 2012).

**Natural hosts.** Humans (Veracx and Raoult 2012; Bonilla et al. 2013).

**Route of infection.** Direct person-to-person contact, contaminated fomites (Bonilla et al. 2013; Veracx and Raoult 2012).

**Site in human host.** Skin; *P.h.humanus* primarily lives off the human host and only migrates over to feed (Bonilla et al. 2013).

**Vectored pathogens.***Rickettsiaprowazekii* (epidemic typhus), *Bartonellaquintana* (trench fever), *Borreliarecurrentis* (louse-borne relapsing fever) (Bonilla et al. 2013).

●●●●●●●●●●●● **Pthiridae Ewing, 1929**

**Genus *Pthirus* Leach, 1815**

*Phthirus*, misspelling

***Pthiruspubis* (Linnaeus, 1758)**

**Geographic distribution.** Worldwide (Anderson and Chaney 2009).

**Natural hosts.** Humans (Anderson and Chaney 2009).

**Route of infection.** Direct person-to-person contact (Anderson and Chaney 2009).

**Site in human host.** Hair shafts, usually coarse hair (pubic hair, eyebrows, eye lashes, facial hair) (Anderson and Chaney 2009).

**Vectored pathogens.** None.

●●●●●●●●●●● **Coleoptera Linnaeus, 1758**

●●●●●●●●●●●● **Ptinidae Latreille, 1802**

**Genus *Stegobium* Motschulsky, 1860**

***Stegobiumpaniceum* (Linnaeus, 1758)**

**Geographic distribution.** Worldwide (Bousquet 1990).

**Natural hosts.** None; causes facultative canthariasis (infestation with beetles) in humans (Smadi et al. 2014).

**Route of infection.** Contamination from the environment (Smadi et al. 2014).

**Site in human host.** Skin (Smadi et al. 2014).

**Vectored pathogens.** None.

**Notes.** This species was reported from the skin of a patient with lupus after treatment for facultative myiasis caused by *Luciliasericata* (Smadi et al. 2014). This appears to represent facultative canthariasis, with the beetles feeding on dead and/or dried skin.

**Genus *Trogoderma* Dejean, 1821**

**Geographic distribution.** Worldwide (Bousquet 1990).

**Natural hosts.** None; causes facultative canthariasis in humans (Smadi et al. 2014).

**Route of infection.** Contamination from the environment (Smadi et al. 2014).

**Site in human host.** Skin (Smadi et al. 2014).

**Vectored pathogens.** None

**Notes.** A member of this genus was reported from the skin of a patient with lupus after treatment for facultative myiasis caused by *Luciliasericata* (Smadi et al. 2014). This appears to represent facultative canthariasis, with the beetles feeding on dead and/or dried skin.

●●●●●●●●●●●● **Tenebrionidae Latreille, 1802**

**Genus *Tenebrio* Linnaeus, 1758**

***Tenebriomolitor* Linnaeus, 1758**

**Geographic distribution.** Worldwide (Bousquet 1990).

**Natural hosts.** None; causes facultative canthariasis in humans (Rodriguez-Morales 2018).

**Route of infection.** Contamination from the environment (Rodriguez-Morales 2018).

**Site in human host.** Skin ulcer (Rodriguez-Morales 2018).

**Vectored pathogens.** None.

**Notes.***Tenebriomolitor* is a cosmopolitan pest of stored grains and other foodstuffs. A larva of *T.molitor* was discovered in a skin ulcer of an AIDS patient in rural Colombia (Rodriguez-Morales 2018). This case represents facultative canthariasis. Previous reports of *T.molitor* from the human intestinal tract (Palmer 1946) or urinary tract (Aelami et al. 2019) probably represents spurious passage, environmental contamination, or urethral sounding.

●●●●●●●●●●● **Siphonaptera Latreille, 1824**

●●●●●●●●●●●● **Heteropsyllidae Baker, 1904**

**Genus *Tunga* Jarocki, 1838**

***Tungapenetrans* (Linnaeus, 1758)**

**Geographic distribution.** Central and South America, Caribbean, Africa, Madagascar, Central Asia (Feldmeier et al. 2014).

**Natural hosts.** Many mammals. Zoonotic on humans (Feldmeier et al. 2014).

**Route of infection.** Contact with contaminated soil (e.g., walking barefoot) (Feldmeier et al. 2014).

**Site in human host.** Subcutaneous, especially on the bottom of feet and between toes (Feldmeier et al. 2014).

**Vectored pathogens.** None.

***Tungatrimamillata* Pampiglione et al., 2002**

**Geographic distribution.** South America (Peru, Bolivia, Brazil) (Feldmeier et al. 2014).

**Natural hosts.** Mammals, primarily goats, cattle, pigs. Zoonotic on humans (Feldmeier et al. 2014).

**Route of infection.** Contact with contaminated soil (e.g., walking barefoot) (Feldmeier et al. 2014).

**Site in human host.** Subcutaneous, especially on the bottom of feet and between toes (Feldmeier et al. 2014).

**Vectored pathogens.** None.

●●●●●●●●●●●● **Ceratophyllidae Dampf, 1908**

**Genus *Nodopsyllus* Jordan, 1933**

***Nodopsyllusfasciatus* (Bosc, 1800)**

**Geographic distribution.** Worldwide (Bitam et al. 2010).

**Natural hosts.** Rodents. Zoonotic on humans (Bitam et al. 2010).

**Route of infection.** Exposure to adults in the environment.

**Site in human host.** Skin.

**Vectored pathogens.***Hymenolepisnana* (dwarf tapeworm disease), *H.diminuta* (rate tapeworm disease), *Yersiniapestis* (plague) (Bitam et al. 2010).

**Genus *Oropsylla* Wagner & Ioff, 1926**

***Oropsyllamontana* (Baker, 1895)**

**Geographic distribution.** Western North America (Hinnebusch et al. 2017).

**Natural hosts.** Rodents, primarily prairie dogs and ground squirrels. Zoonotic on humans (Hinnebusch et al. 2017).

**Route of infection.** Exposure to adults in the environment.

**Site in human host.** Skin.

**Vectored pathogens.***Yersiniapestis* (plague) (Hinnebusch et al. 2017).

●●●●●●●●●●●● **Pulicidae Billberg, 1820**

**Genus *Ctenocephalides* Stiles & Collins, 1930**

***Ctenocephalidescanis* (Curtis, 1826)**

**Geographic distribution.** Worldwide (Mathison and Pritt 2014).

**Natural hosts.** Wild and domestic canids and felids. Zoonotic on humans (Mathison and Pritt 2014).

**Route of infection.** Exposure to adults in the environment.

**Site in human host.** skin.

**Vectored pathogens.***Dipylidiumcaninum* (dog tapeworm disease), *Hymenolepisnana* (dwarf tapeworm disease), *H.diminuta* (rat tapeworm disease) (Mathison and Pritt 2014).

***Ctenocephalidesfelis* (Bouché, 1835)**

**Geographic distribution.** Worldwide (Mathison and Pritt 2014).

**Natural hosts.** Wild and domestic canids and felids. Zoonotic on humans (Mathison and Pritt 2014).

**Route of infection.** Exposure to adults in the environment.

**Site in human host.** skin.

**Vectored pathogens.***Dipylidiumcaninum* (dog tapeworm disease), *Hymenolepisnana* (dwarf tapeworm disease), *H.diminuta* (rate tapeworm disease), *Bartonellahenselae* (cat-scratch disease), *Rickettsiafelis* (feline rickettsiae), *Acanthocheilonemareconditum* (ocular filariasis) (Orihel and Eberhard 1998; Mathison and Pritt 2014).

**Genus *Echidnophaga* Olliff, 1886**

***Echidnophagagallinacea* (Westwood, 1875)**

**Geographic distribution.** Worldwide (Carlson and Fox 2009).

**Natural hosts.** Many birds and mammals. Zoonotic on humans (Carlson and Fox 2009).

**Route of infection.** Exposure to adults in the environment; exposure to infected birds (Carlson and Fox 2009).

**Site in human host.** Skin.

**Vectored pathogens.** None.

**Genus *Synopsyllus* Wagner & Roubaud, 1932**

***Synopsyllusfonquerniei* Wagner & Roubaud, 1932**

**Geographic distribution.** Madagascar (Kreppel et al. 2016).

**Natural hosts.** Rats. Zoonotic on humans (Kreppel et al. 2016).

**Route of infection.** Exposure to adults in the environment.

**Site in human host.** Skin.

**Vectored pathogens.***Yersiniapestis* (plague) (Kreppel et al. 2016).

**Genus *Pulex* Linnaeus, 1758**

***Pulexirritans* Linnaeus, 1758**

**Geographic distribution.** Worldwide (Mathison and Pritt 2014).

**Natural hosts.** Many mammals, including humans (Mathison and Pritt 2014).

**Route of infection.** Exposure to adults in the environment.

**Site in human host.** Skin.

**Vectored pathogens.***Dipylidiumcaninum* (dog tapeworm disease), *Hymenolepisnana* (dwarf tapeworm disease), *H.diminuta* (rate tapeworm disease) (Mathison and Pritt 2014).

**Genus *Xenopsylla* Glinkiewicz, 1907**

***Xenopsyllabrasiliensis* (Baker, 1904)**

**Geographic distribution.** Africa, South America, India (Miarinjara et al. 2016; Mohammadi et al. 2021).

**Natural hosts.** Rats. Zoonotic on humans (Miarinjara et al. 2016; Mohammadi et al. 2021).

**Route of infection.** Exposure to adults in the environment.

Site in human host: Skin.

Vectored pathogens. *Yersiniapestis* (plague) (Miarinjara et al. 2016; Mohammadi et al. 2021).

***Xenopsyllacheopis* (Rothschild, 1903)**

**Geographic distribution.** Worldwide (Perry and Fetherston 1997).

**Natural hosts.** Mammals, primarily rodents (rats). Zoonotic on humans (Perry and Fetherston 1997).

**Route of infection.** Exposure to adults in the environment.

**Site in human host.** Skin.

**Vectored pathogens.***Yersiniapestis* (plague), *Rickettsiatyphi* (murine (epidemic) typhus) (Mathison and Pritt 2014; Perry and Fetherston 1997).

●●●●●●●●●●● **Diptera Linnaeus, 1758**

●●●●●●●●●●●● **Phoridae Curtis, 1833**

**Genus *Megaselia* Róndani, 1856**

***Megaseliascalaris* Loew, 1866**

**Geographic distribution.** Worldwide (Disney 2008).

**Natural hosts.** None, agent of facultative and incidental myiasis in humans (Disney 2008).

**Route of infection.** Oviposition on pre-existing wounds (Disney 2008).

**Site in human host.** Soft tissues, pre-existing wounds (Hira et al. 2004; Disney 2008).

**Notes.** Although this species is known to cause facultative myiasis is pre-existing wounds of soft tissues and the upper respiratory tract (Hira et al. 2004; Disney 2008), reports from urine (Ghavami and Djalilvand 2015) should be met with caution.

●●●●●●●●●●●● **Muscidae Latreille, 1802**

**Genus *Musca* Linnaeus, 1758**

***Muscadomestica* Linnaeus, 1758**

*Muscanebulo* Fabricius, 1794

*Muscahottentota* Robineau-Desvoidy, 1830

*Muscafrontalis* Macquart, 1843

*Muscasenegalensis* Macquart, 1843

*Muscasantae-helenae* Macquart, 1848

*Muscagymnosomea* Róndani, 1862

*Muscaniveisquama* Thomson, 1869

*Muscacurviforceps* Saccà & Rivosechi, 1956

**Geographic distribution.** Worldwide (Francesconi and Lupi 2012).

**Natural hosts.** None, agent of facultative and incidental myiasis in humans (Francesconi and Lupi 2012).

**Route of infection.** Oviposition on pre-existing wounds (Francesconi and Lupi 2012).

**Site in human host.** Skin, soft tissues, pre-existing wounds (Francesconi and Lupi 2012).

●●●●●●●●●●●● **Fanniidae Schnabl & Dziedzicki, 1911**

**Genus *Fannia* Robineau-Desvoidy, 1830**

***Fanniacanicularis* (Linnaeus, 1761)**

**Geographic distribution.** Worldwide (Francesconi and Lupi 2012).

**Natural hosts.** None, agent of facultative and incidental myiasis in humans (Francesconi and Lupi 2012).

**Route of infection.** Oviposition on pre-existing wounds (Francesconi and Lupi 2012).

**Site in human host.** Soft tissues, pre-existing wounds (Francesconi and Lupi 2012).

●●●●●●●●●●●● **Calliphoridae Brauer & Bergenstamm, 1889**

**Genus *Auchmeromyia* Brauer & Bergenstamm, 1891**

***Auchmeromyiasenegalensis* Macquart, 1851**

**Geographic distribution.** Sub-Saharan Africa, Cape Verde Islands (Francesconi and Lupi 2012).

**Natural hosts.** Several mammals, including humans (Francesconi and Lupi 2012).

**Route of infection.** Exposure to larvae in the environment (Francesconi and Lupi 2012).

**Site in human host.** Skin (sanguinivorous) (Francesconi and Lupi 2012).

**Genus *Calliphora* Robineau-Desvoidy, 1820**

***Calliphorahilli* Patton, 1925**

**Geographic distribution.** Australia (Francesconi and Lupi 2012).

**Natural hosts.** None; causes facultative myiasis in humans (Francesconi and Lupi 2012).

**Route of infection.** Oviposition on pre-existing wounds (Francesconi and Lupi 2012).

**Site in human host.** Eye (Francesconi and Lupi 2012).

***Calliphoravicina* Robineau-Desvoidy, 1830**

**Geographic distribution.** Worldwide (Francesconi and Lupi 2012).

**Natural hosts.** None; causes facultative myiasis in humans (Francesconi and Lupi 2012).

**Route of infection.** Oviposition on pre-existing wounds (Francesconi and Lupi 2012).

**Site in human host.** Skin, soft tissues, pre-existing wounds (Francesconi and Lupi 2012).

**Genus *Cochliomyia* Townsend, 1915**

***Cochliomyiahominovorax* (Coquerel, 1858)**

**Geographic distribution.** Central and South America, Caribbean (Scott et al. 2017; Parker et al. 2020).

**Natural hosts.** Most mammals, including humans (Scott et al. 2017; Parker et al. 2020).

**Route of infection.** Oviposition on pre-existing wounds, ears, nose, oral cavity (Francesconi and Lupi 2012).

**Site in human hose.** Skin, soft tissues, pre-existing wounds, ears, nose, oral cavity (Francesconi and Lupi 2012).

**Genus *Chrysomya* Robineau-Desvoidy, 1830**

***Chrysomyaalbiceps* (Wiedemann, 1819)**

**Geographic distribution.** Worldwide (Francesconi and Lupi 2012).

**Natural hosts.** None; causes facultative myiasis in humans and sheep (Francesconi and Lupi 2012).

**Route of infection.** Oviposition on pre-existing wounds (Francesconi and Lupi 2012).

**Site in human host.** Skin, soft tissues, pre-existing wounds (Francesconi and Lupi 2012).

***Chrysomyabezziana* (Villeneuve, 1914)**

**Geographic distribution.** India, Arabian Peninsula, Indonesia, Philippines, New Guinea (Francesconi and Lupi 2012).

**Natural hosts.** Sheep. Zoonotic on humans as incidental hosts (Francesconi and Lupi 2012).

**Route of infection.** Oviposition on pre-existing wounds (Francesconi and Lupi 2012).

**Site in human host.** Skin, soft tissues, pre-existing wounds (Francesconi and Lupi 2012).

***Chrysomyamegacephala* (Fabricius, 1794)**

**Geographic distribution.** Worldwide (Francesconi and Lupi 2012).

**Natural hosts.** None; causes facultative myiasis in humans and other mammals (Francesconi and Lupi 2012).

**Route of infection.** Oviposition on pre-existing wounds (Francesconi and Lupi 2012).

**Site in human host.** Skin, soft tissues, pre-existing wounds (Francesconi and Lupi 2012).

***Chrysomyarufifacies* (Macquart, 1842)**

**Geographic distribution.** Worldwide (Francesconi and Lupi 2012).

**Natural hosts.** None; causes facultative myiasis in humans and other mammals (Francesconi and Lupi 2012).

**Route of infection.** Oviposition on pre-existing wounds (Francesconi and Lupi 2012).

**Site in human host.** Skin, soft tissues, pre-existing wounds (Francesconi and Lupi 2012).

**Genus *Cordylobia* Gruenberg, 1903**

***Cordylobiaanthropophaga* Blanchard, 1872**

**Geographic distribution.** East, Central, and West Africa (Francesconi and Lupi 2012).

**Natural hosts.** Mammals, including rodents, monkeys, ruminants, and humans (Francesconi and Lupi 2012).

**Route of infection.** Exposure to first-instar larvae on fomites (soil, air-drying laundry) (Francesconi and Lupi 2012).

**Site in human host.** Skin (Francesconi and Lupi 2012).

***Cordylobiarodhaini* Gedoelst, 1910**

**Geographic distribution.** Tropical Africa (Pezzi et al. 2015).

**Natural hosts.** Mammals, primarily rodents. Zoonotic on humans (Pezzi et al. 2015).

**Route of infection.** Exposure to larvae on fomites (soil, air-drying laundry) (Pezzi et al. 2015).

**Site in human host.** Skin (Pezzi et al. 2015).

**Genus *Lucilia* Robineau-Desvoidy, 1830**

***Luciliacuprina* (Wiedemann, 1830)**

**Geographic distribution.** Nearly worldwide, more common in warm climates (Francesconi and Lupi 2012).

**Natural hosts.** None, causes facultative myiasis in sheep and humans (Francesconi and Lupi 2012).

**Route of infection.** Oviposition on pre-existing wounds (Francesconi and Lupi 2012).

**Site in human host.** Skin, soft tissues, pre-existing wounds (Francesconi and Lupi 2012).

***Luciliasericata* (Meigan, 1826)**

**Geographic distribution.** Europe, North, Central, and South America (Francesconi and Lupi 2012).

**Natural hosts.** None, causes facultative myiasis in sheep and humans (Francesconi and Lupi 2012).

**Route of infection.** Oviposition on pre-existing wounds (Francesconi and Lupi 2012).

**Site in human host.** Skin, soft tissues, pre-existing wounds (Francesconi and Lupi 2012).

**Vectored pathogens.***Wohlfahrtiimonaschitiniclastica*, *Ignatzschineriaindica* (Lysaght et al. 2018).

**Genus *Phormia* Robineau-Desvoidy, 1830**

***Phormiaregina* (Meigen, 1826)**

**Geographic distribution.** Northern Hemisphere (Francesconi and Lupi 2012).

**Natural hosts.** None, causes facultative myiasis in humans (Francesconi and Lupi 2012).

**Route of infection.** Oviposition on pre-existing wounds (Francesconi and Lupi 2012).

**Site in human host.** Skin, soft tissues, pre-existing wounds (Francesconi and Lupi 2012).

**Genus *Protophormia* Townsend, 1908**

***Protophormiaterraenovae* Robineau-Desvoidy, 1830**

**Geographic distribution.** Northern Hemisphere (Francesconi and Lupi 2012).

**Natural hosts.** None, causes facultative myiasis in humans (Francesconi and Lupi 2012).

**Route of infection.** Oviposition on pre-existing wounds (Francesconi and Lupi 2012).

**Site in human host.** Skin, soft tissues, pre-existing wounds (Francesconi and Lupi 2012).

●●●●●●●●●●●● **Sarcophagidae Macquart, 1834**

**Genus *Peckia* Robineau-Desvoidy, 1830**

***Peckialambens* (Wiedemann, 1830)**

**Geographic distribution.** Central and South America, Caribbean (Fernandes et al. 2009).

**Natural hosts.** None; cause facultative myiasis in humans (Fernandes et al. 2009).

**Route of infection.** Oviposition on pre-existing wounds (Fernandes et al. 2009).

**Site in human host.** Skin and soft tissues (Fernandes et al. 2009).

**Genus *Sarcophaga* Meigen, 1826**

***Sarcophagadux* Thomson, 1869**

**Geographic distribution.** Worldwide (Sukontason et al. 2014).

**Natural hosts.** None, causes facultative myiasis in humans (Sukontason et al. 2014).

**Route of infection.** Oviposition on pre-existing wounds, ear (Sukontason et al. 2014).

**Site in human host.** Ear, skin and soft tissues (Chaiwong et al. 2014; Sukontason et al. 2014).

***Sarcophagaperegrina* (Robineau-Desvoidy, 1830)**

*Sarcophagafucicauda* Böttcher, 1912

*Sarcophagahutsoni* Parker, 1923

*Sarcophagameriana* Zumpt, 1951

**Geographic distribution.** Asia, Africa, Australia, New Zealand (Kim et al. 2018).

**Natural hosts.** None, causes facultative myiasis in humans (Kim et al. 2018).

**Route of infection.** Oviposition on pre-existing wounds, ear, eye (Chigusa et al. 2000; Miura et al. 2005).

**Site in human host.** Nose, oral cavity, eye (Chigusa et al. 2005; Miura et al. 2005).

***Sarcophagapernix* Harris, 1789**

*Muscahaemorrhoidalis* Fallén, 1817

**Geographic distribution.** Europe, Asia, North Africa (Francesconi and Lupi 2012).

**Natural hosts.** None, causes facultative myiasis in humans (Francesconi and Lupi 2012).

**Route of infection.** Oviposition on pre-existing wounds, ear (Braverman et al. 1994).

**Site in human host.** Ear, skin and soft tissues (Braverman et al. 1994).

***Sarcophagaruficornis* (Fabricius, 1794)**

**Geographic distribution.** Worldwide (Suwannayod et al. 2013).

**Natural hosts.** None, causes facultative myiasis in humans (Suwannayod et al. 2013).

**Route of infection.** Oviposition on pre-existing wounds (Suwannayod et al. 2013).

**Site in human host.** Skin and soft tissues (Suwannayod et al. 2013).

***Sarcophagaseptentrionalis* (Rohdendorf, 1937)**

**Geographic distribution.** East Asia (Chigusa et al. 2006).

**Natural hosts.** None; causes facultative myiasis in humans (Chigusa et al. 2006)

**Route of infection.** Oviposition on pre-existing wounds (Chigusa et al. 2006).

**Site in human host.** Oral cavity (Chigusa et al. 2006).

**Genus *Wohlfahrtia* Brauer & von Bergenstamm, 1889**

***Wohlfahrtiamagnifica* (Schiner, 1861)**

**Geographic distribution.** Mediterranean Europe, North Africa, Central and Southeast Asia, Russia (Francesconi and Lupi 2012).

**Natural hosts.** Mammals and birds, including sheep, cattle, poultry, and humans (Francesconi and Lupi 2012).

**Route of infection.** Larviposition on wounds, tissues (Francesconi and Lupi 2012).

**Site in human host.** Skin, soft tissues, pre-existing wounds, oral cavity, ears (Francesconi and Lupi 2012).

**Vectored pathogens.***Wohlfahrtiimonaschitiniclastica*, *Ignatzschineriaindica* (Barker et al. 2014).

***Wohlfahrtiaopaca* (Coquillett, 1897)**

**Geographic distribution.** Western North America (Francesconi and Lupi 2012).

**Natural hosts.** Wild and domestic canids and felids, rabbits, mustelids. Zoonotic in human as incidental hosts (Francesconi and Lupi 2012).

**Route of infection.** Larviposition on wounds, tissues (Francesconi and Lupi 2012).

**Site in human host.** Skin and soft tissues, pre-existing wounds (Francesconi and Lupi 2012).

***Wohlfahrtiavigil* (Walker, 1849)**

**Geographic distribution.** Eastern North America, southern Europe, Russia, Pakistan (Francesconi and Lupi 2012).

**Natural hosts.** Wild and domestic canids and felids, rabbits, mustelids. Zoonotic in human as incidental hosts (Francesconi and Lupi 2012).

**Route of infection.** Larviposition on wounds, tissues (Francesconi and Lupi 2012).

**Site in human host.** Skin and soft tissues, pre-existing wounds (Francesconi and Lupi 2012).

●●●●●●●●●●●● **Oestridae Leach, 1815**

**Genus *Alouattamyia* Townsend, 1931**

***Allouattamyiabaeri* (Shannon & Green, 1926)**

**Geographic distribution.** South America (Francesconi and Lupi 2012).

**Natural hosts.** Non-human primates. Zoonotic in human as incidental hosts (Francesconi and Lupi 2012).

**Route of infection.** Exposure to first-instar larvae in the environment (Francesconi and Lupi 2012).

**Site in human host.** Upper respiratory tract (Francesconi and Lupi 2012).

**Genus *Dermatobia* Brauer, 1860**

***Dermatobiahominis* (Linnaeus in Pallas, 1781)**

**Geographic distribution.** Central and South America, Caribbean (Francesconi and Lupi 2012).

**Natural hosts.** Many mammals, including humans, and birds; vectored by mosquitoes and ticks (Francesconi and Lupi 2012).

**Route of infection.** Bite of a mosquito or tick, the phoretic host for *D.hominis* eggs (Francesconi and Lupi 2012).

**Site in human host.** Skin (Francesconi and Lupi 2012).

**Genus *Cuterebra* Clark, 1815**

**Geographic distribution.** North and Central America (Francesconi and Lupi 2012).

**Natural hosts.** Rodents and lagomorphs; cats and other mammals can serve as reservoir hosts. Zoonotic in human as incidental hosts (Francesconi and Lupi 2012).

**Route of infection.** Exposure to first-instar larvae in contaminated soil, litter, etc.

**Site in human host.** Skin (second-instar, third-instar larvae), viscera and lungs (third-instar larvae) (Cornet et al. 2003; Delshad et al. 2008; Francesconi and Lupi 2012; Hale et al. 2019).

**Notes.** Cases of human infestation with *Cuterebra* are usually not identified to the species level.

**Genus *Gasterophilus* Leach, 1817**

**Geographic distribution.** Worldwide (Zumpt 1965; Francesconi and Lupi 2012).

**Natural hosts.** Horses, zebras, elephants, rhinoceroses. Zoonotic in human as incidental hosts (Zumpt 1965; Francesconi and Lupi 2012).

**Route of infection.** Oviposition on skin and mucus membranes (Francesconi and Lupi 2012).

**Site in human host.** Skin, eye, oral cavity (Francesconi and Lupi 2012).

**Notes.** Cases of human infestation with *Gasterophilus* are usually not identified to the species level.

**Genus *Hypoderma* Latreille, 1818**

***Hypodermabovis* (Linnaeus, 1758)**

**Geographic distribution.** North America, Europe, Asia, Africa (Zumpt 1965; Francesconi and Lupi 2012).

**Natural hosts.** Wild and domestic ruminants. Zoonotic in human as incidental hosts (Zumpt 1965; Francesconi and Lupi 2012).

**Route of infection.** Oviposition on hair or skin, or possibly direct contact with first-instar larvae on infected animals (Francesconi and Lupi 2012).

**Site in human host.** Skin (Francesconi and Lupi 2012).

***Hypodermalineatum* (Viller, 1789)**

**Geographic distribution.** North America, Europe, Asia, Africa (Zumpt 1965; Francesconi and Lupi 2012).

**Natural hosts.** Wild and domestic ruminants. Zoonotic in human as incidental hosts (Zumpt 1965; Francesconi and Lupi 2012).

**Route of infection.** Oviposition on hair or skin, or possibly direct contact with first-instar larvae on infected animals (Francesconi and Lupi 2012)

**Site in human host.** Skin (Francesconi and Lupi 2012).

***Hypodermatarandi* (Linnaeus, 1758)**

**Geographic distribution.** Northern North America, Northern Eurasia (Zumpt 1965; Francesconi and Lupi 2012).

**Natural hosts.** Caribou. Zoonotic in human as incidental hosts (Zumpt 1965; Francesconi and Lupi 2012).

**Route of infection.** Oviposition on hair, skin, or mucus membranes, or possibly direct contact with first-instar larvae on infected animals (Francesconi and Lupi 2012).

**Site in human host.** Skin, eyes, oral cavity (Francesconi and Lupi 2012).

**Genus *Oestrus* Linnaeus, 1758**

***Oestrusovis* Linnaeus, 1758**

**Geographic distribution.** Worldwide (Francesconi and Lupi 2012).

**Natural hosts.** Sheep, goats, other ruminants. Zoonotic in human as incidental hosts (Francesconi and Lupi 2012).

**Route of infection.** Larviposition on eyes and mucus membranes (Francesconi and Lupi 2012).

**Site in human host.** Eye, throat (Francesconi and Lupi 2012).

●●●●● **Nematoda Diesking, 1861**

●●●●●● **Dorylaimia Inglis, 1983**

●●●●●●● **Dioctophymatida Baylis & Daubney, 1926**

●●●●●●●● **Dioctophymidae Railliet, 1915**

**Genus *Dioctoyphyme* Collet-Meygret, 1802**

*Dioctophyma*, misspelling

***Dioctophymerenale* (Goeze, 1782)**

**Geographic distribution.** Worldwide (Angelou et al. 2020)

**Natural hosts.** First intermediate hosts are annelids. Second intermediate and paratenic hosts are primarily freshwater fish and amphibians. Definitive hosts are carnivores, including mustelids and canids. Zoonotic in humans, harboring L3 larvae or adults (Angelou et al. 2020).

**Route of infection.** Ingestion of L3 larvae in undercooked paratenic hosts (Orihel and Ash 1995).

**Site in human host.** Subcutaneous (L3 larvae), kidneys (adults) (Orihel and Ash 1995).

**Genus *Eustrongylides* Jägerskiöld, 1909**

**Geographic distribution.** Worldwide (Anderson 2000).

**Natural hosts.** First intermediate hosts are aquatic annelids. The second intermediate hosts are fish; fish, amphibians, and reptiles can serve as paratenic hosts. Definitive hosts are birds. Zoonotic in humans as dead-end hosts harboring L3 or L4 larvae (Anderson 2000).

**Route of Infection.** Ingestion of L3 or precocious L4 larvae in infected intermediate or paratenic hosts (Anderson 2000).

**Site in human host.** Skin, Abdominal and peritoneal cavities, possible ectopic migration to other parts of the body (Orihel and Ash 1995; Eberhard and Ruiz-Tiben 2014).

**Notes.** There about 20 described species of *Eustrongylides*. Human cases with larval *Eustrongylides* have been reported from North America and East Africa and have not been characterized at the species level.

●●●●●●● **Trichinellida Hall, 1916**

Trichocephalida Spasski, 1954

●●●●●●●● **Anatrichosomatidae Yamaguti, 1961**

**Genus *Anatrichosoma* Smith & Chitwood, 1954**

**Geographic distribution.** Worldwide (Eberhard et al. 2010, 2014a).

**Natural hosts.** The life cycle is not completely understood, and it is not known if there are more than one host. Definitive hosts include non-human primates, tree shrews, rodents, marsupials. Zoonotic in humans as incidental hosts (Eberhard et al. 2010, 2014a).

**Route of infection.** Unknown, possible ingestion of embryonated eggs or unknown intermediate host (Eberhard et al. 2010, 2014a).

**Site in human host.** Skin and soft tissues, oral mucosa (Eberhard et al. 2010, 2014a).

**Notes.** Human cases of anatrichosomiasis have been diagnosed by histopathology. Because *Anatrichosoma* spp. have not been described morphologically by histopathology, it is not currently possible to characterize human isolates at the species level. Given the morphology and epidemiology, cases from the human oral cavity from Mexico and the United States are probably attributable to *A.buccalis* Pence & Little, 1972 (Eberhard et al. 2010, 2014a).

●●●●●●●● **Capillariidae Railliet, 1915**

**Genus *Calodium* Dujardin, 1845**

***Calodiumhepaticum* (Bancroft, 1893)**

**Geographic distribution.** Worldwide (Li et al. 2010).

**Natural hosts.** Rodents. Zoonotic in humans as incidental hosts (Li et al. 2010).

**Route of infection.** Ingestion of embryonated eggs on soil-contaminated fomites (Li et al. 2010).

**Site in human host.** Liver (Li et al. 2010).

**Notes.** Eggs of *C.hepaticum* detected in human stool probably represent spurious passage and not true infection.

**Genus *Eucoleus* Dujardin, 1845**

***Eucoleusaerophilus* (Creplin, 1839)**

**Geographic distribution.** Worldwide (Traversa et al. 2011).

**Natural hosts.** Paratenic hosts are earthworms. Definitive hosts are carnivores. Zoonotic in humans as incidental hosts (Traversa et al. 2011).

**Route of infection.** Ingestion of embryonated eggs or paratenic hosts (Traversa et al. 2011).

**Site in human host.** Lungs (Traversa et al. 2011).

**Genus *Paracapillaria* Chitwood et al., 1968**

***Paracapillariaphilippinensis* (Velasquez et al., 1968)**

**Geographic distribution.** Southeast Asia, Philippines, Egypt, South America (Cross 1992; Attia et al. 2012; Eiras et al. 2018).

**Natural hosts.** Intermediate hosts are freshwater fish. Definitive hosts are humans, fish-eating birds (Cross 1992).

**Route of infection.** Ingestion of L1 larvae in infected intermediate host (Cross 1992).

**Site in human host.** Small intestine (Cross 1992).

●●●●●●●● **Trichinellidae Ward, 1907**

**Genus *Trichinella* Railliet, 1895**

***Trichinellabritovi* Pozio et al., 1992**

*Trichinella* strain T3

**Geographic distribution.** Europe, North America, Asia, West Africa (Gottstein et al. 2009).

**Natural hosts.** Mammals. Zoonotic in humans (Gottstein et al. 2009).

**Route of infection.** Ingestion of L1 larvae in infected hosts; common sources of human infection are wild and domestic pigs, horses, foxes, jackals (Gottstein et al. 2009).

**Site in human host.** Small intestine (adults), skeletal muscle (L1 larvae) (Gottstein et al. 2009).

***Trichinellamurelli* Pozio & La Rosa, 2000**

*Trichinella* strain T5

**Geographic distribution.** North America (Gottstein et al. 2009; Hall et al. 2012).

**Natural hosts.** Mammals, primarily carnivores. Zoonotic in humans (Gottstein et al. 2009).

**Route of infection.** Ingestion of L1 larvae in infected hosts; common sources of human are bears and horses (Gottstein et al. 2009; Hall et al. 2012).

**Site in human host.** Small intestine (adults), skeletal muscle (L1 larvae) (Gottstein et al. 2009).

***Trichinellanativa* Britov & Boev, 1972**

*Trichinella* strain T2

**Geographic distribution.** Circumboreal (Gottstein et al. 2009).

**Natural hosts.** Mammals, primarily carnivores. Zoonotic in humans (Gottstein et al. 2009).

**Route of infection.** Ingestion of L1 larvae in infected hosts; common sources of human infection are bears and walrus (Gottstein et al. 2009; Springer et al. 2017).

**Site in human host.** Small intestine (adults), skeletal muscle (L1 larvae) (Gottstein et al. 2009).

***Trichinellanelsoni* Britov & Boev, 1972**

*Trichinella* strain T7

**Geographic distribution.** Europe, North America, Asia, West Africa (Gottstein et al. 2009).

**Natural hosts.** Mammals. Zoonotic in humans (Gottstein et al. 2009).

**Route of infection.** Ingestion of L1 larvae in infected hosts; common sources of human infection are wild and domestic pigs, horses, foxes, jackals (Gottstein et al. 2009).

**Site in human host.** Small intestine (adults), skeletal muscle (L1 larvae) (Gottstein et al. 2009).

***Trichinellapapuae* Pozio et al., 1999**

**Geographic distribution.** Papua New Guinea, Thailand (Gottstein et al. 2009).

**Natural hosts.** Mammals (primarily pigs), saltwater crocodiles. Zoonotic in humans (Gottstein et al. 2009).

**Route of infection.** Ingestion of L1 larvae in infected hosts; common sources of human infection are wild and domestic pigs (Gottstein et al. 2009).

**Site in human host.** Small intestine (adults), skeletal muscle (L1 larvae) (Gottstein et al. 2009).

***Trichinellapseudospiralis* Garkavi, 1972**

*Trichinella* strain T4

**Geographic distribution.** Worldwide (Gottstein et al. 2009).

**Natural hosts.** Mammals and birds. Zoonotic in humans (Gottstein et al. 2009).

**Route of infection.** Ingestion of L1 larvae in infected hosts; common sources of human infection are wild and domestic pigs (Gottstein et al. 2009).

**Site in human host.** Small intestine (adults), skeletal muscle (L1 larvae) (Gottstein et al. 2009).

***Trichinellaspiralis* (Owen, 1835)**

*Trichinella* strain T1

**Geographic distribution.** Worldwide (Gottstein et al. 2009).

**Natural hosts.** Mammals. Zoonotic in humans (Gottstein et al. 2009).

**Route of infection.** Ingestion of L1 larvae in infected hosts; common sources of human infection are wild and domestic pigs (Gottstein et al. 2009).

**Site in human host.** Small intestine (adults), skeletal muscle (L1 larvae) (Gottstein et al. 2009).

***Trichinella* Strain T6**

**Geographic distribution.** Northern and montane North America (Gottstein et al. 2009).

**Natural hosts.** Mammals, primarily carnivores. Zoonotic in humans (Gottstein et al. 2009).

**Route of infection.** Ingestion of L1 larvae in infected hosts; common sources of human infection are bears, wild felids (Gottstein et al. 2009).

**Site in human host.** Small intestine (adults), skeletal muscle (L1 larvae) (Gottstein et al. 2009).

●●●●●●● **Muspiceida Bain & Chabaud, 1959**

●●●●●●●● **Robertdollfusidae Chabaud & Campana, 1950**

**Genus *Haycocknema* Spratt et al., 1999**

***Haycocknemaperplexum* Spratt et al., 1999**

**Geographic distribution.** Australia (McKelvie et al. 2013; Vos et al. 2016).

**Natural hosts.** Unknown, presumed zoonotic in humans (McKelvie et al. 2013; Vos et al. 2016).

**Route of infection.** Unknown, presumed ingestion of larvae in natural hosts (McKelvie et al. 2013; Vos et al. 2016).

**Site in human host.** Skeletal muscle (McKelvie et al. 2013; Vos et al. 2016).

●●●●●●●● **Trichuridae Ransom, 1911**

**Genus *Trichuris* Roederer, 1761**

***Trichuristrichiura* (Linnaeus, 1771)**

**Geographic distribution.** Worldwide (Betson et al. 2015).

**Natural hosts.** Humans (Betson et al. 2015).

**Route of infection.** Ingestion of embryonated eggs in fecally contaminated food, water, fomites (Betson et al. 2015).

**Site in human host.** Large intestine (Betson et al. 2015).

**Notes.** The zoonotic potential of *T.trichiura* is not well understood and is unclear whether there are multiple species infecting humans or whether other animals can harbor *T.trichiura* and serve as reservoir hosts for human infection (Betson et al. 2015).

***Trichurisvulpis* Froelich, 1789**

**Geographic distribution.** Worldwide (Dunn et al. 2002).

**Natural hosts.** Wild and domestic canids. Zoonotic in humans (Dunn et al. 2002).

**Route of infection.** Ingestion of embryonated eggs (Dunn et al. 2002).

**Site in human host.** Large intestine (Dunn et al. 2002).

**Notes.** Fully-embryonated eggs of *T.vulpis* in human stool specimens may also represent spurious passage and not true infection.

●●●●●● **Chromadoria Inglis, 1983**

●●●●●●● **Spururina Railliet & Henry, 1915**

●●●●●●●● **Ascaridida DeLay & Blaxter, 2002**

●●●●●●●●● **Anisakidae Skrjabin & Karokhin, 1945**

**Genus *Anisakis* Dujardin, 1845**

**Geographic distribution.** Worldwide; human infection more common in coastal areas (Audicana and Kennedy 2008; Mathison and da Silva 2018).

**Natural hosts.** First intermediate hosts are marine microcrustaceans. Paratenic hosts are marine fish and squid. Definitive hosts are fish-eating mammals, primarily cetaceans and pinnipeds. Zoonotic in humans as dead-end hosts harboring L3 larvae (Audicana and Kennedy 2008; Mathison and da Silva 2018).

**Route of infection.** Ingestion of L3 larvae in infected paratenic hosts (Audicana and Kennedy 2008; Mathison and da Silva 2018).

**Site in human host.** Stomach, esophagus, oral cavity, large intestine; ectopic colonization of peritoneal cavity, mesenteries, omentum, mesocolic lymph nodes, spleen, pleural cavity, and parametrium (Audicana and Kennedy 2008; Mathison and da Silva 2018).

**Notes.** While most human cases have been attributed to members of *A.simplex* sensu lato (including *A.simplex* (Rudolphi, 1809), *A.pegreffii* (Campana-Rouget & Biocca, 1955), and *A.berlandi* Mattiucci et al. 2014) or *A.physeteris* (Baylis, 1923), reliable identification human isolates at the species level is difficult and seldom performed due to a lack of species-level morphologic features of the L3 larvae (the stage found in human host) and the lack of a reliable sequencing library for molecular analysis (Umehara et al. 2007).

**Genus *Contracaecum* Railliet & Henry, 1912**

**Geographic distribution.** Worldwide; human infection more common on coastal areas (Mathison and da Silva 2018).

**Natural hosts.** First intermediate hosts are marine microcrustaceans. Paratenic hosts are marine fish and squid. Definitive hosts are fish-eating mammals, primarily cetaceans and pinnipeds, and birds. Zoonotic in humans as dead-end hosts harboring L3 larvae (Mathison and da Silva 2018).

**Route of infection.** Ingestion of L3 larvae in infected paratenic hosts (Mathison and da Silva 2018).

**Site in human host.** Stomach, esophagus, oral cavity, large intestine; ectopic colonization of various organs possible (Mathison and da Silva 2018).

**Notes.** It is not currently possible to reliably identify human isolates of *Contracaecum* at the species level due to a lack of species-level morphologic features of the L3 larvae (the stage found in human host) and the lack of a reliable sequencing library for molecular analysis.

**Genus *Pseudoterranova* Mozgovoi, 1951**

*Phocanema* Myers, 1959

**Geographic distribution.** Worldwide; human infection more common on coastal areas (Mathison and da Silva 2018).

**Natural hosts.** First intermediate hosts are marine microcrustaceans. Paratenic hosts are marine fish and squid. Definitive hosts are fish-eating mammals, primarily cetaceans and pinnipeds. Zoonotic in humans as dead-end hosts harboring L3 larvae (Mathison and da Silva 2018).

**Route of infection.** Ingestion of L3 larvae in infected paratenic hosts (Mathison and da Silva 2018).

**Site in human host.** Stomach, esophagus, oral cavity, large intestine; ectopic colonization of peritoneal cavity, mesenteries, omentum, mesocolic lymph nodes, spleen, pleural cavity, and parametrium (Mathison and da Silva 2018).

**Notes.** While many human cases have been attributed to the *P.decepiens* (Krabbe, 1878) complex, reliable identification human isolates at the species level is difficult and seldom performed due to a lack of species-level morphologic features of the L3 larvae (the stage found in human host) and the lack of a reliable sequencing library for molecular analysis.

●●●●●●●●● **Ascarididae Baird, 1853**

**Genus *Ascaris* Linnaeus, 1758**

***Ascarislumbricoides* Linnaeus, 1758**

*Ascarissuum* (Goeze, 1782)

**Geographic distribution.** Worldwide (Betson et al. 2014).

**Natural hosts.** Pigs. Zoonotic in humans (Betson et al. 2014).

**Route of infection.** Ingestion of embryonated eggs in fecally contaminated food, water, fomites (Betson et al. 2014).

**Site in human host.** Lungs (L3 larvae); Small intestine (adults), with ectopic colonization of liver, spleen, gall bladder (Betson et al. 2014).

**Notes.** The relationship between *A.lumbricoides* and *A.suum* is unresolved. While there are genetic differences among *Ascaris* isolates throughout the world, it is unclear whether such differences represent more than one species with a common ancestor, multiple colonization events between pigs and humans in different geographical areas, or there is one species with geographic variations (Betson et al. 2014). We are maintaining a conservative approach in keeping *A.suum* in synonymy with *A.lumbricoides*, based on ecological, biological, morphological, and molecular data (Leles et al. 2012; Liu et al. 2012).

**Genus *Baylisascaris* Sprent, 1968**

***Baylisascarisprocyonis* (Stefanski & Zarnowski, 1951)**

**Geographic distribution.** North and Central America, introduced to Europe and Asia (Gavin et al. 2005).

**Natural hosts.** Raccoons and other procyonids; dogs can serve as reservoir hosts. Zoonotic in humans as dead-end hosts harboring L3 larvae (Gavin et al. 2005).

**Route of infection.** Ingestion of embryonated eggs in food, water, fomites contaminated with raccoon feces; possibly also ingestion of paratenic hosts (Gavin et al. 2005).

**Site in human host.** Brain, eye, liver, lungs (Gavin et al. 2005).

**Genus *Lagochilascaris* Leiper, 1909**

***Lagochilascarisminor* Leiper, 1909**

**Geographic distribution.** Central and South America (Campos et al. 2017).

**Natural hosts.** Unknown. It has been prosed rodents are intermediate hosts and carnivores are definitive hosts, with humans serving as incidental definitive hosts (Campos et al. 2017).

**Route of infection.** Unknown, presumed to be ingestion of larvae in infected intermediate or paratenic hosts, possibly ingestion of embryonated eggs (Campos et al. 2017).

**Site in human host.** Disseminated infection; common sites of infection are subcutaneous, ear, tonsils, nasal sinuses, and mastoid (Campos et al. 2017).

**Genus *Toxocara* Stiles, 1905**

***Toxocaracanis* (Werner, 1792)**

**Geographic distribution.** Worldwide (Despommier 2003).

**Natural hosts.** Wild and domestic canids. Zoonotic in humans as dead-end hosts harboring L3 larvae (Despommier 2003).

**Route of infection.** Ingestion of embryonated eggs in food, water, fomites contaminated with canid feces (Despommier 2003).

**Site in human host.** Disseminated infection; common sites of infection are liver, eye, CNS, and lungs (Despommier 2003).

***Toxocaracati* Schrank, 1788**

*Toxocaramystax* (Zeder, 1800)

**Geographic distribution.** Worldwide (Despommier 2003).

**Natural hosts.** Wild and domestic felids. Zoonotic in humans as dead-end hosts harboring L3 larvae (Despommier 2003).

**Route of infection.** Ingestion of embryonated eggs in food, water, fomites contaminated with felid feces (Despommier 2003).

**Site in human host.** Disseminated infection; common sites of infection are liver, eye, CNS, and lungs (Despommier 2003).

●●●●●●●● **Oxyurida Weinland, 1858**

●●●●●●●●● **Oxyuridae Cobbold, 1864**

**Genus *Enterobius* Baird, 1853**

***Enterobiusvermicularis* (Linnaeus, 1758)**

*Enterobiusgregorii* Hugot, 1983

**Geographic distribution.** Worldwide (Wendt et al. 2019).

**Natural hosts.** Humans (Wendt et al. 2019).

**Route of infection.** Ingestion of embryonated eggs on contaminated fomites (Wendt et al. 2019).

**Site in human host.** Cecum, large intestine, appendix; ectopic colonization of female urogenital tract (Wendt et al. 2019).

**Genus *Syphacia* Seurat, 1916**

***Syphaciaoblevata* (Rudolphi, 1802)**

**Geographic distribution.** Worldwide (Riley 1919).

**Natural hosts.** Rodents (Riley 1919).

**Route of infection.** Presumed ingestion of embryonated eggs on contaminated fomites (Riley 1919).

**Site in human host.** Large intestine (Riley 1919).

●●●●●●●● **Spirudida De Ley & Blaxter, 2002**

●●●●●●●●● **Filarioidea Chabaoud & Anderson, 1959**

●●●●●●●●●● **Onchocercidae Chabaud & Anderson, 1959**

**Genus *Acanthocheilonema* Cobbold, 1870**

***Acanthocheilonemareconditum* (Grassi, 1889)**

**Geographic distribution.** Worldwide (Otranto and Eberhard 2011).

**Natural hosts.** Vector intermediate hosts are fleas and lice, primarily the cat flea (*Ctenocephalidesfelis*). Definitive hosts are canids. Zoonotic in humans (Otranto and Eberhard 2011).

**Route of infection.** Introduction of L3 larvae via the bite of an infected flea (Otranto and Eberhard 2011).

**Site in human host.** Eyes (Huynh et al. 2001; Otranto and Eberhard 2011).

**Notes.** Several cases of *Acanthocheilonema*-like nematodes recovered from human eyes have been reported, though not identified further (Otranto and Eberhard 2011).

**Genus *Breinlia* Yorke & Maplestone, 1926**

***Breinliaannulipapillata* (Johnston & Mawson, 1938)**

**Geographic distribution.** Australia (Koehler et al. 2021).

**Natural hosts.** Vector intermediate hosts are mosquitoes, although the full range of competent genera is not known. Definitive hosts are kangaroos and wallabies. Zoonotic in humans (Koehler et al. 2021).

**Route of infection.** Introduction of L3 larvae via the bite of an infected mosquito (Koehler et al. 2021).

**Site in human host.** Eye (Koehler et al. 2021).

**Genus *Brugia* Buckley, 1960**

***Brugiamalayi* (Brug, 1927)**

**Geographic distribution.** Southeast Asia, including Philippines, Malaysia, Indonesia, South Korea, Vietnam, India (Mak 1987; Dietrich et al. 2019).

**Natural hosts.** Vector intermediate hosts are mosquitoes, primarily in the genera *Aedes* and *Mansonia*. Definitive hosts are humans (Mak 1987; Dietrich et al. 2019).

**Route of infection.** Introduction of L3 larvae via the bite of an infected mosquito (Mak 1987; Dietrich et al. 2019).

**Site in human host.** Lymphatic system (Mak 1987; Dietrich et al. 2019).

***Brugiatimori* Partono et al., 1977**

**Geographic distribution.** Lesser Sunda Archipelago, including the islands of Timor, Sumba, Lembata, Pentar, Alor (Fischer et al. 2004).

**Natural hosts.** Vector intermediate hosts are mosquitoes in the genus *Anopheles*. Definitive hosts are humans (Fischer et al. 2004; Dietrich et al. 2019).

**Route of infection.** Introduction of L3 larvae via the bite of an infected mosquito (Fischer et al. 2004; Dietrich et al. 2019).

**Site in human host.** Lymphatic system (Fischer et al. 2004; Dietrich et al. 2019).

**Unassigned *Brugia* species**:

**American *Brugia* spp.**

**Geographic distribution.** North, Central, and South America (Orihel and Eberhard 1998).

**Natural hosts.** Unknown. Vector intermediate hosts are presumed to be mosquitoes. Possible definitive hosts include raccoons, rabbits, and felids. Presumed zoonotic in humans (Orihel and Eberhard 1998).

**Route of infection.** Introduction of L3 larvae via the bite of an infected mosquito (Orihel and Eberhard 1998).

**Site in human host.** Lymphatic system (Orihel and Eberhard 1998).

**Notes.** Several cases of zoonotic *Brugia* spp. have reported in the Americas. The precise identification of the causal agents is not known due to limited information on the morphologic features observed on histopath. Human infection in North America has been tentatively attributed to *B.beaveri* Ash & Little, 1964 or *B.lepori* Eberhard, 1984 (Orihel and Ash 1995; Orihel and Eberhard 1998).

**Genus *Dirofilaria* Railliet & Henry, 1911**

**SubgenusDirofilaria Railliet & Henry, 1911**

**Dirofilaria (D.) immitis (Leidy, 1856)**

*Filariamagalhaesi* Blanchard, 1896

*Dirofilarianasuae* Mazza, 1926

*Dirofilariapongoi* Vogel & Vogelsang, 1930

*Dirofilarialouisianensis* Faust et al., 1941

**Geographic distribution.** Worldwide (Orihel and Eberhard 1998).

**Natural hosts.** Vector intermediate hosts are mosquitoes. Definitive hosts are primarily wild and domestic canids. Zoonotic in humans (Orihel and Eberhard 1998).

**Route of infection.** Introduction of L3 larvae via the bite of an infected mosquito (Orihel and Eberhard 1998).

**Site in human host.** Heart, pulmonary vessels (Orihel and Eberhard 1998).

***Dirofilaria* (*D.*) *spectans* Freitas & Lint, 1949**

**Geographic distribution.** South America (Orihel and Eberhard 1998).

**Natural hosts.** Vector intermediate hosts are presumed to be mosquitoes. Definitive hosts is the giant river otter (*Pteronurabrasiliensis*). Zoonotic in humans (Orihel and Eberhard 1998).

**Route of infection.** Introduction of L3 larvae via the bite of an infected mosquito (Orihel and Eberhard 1998).

**Site in human host.** Digital artery (Orihel and Eberhard 1998).

**Notes.** Few features can separate *D.spectans* from *D.immitis*, thus the validity of the species and its involvement in human infections remain in question.

**Subgenus Nochtiella Faust, 1937**

***Dirofilaria* (*N.*) *repens* Railliet & Henry, 1911**

*Filariaconfunctivae* Addario, 1885, in part

**Geographic distribution.** Europe, Africa, Asia (Orihel and Eberhard 1998).

**Natural hosts.** Vector intermediate hosts are mosquitoes. Definitive hosts are carnivores, primarily wild and domestic canids, also felids. Zoonotic in humans (Orihel and Eberhard 1998).

**Route of infection.** Introduction of L3 larvae via the bite of an infected mosquito (Orihel and Eberhard 1998).

**Site in human host.** Skin; ectopic migration to the eye (Orihel and Eberhard 1998).

**Dirofilaria (N.) striata (Molin, 1858)**

**Geographic distribution.** North, Central, and South America (Orihel and Eberhard 1998).

**Natural hosts.** Vector intermediate hosts are mosquitoes. Definitive hosts are wild felids. Zoonotic in humans (Orihel and Eberhard 1998).

**Route of infection.** Introduction of L3 larvae via the bite of an infected mosquito (Orihel and Eberhard 1998).

**Site in human host.** Eye (Orihel and Eberhard 1998).

***Dirofilaria* (*N.*) *subdermata* (Monnig, 1924)**

**Geographic distribution.** North America (Orihel and Eberhard 1998).

**Natural hosts.** Vector intermediate hosts are mosquitoes. Definitive hosts are porcupines. Zoonotic in humans (Orihel and Eberhard 1998).

**Route of infection.** Introduction of L3 larvae via the bite of an infected mosquito (Orihel and Eberhard 1998).

**Site in human host.** Skin (Orihel and Eberhard 1998).

**Notes.** Features available in transverse histologic sections are unable to reliably distinguish *D.subdermata* from *D.ursi*; therefore, specimens recovered from human cases are generally reported as “*D.ursi*-like”.

**Dirofilaria (N.) tenuis Chandler, 1942**

*Filariaconfunctivae* Addario, 1885, in part

**Geographic distribution.** North America (Orihel and Eberhard 1998).

**Natural hosts.** Vector intermediate hosts are mosquitoes. Definitive hosts are primarily raccoons. Zoonotic in humans (Orihel and Eberhard 1998).

**Route of infection.** Introduction of L3 larvae via the bite of an infected mosquito (Orihel and Eberhard 1998).

**Site in human host.** Skin; ectopic migration to the eyes (Orihel and Eberhard 1998).

**Dirofilaria (N.) ursi Yamaguti, 1941**

**Geographic distribution.** North America, Russia, Japan (Orihel and Eberhard 1998).

**Natural hosts.** Vector intermediate hosts are black flies in the genus *Simulium*. Definitive hosts are bears. Zoonotic in humans (Orihel and Eberhard 1998).

**Route of infection.** Introduction of L3 larvae via the bite of an infected black fly (Orihel and Eberhard 1998).

**Site in human host.** Skin (Orihel and Eberhard 1998).

**Notes.** Features available in transverse histologic sections are unable to reliably distinguish *D.subdermata* from *D.ursi*; therefore, specimens recovered from human cases are generally reported as “*D.ursi*-like”.

***Dirofilaria* , incertae sedis**:

***Dirofilaria* sp. Genotype Hong Kong**

*Dirofilariahongkongensis* To et al., 2012

**Geographic distribution.** Southeast Asia (Dantas-Torres and Otranto 2020; To et al. 2012).

**Natural hosts.** Vector intermediate hosts are unknown. Definitive hosts are canids. Zoonotic in humans (To et al. 2012; Dantas-Torres and Otranto 2020).

**Route of infection.** Presumably via the introduction of L3 larvae via the bite of an infected vector.

**Site in human host.** Skin (To et al. 2012; Dantas-Torres and Otranto 2020).

**Notes.***Dirofilariahonkongensis* was described from dogs and humans in Hong Kong based on molecular characterization (To et al. 2012). The species was not described under the criteria of the ICZN and no type specimen was designated. To complicate matters, they described the species as ‘*Candidatus*Dirofilariahonkongensis’. The term candidatus is not used in zoological nomenclature and is usually only used for bacteria and other organisms that cannot be cultured. The species should be considered nomen nudum (Mathison and Pritt 2019; Dantas-Torres and Otranto 2020).

**Genus *Dunnifilaria* Mullin & Balastingam, 1973**

**Geographic distribution.** Mexico, Southeast Asia (Lam et al. 2010).

**Natural hosts.** Vector intermediate hosts are unknown. Definitive hosts are rodents. Zoonotic in humans (Lam et al. 2010).

**Route of Infection.** Presumed introduction of L3 larvae via the bite of an infected vector.

**Site in human host.** Eye (Lam et al. 2010).

**Notes.** The single human case was reported from the eye of a patient in Malaysia (Lam et al. 2010). The worm was not identified at the species level but was reported as being most consistent with *D.ramachandrani* Mullin & Balasingam, 1973, a parasite of the long-tailed giant rat in Malaysia.

**Genus *Loa* Stiles, 1905**

***Loaloa* (Cobbold, 1864)**

**Geographic distribution.** West-central Africa (Whittaker et al. 2018).

**Natural hosts.** Vector intermediate hosts are deer flies in the genus *Chrysops*. Definitive hosts are humans (Whittaker et al. 2018).

**Route of infection.** Introduction of L3 larvae via the bite of an infected deer fly (Whittaker et al. 2018).

**Site in human host.** Skin; ectopic migration to the eyes (Whittaker et al. 2018).

**Notes.** Cases of *L.loa* in non-travelers outside of endemic areas should be considered as possible misidentifications. There are many case reports of *L.loa* from India, many of which appear to represent misidentifications of *Dirofilaria* (prob. *D.repens*).

**Genus *Mansonella* Faust, 1929**

***Mansonellaozzardi* Manson, 1897**

**Geographic distribution.** Central and South America, Caribbean (Lima et al. 2016).

**Natural hosts.** Vector intermediate hosts are biting midges (genus *Culicoides*) and black flies (genus *Simulium*). Definitive hosts are humans (Lima et al. 2016).

**Route of infection.** Introduction of L3 larvae via the bite of an infected arthropod vector (Lima et al. 2016).

**Site in human host.** Skin (Lima et al. 2016).

***Mansonellaperstans* (Manson, 1891)**

*Dipetalonemaberghei* Chardome & Peel, 1951

**Geographic distribution.** Africa, South America, Caribbean (Mediannikov and Ranque 2018).

**Natural hosts.** Vector intermediate hosts are biting midges in the genus *Culicoides*. Definitive hosts are humans (Mediannikov and Ranque 2018).

**Route of infection.** Introduction of L3 larvae via the bite of an infected *Culicoides* biting midge (Mediannikov and Ranque 2018).

**Site in human host.** Peritoneal cavity, pleural cavity, pericardium, mesenteries (Mediannikov and Ranque 2018).

***Mansonellastreptocerca* (MacFie & Corson, 1922)**

**Geographic distribution.** Sub-Saharan Africa (Mediannikov and Ranque 2018).

**Natural hosts.** Vector intermediate hosts are biting midges in the genus *Culicoides*. Definitive hosts are humans, non-human primates (Mediannikov and Ranque 2018).

**Route of infection.** Introduction of L3 larvae via the bite of an infected *Culicoides* biting midge (Mediannikov and Ranque 2018).

**Site in human host.** Skin (Mediannikov and Ranque 2018).

**Genus *Meningonema* Orihel & Esslinger, 1973**

***Meningonemaperuzzii* Orihel & Esslinger, 1973**

**Geographic distribution.** Africa (Orihel and Eberhard 1998).

**Natural hosts.** Vector intermediate hosts are unknown. Definitive hosts are monkeys. Zoonotic in humans (Orihel and Eberhard 1998).

**Route of infection.** Introduction of L3 larvae via the bite of an infected arthropod vector (Orihel and Eberhard 1998).

**Site in human host.** CNS (Orihel and Eberhard 1998).

**Genus *Molinema* Freitas & Lint, 1939**

**Geographic distribution.** North, Central, and South America (Orihel and Eberhard 1998).

**Natural hosts.** Vector intermediate hosts are mosquitoes. Definitive hosts are rodents. Zoonotic in humans (Orihel and Eberhard 1998).

**Route of infection.** Introduction of L3 larvae via the bite of an infected mosquito (Orihel and Eberhard 1998).

**Site in human host.** Eyes (Orihel and Eberhard 1998).

**Notes.** Human cases of *Molinema* are rare. Cases from the northwestern United States are believed to be attributed to either *M.arbuta* (Highby, 1943), a parasite of porcupines, or *M.sprenti* (Anderson, 1953), a parasites of beaver (Orihel and Eberhard 1998).

**Genus *Onchocerca* Diesing, 1841**

***Onchocercacervicalis* Railliet & Henry, 1910**

**Geographic distribution.** Worldwide (Cambra-Pellejà et al. 2020; Papini et al. 2020).

**Natural hosts.** Vector intermediate hosts are biting midges in the genus *Culicoides*. Definitive hosts are horses and other equids. Zoonotic in humans (Cambra-Pellejà et al. 2020; Papini et al. 2020).

**Route of infection.** Introduction of L3 larvae via the bite of an infected black fly (Orihel and Eberhard 1998).

**Site in human host.** Skin, eyes (Burr et al. 1998; Lai et al. 2014).

***Onchocercadewitteijaponica* Uni et al., 2001**

**Geographic distribution.** Japan (Uni et al. 2015, 2017).

**Natural hosts.** Vector intermediate hosts are black flies in the genus *Simulium*. Definitive hosts are wild pigs. Zoonotic in humans (Uni et al. 2015, 2017).

**Route of infection.** Introduction of L3 larvae via the bite of an infected black fly (Uni et al. 2015, 2017).

**Site in human host.** Skin (Uni et al. 2015, 2017).

***Onchocercagutturosa* (Neumann, 1919)**

**Geographic distribution.** Africa, Europe, Asia, Australia (Cambra-Pellejà et al. 2020).

**Natural hosts.** Vector intermediate hosts are biting midges in the genus *Culicoides*. Definitive hosts are cattle. Zoonotic in humans (Cambra-Pellejà et al. 2020).

**Route of infection.** Introduction of L3 larvae via the bite of an infected black fly (Cambra-Pellejà et al. 2020).

**Site in human host.** Skin (Lai et al. 2014).

***Onchocercajakutensis* (Gubanov, 1964)**

**Geographic distribution.** Europe, Asia (Cambra-Pellejà et al. 2020).

**Natural hosts.** Vector intermediate hosts are black flies in the genus *Simulium*. Definitive hosts are deer. Zoonotic in humans (Cambra-Pellejà et al. 2020).

**Route of infection.** Introduction of L3 larvae via the bite of an infected black fly (Cambra-Pellejà et al. 2020).

**Site in human host.** Skin, eyes (Koehsler et al. 2007).

***Onchocercalupi* Rodonaja, 1967**

**Geographic distribution.** Europe, Asia, North America (Cantey et al. 2016).

**Natural hosts.** Putative vector intermediate hosts are black flies in the genus *Simulium*. Definitive hosts are wild and domestic canids and felids. Zoonotic in humans (Cantey et al. 2016).

**Route of infection.** Introduction of L3 larvae via the bite of an infected black fly (Cantey et al. 2016).

**Site in human host.** Skin, skeletal muscle, cervical nodules (Cantey et al. 2016).

***Onchocercavolvulus* (Leuckart, 1893)**

**Geographic distribution.** Sub-Saharan Africa, Yemen, Central and South America (Udall 2007).

**Natural hosts.** Vector intermediate hosts are black flies in the genus *Simulium*. Definitive hosts are humans (Udall 2007).

**Route of infection.** Introduction of L3 larvae via the bite of an infected black fly (Udall 2007).

**Site in human host.** Skin, subcutaneous nodules (Udall 2007).

**Genus *Pelecitus* Railliet & Henry, 1910**

**Geographic distribution.** Worldwide (Bain et al. 2011).

**Natural hosts.** Vector intermediate hosts are various avian live, mosquitoes, and tabanid flies. Definitive hosts are birds, lagomorphs, and marsupials. Zoonotic in humans (Bain et al. 2011).

**Route of infection.** Introduction of L3 larva via the bite of an infected mosquito (Bain et al. 2011).

**Site in human host.** Eyes (Bain et al. 2011).

**Notes.** The single case of human infection with *Pelecitus* was from Brazil and was not identified to the species level (Bain et al. 2011).

**Genus *Wuchereria* Da Silva Araujo, 1877**

***Wuchereriabancrofti* (Cobbold, 1877)**

*Wuchereriapacifica* Manson-Bahr, 1941

**Geographic distribution.** Circumtropical, including South America, Caribbean, Africa, Asia, and Pacific Islands (Mak 1987; Dietrich et al. 2019).

**Natural hosts.** Vector intermediate hosts are mosquitoes, including the genera *Aedes*, *Anopheles*, and *Culex*. Definitive hosts are humans (Mak 1987; Dietrich et al. 2019).

**Route of infection.** Introduction of L3 larvae via the bite of an infected mosquito (Mak 1987; Dietrich et al. 2019).

**Site in human host.** Lymphatic system (Mak 1987; Dietrich et al. 2019).

●●●●●●●●●● **Setariidae Skrjabin & Shikhobalova, 1945**

**Genus *Setaria* Viborg, 1795**

***Setariaequina* (Abildgaard, 1789)**

**Geographic distribution.** Worldwide (Nabie et al. 2017).

**Natural hosts.** Vector intermediate hosts are believed to be mosquitoes in the genera *Aedes* or *Culex*. Definitive hosts are horses and other equids. Zoonotic in humans (Nabie et al. 2017).

**Route of infection.** Introduction of L3 larvae via the bite of an infected mosquito (Nabie et al. 2017).

**Site in human host.** Eyes (Nabie et al. 2017).

***Setarialabiatopapillosa* (Allessandrini, 1838)**

**Geographic distribution.** Worldwide (Panaitescu et al. 1999).

**Natural hosts.** Vector intermediate hosts are believed to be mosquitoes in the genus *Aedes*. Definitive hosts are bovids. Zoonotic in humans (Panaitescu et al. 1999).

**Route of infection.** Introduction of L3 larvae via the bite of an infected mosquito (Panaitescu et al. 1999).

**Site in human host.** Eyes (Panaitescu et al. 1999).

**Filaroidea, incertae sedis**:

“**Microfilaria” *bolivarensis* (Godoy et al., 1980)**

**Geographic distribution.** Venezuela (Orihel and Eberhard 1998).

**Natural hosts.** Unknown; presumed to be zoonotic in humans (Orihel and Eberhard 1998).

**Route of infection.** Presumed introduction of L3 larvae via the bite of an infected arthropod vector.

**Site in human host.** Unknown, only described from microfilariae in blood (Orihel and Eberhard 1998).

“**Microfilaria” *rodhaini* (Peel & Chardome, 1946)**

**Geographic distribution.** Gabon (Orihel and Eberhard 1998).

**Natural hosts.** Vector intermediate hosts unknown. Definitive hosts presumed to be bonobos and chimpanzees. Zoonotic in humans (Orihel and Eberhard 1998).

**Route of infection.** Presumed introduction of L3 larvae via the bite of an infected arthropod vector

**Site in human host.** Unknown, only described from microfilariae in blood (Orihel and Eberhard 1998).

“**Microfilaria” *semiclarum* (Fain, 1974)**

**Geographic distribution.** Democratic Republic of Congo (Orihel and Eberhard 1998).

**Natural hosts.** Unknown; presumed to be zoonotic in humans (Orihel and Eberhard 1998).

**Route of infection.** Presumed introduction of L3 larvae via the bite of an infected arthropod vector.

**Site in human host.** Unknown, only described from microfilariae in blood (Orihel and Eberhard 1998).

●●●●●●●●●● **Spiruroidea Oerley, 1885**

●●●●●●●●●●● **Gongylonematidae Sobolev, 1949**

**Genus *Gongylonema* Molin, 1857**

***Gongylonemapulchrum* Molin, 1857**

**Geographic distribution.** Worldwide (Libertin et al. 2017).

**Natural hosts.** Intermediate hosts are insects, primarily coprophagous beetles and cockroaches. Definitive hosts are mammals, including ruminants, carnivores, insectivores, lagomorphs. Zoonotic in humans (Libertin et al. 2017).

**Route of infection.** Ingestion of L3 larvae in infected insects (Libertin et al. 2017).

**Site in human host.** Oral mucosa (Libertin et al. 2017).

●●●●●●●●●●● **Gnathostomatidae Railliet, 1895**

**Genus *Gnathostoma* Owen, 1837**

***Gnathostomabinucleatum* (Almeyda-Artigas, 1991)**

**Geographic distribution.** Central and South America (Cornaglia et al. 2016).

**Natural hosts.** First intermediate hosts are freshwater copepods. Second intermediate hosts are freshwater fish, amphibians, birds, rodents, and reptiles can serve as paratenic hosts. Definitive hosts are wild and domestic felids and canids. Zoonotic in humans as dead-end hosts harboring L3 larvae (Cornaglia et al. 2016).

**Route of infection.** Ingestion of L3 larvae in infected intermediate or paratenic hosts (Cornaglia et al. 2016).

**Site in human host.** Skin, heart, intestinal tract, ears, eyes, CNS (Cornaglia et al. 2016).

***Gnathostomadoloresi* (Tubangui, 1925)**

**Geographic distribution.** Southeast Asia, Japan (Herman and Chiodini 2009).

**Natural hosts.** First intermediate hosts are freshwater copepods. Second intermediate hosts are freshwater fish. Amphibians, birds, rodents, and reptiles can serve as paratenic hosts. Definitive hosts are wild and domestic pigs. Zoonotic in humans as dead-end hosts harboring L3 larvae (Herman and Chiodini 2009).

**Route of infection.** Ingestion of L3 larvae in infected intermediate or paratenic hosts (Herman and Chiodini 2009).

**Site in human host.** Skin, heart, intestinal tract, ears, eyes, CNS (Herman and Chiodini 2009).

***Gnathostomahispidum* (Fedtschenko, 1872)**

**Geographic distribution.** Southeast Asia, India, Australia (Herman and Chiodini 2009).

**Natural hosts.** First intermediate hosts are freshwater copepods. Second intermediate hosts are freshwater fish. Amphibians, birds, rodents, and reptiles can serve as paratenic hosts. Definitive hosts are wild and domestic pigs. Zoonotic in humans as dead-end hosts harboring L3 larvae (Herman and Chiodini 2009).

**Route of infection.** Ingestion of L3 larvae in infected intermediate or paratenic hosts (Herman and Chiodini 2009).

**Site in human host.** Skin, heart, intestinal tract, ears, eyes, CNS (Herman and Chiodini 2009).

***Gnathostomamalaysiae* (Miyazaki & Dunn, 1965)**

**Geographic distribution.** Southeast Asia (Nomura et al. 2000).

**Natural hosts.** First intermediate hosts are freshwater copepods. Second intermediate hosts are freshwater fish. Amphibians, birds, rodents, and reptiles can serve as paratenic hosts. Definitive hosts are rodents, including rats. Zoonotic in humans as dead-end hosts harboring L3 larvae (Nomura et al. 2000).

**Route of infection.** Ingestion of L3 larvae in infected intermediate or paratenic hosts (Nomura et al. 2000).

**Site in human host.** Skin (Nomura et al. 2000).

**Notes.** The single case report of *G.malaysiae* in humans was reported from two Japanese fisherman who described eating raw freshwater shrimp in Myanmar. The species-level identification was considered presumptive (Nomura et al. 2000).

***Gnathostomanipponicum* Yamaguti, 1941**

**Geographic distribution.** Japan, Korea, China (Herman and Chiodini 2009).

**Natural hosts.** First intermediate hosts are freshwater copepods. Second intermediate hosts are freshwater fish. Amphibians, birds, rodents, and reptiles can serve as paratenic hosts. Definitive hosts are weasels and minks. Zoonotic in humans as dead-end hosts harboring L3 larvae (Herman and Chiodini 2009).

**Route of infection.** Ingestion of L3 larvae in infected intermediate or paratenic hosts (Herman and Chiodini 2009).

**Site in human host.** Skin, heart, intestinal tract, ears, eyes, CNS (Herman and Chiodini 2009).

***Gnathostomaspinigerum* Levinsen, 1889**

**Geographic distribution.** Southeast Asia, southern Africa, Madagascar, Australia (Herman and Chiodini 2009).

**Natural hosts.** First intermediate hosts are freshwater copepods. Second intermediate hosts are freshwater fish. Amphibians, birds, rodents, and reptiles can serve as paratenic hosts. Definitive hosts are wild and domestic canids and felids. Zoonotic in humans as dead-end hosts harboring L3 larvae (Herman and Chiodini 2009).

**Route of infection.** Ingestion of L3 larvae in infected intermediate or paratenic hosts (Herman and Chiodini 2009).

**Site in human host.** Skin, heart, intestinal tract, ears, eyes, CNS (Herman and Chiodini 2009).

●●●●●●●●●● **Camallanida Travassos, 1920**

●●●●●●●●●●● **Dracunculidae Stiles, 1907**

**Genus *Dracunculus* Reichard, 1759**

***Dracunculusmedinensis* (Linnaeus, 1758)**

**Geographic distribution.** Focal areas in sub-Saharan Africa, including Chad, Angola, and South Sudan (Eberhard et al. 2014b; Hopkins et al. 2020).

**Natural hosts.** Intermediate hosts are freshwater copepods; freshwater fish and amphibians can serve as paratenic or transport hosts. Definitive hosts are humans; canids, felids, and non-human primates can serve as reservoir hosts (Eberhard et al. 2014b; Hopkins et al. 2020).

**Route of infection.** Ingestion of L3 larvae in infected copepods or paratenic hosts (Eberhard et al. 2014b; Hopkins et al. 2020).

**Site in human host.** Subcutaneous tissues (Eberhard et al. 2014b; Hopkins et al. 2020).

**Notes.** Reports of *D.medinensis* infections acquired outside of historically endemic zones and/or in zones where eradication has been well-established are highly likely to represent misidentifications with other nematodes. In limited instances, these represent genuine infections with other unknown *Dracunculus* spp. (Kobayashi et al. 1986).

***Dracunculus* sp.**

**Geographic distribution.** Vietnam (Thach et al. 2021).

**Natural hosts.** Unknown; presumed zoonotic in humans (Thach et al. 2021).

**Route of infection.** Unknown; presumed ingestion of intermediate or reservoir host.

**Site in human host.** Subcutaneous abscesses (Thach et al. 2021).

**Notes.** There is a single report of human infection with a non-*medinensisDracunculus* species from Vietnam. Genetically it is most similar to *D.insignis* (Leidy, 1858) and *D.lutrae* Crichton et Beverley-Burton, 1973, parasites of carnivores (Thach et al. 2021).

**Spiruroidea, incertae sedis**:

**Spirurina Type X**

**Geographic distribution.** Japan (Goto et al. 1998; Makino et al. 2014).

**Natural hosts.** Intermediate or paratenic hosts are marine squid and fish. Definitive hosts unknown. Zoonotic in humans (Goto et al. 1998; Makino et al. 2014).

**Route of infection.** Presumed ingestion of L3 larvae in undercooked squid or fish (Goto et al. 1998; Makino et al. 2014).

**Site in human host.** Skin, eyes, ileum (Goto et al. 1998; Makino et al. 2014).

**Notes.** Around 50 infections with Spirurina type X larvae have been identified in humans who have consumed various seafood items, particularly small squid species. Molecular studies tentatively identified similar type X larvae as those of *Crassicaudagiliakiana* Skrjaban & Andreeva, 1934, a nematode of Baird’s beaked whale (*Berardiusbairdii*) (Sugiyama et al. 2007).

●●●●●●●●●● **Thelazioidea Skrjabin, 1915**

●●●●●●●●●●● **Thelaziidae Skrjabin, 1915**

**Genus *Oxyspirura* Dräsche in Stossich, 1897**

**Geographic distribution.** Worldwide (Dung et al. 2020).

**Natural hosts.** Intermediate hosts are arthropods. Definitive hosts are birds and non-human primates. Zoonotic in humans as dead-end hosts harboring L3 larvae (Dung et al. 2020).

**Route of Infection.** Ingestion of infected arthropod intermediate host (Dung et al. 2020).

**Site in human host.** Skin (Dung et al. 2020).

**Notes.***Oxyspirura* has been reported as a cause of pruritic cutaneous larval migrans in a patient in Vietnam, but the specimen was not characterized at the species level (Dung et al. 2020).

**Genus *Rictularia* Froelich, 1802**

**Geographic distribution.** Worldwide (Kenney et al. 1975).

**Natural hosts.** Arthropods are intermediate hosts. A wide range of mammals serve as definitive hosts. Zoonotic in humans (Kenney et al. 1975).

**Route of infection.** Presumed ingestion of L3 larvae in infected intermediate hosts.

**Site in human host.** Appendix (Kenney et al. 1975).

**Notes.** The single human case of infection with *Rictularia* was reported from a patient in New York postmortem (Kenney et al. 1975).

**Genus *Thelazia* Bosc in Blainville 1819**

***Thelaziacaliforniensis* Price, 1930**

**Geographic distribution.** Western North America (Bradbury et al. 2018).

**Natural hosts.** Vector intermediate hosts are flies in the genus *Fannia*. Definitive hosts are mammals, including wild and domestic carnivores, deer, sheep, and lagomorphs. Zoonotic in humans (Bradbury et al. 2018).

**Route of infection.** Deposition of L3 larvae on the eye by vector fly (Bradbury et al. 2018).

**Site in human host.** Eye (Bradbury et al. 2018).

***Thelaziacallipaeda* Railliet & Henry, 1910**

**Geographic distribution.** Europe, Asia (Otranto and Dutto 2008; Sah et al. 2018).

**Natural hosts.** Vector intermediate hosts are flies in the families Drosophilidae, primarily the genus *Phortica*. Definitive hosts are mammals, primarily carnivores and lagomorphs. Zoonotic in humans (Otranto and Dutto 2008; Sah et al. 2018).

**Route of infection.** Deposition of L3 larvae on the eye by vector fly (Otranto and Dutto 2008; Sah et al. 2018).

**Site in human host.** Eye (Otranto and Dutto 2008; Sah et al. 2018).

***Thelaziagulosa* (Railliet & Henry, 1910)**

**Geographic distribution.** North America, Europe, Central Asia, Australia (Bradbury et al. 2018; Bradbury et al. 2019a).

**Natural hosts.** Vector intermediate hosts are muscid flies in the genus *Musca*. Definitive hosts are ungulates, primarily cattle. Zoonotic in humans (Bradbury et al. 2018; Bradbury et al. 2019a).

**Route of infection.** Deposition of L3 larvae on the eye by vector fly (Bradbury et al. 2018; Bradbury et al. 2019a).

**Site in human host.** Eye (Bradbury et al. 2018; Bradbury et al. 2019a).

●●●●●●●●● **Physalopteroidea Railliet, 1893**

●●●●●●●●●● **Physalopteridae Railliet, 1893**

**Genus *Physaloptera* Rudolfi, 1819**

***Physalopteracaucasica* (von Linstow, 1902)**

**Geographic distribution.** Africa, Asia (Vandepitte et al. 1964; Makki et al. 2017).

**Natural hosts.** Intermediate hosts are insects, especially crickets, cockroaches, and beetles. Definitive hosts are non-human primates. Zoonotic in humans (Vandepitte et al. 1964; Makki et al. 2017).

**Route of infection.** Presumed ingestion of L3 larvae in infected insects (Vandepitte et al. 1964; Makki et al. 2017).

**Site in human host.** Gastrointestinal tract (Vandepitte et al. 1964; Makki et al. 2017).

**Notes.** This species is often placed in the genus *Abbreviata* Travassos, 1926. Eggs consistent with *Physaloptera* spp. have been recovered from human coprolites in archaeological sites across the world, but species identity has not been established in paleoparasitological cases (Makki et al. 2017).

●●●●●●● **Rhabditina Chitwood, 1933**

●●●●●●●● **Rhabditida Orley, 1880**

●●●●●●●●● **Rhabditidae Orley, 1880**

**Genus *Diploscapter* Cobb, 1913**

***Diploscaptercoronata* (Cobb, 1893)**

**Geographic distribution.** Worldwide (Morimoto et al. 2006; Chandler 2009).

**Natural hosts.** None (environmental); humans are incidental hosts (Morimoto et al. 2006; Chandler 2009).

**Route of infection.** Unknown, presumed ingestion of L3 larvae on contaminated produce (Morimoto et al. 2006; Chandler 2009).

**Site in human host.** Gastrointestinal tract (Morimoto et al. 2006; Chandler 2009).

**Genus *Halicephalobus* Timm, 1956**

***Halicephalobusgingivalis* (Stephanski, 1954)**

*Micronemadeletrix* Anderson & Bemrick, 1965

**Geographic distribution.** Worldwide (Onyiche et al. 2018).

**Natural hosts.** None (environmental); humans are incidental hosts (Onyiche et al. 2018).

**Route of infection.** Unknown, presumed inoculation of L3 larvae into wounds or mucus membranes; organ transplantation (Onyiche et al. 2018).

**Site in human host.** Disseminated infection with predilection for CNS (Papadi et al. 2013; Onyiche et al. 2018).

**Genus *Pelodera* Schneider, 1866**

***Peloderastrongyloides* (Schneider, 1860)**

**Geographic distribution.** Worldwide (Saari and Nikander 2006).

**Natural hosts.** None (environmental); humans are incidental hosts (Saari and Nikander 2006).

**Route of infection.** Unknown, presumed direct penetration of skin by L3 larvae or possibly inoculation into wounds (Saari and Nikander 2006).

**Site in human host.** Skin (Orihel and Ash 1995).

**Genus *Rhabditis* Dujardin, 1845**

**Geographic distribution.** Worldwide (Fadaei Tehrani et al. 2019).

**Natural hosts.** None (environmental); humans are incidental hosts (Fadaei Tehrani et al. 2019).

**Route of infection.** Unknown, presumed inoculation of L3 larvae into wounds or mucus membranes (Fadaei Tehrani et al. 2019).

**Site in human host.** Small intestine, urogenital tract, ear (Teschner et al. 2014; Fadaei Tehrani et al. 2019).

**Notes.** Human cases of *Rhabditis* are not usually characterized at the species level. There have been cases specifically attributed to *R.axei* (Cobbold, 1884) from China (Yu et al. 2019), Iran (Meamar et al. 2007), and Zimbabwe (Goldsmid 1967) and *R.hominis* Kobayashi, 1920 from Japan and the United States (Sandground 1925). It is not always clear which cases represent true infection versus environmental contamination of clinical specimens (Sandground 1925).

●●●●●●●●● **Strongyloididae Chitwood & McIntosh, 1934**

**Genus *Strongyloides* Grassi, 1879**

***Strongyloidesfuellbornifuellborni* von Linstow, 1905**

**Geographic distribution.** Africa, Southeast Asia (Viney et al. 1991; Barratt et al. 2019).

**Natural hosts.** Non-human primates. Zoonotic in humans (Viney et al. 1991; Barratt et al. 2019).

**Route of infection.** Penetration of the skin by L3 larvae (Viney et al. 1991; Barratt et al. 2019).

**Site in human host.** Small intestine, disseminated infection in immunocompromised hosts, including to the lung, skin, kidneys, and brain (Viney et al. 1991; Barratt et al. 2019).

***Strongyloidesfuellbornikellyi* Viney et al., 1991**

**Geographic distribution.** Papua New Guinea (Viney et al. 1991; Barratt et al. 2019).

**Natural hosts.** Non-human primates. Zoonotic in humans (Viney et al. 1991; Barratt et al. 2019).

**Route of infection.** Penetration of the skin by L3 larvae (Viney et al. 1991; Barratt et al. 2019).

**Site in human host.** Small intestine, disseminated infection in immunocompromised hosts, including to the lung, skin, kidneys, and brain (Viney et al. 1991; Barratt et al. 2019).

***Strongyloidesmyopotami* Artigas & Pacheco, 1933**

**Geographic distribution.** South America, North America, Europe, Africa, Asia (Choe et al. 2014).

**Natural hosts.** Nutria. Zoonotic in humans as a dead-end host (Choe et al. 2014).

**Route of infection.** Penetration of skin by L3 larvae (Choe et al. 2014).

**Site in human host.** Skin (Choe et al. 2014).

**Notes.***Strongyloidesmyopotami* causes a short-lived dermatologic reaction in human skin, commonly referred to as ‘nutria itch’ or ‘marsh itch’. The parasite cannot survive in the human host and does not migrate beyond the dermis (Choe et al. 2014). Experimental evidence suggests the possibility of fleeting patent infection in humans (Little 1965).

***Strongyloidesprocyonis* Little, 1966**

**Geographic distribution.** North America, Asia (Choe et al. 2014).

**Natural hosts.** Raccoons. Zoonotic in humans as a dead-end host (Choe et al. 2014).

**Route of infection.** Penetration of skin by L3 larvae (Choe et al. 2014).

**Site in human host.** Skin (Choe et al. 2014).

**Notes.***Strongyloidesprocyonis* causes a short-lived dermatologic reaction in human skin, commonly referred to as ‘swimmer’s itch’ or ‘marsh itch’. The parasite cannot survive in the human host and does not migrate beyond the dermis (Choe et al. 2014).

***Strongyloidesstercoralis* Bavay, 1876**

**Geographic distribution.** Worldwide (Page et al. 2018; Barratt et al. 2019).

**Natural hosts.** Humans; dogs may serve as reservoir hosts (Page et al. 2018; Barratt et al. 2019).

**Route of infection.** Penetration of the skin by L3 larvae; organ transplantation (Abanyie et al. 2015; Page et al. 2018; Barratt et al. 2019).

**Site in human host.** Small intestine, disseminated infection in immunocompromised hosts, including to the lung, skin, kidneys, and brain (Page et al. 2018; Barratt et al. 2019).

●●●●●●●● **Strongylida Molin, 1861**

●●●●●●●●● **Chabertiidae Popova, 1952**

**Genus *Oesophagostomum* Molin, 1861**

***Oesophagostomumaculaetum* (von Linstow, 1879)**

**Geographic distribution.** Southeast Asia, Japan (Polderman and Blotkamp 1995).

**Natural hosts.** Non-human primates. Zoonotic in humans (Polderman and Blotkamp 1995).

**Route of infection.** Ingestion of L3 larvae on plants (Polderman and Blotkamp 1995).

**Site in human host.** Large intestine (Ghai et al. 2014; Polderman and Blotkamp 1995).

***Oesophagostomumbifurcum* (Creplin, 1849)**

**Geographic distribution.** Sub-Saharan Africa (Polderman and Blotkamp 1995).

**Natural hosts.** Non-human primates. Zoonotic in humans (Polderman and Blotkamp 1995).

**Route of infection.** Ingestion of L3 larvae on plants (Polderman and Blotkamp 1995).

**Site in human host.** Large intestine (Polderman and Blotkamp 1995; Ghai et al. 2014).

***Oesophagostomumstephanostomum* Stossich, 1904**

**Geographic distribution.** Sub-Saharan Africa, Brazil (Polderman and Blotkamp 1995).

**Natural hosts.** Non-human primates. Zoonotic in humans (Glen and Brooks 1985; Polderman and Blotkamp 1995).

**Route of infection.** Ingestion of L3 larvae on plants (Polderman and Blotkamp 1995).

**Site in human host.** Large intestine (Polderman and Blotkamp 1995; Ghai et al. 2014).

●●●●●●●●● **Strongylidae Baird, 1853**

**Genus *Ternidens* Railliet & Henry, 1909**

***Ternidensdeminutus* (Railliet & Henry, 1905)**

**Geographic distribution.** Sub-Saharan Africa (Bradbury 2019).

**Natural hosts.** Non-human primates. Zoonotic in humans (Bradbury 2019).

**Route of infection.** Unknown; presumed ingestion of L3 larvae on contaminated food or fomites, or ingestion of L3 in arthropod intermediate or paratenic hosts (Bradbury 2019).

**Site in human host.** Large intestine (Bradbury 2019).

●●●●●●●●● **Syngamidae Leiper, 1912**

**Genus *Mammomonogamus* Ryzhikovk, 1948**

***Mammomonogamuslaryngeus* Ryzhikovk, 1948**

**Geographic distribution.** Circumtropical (Lopes-Torres et al. 2020).

**Natural hosts.** Mammals, including ruminants and felids. Annelids, mollusks, and arthropods may serve as paratenic hosts. Zoonotic in humans (da Costa et al. 2005; Lopes-Torres et al. 2020).

**Route of infection.** Unknown, presumed to be from ingestion of embryonated eggs or larvae, or the consumption of a paratenic host (da Costa et al. 2005).

**Site in human host.** Bronchial tree (da Costa et al. 2005).

●●●●●●●●● **Ancylostomatidae Looss, 1905**

**Genus *Ancylostoma* Dubini, 1843**

***Ancylostomabraziliense* Gomes de Faria, 1910**

**Geographic distribution.** Southern United States, Central and South America, southern Africa, Southeast Asia (Tekely et al. 2013).

**Natural hosts.** Wild and domestic canids and felids. Zoonotic in humans as a dead-end host (Tekely et al. 2013).

**Route of infection.** Penetration of skin by L3 larvae (Tekely et al. 2013).

**Site in human host.** Skin (Tekely et al. 2013).

***Ancylostomacaninum* (Ercolani, 1859)**

**Geographic distribution.** Worldwide (Tekely et al. 2013; Shepherd et al. 2018).

**Natural hosts.** Wild and domestic canids and felids. Zoonotic in humans (Tekely et al. 2013; Shepherd et al. 2018).

**Route of infection.** Penetration of skin by L3 larvae (Tekely et al. 2013).

**Site in human host.** Skin, rarely small intestine (Tekely et al. 2013).

***Ancylostomaceylanicum* Looss, 1911**

**Geographic distribution.** Southeast Asia, Australia, Middle East (Traub 2013).

**Natural hosts.** Wild and domestic canids and felids. Zoonotic in humans (Traub 2013).

**Route of infection.** Penetration of skin by L3 larvae (Traub 2013).

**Site in human host.** Small intestine (Traub 2013).

***Ancylostomaduodenale* (Dubini, 1843)**

**Geographic distribution.** Worldwide in tropics and subtropics; host spots of endemicity for human infection are China, India, Egypt, northern Australia, Latin America (Brooker et al. 2004; Hotez et al. 2004).

**Natural hosts.** Mammals, including humans, dogs, cats (Brooker et al. 2004; Hotez et al. 2004).

**Route of infection.** Penetration of skin by L3 larvae (Brooker et al. 2004; Hotez et al. 2004).

**Site in human host.** Small intestine (Brooker et al. 2004; Hotez et al. 2004).

**Genus *Bunostomum* Railliet, 1902**

***Bunostomumphlebotomum* (Railliet, 1900)**

**Geographic distribution.** Worldwide (Tekely et al. 2013).

**Natural hosts.** Bovids. Zoonotic in humans as a dead-end host (Tekely et al. 2013).

**Route of infection.** Penetration of skin by L3 larvae (Tekely et al. 2013).

**Site in human host.** Skin (Tekely et al. 2013).

**Genus *Necator* Stiles, 1903**

***Necatoramericanus* (Stiles, 1902)**

**Geographic distribution.** Worldwide in tropics and subtropics; hot spots of endemicity for human infection are southern China, southern India, Southeast Asia, sub-Saharan Africa, Latin America, southeastern USA (Brooker et al. 2004; Hotez et al. 2004).

**Natural hosts.** Humans (Brooker et al. 2004; Hotez et al. 2004).

**Route of infection.** Penetration of skin by L3 larvae (Brooker et al. 2004; Hotez et al. 2004).

**Site in human host.** Small intestine (Brooker et al. 2004; Hotez et al. 2004).

***Necatorgorillae* Noda & Yamada, 1964**

**Geographic distribution.** Sub-Saharan Africa (Kalousová et al. 2016).

**Natural hosts.** Gorillas, chimpanzees. Zoonotic in humans (Kalousová et al. 2016).

**Route of infection.** Penetration of skin by L3 larvae (Kalousová et al. 2016).

**Site in human host.** Small intestine (Kalousová et al. 2016).

**Genus *Uncinaria* Frölich, 1789**

***Uncinariastenocephala* (Railliet, 1884)**

**Geographic distribution.** Temperate and subarctic regions of the Northern Hemisphere (Tekely et al. 2013).

**Natural hosts.** Carnivores, including wild and domestic canids and felids. Zoonotic in humans as a dead-end host (Tekely et al. 2013).

**Route of infection.** Penetration of skin by L3 larvae (Tekely et al. 2013).

**Site in human host.** Skin (Tekely et al. 2013).

●●●●●●●●● **Trichostrongylidae Leiper, 1912**

**Genus *Haemonchus* Cobb, 1989**

***Haemonchuscontortus* (Rudolphi, 1802)**

**Geographic distribution.** Worldwide (Ghadirian and Arfaa 1975).

**Natural hosts.** Many ruminants. Zoonotic in humans (Ghadirian and Arfaa 1975).

**Route of infection.** Ingestion of L3 larvae on contaminated plants and produce (Ghadirian and Arfaa 1975).

**Site in human host.** Small intestine (Ghadirian and Arfaa 1975).

**Genus *Marshallagia* Orloff, 1933**

***Marshallagiamarshalli* (Ransom, 1907)**

**Geographic distribution.** Worldwide (Ghadirian and Arfaa 1973).

**Natural hosts.** Many ruminants. Zoonotic in humans (Ghadirian and Arfaa 1973).

**Route of infection.** Ingestion of L3 larvae on contaminated plants and produce (Ghadirian and Arfaa 1973).

**Site in human host.** Small intestine (Ghadirian and Arfaa 1973).

**Genus *Nematodirus* Ransom, 1907**

***Nematodirusabnormalis* (May, 1920)**

**Geographic distribution.** Worldwide (Ghadirian and Arfaa 1973; Bradbury 2006).

**Natural hosts.** Primarily sheep. Zoonotic in humans (Ghadirian and Arfaa 1973; Bradbury 2006).

**Route of infection.** Ingestion of L3 larvae on contaminated plants and produce (Ghadirian and Arfaa 1973; Bradbury 2006).

**Site in human host.** Small intestine (Ghadirian and Arfaa 1973; Bradbury 2006).

**Genus *Ostertagia* Ransom, 1907**

***Ostertagiaostertagi* (Stiles, 1892)**

**Geographic distribution.** Worldwide (Ghadirian and Arfaa 1973; Anderson 1988).

**Natural hosts.** Primarily bovids; also sheep, goats, equids, and other ruminants. Zoonotic in humans (Ghadirian and Arfaa 1973; Anderson 1988).

**Route of infection.** Ingestion of L3 larvae on contaminated plants and produce (Ghadirian and Arfaa 1973).

**Site in human host.** Small intestine (Ghadirian and Arfaa 1973).

**Genus *Teladorsagia* Andreeva & Satubaldin, 1953**

***Teladorsagiacircumcincta* (Stadelman, 1894)**

**Geographic distribution.** Temperate climates (Ashrafi et al. 2020).

**Natural hosts.** Primarily sheep, also goats; zoonotic in humans (Ashrafi et al. 2020).

**Route of infection.** Ingestion of L3 larvae on plants and produce (Ashrafi et al. 2020).

**Site in human host.** Small intestine (Ashrafi et al. 2020).

**Genus *Trichostrongylus* Looss, 1905**

***Trichostrongylusaxei* (Cobbold, 1879)**

**Geographic distribution.** Worldwide (Sato et al. 2011).

**Natural hosts.** Ungulates, primarily cattle, sheep, goats, and horses. Zoonotic in humans (Sato et al. 2011).

**Route of infection.** Ingestion of L3 larvae on plants and produce (Cancrini et al. 1982; Sato et al. 2011).

**Site in human host.** Small intestine (Sato et al. 2011).

***Trichostrongyluscapricola* Ransom, 1907**

**Geographic distribution.** Worldwide (Ghadirian and Arfaa 1973; Cancrini et al. 1982).

**Natural hosts.** Ungulates, primarily cattle, sheep, and goats. Zoonotic in humans (Ghadirian and Arfaa 1973; Cancrini et al. 1982).

**Route of infection.** Ingestion of L3 larvae on plants and produce (Ghadirian and Arfaa 1973; Cancrini et al. 1982).

**Site in human host.** Small intestine (Ghadirian and Arfaa 1973; Cancrini et al. 1982).

***Trichostrongyluscolubriformis* (Giles, 1892)**

**Geographic distribution.** Worldwide; predominate in the Middle East (Sato et al. 2011).

**Natural hosts.** Ungulates, primarily cattle, sheep, and goats. Zoonotic in humans (Sato et al. 2011).

**Route of infection.** Ingestion of L3 larvae on plants and produce (Sato et al. 2011).

**Site in human host.** Small intestine (Sato et al. 2011).

***Trichostrongyluslongispicularis* Gordon, 1933**

*Trichostrongyluslerouxi* Biocca et al., 1974

**Geographic distribution.** Worldwide (excluding Africa) (Ghadirian 1977).

**Natural hosts.** Ungulates, primarily cattle, sheep, and goats. Zoonotic in humans (Ghadirian 1977).

**Route of infection.** Ingestion of L3 larvae on plants and produce (Ghadirian 1977).

**Site in human host.** Small intestine (Ghadirian 1977).

***Trichostrongylusorientalis* Jimbo, 1914**

**Geographic distribution.** Southeast Asia, Japan (Sato et al. 2011).

**Natural hosts.** Ungulates, including cattle, sheep, goats, and horses. Zoonotic in humans (Sato et al. 2011).

**Route of infection.** Ingestion of L3 larvae on plants and produce (Sato et al. 2011).

**Site in human host.** Small intestine (Sato et al. 2011).

***Trichostrongylusvitrinus* Looss, 1905**

**Geographic distribution.** Worldwide (Ghadirian and Arfaa 1975; Cancrini et al. 1982).

**Natural hosts.** Ungulates, including sheep, goats. Zoonotic in humans (Ghadirian and Arfaa 1975; Cancrini et al. 1982).

**Route of infection.** Ingestion of L3 larvae on plants and produce (Ghadirian and Arfaa 1975; Cancrini et al. 1982).

**Site in human host.** Small intestine (Ghadirian and Arfaa 1975; Cancrini et al. 1982).

●●●●●●●●● **Angiostrongylidae Boehm & Gebauer, 1934**

**Genus *Angiostrongylus* Kamensky, 1905**

***Angiostrongyluscantonensis* (Chen, 1935)**

**Geographic distribution.** Asia, South Pacific, Hawaii, Caribbean, Africa, southern United States, Central and South America (Cowie 2013).

**Natural hosts.** Intermediate hosts are terrestrial mollusks. Paratenic hosts include reptiles, amphibians, planarians. Definitive hosts are rodents, primarily rats (*Rattus*) and cotton rats (*Sigmodon*). Zoonotic in humans as a dead-end host harboring L4 larvae and young adults (Cowie 2013).

**Route of infection.** Ingestion of L3 larvae in infected mollusks or paratenic hosts (Cowie 2013).

**Site in human host.** CNS (Cowie 2013).

***Angiostrongyluscostaricensis* Morera & Cespedes, 1971**

**Geographic distribution.** Southern United States, Central and South America (Romero-Alegria 2014).

**Natural hosts.** Intermediate hosts are terrestrial mollusks. Definitive hosts are rodents. Zoonotic in humans (Romero-Alegria 2014).

**Route of infection.** Ingestion of L3 larvae in infected mollusks or contaminated produce (Romero-Alegria 2014).

**Site in human host.** Mesenteric blood vessels (Romero-Alegria 2014).

***Angiostrongylusmalaysiensis* Bhaibulaya & Cross, 1971**

*Angiostrongyluscantonensis* Malaysia strain

**Geographic distribution.** Southeast Asia (Rodpai et al. 2016).

**Natural hosts.** Intermediate hosts are terrestrial mollusks. Paratenic hosts include reptiles, amphibians, planarians. Definitive hosts are rodents, primarily rats. Apparently zoonotic in humans as a dead-end host harboring larvae (Rodpai et al. 2016; Watthanakulpanich et al. 2021).

**Route of infection.** Ingestion of L3 larvae in infected mollusks or paratenic hosts (Watthanakulpanich et al. 2021).

**Site in human host.** CNS (Watthanakulpanich et al. 2021).

●●●●●●●●● **Metastrongylidae Leiper, 1909**

**Genus *Metastrongylus* Molin, 1861**

***Metastrongyluselongatus* Dujardin, 1845**

*Strongylusapri* Gmelin, 1790

**Geographic distribution.** Worldwide (Calvopina et al. 2016).

**Natural hosts.** Intermediate hosts are earthworms. Definitive hosts are wild and domestic pigs. Zoonotic in humans (Calvopina et al. 2016).

**Route of infection.** Presumed ingestion of L3 larvae in infected earthwormsv

**Site in human host.** Lungs (Calvopina et al. 2016).

***Metastrongylussalmi* Gedoelst, 1923**

**Geographic distribution.** Worldwide (Calvopina et al. 2016).

**Natural hosts.** Wild and domestic swine are definitive hosts; one incidental infections in reported in a human (Calvopina et al. 2016).

**Route of infection.** Presumed ingestion of L3 larvae in infected earthworms.

**Site in human host.** Lungs (Calvopina et al. 2016).

### Excluded species

The following genera and species have been previously reported as human parasites. They are excluded from the above checklist because it is not believed they can cause parasitic infection in the human host, are based on demonstrable misidentifications, confirmation of identification is required, or because of taxonomic changes. The organisms are listed in alphabetical order.

***Agamomermis*** spp. Members of the genus *Agamomermis* are mermithid nematodes that are free-living as adults but infect insects as larvae. Human cases are believed to represent spurious passage following accidental ingestion of worms in contaminated food or water (Leon 1946; Chabaud and Lanz 1951).

***Amblyommaargentinae*** Neumann, 1905. This Neotropical tick was recorded as a human parasite under its synonym *A.testudinis* (Conil, 1877) (Doss 1974), but that record is believed to be in error (Guglielmone and Robbins 2018).

***Amblyommaauricularium*** (Conil, 1878). This Neotropical tick is a parasite of several groups of mammals and birds. Records of this species from humans are believed to represent misidentifications, primarily of *A.parvum* (Guglielmone and Robbins 2018).

***Amblyommacalcaris*** Nakatsudi, 1942. This tick was described from a human in China (Nakatsudi 1942). Apparently the species was not described properly and the name is invalid (Guglielmone and Robbins 2018)

***Amblyommacompressum*** (Macalister, 1872). This African tick species is a parasite of mammals, especially pangolins and rodents, birds, and reptiles. Records of this species from unknown locations in Africa (Theiler 1962) require confirmation (Guglielmone and Robbins 2018).

***Amblyommageayi*** Neumann, 1899. This tick is a parasite of various mammals and birds in Central and South America. A record of this species from a human (Esser et al. 2016) is based on conflicting host data in the paper and needs to be confirmed (Guglielmone and Robbins 2018).

***Amblyommahelvolum*** Koch, 1844. This Southeast Asian tick is primarily a parasite of reptiles, and occasionally mammals. Records of this species feeding on humans (Doss 1974) is based on an earlier publication that stated the ticks were merely crawling on humans (Audy 1960). To date, there are no records of *A.helvolum* feeding on humans (Guglielmone and Robbins 2018).

***Amblyommamacfarlandi*** Kierans et al., 1973. This tick is a parasite of tortoises on the Galapagos Islands. The single record from a human (Guglielmone et al. 2006) was not confirmed as feeding on the human host and may have merely been an incidental finding (Guglielmone and Robbins 2018).

***Amblyommapomposum*** Dönitz, 1909. This African tick is a parasite of several groups of mammals and birds. Records from humans require confirmation (Guglielmone and Robbins 2018).

***Amblyommasylvaticum*** (De Geer, 1778). This tick is a parasite of reptiles in South Africa. Records of this species from humans are believed to represent misidentifications (Guglielmone and Robbins 2018).

***Amblyommausingeri*** Keirans et al., 1973. This tick is a parasite of tortoises on the Galapagos Islands. The single record from a human (Guglielmone et al. 2006) was not confirmed as feeding on the human host and may have merely been an incidental finding (Guglielmone and Robbins 2018).

***Amphimermiselegans*** (Hagmeier, 1912). *Amphimermiselegans* is an Asian mermithid nematode parasitic on orthopteran insects. A human case (as *Mermis*) reportedly recovered from urine probably represents pseudoparasitism or contamination of the toilet by the insect host (Hasegawa et al. 1996).

***Androlaelapscasalis*** (Berlese, 1887). This laelapid mite is a predator on other mites and insects. It was reported as a cause of human dermatitis in Israel (Rosen et al. 2002). The mouthparts of *A.casalis* are not adapted for piercing vertebrate skin and the dermatitis in that report may have been associated with *Dermanyssusgallinae*, which was also reported by the authors and is a host for *A.casalis*. More research is needed to determine if *A.casalis* can bite humans and be a cause of dermatitis.

***Anisopus* sp.** Anisopodid flies are commonly called wood gnats or window gnats. They have been implicated in intestinal and urogenital myiasis (Smith and Taylor 1966). The larvae of anisopodids breed in decaying vegetation, fermenting sap, animal manure, tree holes, mud, and sewage. Their presence in toilets, latrines, and similar should be regarded as incidental as they feed on detritus in these substrates.

***Bertiellasatyri*** Blanchard, 1891. This cestode was originally described from orangutans. Reports from humans (Chandler 1925) are believed to represent misidentifications of *B.studeri* (Sapp and Bradbury 2020).

***Caccobiusvulcanus*** (Fabricius, 1801). *Caccobiusvulcanus* is a Palearctic coprophagous scarab beetles that has been implicated as a cause of scarabiasis in India (as *C.mutans* Sharp, 1875) (Iyengar 1928). Scarabiasis is a proposed phenomenon describing the colonization of the human intestinal tract with coprophagous scarab beetles, usually based on the finding of beetles in the soiled diapers of children. There is no pathology described for parasitism by scarab beetles and the phenomenon probably represents post-defecation contamination of diapers, toilets, latrines, and similar.

***Clogmiaalbipunctata*** (Williston, 1893). *Clogmiaalbipunctata* is a psychodid fly with a nearly worldwide distribution. This species has been implicated as a cause of urogenital (El-Dib et al. 2017; Farrag et al. 2019), intestinal (Tu et al. 2007; Mokhtar et al. 2016a), and nasopharyngeal (Nevill et al. 1969; Mohammed and Smith 1976) myiasis, sometimes under the name *Telmatoscopusalbipuntatus*. Psychodid flies reported as causative agents of urogenital and intestinal myiasis is usually due to the incidental finding of the flies in toilets, latrines, sinks, and bathtubs. The larvae of psychodid flies breed in biofilms, including in faucets and drains, giving the false impression they were shed in stool or urine. There is no evidence that psychodid fly larvae use human tissue as a nutritive source.

***Crasodactyluspunctatus*** Guérin-Méneville, 1847. This carabid beetle was reported from the ear of two patients in Oman (misspelled as *Crasydactyluspunctatus*) (Bhargava and Victor 1911). This beetle is predaceous on other insects and small invertebrates and its presence in the human ear canal should be regarded as incidental.

***Cryptostrongyluspulmoni***. The name *Cryptostrongyluspulomoni* is a provisional name given to suspect helminths associated with chronic fatigue syndrome (Klapow 1999). Images depicting this ‘parasite’ appear to be synthetic fibers. There is no evidence that *C.pulmoni* represents an actual animal, let alone one capable of parasitizing humans.

***Cyclocephalaborealis*** Arrow, 1911. This scarab beetle was implicated in a large-scale infestation of the ears of Boy Scouts in Pennsylvania, USA (Maddock and Fehn 1958). *Cyclocephala* species are phytophagous, and its presence in the human ear canal should be regarded as incidental.

***Dermacentorcruentus*** Koch, 1844. This European tick was listed as a human parasite based on the original description (Doss 1974), but there is no indication in the original description it was observed feeding on humans (Guglielmone and Robbins 2018).

***Dermacentorhalli*** McIntosh, 1931. This tick is a parasite of various mammals in North and Central America. Records from humans require confirmation (Guglielmone and Robbins 2018).

***Dermacentortaiwanensis*** Sugmito, 1935. This tick is a parasite of various mammals in China, Vietnam, and Taiwan. Records of this species from humans are believed to represent misidentifications (Guglielmone and Robbins 2018).

***Dibothriocephalusalascense*** (Rausch & Rausch, 1956). This species was originally described from a domestic dog in the Yukon-Kuskokwim Delta of Canada. A single human case of this species was reported from an Eskimo in Alaska (Rausch et al. 1967), but the record is considered doubtful (Scholz and Kuchta 2016; Waeschenbach et al. 2017).

***Diphyllobothriumcameroni*** Rausch, 1969. This species was originally described from the Hawaiian monk seal (*Neomonachusschauinslandi*) and has been recorded twice from humans in Japan (Kamo H. 1986), but the records are considered doubtful (Scholz and Kuchta 2016; Waeschenbach et al. 2017).

***Diphyllobothriumelegans*** (Krabbe, 1865). This is a parasite of seals and has been described once from a human in Japan (Kamo H. 1986), but the record is considered doubtful (Scholz and Kuchta 2016; Waeschenbach et al. 2017).

***Diphyllobothriumorcini*** Hatsushika & Shirouzu, 1990. This species was described from killer whales. There are two records from humans from Japan (Kifune 2000; Nakazawa 1992), but those records are considered doubtful (Waeschenbach et al. 2017).

***Diphyllobothriumscoticum*** (Rennie & Reid, 1912). This species was described from the leopard seal (*Hydrurgaleptonyx*) and has been recorded once from a human in Japan (Fukumoto 1988), but that record is considered doubtful (Waeschenbach et al. 2017).

***Drosophilamelanogaster*** Meigen, 1830. This common ‘fruit fly’ has been infrequently reported as a cause of nasal and ocular myiasis (Francesconi and Lupi 2012). Larvae breed in decaying vegetation and their presence in clinical specimens should be considered incidental.

***Dryomyzaformosa*** (Wiedemann, 1830). This dryomyzid fly was reported as a cause of gastrointestinal myiasis in a patient from Japan suffering from delusional parasitosis. Larvae were observed in fresh stool and it was speculated they represent spurious passage following accidental ingestion of the larvae (Chigusa et al. 2000). Dryomyzid larvae feed on decaying organic material and there is no evidence they use human intestinal tissue as a nutritive source.

***Emysorbicularis*** (Linnaeus, 1758). *Emysorbicularis* is the Latin name of the European pond turtle. This name was used in the 23^rd^ edition of “Manson’s Tropical Diseases” (Farrar 2014) as a species of leech that parasitizes humans. This probably represents an editorial error and may have been intended to refer to a leech that normally parasitizes the turtle.

***Eristalistenax*** (Linnaeus, 1758). This syrphid fly is frequently implicated in causing intestinal or urogenital myiasis (Francesconi and Lupi 2012). The larvae breed in decaying organic substrates, including manure, sewage, and contaminated water. Most reported cases are from the finding of larvae in latrines, outhouses, toilets, and similar and their presence in such locations is believed to be incidental.

***Euparyphium* spp.** Members of this genus of echinostome flukes has been recorded from humans in Laos (Toledo and Esteban 2016); however, the identity of human isolates attributed to *Euparyphium* should be confirmed (Chai et al. 2012).

***Fanniascalaris*** (Fabricius, 1794). This fly was reported as a cause of urogenital myiasis in 1975 (Werner et al. 1975; Francesconi and Lupi 2012). The specimen was reportedly recovered in urine and likely represents contamination of a toilet, latrine, or the specimen itself.

***Gordius*** spp. A number of “gordiid worms” or “horsehair worms”, including *Gordius*, have been recovered from humans (typically in vomitus) (Kagei et al. 1966; Uchikawa et al. 1987; Herter and Nesse 1989; Lee et al. 2003). These most likely represent spurious passage following accidental ingestion of infected arthropod hosts or worms free in the environment. The finding of such worms in toilets is usually due to the drowning of the insect host by the parasite and liberation of the parasite therefrom.

***Haemaphysaliscinnabarina*** Koch, 1844. Records of this Brazilian tick from humans refer to *H.chordeilis* (Guglielmone and Robbins 2018).

***Haemaphysaliskashmirensis*** Hoogstraal & Varma, 1962. This tick is a parasite of mammals and reptiles in India and Pakistan. Records from humans require confirmation (Guglielmone and Robbins 2018).

***Haemaphysalismuhsamae*** Santos Dias, 1954. This tick is a parasite of birds in Africa. Records of this species from humans require confirmation due to the morphologic challenges in identifying this species (Guglielmone and Robbins 2018).

***Haemaphysalisproxima*** Aragão, 1911. This species has been recorded from humans in Colombia. There is no formal description for this species and the name is considered *nomen nudum* (Guglielmone and Robbins 2018). Records of this species from humans probably pertain to *H.leporispalustris* (Guglielmone and Robbins 2018).

***Haemaphysaliswarburtoni*** Nuttall, 1912. This tick is a parasite of bovids and rodents in India, Nepal, and China. Records from humans may be based on misidentifications and require confirmation (Guglielmone and Robbins 2018).

***Hyalommafranchinii*** Tonelli-Rondelli, 1932. This tick is a parasite of mammals and reptiles in North Africa and the Middle East. Records from humans are based on misidentifications of *H.excavatum* and *H.marginatum* (Guglielmone and Robbins 2018).

***Hyalommaimpressum*** Koch, 1844. This tick is a parasite of various mammals and birds from sub-Saharan Africa. This species has been recorded from a human in South Africa (Bedford 1927); however, *H.impressum* does not occur in South Africa and that record may represent a misidentification (Guglielmone and Robbins 2018).

***Hyalommaplubeum*** (Panzer, 1795). This name is currently considered incertae sedis, and records of *H.plumbeum* from humans currently pertain to *H.marginatum* (Guglielmone and Robbins 2018).

***Hermetia* spp.** Larvae of these soldier flies have been implicated in cases of intestinal and furuncular myiasis (Francesconi and Lupi 2012). Larvae of *Hermetia* spp. breed in decaying organic material, including outhouses. Their presence in clinical specimens is probably incidental and it is not believed they cause myiasis.

***Ixodesaffinis*** Neumann, 1899. This North and Central American tick is primarily a parasite of a wide variety of mammals, including carnivores, marsupials, and ungulates. The species is difficult to identify morphologically and past records of this species from humans are believed to be misidentifications (Guglielmone and Robbins 2018).

***Ixodeshumanus*** Koch, 1844. This species was described from a human in Brazil, however the identity of the species is not fully understood and it may be synonymous with a member of the genus *Amblyomma* (Guglielmone and Robbins 2018).

***Ixodesjellisoni*** Cooley & Kohls, 1938. This is a North American tick found on rodents and carnivores. Records of this species from humans are believed to be misidentifications (Guglielmone and Robbins 2018).

***Ixodeslaysanensis*** Wilson, 1964. This tick is a bird parasite in Hawaii. Records of this species feed in humans (Doss 1974) are based on an earlier record that reported the tick crawling, and not feeding, on humans. To date, there is no evidence *I.laysanensis* feeds on humans (Guglielmone and Robbins 2018).

***Ixodesloricatus*** Neumann, 1899. This tick is a parasite of marsupials and rodents in South America. Records from humans in Brazil (Serra-Freire 2011) require confirmation and are provisionally excluded as a parasite of humans (Guglielmone and Robbins 2018).

***Ixodesluciae*** Sénevet, 1940. This species is a Neotropical tick of various mammals, especially marsupials and rodents. The single record of this species on a human from Argentina (Ivancovich 1992) is believed to be based on a misidentification (Guglielmone and Robbins 2018).

***Ixodesmolestus*** James, 1923. This species was described as attacking humans in the USA, but the name is currently regarded as *nomen dubium* (Guglielmone and Robbins 2018).

***Ixodessimplex*** Neumann, 1906. Records of this broadly-distributed Old World tick species of bats on humans from Japan cannot be confirmed and may be based on misidentifications (Guglielmone and Robbins 2018).

***Ixodestrichosuri*** Roberts, 1960. This tick species is a parasite of marsupials and rodents in Australia. Records of this parasite infecting humans (Russell 2001) are based on unpublished records that require confirmation. Until such time, this species is removed from the list of ticks parasitizing humans (Guglielmone and Robbins 2018).

***Lasiodermaserricorne*** (Fabricius, 1792). *Lasiodermaserricorne* is a cosmopolitan pest of dried, organic materials, including tobacco, cereals and other grains, dried fruit, and dried animal products. It was been reported as cause of canthariasis in infants in China (Sun et al. 2016) and Malaysia (Mokhtar et al. 2016b). Given the beetle’s predilection for food products, and the age of the patients, this probably represents spurious passage following consumption of contaminated food.

***Ligula* spp.***Ligula* species are diphyllobothriid parasites of fish-eating birds. There are two reports from humans, initially reported under the names *Diplogonophorusbrauni* Leon, 1907 and *Brauniajassyensis* Leon, 1908. These records are believed to represent misidentifications (Scholz and Kuchta 2016).

***Lophomonasblattarum*** Stein, 1860. There are many case reports in the literature of *L.blattarum* being isolated from human respiratory specimens. These all appear to be misidentifications of ciliocytophthoria, a condition whereby detached, motile epithelial cells are observed in clinical specimens (Hadziyannis et al. 2000; Li and Gao 2016). *Lophomonasblattarum* is a commensal in the gut of cockroaches and not a proven agent of human infection.

***Maladeracastanea*** (Arrow, 1913). This scarab beetle was implicated in a large-scale infestation of the ears of Boy Scouts in Pennsylvania, USA (as *Autosericacastanea*) (Maddock and Fehn 1958). *Maladera* species are phytophagous, and its presence in the human ear canal should be regarded as incidental.

***Melophagusovinus*** (Linnaeus, 1758). Commonly called the ‘sheep ked’, *M.ovinus* is a cosmopolitan parasite of domestic sheep (*Ovusaries*), as well as wild ungulates, rabbits, and wild and domestic canids. It has been reported as parasitizing humans (Zhao et al. 2018), however there is no evidence it can survive on human blood.

***Muscanebulo*** Fabricius, 1794. This species was reported as cause of oral myiasis in India (Sharma et al. 2008). *Muscanebulo* is generally considered a synonym of *M.curviforceps* Saccà & Rivosecchi, 1956, which itself is considered a subspecies or synonym of *M.domestica*. Also, *M.curviforceps* is considered endemic to sub-Saharan Africa (Marquez and Krafsur 2002), which should preclude it from causing myiasis in India.

***Muscina* spp.** Flies in the genus *Muscina* have repeatedly been reported as causing intestinal myiasis following the recovery of fly larvae in stool (Francesconi and Lupi 2012). This probably represents post-defecation contamination of the stool specimen. There is no evidence *Muscina* uses human tissue as a nutritive source.

***Onthophagusbifasciatus*** (Fabricius, 1781). *Onthophagusbifasciatus* is a Palearctic dung beetle that has been implicated as a causative agent of scarabiasis in India (Iyengar 1928). Scarabiasis is a proposed phenomenon describing the colonization of the human intestinal tract with coprophagous scarab beetles, usually based on the finding of beetles in the soiled diapers of children. There is no pathology described for parasitism by scarab beetles and the phenomenon probably represents post-defecation contamination of diapers, toilets, latrines, and similar.

***Onthophagusunifasciatus*** (Schaller, 1783). *Onthophagusunifasciatus* is a Palearctic dung beetle that has been implicated as a causative agent of scarabiasis in Sri Lanka (Iyengar 1928; Gunawardena 1963). Scarabiasis is a proposed phenomenon describing the colonization of the human intestinal tract with coprophagous scarab beetles, usually based on the finding of beetles in the soiled diapers of children. There is no pathology described for parasitism by scarab beetles and the phenomenon probably represents post-defecation contamination of diapers, toilets, latrines, and similar.

***Palpodascutellaris*** (Fabricius, 1805). This is a species of syrphid fly from Central and northern South America. It was reported as the cause of human intestinal myiasis in Costa Rica (Pérez-Bañón et al. 2020). The larvae breed in decaying organic substrates, including manure, sewage, and contaminated water. Most reported cases are from the finding of larvae in latrines, outhouses, toilets, and similar and their presence in such locations is believed to be incidental.

***Parachordodes* spp.** A number of “gordiid worms” or “horsehair worms”, including *Parachordodes*, have been recovered from humans (typically in vomitus) (Yamada et al. 2012). These most likely represent spurious passage following accidental ingestion of infected arthropod hosts or worms free in the environment. The finding of such worms in toilets is usually due to the drowning of the insect host by the parasite and liberation of the parasite therefrom.

***Paragordiusvarius*** (Leidy, 1851). A number of “gordiid worms” or “horsehair worms”, including *Paragordius*, have been recovered from humans (typically in vomitus) (Ali-Khan and Ali-Khan 1977). These most likely represent spurious passage following accidental ingestion of infected arthropod hosts or worms free in the environment. The finding of such worms in toilets is usually due to the drowning of the insect host by the parasite and liberation of the parasite therefrom.

***Pericoma* spp.***Pericoma* is a genus of Psychodidae that has been implicated as a cause of urinary myiasis in India (Singla et al. 2018). Psychodid flies reported as causative agents of urogenital and intestinal myiasis is usually due to the incidental finding of the flies in toilets, latrines, sinks, and bathtubs. The larvae of psychodid flies breed in biofilms, including in faucets and drains, giving the false impression they were shed in stool or urine. There is no evidence psychodid fly larvae use human tissue as a nutritive source.

***Piophilacasei*** (Linnaeus, 1758). This species, commonly called the ‘cheese fly’, has been infrequently reported as a cause of intestinal or urogenital myiasis (Peckenscneider et al. 1952; Saleh and el Sibae 1993). The larvae of this fly breeds in foodstuffs, and its presence in toilets and latrines should be considered incidental, probably following spurious passage of the larvae after eating infested food.

***Psychodaalbipennis*** Zetterstedt, 1850. This psychodid has been implicated as an agent of urogenital myiasis (Francesconi and Lupi 2012; Shimpi et al. 2018). Psychodid flies reported as causative agents of urogenital and intestinal myiasis is usually due to the incidental finding of the flies in toilets, latrines, sinks, and bathtubs. The larvae of psychodid flies breed in biofilms, including in faucets and drains, giving the false impression they were shed in stool or urine. There is no evidence psychodid fly larvae use human tissue as a nutritive source.

***Psychodaalternata*** Say, 1824. This psychodid has been implicated as an agent of urogenital myiasis (Abul Hab 2001) and has been recorded from human sputa (Scott 1964). Psychodid flies reported as causative agents of urogenital and intestinal myiasis is usually due to the incidental finding of the flies in toilets, latrines, sinks, and bathtubs. The larvae of psychodid flies breed in biofilms, including in faucets and drains, giving the false impression they were shed in stool or urine. There is no evidence psychodid fly larvae use human tissue as a nutritive source.

***Psychodasexpunctata*** Curtis, 1839. This psychodid has been implicated as a source of gasterointestinal myiasis (Okada 1927). Psychodid flies reported as causative agents of urogenital and intestinal myiasis is usually due to the incidental finding of the flies in toilets, latrines, sinks, and bathtubs. The larvae of psychodid flies breed in biofilms, including in faucets and drains, giving the false impression they were shed in stool or urine. There is no evidence psychodid fly larvae use human tissue as a nutritive source.

***Rhipicentorbicornis*** Nutall & Warburton, 1908. This tick is a parasite of various mammals in sub-Saharan Africa. This species was recorded as a parasite on humans (Doss 1974), however that record is based on a misunderstanding that a tick observed in a human dwelling meant it feeds on humans. To date, there are no records of *R.bicornis* feeding on humans (Guglielmone and Robbins 2018).

***Sappiniadiploidea*** (Hartmann & Naegler, 1908). The first case of human infection with a member of the genus *Sappinia* was reported to have been caused by *S.diploidea* based on morphologic criteria (Gelman et al. 2001). However, molecular characterization later demonstrated the species to be *S.pedata* (Qvarnstrom et al. 2009).

***Scaritessulcatus*** Olivier, 1795. *Scaritessulcatus* is a Palearctic ground beetle that has been implicated as a cause of genital canthariasis (Paul 2007). *Scarites* species are free-living predaceous beetles. Given the habits of these beetles, this case probably represents environmental contamination or possibly urethral sounding.

***Scenopinus* spp.** Members of this genus of flies have been implicated in urogenital myiasis (Thompson et al. 1970). Larvae of *Scenopinus* are predators on the larvae of other insects. The presence of *Scenopinus* larvae in clinical specimens is probably incidental.

***Trichophryapiscium*** Bütschli, 1899. This freshwater fish pathogen was reported was reported from sinus aspirates of a patient in Iraq (Al-Duboon and Disher 2018). Images of the suspect organism in the publication showed this to be a misidentification of ciliocytophthoria, a condition whereby detached, motile epithelial cells are observed in clinical specimens (Hadziyannis et al. 2000; Li and Gao 2016).

***Tyroglyphuslongior*** (Gervais, 1844). This grain mite has been implicated in intestinal acariasis by the finding of mites in the stool of two patients with generalized intestinal complaints (Harold Hinman and Kampmeier 1934). The finding of these mites probably represents spurious passage after incidental ingestion of the mites in contaminated foodstuffs.

***Tyroglyphusputrescentiae*** (Schrank, 1781). This grain and mold mite has been implicated as a cause of intestinal acariasis (Khalifa et al. 2016). The finding of these mites probably represents spurious passage after incidental ingestion of the mites in contaminated foodstuffs.

***Urbanorum***. The name ‘*Urbanorum*’ has been given to usual objects observed in stool specimens. Most cases have been reported from Central and South America (de Aguiar and Alves 2018). Generally referred to as protozoans, there does not appear to be any formal description of *Urbanorum* in the zoological literature and the general consensus is that these objects are nothing more than peculiar artifacts. No biochemical, ultrastructural, or genetic studies have been undertaken to confirm its status as a living organism, although the exact identity of it has not been ascertained.
